# Supplementary material for: Body mass index and all-cause mortality in HUNT and UK biobank studies: revised non-linear Mendelian randomisation analyses
Source: BMJ Open. 2024 May 15;14(5):e081399. doi: 10.1136/bmjopen-2023-081399 (PMC11097829; doi:10.1136/bmjopen-2023-081399)
Supplement: Supplementary data [file bmjopen-2023-081399supp001.pdf]

SUPPLEMENTARY MATERIAL

We here perform a simulation study investigating whether adjusting genetic associations for factors that influence selection reduces selection bias within strata in a non-linear Mendelian randomization framework. We generate simulated data for 1,000,000 individuals on a genetic variant *G*, an exposure *X*, an outcome *Y*, and competing risk factors *U* and *V*. For simplicity, all variables are modelled as continuous and normally distributed.

We consider two scenarios. In each scenario, the genetic variant is a valid instrumental variable in the population as a whole, and there is no true effect of the exposure on the outcome. This implies that the genetic variant should not be associated with the outcome. We estimate the association between the genetic variant and the outcome in a selected subset of the population.

In scenario 1, the data-generating model is:

$$G_i \sim Normal(0,1)$$
$$U_i \sim Normal(0,1)$$
$$V_i \sim Normal(0,1)$$
$$X_i \sim Normal(0.2\ G_i + 0.4\ U_i + 0.4V_i, 1)$$
$$Y_i \sim Normal(-0.5\ U_i + 0.2\ V_i, 1)$$

Participants (indexed by the *i* subscript) are selected into the sample with probability $expit(-2 + U_i + X_i)$, where $expit(x) = \frac{\exp(x)}{1+\exp(x)}$ is the inverse of the logistic (logit) function. Here *U* and *V* are both confounders of the relationship between the exposure and outcome, and selection depends on both *U* and *X*. We compare genetic associations with the outcome with and without adjustment for *U*.

In scenario 2, the data-generating model is:

$$G_i \sim Normal(0,1)$$
$$V_i \sim Normal(0,1)$$
$$X_i \sim Normal(0.2\ G_i + 0.8\ V_i, 1)$$
$$U_i \sim Normal(X_i, 1)$$
$$Y_i \sim Normal(0.5\ V_i, 1)$$

Participants are selected into the sample with probability $expit(-2 + U_i)$. Here *V* is a confounder (common cause) of the relationship between the exposure and outcome, and *U* is a downstream effect of the exposure *X*, and hence *U* is a collider (common effect) of *G* and *V*. Selection depends on *U* only. Again, we compare genetic associations with the outcome with and without adjustment for *U*.

In both cases, all normal distributions are independent. We simulate 1000 datasets for each scenario, and compare the mean estimate of the genetic association with the outcome and its empirical Type 1 error rate at a 5% significance level (that is, the proportion of 95% confidence intervals excluding the null). Around 15-20% of the 1,000,000 participants are selected in each simulated dataset.

In scenario 1, the mean association of the genetic variant with the outcome without adjustment for *U* was 0.014 (mean standard error 0.002), and the empirical Type 1 error rate was 100.0%. With adjustment for *U*, the mean association was -0.002 (mean standard error 0.002), and the empirical Type 1 error rate was 17.1%.

In scenario 2, the mean association of the genetic variant with the outcome without adjustment for *U* was -0.010 (mean standard error 0.002), and the empirical Type 1 error rate was 97.9%. With

Burgess S, *et al. BMJ Open* 2024; 14:e081399. doi: 10.1136/bmjopen-2023-081399

adjustment for *U*, the mean association was -0.030 (mean standard error 0.002), and the empirical Type 1 error rate was 100.0%.

In scenario 1, bias reduced considerably on adjustment for the factor influencing selection. Mean bias did not reduce to zero, as selection also depends on the exposure. We note that if selection did not additionally depend on the exposure, there would not be selection bias, as *U* is a confounder not a collider. However, even in this case with imperfect adjustment, the reduction in bias was substantial. In scenario 2, there was bias without adjustment for the collider *U*, but mean bias sharpened (got worse) on adjustment for *U*. Scenario 1 is more likely to be representative of the situation we see in the paper, as sex and age cannot be consequences of the exposure, but are likely to be confounders.

This simulation study is deliberately designed to be relatively simple and limited in scope, as we do not believe that any simulation study would be able to prove conclusively that covariate adjustment eliminates selection bias in all cases where selection depends on a confounder. Indeed, we do not make that claim; rather we claim that adjustment for confounders that are key predictors of selection can ameliorate bias due to selection. There will always be intrinsic uncertainty in practice, as we do not know all the factors that influence selection, and we would be cautious to adjust for too many variables in a Mendelian randomization analysis, particularly for variables that could be mediators on the causal pathway from the exposure to the outcome, or could be colliders. As age and sex cannot logically be caused by the exposure, they can be safely adjusted for without risk of these variables being mediators or colliders [S1]. Hence, if age and sex are strong predictors of selection into the sample, then adjustment for age and sex should reduce selection bias in Mendelian randomization investigations.

We believe that any residual bias from selection is likely to be low, in line with previous simulation studies that have shown selection effects need to be substantial in magnitude to have non-negligible biasing effects on Mendelian randomization estimates [S2]. However, we acknowledge that this imposes an additional level of uncertainty on the results of a non-linear Mendelian randomization analysis, on top of the existing uncertainty in the Mendelian randomization paradigm due to the validity of the instrumental variable assumptions, and other factors. All methods for making causal inferences from observational data rely on untestable assumptions, and findings should be interpreted with appropriate caution.

SUPPLEMENTARY REFERENCES

S1. Burgess S, Davey Smith G, Davies NM, Dudbridge F, Gill D, Glymour MM, Hartwig FP, Kutalik Z, Holmes MV, Minelli C, Morrison JV, *et al*. Guidelines for performing Mendelian randomization investigations: update for summer 2023. Wellcome Open Research 2023; 4:186.

S2. Gkatzionis A, Burgess S. Contextualizing selection bias in Mendelian randomization: how bad is it likely to be? International Journal of Epidemiology. 2019; 48(3):691-701.

Supplementary Table 1: Genetic variants and associations with BMI based on European sex-combined analysis from GIANT [23]. Beta-coefficents are in standard deviation units.

SNP	Effect allele*	Other allele	Beta-coefficient	Standard error of beta	Number of subjects	*P*-value
rs1558902	A	T	0.082	0.003	320 073	7.51E-153
rs6567160	C	T	0.056	0.004	321 958	3.93E-53
rs10938397	G	A	0.040	0.003	320 955	3.21E-38
rs543874	G	A	0.048	0.004	322 008	2.62E-35
rs2207139	G	A	0.045	0.004	322 019	4.13E-29
rs11030104	A	G	0.041	0.004	322 103	5.56E-28
rs3101336	C	T	0.033	0.003	316 872	2.66E-26
rs7138803	A	G	0.032	0.003	322 092	8.15E-24
rs10182181	G	A	0.031	0.003	321 759	8.78E-24
rs3888190	A	C	0.031	0.003	321 930	3.14E-23
rs1516725	C	T	0.045	0.005	320 644	1.89E-22
rs12446632	G	A	0.040	0.005	316 758	1.48E-18
rs2287019	C	T	0.036	0.004	300 921	4.59E-18
rs3817334	T	C	0.026	0.003	321 959	5.15E-17
rs2112347	T	G	0.026	0.003	322 019	6.19E-17
rs12566985	G	A	0.024	0.003	319 282	3.28E-15
rs3810291	A	G	0.028	0.004	296 261	4.81E-15
rs7141420	T	C	0.024	0.003	321 970	1.23E-14
rs13078960	G	T	0.030	0.004	322 135	1.74E-14
rs10968576	G	A	0.025	0.003	322 061	6.61E-14
rs17024393	C	T	0.066	0.009	297 874	7.03E-14
rs657452	A	G	0.023	0.003	313 651	5.48E-13
rs12429545	A	G	0.033	0.005	312 934	1.09E-12
rs12286929	G	A	0.022	0.003	321 903	1.31E-12
rs13107325	T	C	0.048	0.007	321 461	1.83E-12
rs11165643	T	C	0.022	0.003	320 730	2.07E-12
rs7903146	C	T	0.023	0.003	322 130	1.11E-11
rs10132280	C	A	0.023	0.003	321 797	1.14E-11
rs17405819	T	C	0.022	0.003	322 085	2.07E-11
rs1016287	T	C	0.023	0.003	321 969	2.25E-11
rs4256980	G	C	0.021	0.003	320 028	2.90E-11
rs17094222	C	T	0.025	0.004	321 770	5.94E-11
rs12401738	A	G	0.021	0.003	322 070	1.15E-10
rs7599312	G	A	0.022	0.003	322 024	1.17E-10
rs2365389	C	T	0.020	0.003	316 768	1.63E-10
rs205262	G	A	0.022	0.004	315 542	1.75E-10
rs2820292	C	A	0.020	0.003	321 707	1.83E-10
rs12885454	C	A	0.021	0.003	320 823	1.94E-10
rs16851483	T	G	0.048	0.008	233 929	3.55E-10
rs1167827	G	A	0.020	0.003	306 238	6.33E-10
rs758747	T	C	0.023	0.004	308 688	7.47E-10
rs1928295	T	C	0.019	0.003	321 979	7.91E-10
rs9925964	A	G	0.019	0.003	318 385	8.11E-10

Burgess S, *et al. BMJ Open* 2024; 14:e081399. doi: 10.1136/bmjopen-2023-081399

rs11126666	A	G	0.021	0.003	321 979	1.33E-09
rs2650492	A	G	0.021	0.004	319 464	1.92E-09
rs6804842	G	A	0.019	0.003	321 463	2.48E-09
rs12940622	G	A	0.018	0.003	322 032	2.49E-09
rs11847697	T	C	0.049	0.008	306 243	3.99E-09
rs4740619	T	C	0.018	0.003	321 887	4.56E-09
rs13191362	A	G	0.028	0.005	321 902	7.34E-09
rs3736485	A	G	0.018	0.003	321 398	7.41E-09
rs17001654	G	C	0.031	0.005	233 722	7.76E-09
rs11191560	C	T	0.031	0.005	321 893	8.45E-09
rs1528435	T	C	0.018	0.003	321 924	1.20E-08
rs2075650	A	G	0.026	0.005	308 408	1.25E-08
rs1000940	G	A	0.019	0.003	321 836	1.28E-08
rs2033529	G	A	0.019	0.003	321 917	1.39E-08
rs11583200	C	T	0.018	0.003	322 095	1.48E-08
rs9400239	C	T	0.019	0.003	321 988	1.61E-08
rs10733682	A	G	0.017	0.003	320 727	1.83E-08
rs11688816	G	A	0.017	0.003	322 051	1.89E-08
rs11057405	G	A	0.031	0.006	314 111	2.02E-08
rs2121279	T	C	0.025	0.004	322 065	2.31E-08
rs29941	G	A	0.018	0.003	321 970	2.41E-08
rs11727676	T	C	0.036	0.006	296 401	2.55E-08
rs3849570	A	C	0.019	0.003	284 339	2.60E-08
rs6477694	C	T	0.017	0.003	322 048	2.67E-08
rs7899106	G	A	0.040	0.007	321 770	2.96E-08
rs2176598	T	C	0.020	0.004	316 848	2.97E-08
rs2245368	C	T	0.032	0.006	205 675	3.19E-08
rs17724992	A	G	0.019	0.004	319 588	3.42E-08
rs7243357	T	G	0.022	0.004	322 107	3.86E-08
rs1808579	C	T	0.017	0.003	322 032	4.17E-08

*BMI-increasing allele

Supplementary Table 2: ICD-10 codes for cause-specific mortality outcomes

Cause-specific mortality	ICD-10 codes
Cardiovascular	G45, I01, I03-I82, I87, I95-I99, F01, Q20-Q28, R96
Cancer	C00-C97, D00-D48
Non-cardiovascular non-cancer	All others

Supplementary Table 3: Distribution of BMI (kg/m^2) in strata defined by the doubly-ranked stratification method

Stratum number	HUNT			UK Biobank		
	Mean	Lower decile	Upper decile	Mean	Lower decile	Upper decile
1	18.4	17.5	19.8	18.6	17.8	20.0
2	19.3	18.2	20.4	19.6	18.5	20.7
3	19.9	18.8	20.8	20.1	19.1	21.1
4	20.3	19.4	21.1	20.5	19.6	21.5
5	20.6	19.7	21.5	20.9	20.0	21.8
6	20.9	20.1	21.7	21.1	20.3	22.0
7	21.1	20.3	21.9	21.4	20.6	22.3
8	21.3	20.5	22.1	21.6	20.8	22.5
9	21.5	20.7	22.3	21.8	21.0	22.7
10	21.7	20.9	22.5	22.0	21.2	22.8
11	21.8	21.1	22.7	22.2	21.4	23.0
12	22.0	21.2	22.8	22.4	21.6	23.2
13	22.1	21.4	22.9	22.5	21.7	23.3
14	22.3	21.5	23.1	22.7	21.9	23.5
15	22.4	21.7	23.2	22.8	22.0	23.7
16	22.5	21.8	23.3	23.0	22.2	23.8
17	22.7	21.9	23.5	23.1	22.3	23.9
18	22.8	22.0	23.6	23.2	22.4	24.1
19	22.9	22.1	23.7	23.4	22.6	24.2
20	23.0	22.2	23.8	23.5	22.7	24.3
21	23.1	22.4	23.9	23.6	22.8	24.4
22	23.2	22.5	24.0	23.7	22.9	24.5
23	23.3	22.6	24.1	23.8	23.0	24.7
24	23.4	22.7	24.2	24.0	23.2	24.8
25	23.6	22.8	24.3	24.1	23.3	24.9
26	23.6	22.9	24.5	24.2	23.4	25.0
27	23.8	23.0	24.5	24.3	23.5	25.2
28	23.8	23.1	24.7	24.4	23.6	25.3
29	23.9	23.1	24.8	24.5	23.7	25.4
30	24.0	23.2	24.8	24.6	23.8	25.5
31	24.1	23.3	25.0	24.7	23.9	25.6
32	24.2	23.4	25.0	24.8	24.0	25.7
33	24.3	23.5	25.1	24.9	24.1	25.8
34	24.4	23.6	25.2	25.0	24.2	25.9
35	24.5	23.7	25.3	25.1	24.3	26.0
36	24.6	23.8	25.4	25.2	24.4	26.1
37	24.7	23.8	25.5	25.3	24.5	26.2
38	24.8	23.9	25.6	25.4	24.6	26.3
39	24.8	24.0	25.7	25.5	24.7	26.4
40	24.9	24.1	25.8	25.6	24.8	26.6
41	25.0	24.2	25.9	25.7	24.9	26.7
42	25.1	24.3	26.0	25.8	25.0	26.8
43	25.2	24.3	26.1	25.9	25.1	26.9
44	25.3	24.4	26.2	26.0	25.1	27.0
45	25.4	24.5	26.3	26.1	25.3	27.1

46	25.5	24.6	26.4	26.2	25.4	27.2
47	25.6	24.7	26.4	26.3	25.4	27.3
48	25.6	24.8	26.6	26.5	25.5	27.4
49	25.7	24.8	26.7	26.6	25.6	27.5
50	25.8	25.0	26.8	26.7	25.7	27.6
51	25.9	25.0	26.9	26.8	25.8	27.7
52	26.0	25.1	26.9	26.9	25.9	27.8
53	26.1	25.2	27.0	27.0	26.0	28.0
54	26.2	25.3	27.1	27.1	26.1	28.1
55	26.3	25.4	27.2	27.2	26.2	28.2
56	26.4	25.5	27.3	27.3	26.3	28.3
57	26.5	25.5	27.4	27.4	26.4	28.4
58	26.6	25.6	27.5	27.5	26.5	28.6
59	26.7	25.7	27.6	27.6	26.6	28.7
60	26.8	25.8	27.7	27.7	26.7	28.8
61	26.9	25.9	27.9	27.9	26.8	28.9
62	27.0	26.0	27.9	28.0	26.9	29.1
63	27.1	26.1	28.1	28.1	27.0	29.2
64	27.2	26.2	28.2	28.2	27.1	29.3
65	27.3	26.3	28.3	28.3	27.2	29.5
66	27.4	26.4	28.4	28.5	27.3	29.6
67	27.5	26.5	28.6	28.6	27.5	29.8
68	27.6	26.6	28.7	28.7	27.6	29.9
69	27.7	26.7	28.8	28.9	27.7	30.0
70	27.9	26.8	28.9	29.0	27.8	30.2
71	28.0	26.9	29.0	29.1	28.0	30.4
72	28.1	27.0	29.1	29.3	28.1	30.5
73	28.2	27.1	29.4	29.4	28.2	30.7
74	28.3	27.2	29.5	29.6	28.4	30.9
75	28.5	27.4	29.6	29.8	28.5	31.1
76	28.6	27.5	29.8	29.9	28.7	31.2
77	28.7	27.6	29.9	30.1	28.8	31.4
78	28.9	27.8	30.1	30.3	28.9	31.6
79	29.0	27.9	30.3	30.4	29.1	31.8
80	29.2	28.0	30.5	30.6	29.3	32.1
81	29.4	28.2	30.7	30.8	29.4	32.3
82	29.5	28.3	30.9	31.0	29.6	32.6
83	29.7	28.4	31.1	31.3	29.8	32.8
84	29.9	28.6	31.3	31.5	30.0	33.0
85	30.1	28.8	31.5	31.7	30.2	33.3
86	30.3	28.9	31.8	32.0	30.4	33.7
87	30.6	29.1	32.0	32.3	30.6	34.0
88	30.8	29.3	32.3	32.5	30.9	34.4
89	31.0	29.5	32.6	32.9	31.1	34.7
90	31.3	29.7	33.0	33.2	31.4	35.2
91	31.6	30.0	33.3	33.6	31.7	35.6
92	32.0	30.3	33.7	34.0	32.0	36.2
93	32.3	30.6	34.2	34.5	32.4	36.7
94	32.8	30.9	34.8	35.0	32.8	37.4
95	33.3	31.4	35.5	35.7	33.3	38.2
96	33.9	31.8	36.1	36.4	33.9	39.2

Burgess S, *et al. BMJ Open* 2024; 14:e081399. doi: 10.1136/bmjopen-2023-081399

97	34.7	32.4	37.1	37.4	34.6	40.5
98	35.8	33.3	38.7	38.7	35.5	42.2
99	37.3	34.1	40.5	40.5	36.7	44.8
100	40.3	35.6	43.2	44.3	38.8	47.9

We report the mean, lower decile (10th percentile of the distribution), and upper decile (90th percentile of the distribution) of the BMI distribution for participants in each stratum created by the doubly-ranked method. In the first and 100th strata, we instead report the 20th and 80th percentiles of the distribution.

Supplementary Figure 1: Associations between the genetic score and BMI in strata defined by the doubly-ranked method plotted against mean BMI in each stratum for: A) HUNT, B) UK Biobank. Error bars represent 95% confidence intervals.

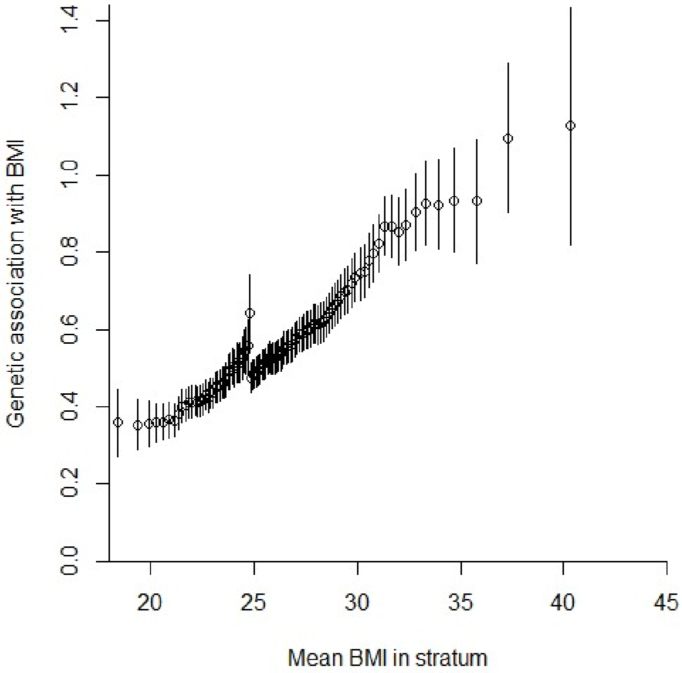

A.

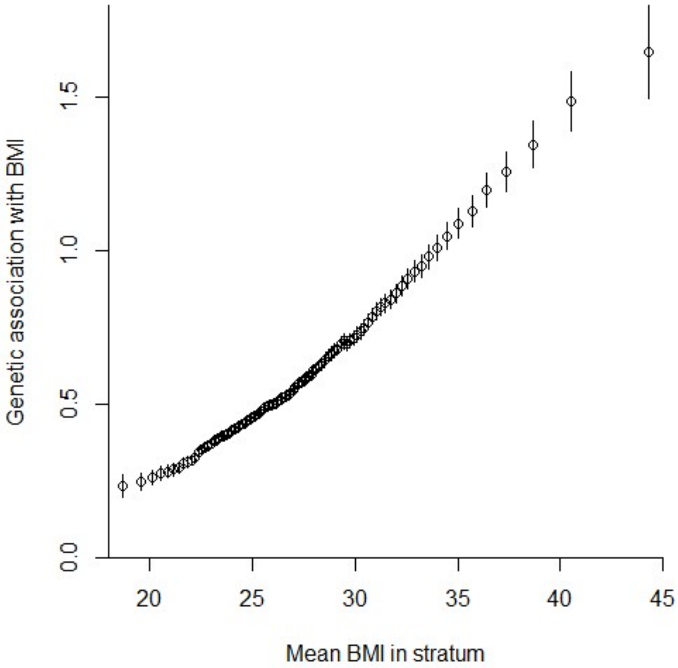

B.

Supplementary Figure 2: Genetic associations with potential competing risk factors in strata of the HUNT study population

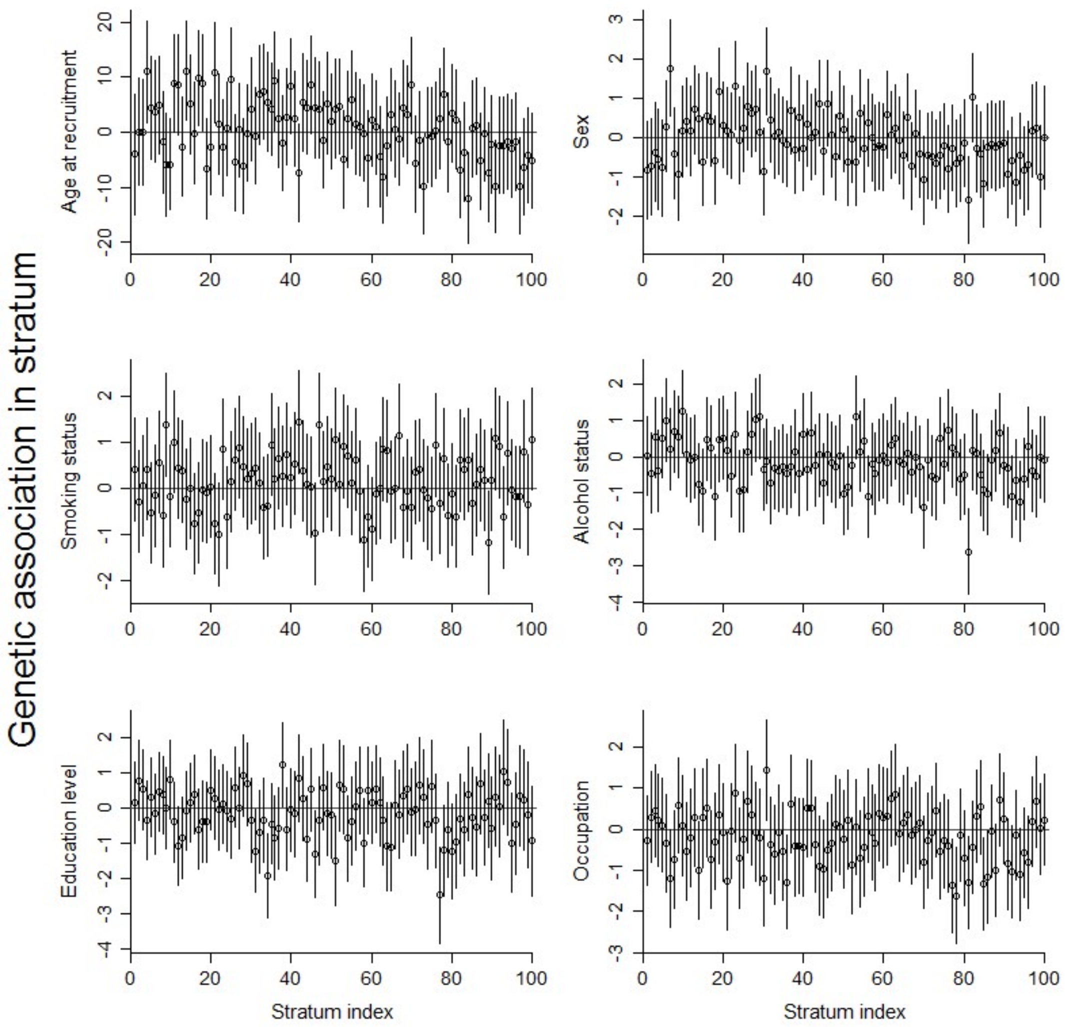

Points represent association estimates from linear (age at recruitment, years) or logistic regression (all other traits). Error bars represent 95% confidence intervals. Stratum 1 has lowest average BMI levels, stratum 100 has highest average BMI levels.

Supplementary Figure 3: Localized average causal effect estimates (stratum-specific estimates) for all-cause mortality plotted against mean BMI in each stratum. Estimates represent hazard ratios per 1 kg/m^2 increase in genetically-predicted BMI. Error bars represent 95% confidence intervals.

A) HUNT, doubly-ranked method:

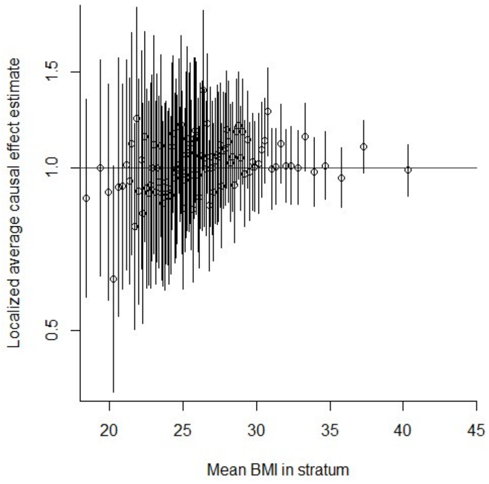

B) UK Biobank, doubly-ranked method:

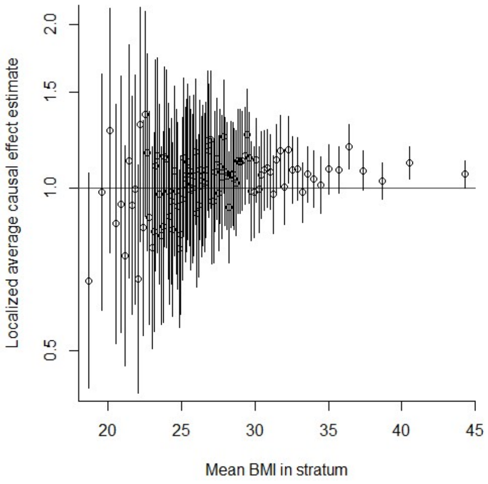

C) HUNT, residual method:

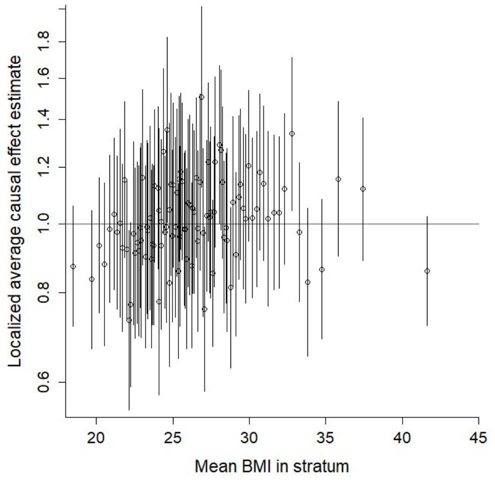

D) UK Biobank, residual method:

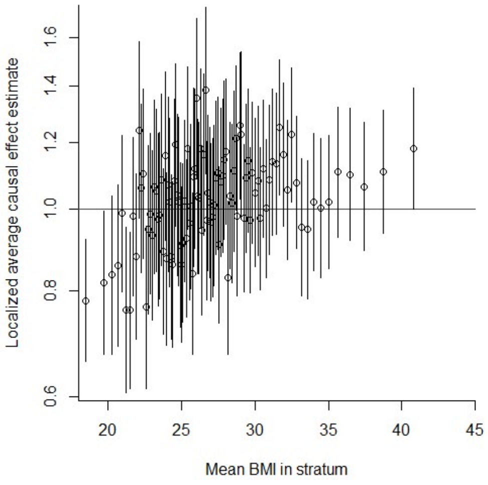

This presentation does not smooth over stratum-specific estimates, hence providing a representation of the causal effect of BMI which is not dependent on the choice of fractional polynomial smoothing. However, stratum-specific estimates are imprecise and variable, as they each only use 1% of the overall sample size. These plots differ from the presentation in the main text Figures: here, we plot the stratum-specific Mendelian randomization estimates, which (when log-transformed) reflect the slope of the curves in the main text figures.

Supplementary Figure 4: Non-linear Mendelian randomization – dose-response curve between body mass index and all-cause mortality using the doubly-ranked method with inverse-normal rank transformed BMI values in UK Biobank. Grey lines represent 95% confidence intervals. The reference value for BMI was taken as the median of the BMI distribution at 26.7 kg/m^2.

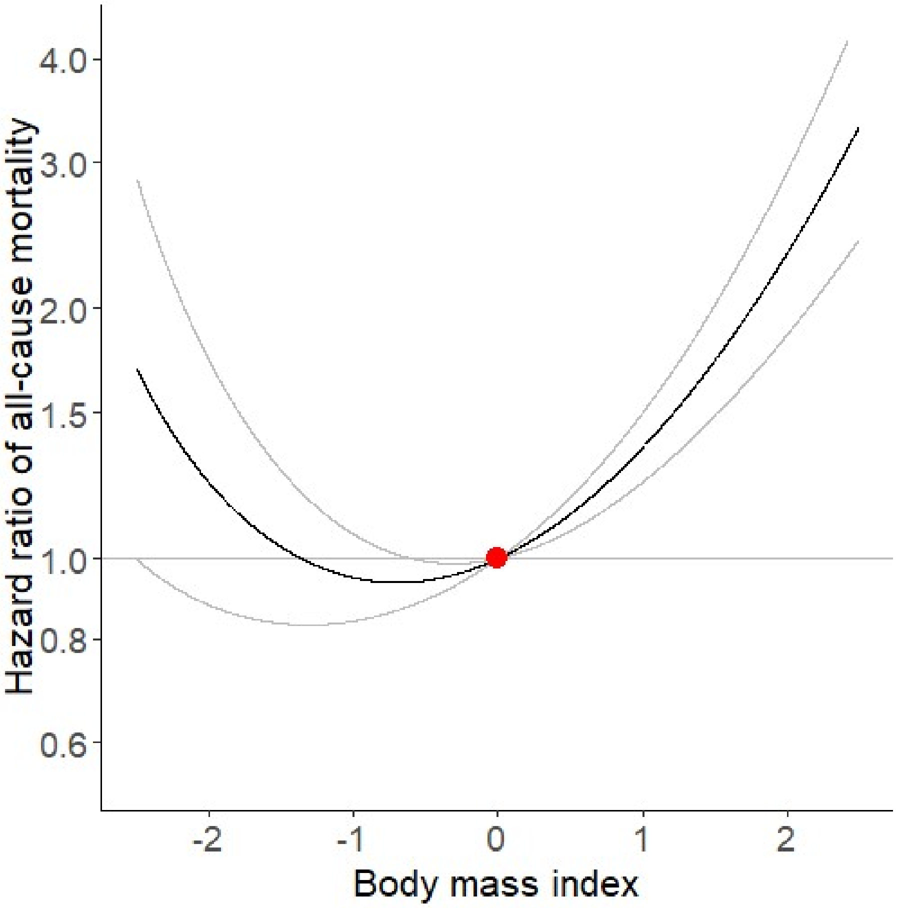

The labels on the horizontal axis represent the number of standard deviations below or above the median of the BMI distribution: for example, -1 represents one standard deviation below the median, and +2 represents two standard deviations above the median.

The reference values are:
-2 = 19.9 kg/m^2
-1 = 23.0 kg/m^2
0 = 26.7 kg/m^2
+1 = 31.6 kg/m^2
+2 = 39.2 kg/m^2

Burgess S, *et al. BMJ Open* 2024; 14:e081399. doi: 10.1136/bmjopen-2023-081399

Supplementary Figure 5: Non-linear Mendelian randomization – dose-response curve between body mass index and all-cause mortality estimated using the doubly-ranked method: A) HUNT, B) UK Biobank dividing into 50 strata; C) HUNT, D) UK Biobank dividing into 200 strata. Gradient at each point of the curve is the localized average causal effect. Grey lines represent 95% confidence intervals. The reference value for BMI was taken as 25 kg/m^2.

A) HUNT, 50 strata:

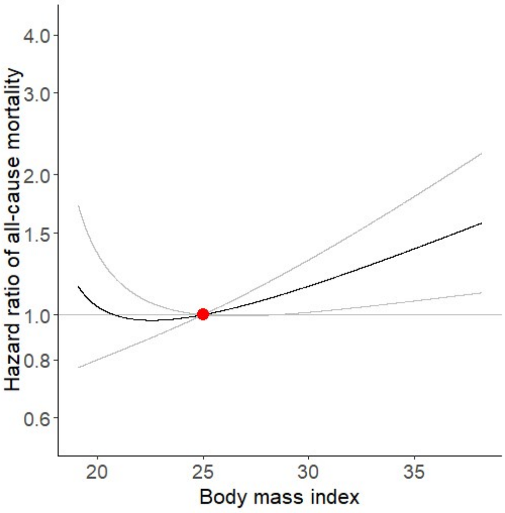

p-value for linearity: 0.50
p-value for trend: 0.49

B) UK Biobank, 50 strata:

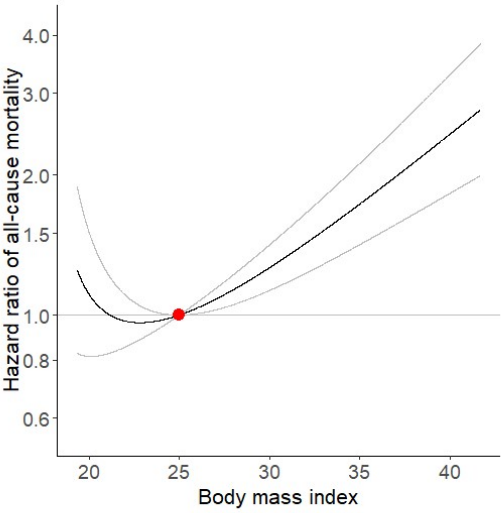

p-value for linearity: 0.10
p-value for trend: 0.09

C) HUNT, 200 strata:

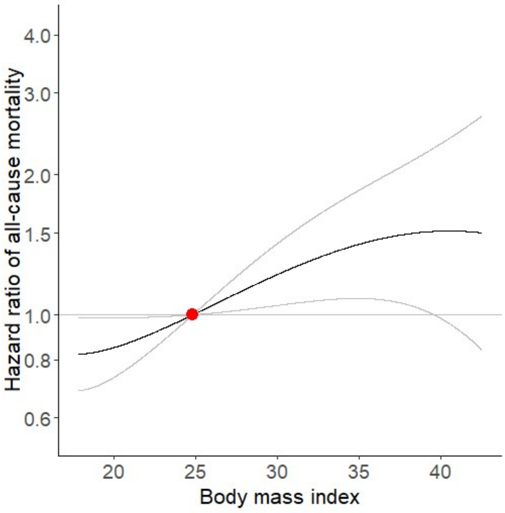

p-value for linearity: 0.99
p-value for trend: 0.88

D) UK Biobank, 200 strata:

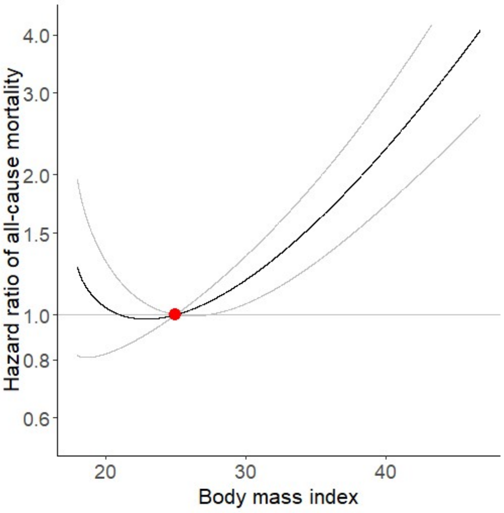

p-value for linearity: 0.28
p-value for trend: 0.16

Burgess S, *et al. BMJ Open* 2024; 14:e081399. doi: 10.1136/bmjopen-2023-081399

Supplementary Figure 6: Non-linear Mendelian randomization – dose-response curve between body mass index and all-cause mortality estimated using the doubly-ranked method and the extended genetic score in UK Biobank. Gradient at each point of the curve is the localized average causal effect. Grey lines represent 95% confidence intervals. The reference value for BMI was taken as 25 kg/m^2.

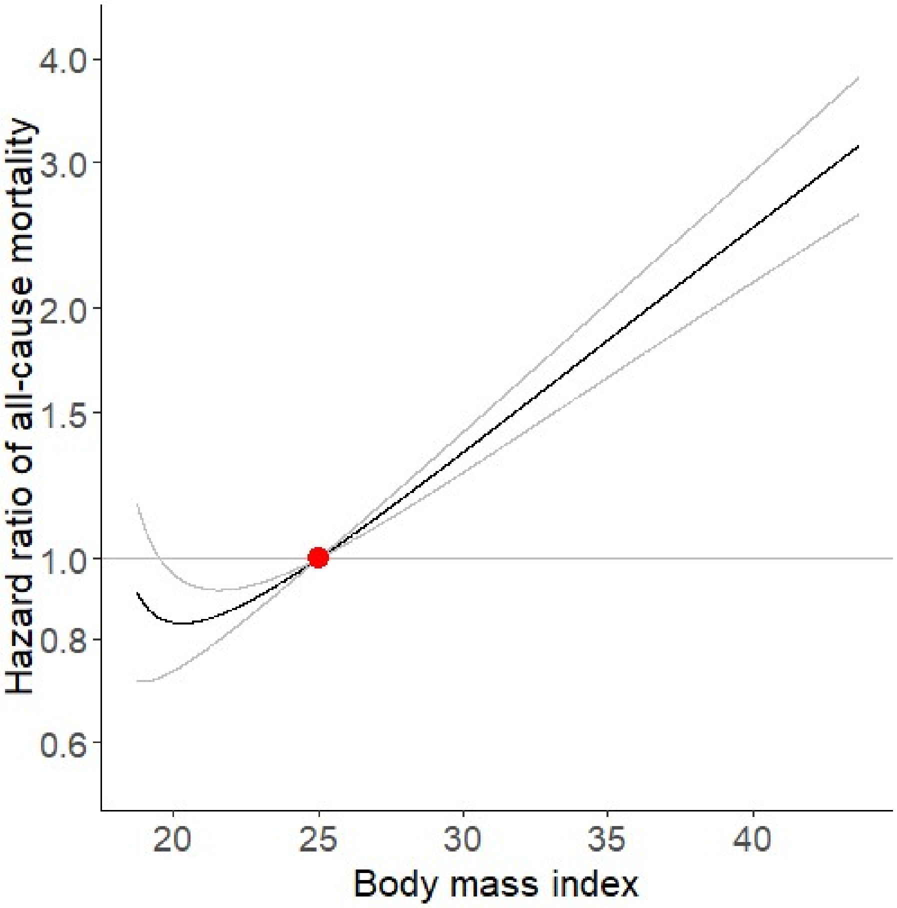

p-value for linearity: 0.99
p-value for trend: 0.32

Burgess S, *et al. BMJ Open* 2024; 14:e081399. doi: 10.1136/bmjopen-2023-081399

Supplementary Figure 7: Localized average causal effect estimates (stratum-specific estimates) for all-cause mortality plotted against mean BMI in each stratum estimated in men and women separately. Estimates represent hazard ratios per 1 kg/m^2 increase in genetically-predicted BMI. Error bars represent 95% confidence intervals.

A) HUNT, men only:

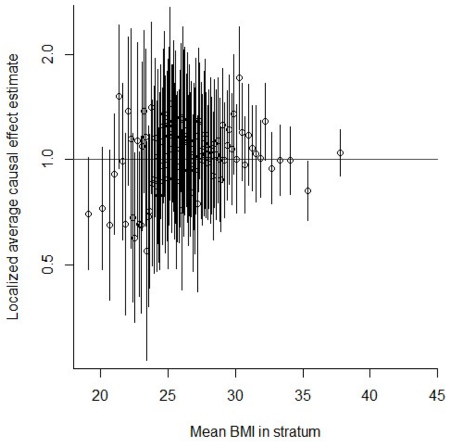

B) HUNT, women only:

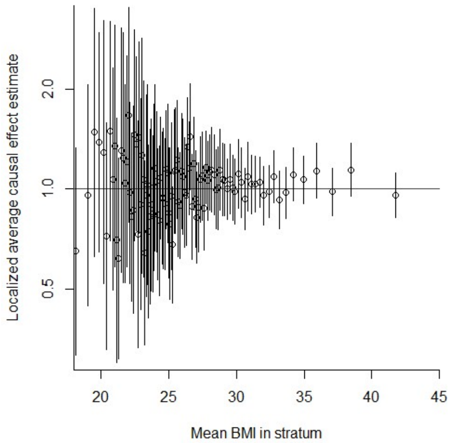

C) UK Biobank, men only:

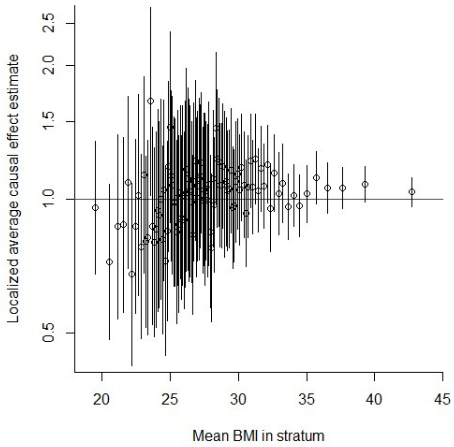

D) UK Biobank, women only:

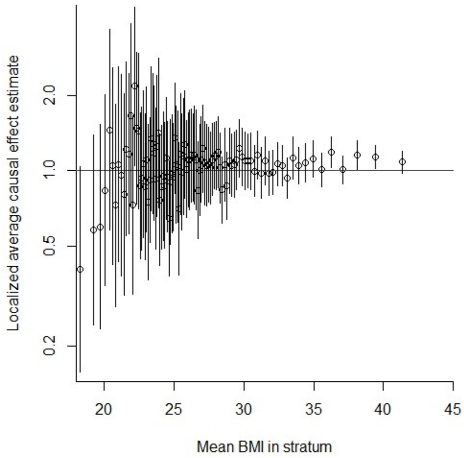

Burgess S, *et al. BMJ Open* 2024; 14:e081399. doi: 10.1136/bmjopen-2023-081399

Supplementary Figure 8: Localized average causal effect estimates (stratum-specific estimates) for cause-specific mortality plotted against mean BMI in each stratum. Estimates represent hazard ratios per 1 kg/m^2 increase in genetically-predicted BMI. Error bars represent 95% confidence intervals.

A: Cardiovascular mortality

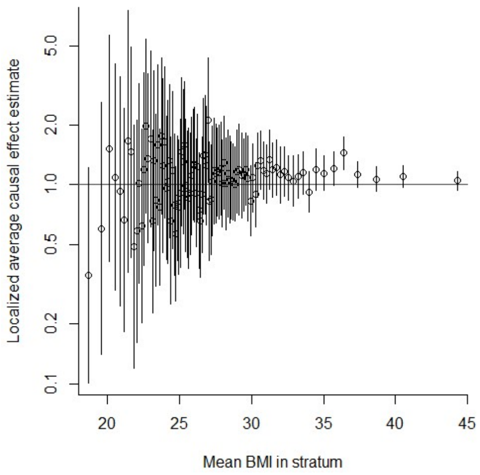

B: Cancer mortality

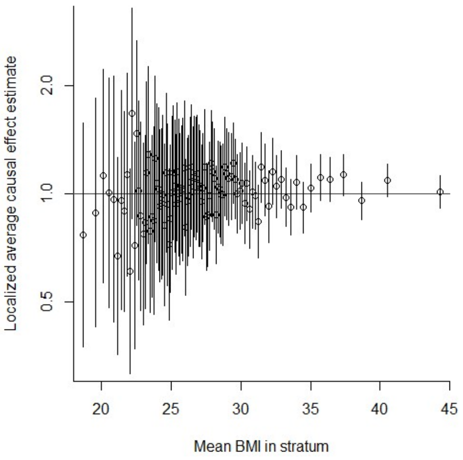

C: Other (non-cardiovascular, non-cancer) mortality

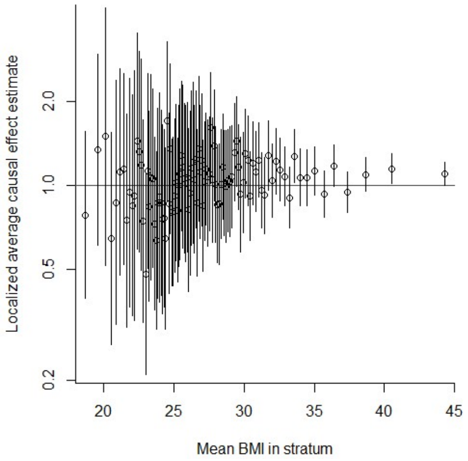

16
