# Supplementary material for: Body mass index and all-cause mortality in HUNT and UK biobank studies: revised non-linear Mendelian randomisation analyses
Source: BMJ Open. 2024 May 15;14(5):e081399. doi: 10.1136/bmjopen-2023-081399 (PMC11097829; doi:10.1136/bmjopen-2023-081399)
Supplement: Supplementary data [file bmjopen-2023-081399supp002.pdf]

STROBE-MR checklist of recommended items to address in reports of Mendelian randomization studies[1 2]

Item No.	Section	Checklist item	Page No.	Relevant text from manuscript
1	**TITLE and ABSTRACT**	Indicate Mendelian randomization (MR) as the study's design in the title and/or the abstract if that is a main purpose of the study	1-2	Title: "Body mass index and all-cause mortality in HUNT and UK Biobank studies: revised non-linear Mendelian randomization analyses"<br>Abstract: "**Objectives**: To estimate the shape of the causal relationship between body mass index (BMI) and mortality risk in a Mendelian randomization framework."
	**INTRODUCTION**			
2	**Background**	Explain the scientific background and rationale for the reported study. What is the exposure? Is a potential causal relationship between exposure and outcome plausible? Justify why MR is a helpful method to address the study question	2,4	Abstract: "To estimate the shape of the causal relationship between body mass index (BMI) and mortality risk in a Mendelian randomization framework."<br>Introduction: "The epidemiological relationship between BMI and mortality is complex, with many observational studies conducted in Western countries showing a J-shaped relationship between BMI and mortality risk."<br>"However, such findings may not reflect the causal relationship between BMI and mortality, as observation association are influenced by confounding and reverse causation."
3	**Objectives**	State specific objectives clearly, including pre-specified causal hypotheses (if any). State that MR is a method that, under specific assumptions, intends to estimate causal effects	2,4	Abstract: "To estimate the shape of the causal relationship between body mass index (BMI) and mortality risk in a Mendelian randomization framework."<br>Introduction: "We first investigate whether the constant genetic effect assumption holds for this example, and explore the validity of the instrumental variable assumptions in strata of the population. We then present non-linear Mendelian randomization findings from the doubly-ranked stratification method."<br>"Hence, associations between genetic predictors of BMI and mortality provide insights into the causal effect of BMI on mortality."

For peer review only - http://bmjopen.bmj.com/site/about/guidelines.xhtml

	**METHODS**			
4	**Study design and data sources**	Present key elements of the study design early in the article. Consider including a table listing sources of data for all phases of the study. For each data source contributing to the analysis, describe the following:		
	a)	Setting: Describe the study design and the underlying population, if possible. Describe the setting, locations, and relevant dates, including periods of recruitment, exposure, follow-up, and data collection, when available.	6	See sections "The HUNT study" and "The UK Biobank study" (some of this detail is in reference 19, as indicated: "We present abbreviated descriptions of the datasets included in the analysis; detailed descriptions are in the original paper [19]").
	b)	Participants: Give the eligibility criteria, and the sources and methods of selection of participants. Report the sample size, and whether any power or sample size calculations were carried out prior to the main analysis	6	See sections "The HUNT study" and "The UK Biobank study" (detail is in reference 19: "We present abbreviated descriptions of the datasets included in the analysis; detailed descriptions are in the original paper [19]").
	c)	Describe measurement, quality control and selection of genetic variants	6	See section "SNPs and genetic score used as instrumental variables"
	d)	For each exposure, outcome, and other relevant variables, describe methods of assessment and diagnostic criteria for diseases	NA	See reference 19 for detail.
	e)	Provide details of ethics committee approval and participant informed consent, if relevant	7	See section "Ethical Approval" and "Patient Involvement"
5	**Assumptions**	Explicitly state the three core IV assumptions for the main analysis (relevance, independence and exclusion restriction) as well assumptions for any additional or sensitivity analysis	4,6	Introduction: "According to Mendel's laws of inheritance, genetic variants should be uncorrelated with traits that they do not affect, and hence should be independent of potential confounders"<br>Methods: "We assessed the instrumental variable assumptions by estimating associations between the genetic score and various traits that are competing risk factors in UK Biobank: smoking status (ever versus never), alcohol status (current versus other), education level (post-secondary or higher versus other), occupation (currently employed versus other), age at recruitment, and sex."
6	**Statistical methods: main analysis**	Describe statistical methods and statistics used	6-7	See Section "Statistical analyses".

For peer review only - http://bmjopen.bmj.com/site/about/guidelines.xhtml

Burgess S, *et al. BMJ Open* 2024; 14:e081399. doi: 10.1136/bmjopen-2023-081399

		a)	Describe how quantitative variables were handled in the analyses (i.e., scale, units, model)	7	See Section "Statistical analyses".
		b)	Describe how genetic variants were handled in the analyses and, if applicable, how their weights were selected	6	See Section "SNPs and genetic score used as instrumental variables"
		c)	Describe the MR estimator (e.g. two-stage least squares, Wald ratio) and related statistics. Detail the included covariates and, in case of two-sample MR, whether the same covariate set was used for adjustment in the two samples	7	See Section "Statistical analyses":<br>"We then calculated the linear Mendelian randomization estimate, referred to as a localized average causal effect (LACE), in each stratum of the population as a ratio of coefficients: the association of the genetic score with the outcome divided by the associations of the genetic score with the exposure. associations with the exposure (BMI) were obtained from linear regression; associations with the outcome (mortality) were obtained from Cox proportional hazards regression, using age as the timescale. All associations were adjusted for age, age-squared, sex, and centre (for UK Biobank)."
		d)	Explain how missing data were addressed	6	"We excluded participants without data on BMI or genetic variants for BMI, leaving 56,150 individuals for analysis."
		e)	If applicable, indicate how multiple testing was addressed	N/A	We did not account for multiple testing, as the presentation of p-values is not a major aspect of the manuscript.
7	**Assessment of assumptions**		Describe any methods or prior knowledge used to assess the assumptions or justify their validity	6	See Section "SNPs and genetic score used as instrumental variables":<br>"We assessed the instrumental variable assumptions by estimating associations between the genetic score and various traits that are competing risk factors in UK Biobank: smoking status (ever versus never), alcohol status (current versus other), education level (post-secondary versus other), occupations (currently employed versus other), age at recruitment, and sex.
8	**Sensitivity analyses and additional analyses**		Describe any sensitivity analyses or additional analyses performed (e.g. comparison of effect estimates from different approaches, independent replication, bias analytic techniques, validation of instruments, simulations)	7	Sensitivity Analyses: "In brief, first we divided the sample into 100 strata using the doubly-ranked method. For comparison, we also present results from stratification using the residual method for the primary outcome of all-cause mortality."<br>Additional analyses: Testing for linearity:

					"We report two tests for non-linearity: a linearity test, which assesses whether a non-linear model fits the LACE estimates better than a linear model, and a trend test, which tests for a linear trend amongst the LACE estimates. "
9	**Software and pre-registration**				
		a)	Name statistical software and package(s), including version and settings used	7	"All statistical analyses were performed in R (version 4.3.1), and non-linear Mendelian randomisation analyses were performed using the SUMnlmr package [25]."
		b)	State whether the study protocol and details were pre-registered (as well as when and where)	N/A	The study protocol was not pre-registered.
	**RESULTS**				
10	**Descriptive data**				
		a)	Report the numbers of individuals at each stage of included studies and reasons for exclusion. Consider use of a flow diagram		All numbers provided in Table 1.
		b)	Report summary statistics for phenotypic exposure(s), outcome(s), and other relevant variables (e.g. means, SDs, proportions)		All numbers provided in Table 1.
		c)	If the data sources include meta-analyses of previous studies, provide the assessments of heterogeneity across these studies		N/A – we present results from the HUNT and UK Biobank studies separately, and do not attempt to meta-analyse or otherwise combine results.
		d)	For two-sample MR:<br>i. Provide justification of the similarity of the genetic variant-exposure associations between the exposure and outcome samples<br>ii. Provide information on the number of individuals who overlap between the exposure and outcome studies		N/A – not two-sample MR.
11	**Main results**				
		a)	Report the associations between genetic variant and exposure, and between genetic variant and outcome, preferably on an interpretable scale		Genetic associations with exposure are shown in Supplementary Figure 1.<br>Scaled genetic association with outcome are shown in Supplementary Figures 3, 5, and 6.
		b)	Report MR estimates of the relationship between exposure and outcome, and the measures of uncertainty from the MR analysis, on an interpretable scale, such as odds ratio or relative risk per SD difference		MR estimates are shown directly in Supplementary Figures 3, 5, and 6. Smoothed versions of these associations are shown in Figures 2, 3, and 4.

For peer review only - http://bmjopen.bmj.com/site/about/guidelines.xhtml

Supplemental material BMJ Publishing Group Limited (BMJ) disclaims all liability and responsibility arising from any reliance placed on this supplemental material which has been supplied by the author(s) BMJ Open

		c)	If relevant, consider translating estimates of relative risk into absolute risk for a meaningful time period		N/A
		d)	Consider plots to visualize results (e.g. forest plot, scatterplot of associations between genetic variants and outcome versus between genetic variants and exposure)		N/A
12	**Assessment of assumptions**				
		a)	Report the assessment of the validity of the assumptions	8	See section "Assessment of instrument validity"
		b)	Report any additional statistics (e.g., assessments of heterogeneity across genetic variants, such as I^2, Q statistic or E-value)	6	F statistics are provided in the Methods section under "SNPs and genetic score used as instrumental variables.<br>Nonlinear tests are reported in Figures 2, 3, and 4.
13	**Sensitivity analyses and additional analyses**				
		a)	Report any sensitivity analyses to assess the robustness of the main results to violations of the assumptions		See Figure 2: residual versus ranked stratification.
		b)	Report results from other sensitivity analyses or additional analyses		Figure 2: residual versus ranked stratification<br>Figure 3: results stratified by sex<br>Figure 4: exploring different cause-specific mortality subtypes.
		c)	Report any assessment of direction of causal relationship (e.g., bidirectional MR)		N/A, directionality not considered.
		d)	When relevant, report and compare with estimates from non-MR analyses	11	In Discussion, paragraph beginning "Previous investigations into the non-linear shape of the causal relationship between BMI and mortality have used a variety of approaches." and in particular "Carslake *et al* performed an instrumental variable analysis to investigate the impact of BMI on mortality in the HUNT study, but using offspring BMI as an instrument for the BMI".
		e)	Consider additional plots to visualize results (e.g., leave-one-out analyses)		N/A
	**DISCUSSION**				
14	**Key results**		Summarize key results with reference to study objectives	10	Results are summarized in the Discussion section:

For peer review only - http://bmjopen.bmj.com/site/about/guidelines.xhtml

Burgess S, *et al. BMJ Open* 2024; 14:e081399. doi: 10.1136/bmjopen-2023-081399

					"While the curves generally display a J-shaped relationship, evidence for a harmful average effect of decreased BMI on mortality at low BMI levels was limited. Evidence supporting non-linearity from the doubly-ranked method was unconvincing for all mortality outcomes." Also see Abstract / Results.
15	**Limitations**		Discuss limitations of the study, taking into account the validity of the IV assumptions, other sources of potential bias, and imprecision. Discuss both direction and magnitude of any potential bias and any efforts to address them	11-12	Limitations are clearly discussed in the Discussion section, paragraph beginning "However, there are important limitations…" Several potential limitations are discussed, including validity of instruments, pleiotropy, bias due to selection bias, representativeness, and reverse causation.
16	**Interpretation**				
		a)	Meaning: Give a cautious overall interpretation of results in the context of their limitations and in comparison with other studies	10	Interpretation of results is provided in the Discussion section: "In conclusion, non-linear Mendelian randomization analyses using the doubly-ranked stratification method provide strong evidence for harmful effects of increased BMI on mortality above 25 kg/m2. Evidence for a harmful effect of low BMI was only present in some analyses, and where present, only below 20 kg/m2.". Also see Abstract/Conclusion.
		b)	Mechanism: Discuss underlying biological mechanisms that could drive a potential causal relationship between the investigated exposure and the outcome, and whether the gene-environment equivalence assumption is reasonable. Use causal language carefully, clarifying that IV estimates may provide causal effects only under certain assumptions		N/A (mechanisms linking BMI and mortality have been discussed elsewhere at length, and evidence for specific mechanisms is not provided here).
		c)	Clinical relevance: Discuss whether the results have clinical or public policy relevance, and to what extent they inform effect sizes of possible interventions	10	Discussion: "Our findings challenge the "obesity paradox" consensus from observational epidemiology that increases in BMI might reduce risk of mortality for a substantial proportion of the population."
17	**Generalizability**		Discuss the generalizability of the study results (a) to other populations, (b) across other exposure periods/timings, and (c) across other levels of exposure	11	Discussion / Limitations: "Our findings may therefore not be applicable to other populations. "

For peer review only - http://bmjopen.bmj.com/site/about/guidelines.xhtml

	**OTHER INFORMATION**			
18	**Funding**	Describe sources of funding and the role of funders in the present study and, if applicable, sources of funding for the databases and original study or studies on which the present study is based	1	See “Funding statement”
19	**Data and data sharing**	Provide the data used to perform all analyses or report where and how the data can be accessed, and reference these sources in the article. Provide the statistical code needed to reproduce the results in the article, or report whether the code is publicly accessible and if so, where	31	See “Data sharing statement”
20	**Conflicts of Interest**	All authors should declare all potential conflicts of interest	1	Provided under “Competing interest statement”

This checklist is copyrighted by the Equator Network under the Creative Commons Attribution 3.0 Unported (CC BY 3.0) license.

1. Skrivankova VW, Richmond RC, Woolf BAR, Yarmolinsky J, Davies NM, Swanson SA, et al. Strengthening the Reporting of Observational Studies in Epidemiology using Mendelian Randomization (STROBE-MR) Statement. JAMA. 2021;under review.

2. Skrivankova VW, Richmond RC, Woolf BAR, Davies NM, Swanson SA, VanderWeele TJ, et al. Strengthening the Reporting of Observational Studies in Epidemiology using Mendelian Randomisation (STROBE-MR): Explanation and Elaboration. BMJ. 2021;375:n2233.

For peer review only - http://bmjopen.bmj.com/site/about/guidelines.xhtml
