# Supplementary material for: Body mass index and all-cause mortality in HUNT and UK biobank studies: revised non-linear Mendelian randomisation analyses
Source: BMJ Open. 2024 May 15;14(5):e081399. doi: 10.1136/bmjopen-2023-081399 (PMC11097829; doi:10.1136/bmjopen-2023-081399)
Supplement: Author's manuscript [file bmjopen-2023-081399.draft_revisions.pdf]

BMJ Open

BMJ Open is committed to open peer review. As part of this commitment we make the peer review history of every article we publish publicly available.

When an article is published we post the peer reviewers' comments and the authors' responses online. We also post the versions of the paper that were used during peer review. These are the versions that the peer review comments apply to.

The versions of the paper that follow are the versions that were submitted during the peer review process. They are not the versions of record or the final published versions. They should not be cited or distributed as the published version of this manuscript.

BMJ Open is an open access journal and the full, final, typeset and author-corrected version of record of the manuscript is available on our site with no access controls, subscription charges or pay-per-view fees (http://bmjopen.bmj.com).

If you have any questions on BMJ Open's open peer review process please email info.bmjopen@bmj.com

BMJ Open

Body mass index and all-cause mortality in HUNT and UK Biobank studies: revised non-linear Mendelian randomization analyses

Journal:	*BMJ Open*
Manuscript ID	bmjopen-2023-081399
Article Type:	Original research
Date Submitted by the Author:	27-Oct-2023

Complete List of Authors:	Burgess, Stephen; University of Cambridge, MRC Biostatistics Unit; University of Cambridge, Cardiovascular Epidemiology Unit, Department of Public Health and Primary Care<br>Sun, Yi-Qian; Norges teknisk-naturvitenskapelige universitet, Department of Clinical and Molecular Medicine (IKOM)<br>Zhou, Ang; University of Cambridge, MRC Biostatistics Unit; University of South Australia, Australian Centre for Precision Health<br>Buck, Christopher; University of Cambridge, MRC Biostatistics Unit<br>Mason, Amy M; University of Cambridge, Department of Public Health and Primary Care<br>Mai, Xiao-Mei; Norwegian University of Science and Technology, Department of Public Health and Nursing

Keywords:	Obesity, Observational Study, GENETICS, EPIDEMIOLOGY

SCHOLARONE™
Manuscripts

I, the Submitting Author has the right to grant and does grant on behalf of all authors of the Work (as defined in the below author licence), an exclusive licence and/or a non-exclusive licence for contributions from authors who are: i) UK Crown employees; ii) where BMJ has agreed a CC-BY licence shall apply, and/or iii) in accordance with the terms applicable for US Federal Government officers or employees acting as part of their official duties; on a worldwide, perpetual, irrevocable, royalty-free basis to BMJ Publishing Group Ltd ("BMJ") its licensees and where the relevant Journal is co-owned by BMJ to the co-owners of the Journal, to publish the Work in this journal and any other BMJ products and to exploit all rights, as set out in our licence.

The Submitting Author accepts and understands that any supply made under these terms is made by BMJ to the Submitting Author unless you are acting as an employee on behalf of your employer or a postgraduate student of an affiliated institution which is paying any applicable article publishing charge ("APC") for Open Access articles. Where the Submitting Author wishes to make the Work available on an Open Access basis (and intends to pay the relevant APC), the terms of reuse of such Open Access shall be governed by a Creative Commons licence – details of these licences and which Creative Commons licence will apply to this Work are set out in our licence referred to above.

Other than as permitted in any relevant BMJ Author's Self Archiving Policies, I confirm this Work has not been accepted for publication elsewhere, is not being considered for publication elsewhere and does not duplicate material already published. I confirm all authors consent to publication of this Work and authorise the granting of this licence.

For peer review only

Body mass index and all-cause mortality in HUNT and UK Biobank studies: revised non-linear Mendelian randomization analyses

Stephen Burgess[1,2,3], Yi-Qian Sun[4,5,6], Ang Zhou[1,7], Christopher Buck[1], Amy M Mason[2,3], Xiao-Mei Mai[8]

1. Medical Research Council Biostatistics Unit, University of Cambridge, Cambridge, UK.
2. Cardiovascular Epidemiology Unit, Department of Public Health and Primary Care, University of Cambridge, Cambridge, UK.
3. Victor Phillip Dahdaleh Heart and Lung Research Institute, University of Cambridge, Cambridge, UK.
4. Department of Clinical and Molecular Medicine, Faculty of Medicine and Health Science, Norwegian University of Science and Technology, Trondheim, Norway.
5. Department of Pathology, Clinic of Laboratory Medicine, St. Olavs Hospital, Trondheim University Hospital, Trondheim, Norway.
6. Center for Oral Health Services and Research Mid-Norway (TkMidt), Trondheim, Norway.
7. Australian Centre for Precision Health, Unit of Clinical and Health Sciences, University of South Australia, Adelaide, Australia.
8. Department of Public Health and Nursing, Faculty of Medicine and Health Science, Norwegian University of Science and Technology, Trondheim, Norway.

Corresponding author: Stephen Burgess (sb452@medschl.cam.ac.uk)

Funding statement: This work was supported by the Wellcome Trust (grant number 225790/Z/22/Z), the United Kingdom Research and Innovation Medical Research Council (grant number MC_UU_00002/7), the British Heart Foundation (RG/18/13/33946) and the National Institute for Health and Care Research (NIHR) Cambridge Biomedical Research Centre (BRC-1215-20014; NIHR203312). YQS was supported by a researcher grant from The Liaison Committee for Education, Research and Innovation in Central Norway (project ID 2018/42794). AMM is funded by the National Institute for Health and Care Research (NIHR) Blood and Transplant Research Unit (BTRU) in Donor Health and Behaviour (NIHR203337). The views expressed are those of the authors and not necessarily those of the NIHR, NHSBT or the Department of Health and Social Care.

Competing interest statement: None declared.

Word count: 299 (abstract), 4357 (main text).

Key words: Obesity paradox, reverse causation, genetic epidemiology, causal inference, doubly-ranked stratification.

Acknowledgments

The Trøndelag Health Study (HUNT) is a collaboration between the HUNT Research Centre (Faculty of Medicine and Health Sciences, NTNU, Norwegian University of Science and Technology), Trøndelag County Council, Central Norway Regional Health Authority and the Norwegian Institute of Public Health.

This research has been conducted using the UK Biobank Resource under Application Number 7439.

For peer review only - http://bmjopen.bmj.com/site/about/guidelines.xhtml

ABSTRACT

Objectives: To estimate the shape of the causal relationship between body mass index (BMI) and mortality risk in a Mendelian randomization framework.

Design: Mendelian randomization analyses of two prospective population-based cohorts.

Setting: Individuals of European ancestries living in Norway or the United Kingdom.

Participants: 56,150 participants from the Trøndelag Health Study (HUNT) in Norway and 366,385 participants from UK Biobank recruited by postal invitation.

Outcomes: All-cause mortality and cause-specific mortality (cardiovascular, cancer, non-cardiovascular non-cancer).

Results: A previously published non-linear Mendelian randomization analysis of these data using the residual stratification method suggested a J-shaped association between genetically-predicted BMI and mortality outcomes with the lowest mortality risk at a BMI of around 25 kg/m^2. However, the "constant genetic effect" assumption required by this method is violated. The re-analysis of these data using the more reliable doubly-ranked stratification method still indicated a J-shaped relationship, but with less precision in estimates at the lower end of the BMI distribution. Evidence for a harmful effect of reducing BMI at low BMI levels was only present in some analyses, and where present, only below 20 kg/m^2. A harmful effect of increasing BMI for all-cause mortality was evident above 25 kg/m^2, for cardiovascular mortality above 24 kg/m^2, for non-cardiovascular non-cancer mortality above 26 kg/m^2, and for cancer mortality above 30 kg/m^2. In UK Biobank, the association between genetically-predicted BMI and mortality at high BMI levels was stronger in women than in men.

Conclusion: This research challenges findings from previous conventional observational epidemiology and Mendelian randomization investigations that the lowest level of mortality risk is at a BMI level of around 25 kg/m^2. Our results provide evidence that reductions in BMI will only increase mortality risk for a small proportion of the population, and increases in BMI will increase mortality risk for those with BMI above 25 kg/m^2.

For peer review only

For peer review only - http://bmjopen.bmj.com/site/about/guidelines.xhtml

Strengths and limitations of the study

- Mendelian randomization design minimizes bias due to confounding and reverse causation
- Large sample sizes enable powerful analyses even in low BMI individuals
- Validity of the genetic variants as instrumental variables cannot be verified
- Bias due to selection could be non-negligible and could vary across strata
- All estimates are averaged across a stratum of the population; individual effects of raising or lowering BMI may vary between individuals

For peer review only

For peer review only - http://bmjopen.bmj.com/site/about/guidelines.xhtml

INTRODUCTION

Body mass index (BMI) is a convenient and accessible measure of obesity. The epidemiological relationship between BMI and mortality is complex, with many observational studies conducted in Western countries showing a J-shaped relationship between BMI and mortality risk in the general population, such that mortality risk is lowest for those in the upper normal weight (BMI 20.0-24.9)[1-3] or even the overweight (BMI 25.0-29.9)[4-6] category rather than the lower normal weight (BMI 18.5-19.9) or underweight category (BMI <18.5). However, such findings may not reflect the causal relationship between BMI and mortality, as observational associations are influenced by confounding and reverse causation.

Mendelian randomization is an epidemiological technique for the analysis of observational data designed to avoid bias due to confounding and reverse causation[7]. Rather than assessing associations between BMI and mortality directly, we assess associations between genetic variants that predict BMI levels and mortality risk[8]. This is analogous to the analysis of a randomized controlled trial for weight loss[9], which would assess associations between the randomized intervention and the outcome, rather than associations with measured BMI in trial participants. According to Mendel's laws of inheritance, genetic variants should be uncorrelated with traits that they do not affect, and hence should be independent of potential confounders. As genetic variants are determined at conception, genetic associations should be protected from reverse causation[10]. These properties mean that genetic variants are plausible instrumental variables; an instrumental variable is a variable that behaves as if it has been randomized in the population[11]. Hence, associations between genetic predictors of BMI and mortality provide insights into the causal effect of BMI on mortality.

Non-linear Mendelian randomization is an extension of standard Mendelian randomization to investigate the shape of the causal relationship between an exposure and an outcome[12]. A typical randomized trial estimates an average causal effect, representing the average impact of a population-wide shift in the distribution of an exposure; similarly, a standard Mendelian randomization analysis estimates a population-averaged causal effect[13]. A widely used method for non-linear Mendelian randomization stratifies the population based on levels of the exposure, and estimates 'localized average causal effects', representing the average impact of a population-wide shift in the distribution of the exposure for that stratum of the population[13].

However, stratifying the population such that the instrumental variable assumptions still hold in the strata of the population is tricky. Stratifying on the exposure directly would break randomization, as exposure levels are influenced by the instrumental variables. Hence an individual in the lowest stratum of the population defined by the exposure with a genetic predisposition to high values of the exposure would be more likely to have low values of the confounders. This is an example of collider bias[14]: the exposure is a common effect of the instrument and exposure—outcome confounders, and so stratification on the exposure leads to a conditional association between the instrument and confounders. The initial proposal for non-linear Mendelian randomization was to stratify on the residual exposure, defined as the residual from regression of the exposure on the instrumental variables[13]. The residual exposure is independent of the instrumental variables by construction, and the instrumental variables will be independent of confounders within strata of the residual exposure under a "constant genetic effect" assumption[15]. However, if the effect of the genetic instrumental variables on the exposure varies between individuals, then this residual stratification method can lead to severe bias in stratum-specific estimates[16,17]. An alternative method, known as the doubly-ranked method, allows the instrumental variable effects on the exposure to vary between individuals provided that a weaker "rank-preserving assumption" holds[18].

For peer review only - http://bmjopen.bmj.com/site/about/guidelines.xhtml

The rank-preserving assumption states that the ranking of individuals according to their exposure levels would be the same at all levels of the instrumental variable. The doubly-ranked method has been shown to be more reliable than the residual method in simulation studies[17,18].

In a previous paper, we presented evidence for the effect of BMI on mortality from non-linear Mendelian randomization using the residual stratification method in the Norwegian Trøndelag Health (HUNT) and UK Biobank studies. A J-shaped association between genetically-predicted BMI and mortality was observed in each study population overall, although a monotonic increasing association (that is, an always-increasing association) was seen in never-smokers[19]. Here, we present a re-analysis of the same datasets using the doubly-ranked stratification method. We first investigate whether the constant genetic effect assumption holds for this example, and explore the validity of the instrumental variable assumptions in strata of the population. We then present non-linear Mendelian randomization findings from the doubly-ranked stratification method.

For peer review only

For peer review only - http://bmjopen.bmj.com/site/about/guidelines.xhtml

METHODS

We present abbreviated descriptions of the datasets included in the analysis; detailed descriptions are in the original paper[19].

The HUNT study

We used data from the second wave (1995-1997) of the HUNT study on 65,229 individuals living in the northern part of Trøndelag and over the age of 20 years[20]. Participants were followed up until 15 June 2023 or their date of death. The original paper only considered mortality outcomes up to April 2015. By extending the follow-up period, we increase the number of mortality events considered in the analysis from 12,015 to 18,836. We excluded participants without data on BMI or genetic variants for BMI, leaving 56,150 individuals for analysis.

The UK Biobank study

The UK Biobank cohort comprises around 500,000 participants (94% of self-reported European ancestry) aged 40 to 69 years at baseline. They were recruited between 2006-2010 in 22 assessment centres throughout the UK, and followed up until 28 February 2021 or their date of death[21]. Again, the original paper only considered mortality outcomes up to February 2016. By extending the follow-up period, we increase the number of mortality events considered in the analysis from 10,344 to 25,021. We performed detailed quality control procedures on UK Biobank participants and on genetic variants as described previously[22]. In total, 366,385 unrelated European ancestry participants were included in analyses.

SNPs and genetic score used as instrumental variables

77 single nucleotide polymorphisms (SNPs) were selected as candidate instrumental variables for BMI based on European sex-combined analyses in a genome-wide association study of the GIANT consortium[23]. Two of these variants (rs12016871 and rs2033732) were not available in the HUNT study, and a further two variants (rs13021737 and rs16951275) were excluded from the analyses due to association with smoking status in the HUNT study. An externally-weighted score was calculated for each individual by multiplying the number of BMI-increasing alleles the individual carries by the variant's association with BMI from the GIANT study (**Supplementary Table 1**), and summing across the remaining 73 variants. Overall, the genetic score explained 2.0% and 1.6% of the variance in BMI in the HUNT and UK Biobank studies respectively, corresponding to F-statistics of 1121 and 5964.

We assessed the instrumental variable assumptions within strata by estimating associations between the genetic score and various traits that are competing risk factors: smoking status (ever versus never), alcohol status (current versus other), education level (post-secondary or higher versus other), occupation (currently employed versus other), age at recruitment, and sex.

Study design

We performed non-linear Mendelian randomization analyses to estimate the shape of the association between genetically-predicted BMI and the outcome. Our primary analysis considered all-cause mortality as the outcome. We also conducted subgroup analyses considering men and women separately. In addition, we studied associations with cause-specific mortality events (cardiovascular, cancer, and non-cardiovascular non-cancer) in UK Biobank. We did not perform analyses stratified by smoking status (as presented in the original paper), as smoking status is influenced by BMI[24], and hence this stratification may introduce collider bias.

For peer review only - http://bmjopen.bmj.com/site/about/guidelines.xhtml

Statistical analyses

To estimate the non-linear relationship between genetically-predicted BMI and mortality risk, a fractional polynomial method was applied[12]. In brief, first we divided the sample into 100 strata using the doubly-ranked method[18]. For comparison, we also present results from stratification using the residual method for the primary outcome of all-cause mortality. We then calculated the linear Mendelian randomization estimate, referred to as a localized average causal effect (LACE), in each stratum of the population as a ratio of coefficients: the association of the genetic score with the outcome divided by the association of the genetic score with the exposure. Associations with the exposure (BMI) were obtained from linear regression; associations with the outcome (mortality) were obtained from Cox proportional hazards regression, using age as the timescale. All associations were adjusted for age, age-squared, sex, and centre (for UK Biobank).

We performed meta-regression of the LACE estimates against the mean of the exposure in each stratum in a flexible semiparametric framework using the derivative of fractional polynomial models of degrees 1 and 2. Under the Mendelian randomization assumptions, the slope of the curve represents the average causal effect of the exposure on the outcome for the stratum with that exposure level. A positive causal effect is evident at a particular exposure level when the lower and upper confidence limits for the curve both have a positive slope; and similarly an inverse causal effect is evident if both confidence limits have a negative slope. The reference point in analyses is set to 25 kg/m^2; however, this reference point is arbitrarily chosen, and the key indicator of an increasing or decreasing effect is not whether confidence intervals include or exclude the reference point, but rather whether the slope of the curve is positive or negative at a given exposure value.

We report two tests for non-linearity: a linearity test, which assesses whether a non-linear model fits the LACE estimates better than a linear model, and a trend test, which tests for a linear trend amongst the LACE estimates. Rejection of the linearity test indicates that a linear model is a suboptimal fit to the data; failure to reject means any improvement in fit for the best fitting non-linear model over the linear model is no more than would be expected due to chance alone.

As LACE estimates from the doubly-ranked method can be sensitive to specification of the analytic sample, we repeated analyses 100 times for each dataset omitting a small number of individuals in each iteration (12 individuals were removed at random in each iteration), and then combined estimates across iterations using Rubin's rules.

All statistical analyses were performed in R (version 4.3.1), and non-linear Mendelian randomization analyses were performed using the SUMnlmr package[25].

Ethical approval

The HUNT study has ethical approval from The Regional Committee for Medical Research in Norway (REK). Approval for individual projects is regulated in conjunction with The Norwegian Social Science Data Services (NSD). The UK Biobank study has ethical approval from the North West Multi-centre Research Ethics Committee (MREC).

Patient involvement

No patients were involved in setting the research question or the outcome measures, nor were they involved in the design or implementation of the study. No patients were asked to advise on interpretation or writing up of results. There are no specific plans to disseminate the results of the research to study participants or the relevant patient community.

For peer review only - http://bmjopen.bmj.com/site/about/guidelines.xhtml

For peer review only

For peer review only - http://bmjopen.bmj.com/site/about/guidelines.xhtml

RESULTS

Study populations

Baseline characteristics of the participants are provided in **Table 1**. Genetic associations with BMI in strata of the population defined by the doubly-ranked method are displayed in **Supplementary Figure 1**, and the BMI distribution in strata of the population is given in **Supplementary Table 2**. Per 1 standard deviation increase in the genetic score, BMI was 0.358 kg/m^2 greater in the lowest percentile of participants in the HUNT study, and 1.126 kg/m^2 greater in the highest percentile. Equivalent values for UK Biobank were 0.232 kg/m^2 in the lowest percentile and 1.645 kg/m^2 in the highest percentile. It is clear that the genetic associations with BMI differ strongly at different levels of BMI, and so the assumption required for the residual stratification method is violated. We note that similar estimates of the genetic associations with the exposure from the residual stratification method are unreliable, as these estimates from the residual method are similar even if the genetic effect on the exposure varies in the population[18].

Assessment of instrument validity

Associations with traits that are competing risk factors in strata of UK Biobank are displayed in **Figure 1**. As previously observed in the literature for other exposures[26], there are clear patterns in genetic associations with age and sex across strata. While there is no overall association of the genetic score with age or sex, there are associations in several of the strata.

As it is logically impossible that autosomal genetic variants can affect either age or sex, and as age and sex are typically measured without error, these associations must represent the effect of selection into the UK Biobank sample. While concerning, as we adjust for age and sex in our Mendelian randomization analyses, these associations will not lead to strong bias in our causal estimates (see **Supplementary Material** for a simulation study investigation into bias mitigation by adjustment for predictors of selection). For other traits, both the magnitude of associations and patterns in associations are less strong, particularly for education level and occupation, which are important measures of social class. For alcohol status and smoking status, it is impossible to distinguish between genetic associations that reflect pleiotropic or selection effects and those that reflect a causal effect of the exposure (sometimes called “vertical pleiotropy”); the latter do not violate the instrumental variable assumptions. Hence both the associations and any pattern of associations may reflect violations of the instrumental variable assumptions or selection bias, but they could also reflect downstream effects of BMI which vary across strata.

Corresponding associations in the HUNT study are shown in **Supplementary Figure 2**. While there are some patterns in the stratum associations with age and sex, these are less pronounced than for UK Biobank, potentially due to differential selection being less strong in the HUNT study.

Comparison of results from residual and doubly-ranked methods

The shape of the association between genetically-predicted BMI and all-cause mortality is displayed in **Figure 2**, and stratum-specific estimates are provided in **Supplementary Figure 3**. When stratifying the population using the residual method, there is a distinct J-shaped relationship between BMI and mortality, particularly in the UK Biobank study, with minimum risk at a BMI level of around 25 kg/m^2. However, as discussed above, results from the residual stratification method are unreliable here. In contrast, when stratifying using the doubly-ranked method, the relationship between BMI and mortality is far more uncertain at the lower end of the BMI distribution. In the HUNT study, the association is mildly J-shaped, with a null slope up to a BMI of around 28 kg/m^2, and a positive slope

For peer review only - http://bmjopen.bmj.com/site/about/guidelines.xhtml

above this level. For UK Biobank, there is some indication of an upturn in disease risk at very low levels of BMI (the slope of the curve is negative below 20 kg/m^2), but otherwise the relationship is flat (and compatible with a null effect) up to a BMI of around 25 kg/m^2, and has an increasing positive slope above this level. The linearity and trend tests for the residual method indicate evidence supporting non-linearity for UK Biobank ($p_{linearity} = 5\times10^{-4}$, $p_{trend} = 2\times10^{-5}$). In contrast, these tests for the more reliable doubly-ranked method do not provide compelling evidence supporting non-linearity (**Figure 2**). Similar results were observed when inverse-normal rank transforming the exposure (**Supplementary Figure 4**).

Sex-stratified analyses

Sex-stratified analyses from the doubly-ranked method are presented in **Figure 3**, and stratum-specific estimates are provided in **Supplementary Figure 5**. In each case, there is some indication of a J-shaped relationship, but an upturn in disease risk is only evident below 20 kg/m^2 (below 22 kg/m^2 for men in the HUNT study). Otherwise, estimates are generally compatible with the null up to a BMI of 25 kg/m^2, and positive above this level. Evidence for a harmful effect of increases in BMI for both men and women is weaker in the HUNT study, although a harmful effect above 30 kg/m^2 is evident for men. The slope above 25 kg/m^2 is steeper for women than men in UK Biobank. The linearity and trend tests do not provide compelling evidence supporting non-linearity in any analysis (**Figure 3**).

Cause-specific mortality

Analyses investigating cause-specific mortality from the doubly-ranked method in UK Biobank are presented in **Figure 4**, and stratum-specific estimates are provided in **Supplementary Figure 6**. Again, there is some indication of a J-shaped relationship for each outcome. This is most pronounced for cardiovascular mortality, and less evident for cancer and non-cardiovascular non-cancer mortality. An upturn in disease risk at low BMI levels was only evident for cardiovascular mortality, and only below 20 kg/m^2. Otherwise, the relationship was compatible with the null up to around 24 kg/m^2 for cardiovascular mortality, 26 kg/m^2 for and non-cardiovascular non-cancer mortality, and 30 kg/m^2 for cancer mortality, and positive above this level. The slope above 25 kg/m^2 is steepest for cardiovascular mortality, then non-cardiovascular non-cancer, and finally for cancer mortality. The linearity and trend tests do not provide any evidence supporting non-linearity for any outcome, indicating that a non-linear model does not fit the data significantly better than a linear model for any outcome (**Figure 4**).

For peer review only

DISCUSSION

In this manuscript, we have investigated the shape of associations between genetically-predicted BMI and mortality outcomes to provide an indication of the causal effect of BMI on mortality risk at different levels of BMI using non-linear Mendelian randomization. While the curves generally display a J-shaped relationship, evidence for a harmful average effect of decreased BMI on mortality at low BMI levels was limited. Evidence supporting non-linearity from the doubly-ranked method was unconvincing for all mortality outcomes. In UK Biobank, the slope representing the estimated effect of BMI on mortality risk above 25 kg/m^2 was sharper for women than for men, and sharpest for cardiovascular mortality compared with non-cardiovascular non-cancer mortality and cancer mortality. There was evidence for a harmful effect of high BMI on all mortality subtypes.

Our findings challenge “obesity paradox” results from observational epidemiology and previous Mendelian randomization analyses using the residual method, which suggest that increases in BMI might reduce risk of mortality for a substantial proportion of the general population. While the minimum of the estimated curve for all-cause mortality was at a BMI of around 23 kg/m^2 in UK Biobank, the curve was near flat from 22-24 kg/m^2, and was positive from 25 kg/m^2 upwards. Evidence for a harmful effect of decreased BMI at low levels of BMI (as indicated by negative slopes for both the upper and lower confidence limits of the BMI—mortality curve) was only present in some analyses, and only below 20 kg/m^2 when present. Amongst cause-specific mortality outcomes, this was only evident for cardiovascular mortality. In the HUNT study, there was no strong evidence for an effect of BMI on all-cause mortality at low BMI levels in overall analyses, and evidence for a harmful effect of increased BMI above around 28 kg/m^2. A potential reason for the discrepancy is that UK Biobank only contains participants from the age of 40 upwards, whereas the HUNT study includes younger individuals. Hence underweight strata in UK Biobank are more likely to comprise primarily of older, frailer individuals, whereas underweight strata in the HUNT study include more healthy, slim individuals.

Our investigation provides further empirical evidence that the assumptions required for the residual stratification method for non-linear Mendelian randomization are likely to be violated in practice, and that results from the residual method can be substantially different to those from the doubly-ranked method[17]. The ratio between the genetic association with BMI in the highest versus lowest percentile group was 3.1 in the HUNT study and 7.1 in UK Biobank, whereas the assumptions required by the residual method imply that these associations should be constant across the distribution of the exposure. Particularly in UK Biobank, the residual method provided strong evidence for non-linearity in the effect of BMI on mortality risk, giving a J-shaped relationship with the minimum risk level at around 25 kg/m^2. This is similar to the confounded association observed in traditional observational studies[2,3]. In contrast, the doubly-ranked method provided little evidence for non-linearity. While there was some indication of a J-shaped curve in all analyses, estimates at the lower end of the BMI distribution were imprecise. One reason for this is that genetic associations with BMI were weaker at low levels of BMI in the doubly-ranked method, and so stratum-specific Mendelian randomization estimates are less precise in these strata.

Previous investigations into the non-linear shape of the causal relationship between BMI and mortality have used a variety of approaches. Wade *et al* used the residual stratification method for non-linear Mendelian randomization in UK Biobank[27], which showed a J-shaped relationship similar to the one we observe here. Jenkins *et al* investigated associations between a genetic score for BMI and mortality in UK Biobank[5], considering analyses in the full dataset, and restricted to those with a pre-existing morbidity condition. While they did not observe evidence for non-linearity, the genetic score only explains a small proportion of variance in BMI, and so substantial non-linearity may not

For peer review only - http://bmjopen.bmj.com/site/about/guidelines.xhtml

be expected on this scale. The difference between average BMI levels at the lowest versus highest decile of the genetic score is less than 2 kg/m^2. Sulc *et al* investigated the effect of BMI on life expectancy (estimated as the mean of parental lifespan) in a Mendelian randomization framework using a polynomial approach similar to the residual method[28], except that instead of stratifying on the residual exposure, this approach adjusts for the residual exposure. They showed a linear effect of increased BMI on reduced life expectancy, with no indication of non-linearity. However, this approach also has been shown in simulations to be vulnerable to variation in genetic effects on the exposure[18]. Carslake *et al* performed an instrumental variable analysis to investigate the impact of BMI on mortality in the HUNT study[29], but using offspring BMI as an instrument for the BMI of study participants. This analysis did not provide evidence for a harmful effect of having low BMI. A similar analysis was performed in the 1958 British birth cohort, and a similar conclusion was reached[30]. Blond *et al* also performed an instrumental variable analysis using offspring BMI as an instrument for the BMI of study participants in the Copenhagen School Health Records Register[31], showing evidence of positive effects of increases in BMI on mortality at both high and low BMI levels. Finally, Kjøllesdal et al performed an instrumental variable analysis using early adulthood BMI as an instrument for the midlife BMI of study participants in various Norwegian health surveys[32], showing mild J-shaped associations in the relationship between BMI and mortality, but far less pronounced than from observational analyses of the same dataset.

Overall, there is general consensus from different approaches that the adverse relationship of low BMI with mortality is exaggerated in observational analyses. However, variation between these results indicates the importance of modelling assumptions, both for the identification of causal effects, and for the modelling of non-linear relationships. Indeed, in our investigations, if we had used fractional polynomials of degree 1 rather than degree 2, then we would have observed monotonic increasing relationships of BMI with all outcomes. This is because the simpler degree 1 models are dominated by behaviour at the upper end of the BMI distribution, where instruments are strongest and most mortality outcomes occur. The simpler degree 1 polynomials smooth over imprecise negative estimates at the lower end of the BMI distribution, and so do not allow uncertainty in the shape of the distribution (particular at its lower end) to be accurately reflected.

Our analysis has several strengths, but also limitations. The Mendelian randomization design minimizes bias from confounding and reverse causation. The large sample sizes of the HUNT and UK Biobank studies enable powerful analyses, even in strata of the population with low BMI levels. These sample sizes also enable a fine stratification of individuals, meaning that outcome associations can be assessed in strata only consisting of low BMI (<20 kg/m^2) individuals.

However, there are important limitations, which should lead to caution in the interpretation of our findings. As with all Mendelian randomization analyses, findings are dependent on the validity of the genetic variants as instrumental variables. To reduce the scope for population stratification, our analyses were limited to European ancestry participants. Our findings may therefore not be applicable to other populations. Further, recruitment into UK Biobank is dependent on age, sex, and other factors, which leads to bias in Mendelian randomization estimates. While the effect of moderate selection on Mendelian randomization estimates is often slight[33], the extent of selection bias may differ between strata, leading to differential bias across stratum-specific estimates[26]. It is likely that age and sex are the strongest predictors of study participants, and differential genetic associations with these traits were observed in UK Biobank. For other traits, patterns in genetic associations across strata were less evident, and may reflect downstream effects of BMI rather than instrument invalidity due to pleiotropy or selection bias. As the HUNT study focused on a specific geographic area of Norway, it achieved much higher recruitment rates (around 70%, compared with

For peer review only - http://bmjopen.bmj.com/site/about/guidelines.xhtml

5% in UK Biobank), and so should be less affected by selection bias. This was corroborated by our analyses, as genetic associations in strata were weaker in the HUNT study. While genetic associations are protected from reverse causation, strata membership is determined by BMI levels, and hence could be subject to reverse causation. In particular, low BMI strata could contain a large proportion of individuals whose BMI levels are low because of comorbidity. However, genetic associations should still provide a reliable guide as to the extent and direction of any causal effect of BMI in these strata. Finally, all estimates represent averages across strata of the population; individual effects of raising or lowering BMI may vary between individuals. Differences in estimates for different strata may reflect the changing composition of the strata, as the characteristics of those with BMI less than 20 kg/m^2 are likely to be different to those with higher BMI. A non-linear curve may reflect a combination of different effects in different subgroups of the population, rather than that the relationship between BMI and mortality risk is non-linear for any individual in the population[34].

In conclusion, non-linear Mendelian randomization analyses using the doubly-ranked stratification method provide strong evidence for harmful effects of increased BMI on mortality above 25 kg/m^2. Evidence for a harmful effect of low BMI was only present in some analyses, and where present, only below 20 kg/m^2.

For peer review only

For peer review only - http://bmjopen.bmj.com/site/about/guidelines.xhtml

Table 1: Baseline characteristics of study participants in HUNT and UK Biobank studies

	HUNT	UK Biobank
Number of participants (n)	56,150	366,385
Men (%)	47.1	45.9
Age at baseline in years (SD)	49.6 (16.6)	56.7 (8.0)
Number of deaths (n)	18,836	25,021
- Cardiovascular mortality events (n)	-	5212
- Cancer mortality events (n)	-	12,880
- Other (non-cardiovascular non-cancer) events (n)	-	6692
BMI in kg/m^2 (SD)	26.3 (4.1)	27.4 (4.8)
Median follow-up years	18.5	7.0
Ever-smokers (%)	55.9	46.1

BMI: body mass index; SD: standard deviation; Data are given as number of subjects (n), percentage (%), median, or mean (standard deviation). For 237 deaths in UK Biobank, sufficient data were not available to classify the event as cardiovascular, cancer, or non-cardiovascular non-cancer.

For peer review only

Figure 1: Genetic associations with potential competing risk factors in strata of the UK Biobank population

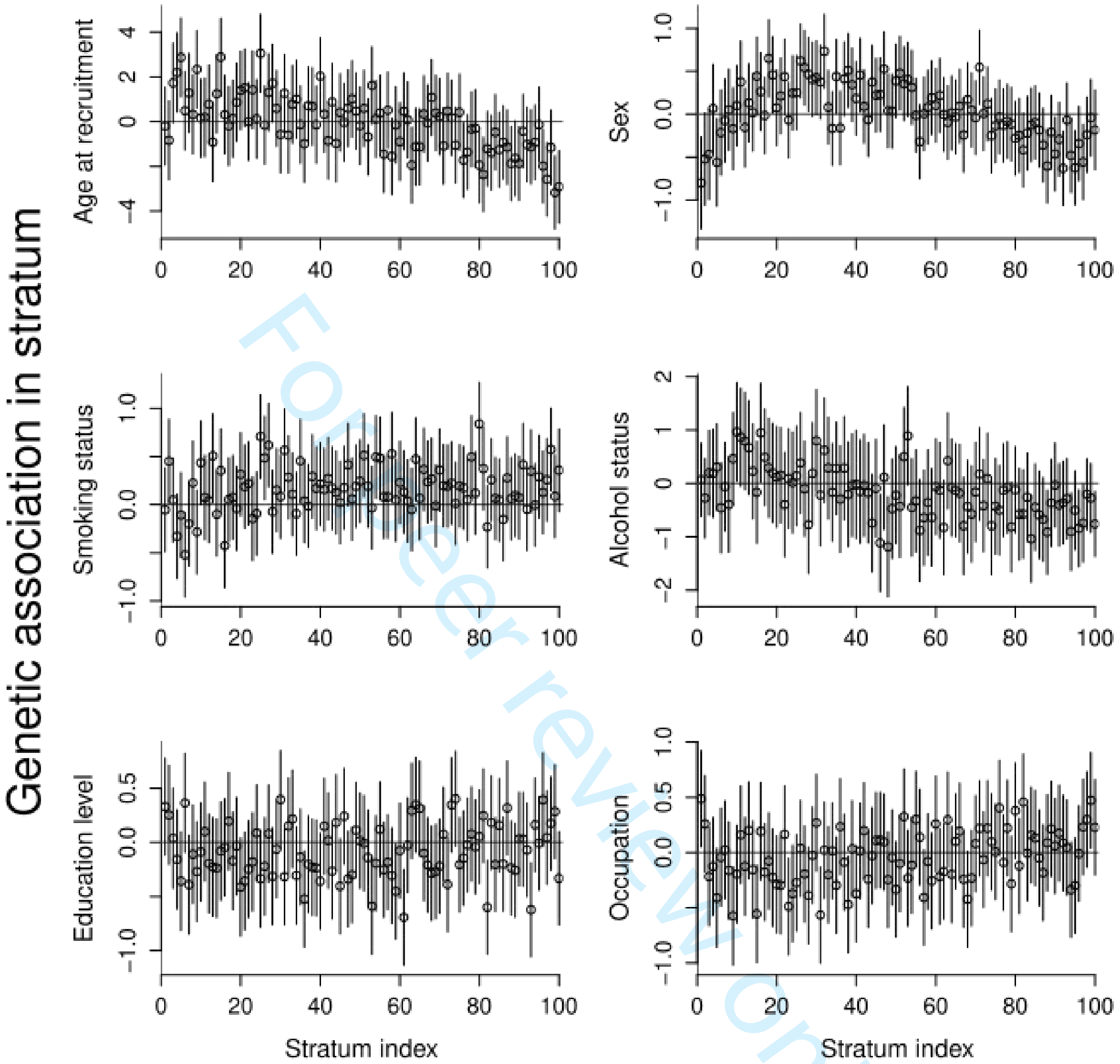

Points represent association estimates from linear (age at recruitment, years) or logistic regression (all other traits). Error bars represent 95% confidence intervals. Stratum 1 has lowest average BMI levels, stratum 100 has highest average BMI levels.

For peer review only - http://bmjopen.bmj.com/site/about/guidelines.xhtml

Figure 2: Non-linear Mendelian randomization – dose-response curve between body mass index and all-cause mortality. Using the doubly-ranked method: A) HUNT, B) UK Biobank; using the residual method (unreliable when the genetic effect on the exposure varies): C) HUNT, D) UK Biobank. Gradient at each point of the curve is the localized average causal effect. Grey lines represent 95% confidence intervals. The reference value for BMI was taken as 25 kg/m².

A) HUNT, doubly-ranked method:

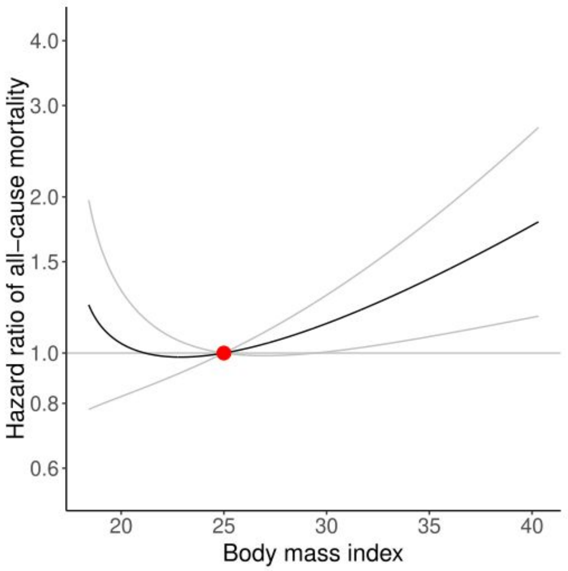

p-value for linearity: 0.35
p-value for trend: 0.35

B) UK Biobank, doubly-ranked method:

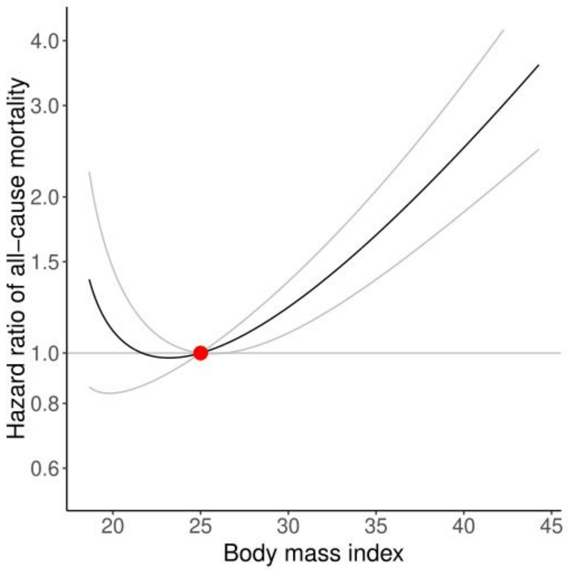

p-value for linearity: 0.064
p-value for trend: 0.056

C) HUNT, residual method:

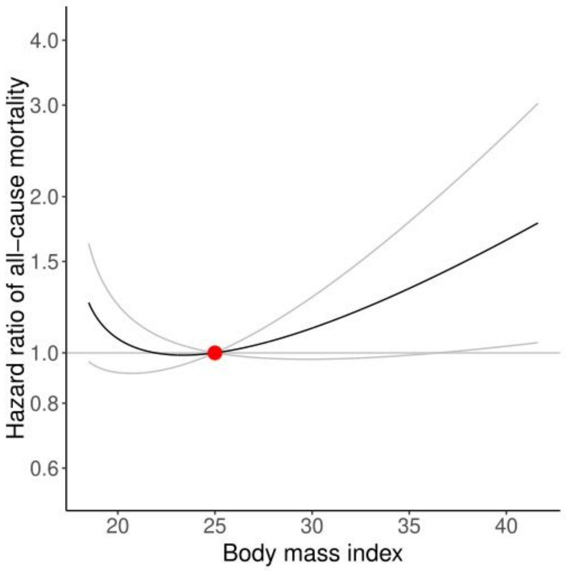

p-value for linearity: 0.12
p-value for trend: 0.12

D) UK Biobank, residual method:

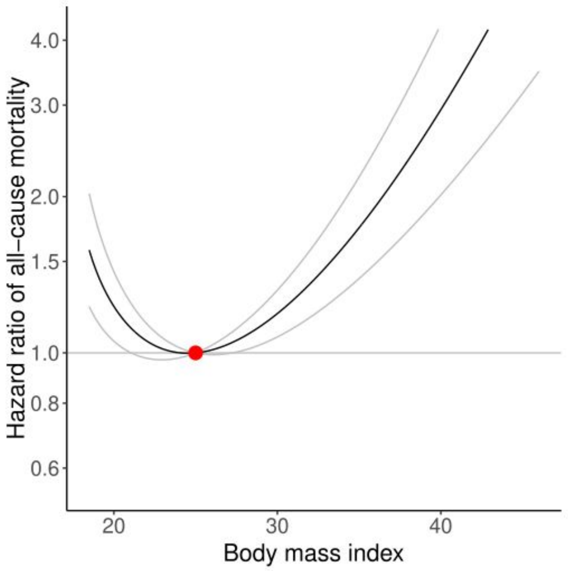

p-value for linearity: 5×10^{-4}
p-value for trend: 2×10^{-5}

For peer review only - http://bmjopen.bmj.com/site/about/guidelines.xhtml

Figure 3: Non-linear Mendelian randomization – dose-response curve between body mass index and all-cause mortality in men and women respectively using doubly-ranked method. A) HUNT, men only; B) HUNT, women only; C) UK Biobank, men only; D) UK Biobank, women only. Gradient at each point of the curve is the localized average causal effect. Grey lines represent 95% confidence intervals. The reference value for BMI was taken as 25 kg/m^2.

A) HUNT, men only:

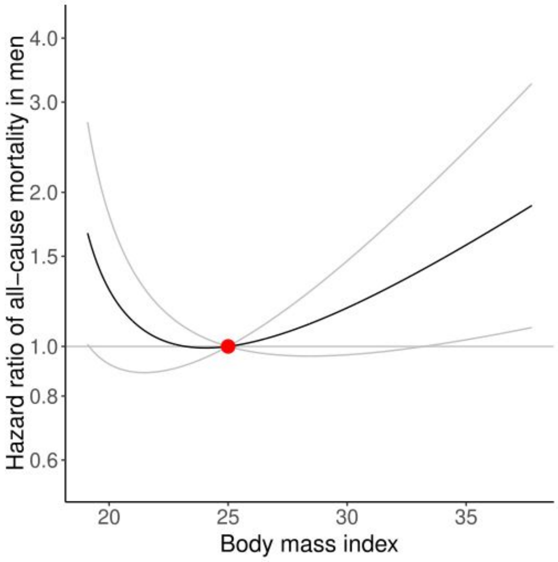

p-value for linearity: 0.050
p-value for trend: 0.22

B) HUNT, women only:

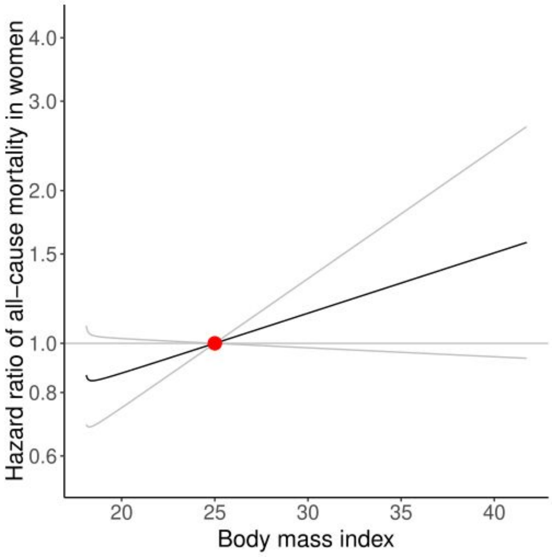

p-value for linearity: 0.99
p-value for trend: 0.79

C) UK Biobank, men only:

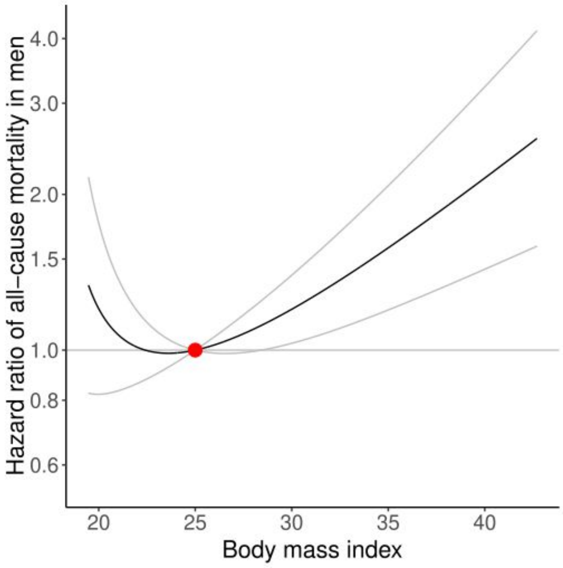

p-value for linearity: 0.18
p-value for trend: 0.18

D) UK Biobank, women only:

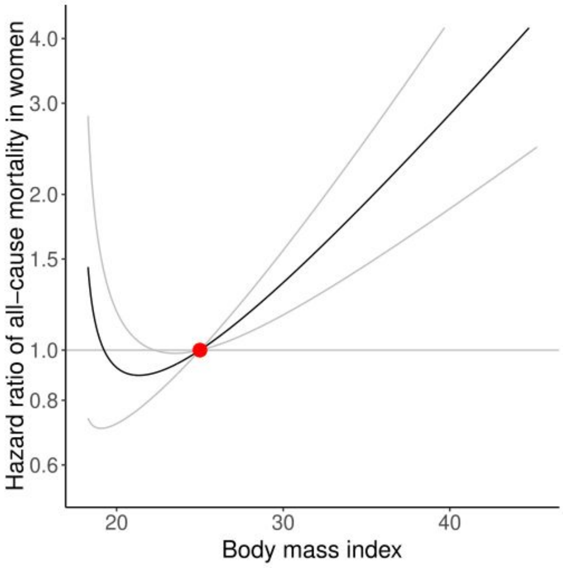

p-value for linearity: 0.21
p-value for trend: 0.18

For peer review only - http://bmjopen.bmj.com/site/about/guidelines.xhtml

Figure 4: Non-linear Mendelian randomization in UK Biobank – dose-response curve between body mass index and cause-specific mortality using doubly-ranked method. A) Cardiovascular disease mortality; B) Cancer mortality. C) Mortality due to other causes (non-cardiovascular, non-cancer). Gradient at each point of the curve is the localized average causal effect. Grey lines represent 95% confidence intervals. The reference value for BMI was taken as 25 kg/m^2.

A: Cardiovascular mortality

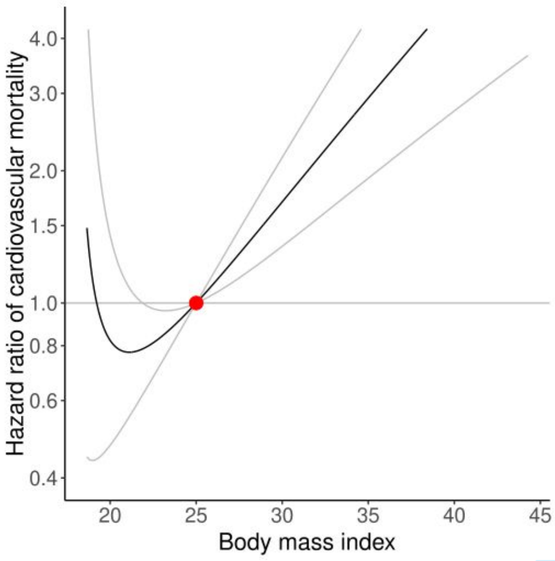

p-value for linearity: 0.99
p-value for trend: 0.92

B: Cancer mortality

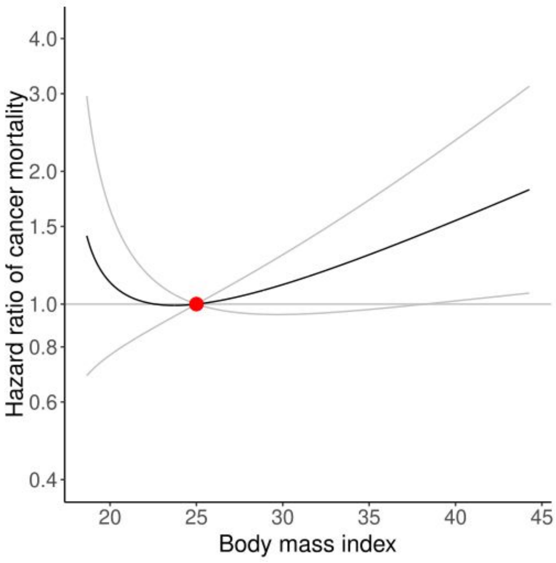

p-value for linearity: 0.39
p-value for trend: 0.38

C: Other (non-cardiovascular, non-cancer) mortality

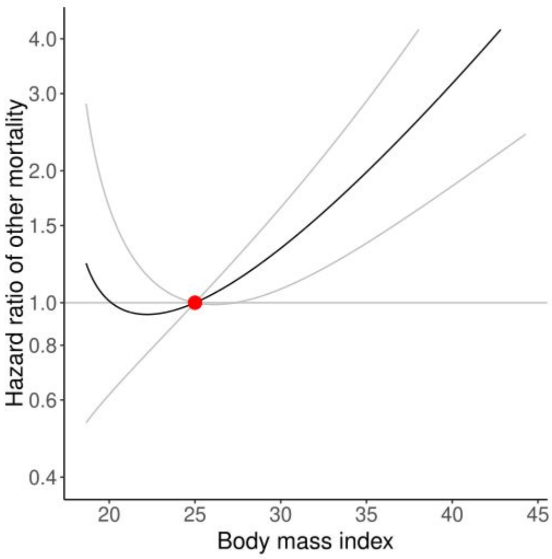

p-value for linearity: 0.20
p-value for trend: 0.19

For peer review only - http://bmjopen.bmj.com/site/about/guidelines.xhtml

REFERENCES

1. Aune D, Sen A, Prasad M, et al. BMI and all cause mortality: systematic review and non-linear dose-response meta-analysis of 230 cohort studies with 3.74 million deaths among 30.3 million participants. *BMJ*. 2016;353:i2156. doi:10.1136/bmj.i2156
2. Bhaskaran K, dos-Santos-Silva I, Leon DA, Douglas IJ, Smeeth L. Association of BMI with overall and cause-specific mortality: a population-based cohort study of 3.6 million adults in the UK. *The Lancet Diabetes & Endocrinology*. 2018;6(12):944-953. doi:10.1016/S2213-8587(18)30288-2
3. Global BMI Mortality Collaboration, Di Angelantonio E, Bhupathiraju Sh N, et al. Body-mass index and all-cause mortality: individual-participant-data meta-analysis of 239 prospective studies in four continents. *Lancet*. Aug 20 2016;388(10046):776-86. doi:10.1016/s0140-6736(16)30175-1
4. Flegal KM, Kit BK, Orpana H, Graubard BI. Association of All-Cause Mortality With Overweight and Obesity Using Standard Body Mass Index Categories: A Systematic Review and Meta-analysis. *JAMA*. 2013;309(1):71-82. doi:10.1001/jama.2012.113905
5. Jenkins DA, Wade KH, Carslake D, et al. Estimating the causal effect of BMI on mortality risk in people with heart disease, diabetes and cancer using Mendelian randomization. *International journal of cardiology*. 2021;330:214-220. doi:10.1016/j.ijcard.2021.02.027
6. Visaria A, Setoguchi S. Body mass index and all-cause mortality in a 21st century U.S. population: A National Health Interview Survey analysis. *PLOS ONE*. 2023;18(7):e0287218. doi:10.1371/journal.pone.0287218
7. Davey Smith G, Ebrahim S. 'Mendelian randomization': can genetic epidemiology contribute to understanding environmental determinants of disease? *International Journal of Epidemiology*. 2003;32(1):1-22.
8. Lawlor DA, Harbord RM, Sterne JA, Timpson N, Davey Smith G. Mendelian randomization: using genes as instruments for making causal inferences in epidemiology. *Statistics in Medicine*. 2008;27(8):1133-63. doi:10.1002/sim.3034
9. Thanassoulis G, O'Donnell CJ. Mendelian randomization: nature's randomized trial in the post-genome era. *JAMA*. 2009;301(22):2386-2388.
10. Burgess S, Swanson SA, Labrecque JA. Are Mendelian randomization investigations immune from bias due to reverse causation? *European Journal of Epidemiology*. 2021;36(3):253-257.
11. Martens EP, Pestman WR, de Boer A, Belitser SV, Klungel OH. Instrumental variables: application and limitations. *Epidemiology*. 2006:260-267.
12. Staley JR, Burgess S. Semiparametric methods for estimation of a nonlinear exposure-outcome relationship using instrumental variables with application to Mendelian randomization. *Genet Epidemiol*. May 2017;41(4):341-352. doi:10.1002/gepi.22041
13. Burgess S, Davies NM, Thompson SG. Instrumental variable analysis with a nonlinear exposure-outcome relationship. *Epidemiology*. Nov 2014;25(6):877-85.
14. Cole SR, Platt RW, Schisterman EF, Chu H, Westreich D, Richardson D, Poole C. Illustrating bias due to conditioning on a collider. *International Journal of Epidemiology*. 2010;39(2):417-420.
15. Small DS. Commentary: Interpretation and Sensitivity Analysis for the Localized Average Causal Effect Curve. *Epidemiology*. 2014;25(6)
16. Davey Smith G. Mendelian randomisation and vitamin D: the importance of model assumptions. *The Lancet Diabetes & Endocrinology*. 2023;11(1):14. doi:https://doi.org/10.1016/S2213-8587(22)00345-X
17. Burgess S. Violation of the constant genetic effect assumption can result in biased estimates for non-linear Mendelian randomization. *Human Heredity*. 2022;(In Press)doi:10.1159/000531659
18. Tian H, Mason AM, Liu C, Burgess S. Relaxing parametric assumptions for non-linear Mendelian randomization using a doubly-ranked stratification method. *PLOS Genetics*. 2023;19(6):e1010823. doi:10.1101/2022.06.28.497930
19. Sun Y-Q, Burgess S, Staley JR, et al. Body mass index and all cause mortality in HUNT and UK Biobank studies: linear and non-linear mendelian randomisation analyses. *British Medical Journal*. 2019;364:l1042. doi:10.1136/bmj.l1042

For peer review only - http://bmjopen.bmj.com/site/about/guidelines.xhtml

20. Krokstad S, Langhammer A, Hveem K, et al. Cohort Profile: The HUNT Study, Norway. *International Journal of Epidemiology*. 2013;42(4):968-977. doi:10.1093/ije/dys095
21. Sudlow C, Gallacher J, Allen N, et al. UK Biobank: an open access resource for identifying the causes of a wide range of complex diseases of middle and old age. *PLOS Medicine*. 2015;12(3):e1001779. doi:10.1371/journal.pmed.1001779
22. Astle WJ, Elding H, Jiang T, et al. The allelic landscape of human blood cell trait variation and links to common complex disease. *Cell*. 2016;167(5):1415-1429. doi:10.1016/j.cell.2016.10.042
23. Locke AE, Kahali B, Berndt SI, et al. Genetic studies of body mass index yield new insights for obesity biology. *Nature*. 2015;518(7538):197-206. doi:10.1038/nature14177
24. Carreras-Torres R, Johansson M, Haycock PC, Relton CL, Davey Smith G, Brennan P, Martin RM. Role of obesity in smoking behaviour: Mendelian randomisation study in UK Biobank. *BMJ*. 2018;361:k1767.
25. Mason AM, Burgess S. Software Application Profile: SUMnlmr, an R package that facilitates flexible and reproducible non-linear Mendelian randomization analyses. *International Journal of Epidemiology*. 2022;51(6):2014-2019. doi:10.1093/ije/dyac150
26. Hamilton FW, Hughes DA, Spiller W, Tilling K, Davey Smith G. Non-linear mendelian randomization: evaluation of biases using negative controls with a focus on BMI and Vitamin D. *medRxiv*. 2023:2023.08.21.23293658. doi:10.1101/2023.08.21.23293658
27. Wade KH, Carslake D, Sattar N, Davey Smith G, Timpson NJ. BMI and Mortality in UK Biobank: Revised Estimates Using Mendelian Randomization. *Obesity*. 2018;26(11):1796-1806. doi:doi:10.1002/oby.22313
28. Sulc J, Sjaarda J, Kutalik Z. Polynomial Mendelian randomization reveals non-linear causal effects for obesity-related traits. *HGG Advances*. 2022;3(3):100124. doi:10.1016/j.xhgg.2022.100124
29. Carslake D, Davey Smith G, Gunnell D, Davies N, Nilsen TIL, Romundstad P. Confounding by ill health in the observed association between BMI and mortality: evidence from the HUNT Study using offspring BMI as an instrument. *International Journal of Epidemiology*. 2018;47(3):760-770.
30. Hyppönen E, Carslake D, Berry DJ, Power C, Davey Smith G. Estimating the influence of body mass index (BMI) on mortality using offspring BMI as an instrumental variable. *International Journal of Obesity*. 2022;46(1):77-84.
31. Blond K, Carslake D, Gjærde LK, Vistisen D, Sørensen TIA, Smith GD, Baker JL. Instrumental variable analysis using offspring BMI in childhood as an indicator of parental BMI in relation to mortality. *Scientific Reports*. 2021;11(1):22408.
32. Kjøllesdal MKR, Davey Smith G, Ariansen I, Kinge JM, Degerud E, Næss Ø. The association between BMI and mortality using early adulthood BMI as an instrumental variable for midlife BMI. *Scientific Reports*. 2018/07/31 2018;8(1):11499.
33. Gkatzionis A, Burgess S. Contextualizing selection bias in Mendelian randomization: how bad is it likely to be? *International Journal of Epidemiology*. 2018;48(3):691-701. doi:10.1093/ije/dyy202
34. Small DS. Commentary: Interpretation and sensitivity analysis for the localized average causal effect curve. *Epidemiology*. 2014;25(6):886-888.

For peer review only

For peer review only - http://bmjopen.bmj.com/site/about/guidelines.xhtml

Author contributions: Conceptualization: Burgess, Sun, Mai; Formal analysis: Burgess, Sun; Methodology: all authors; Writing – Original Draft: Burgess; Writing – Review & Editing: all authors.

STROBE statement: Included in submission.

Data sharing statement: Data from the HUNT and UK Biobank studies are available on application to any bona fide scientific researcher. Summary statistics for genetic associations with the exposure and outcome in strata for UK Biobank are available at [to be done after initial review].

For peer review only

For peer review only - http://bmjopen.bmj.com/site/about/guidelines.xhtml

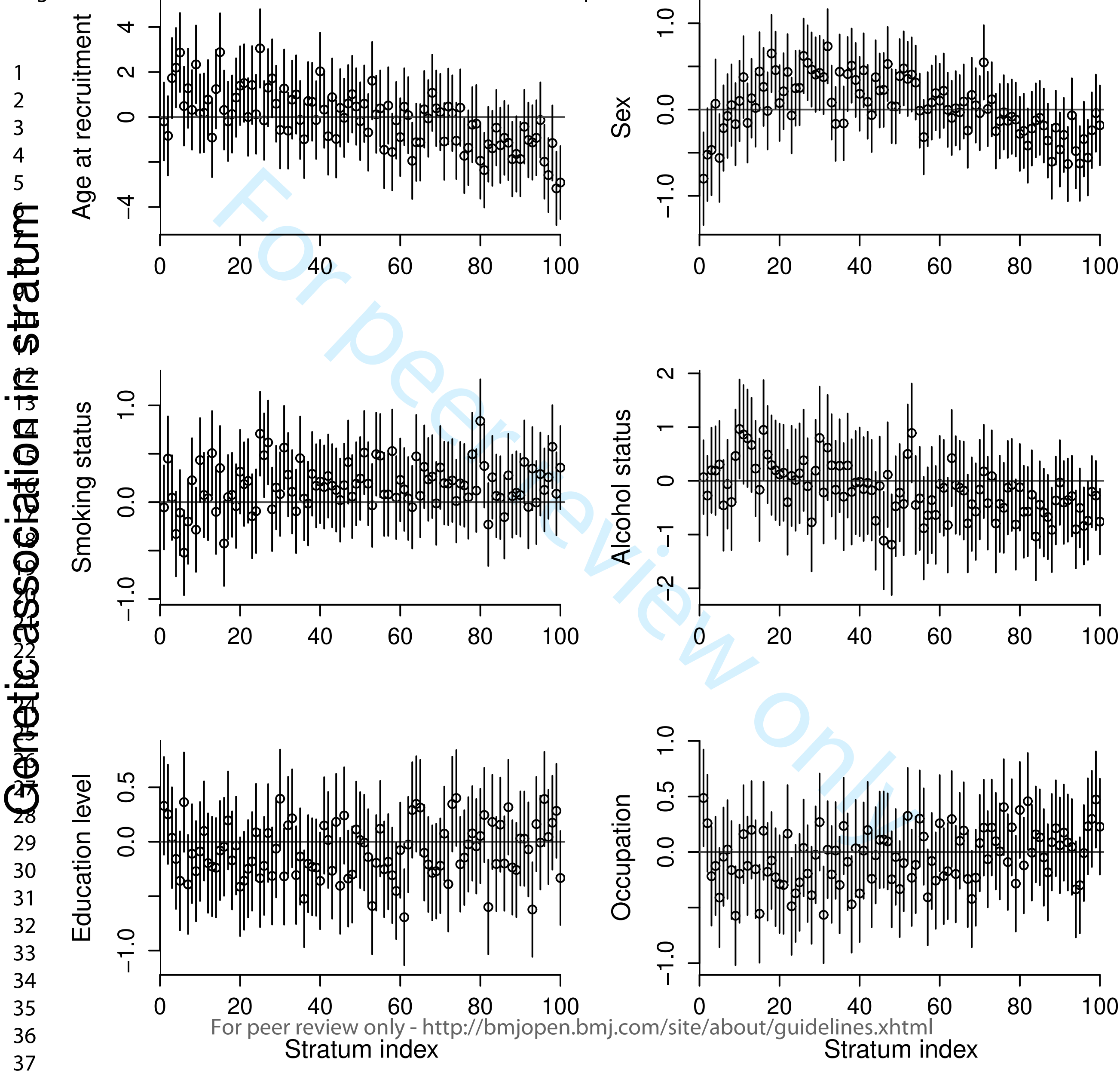

For peer review only

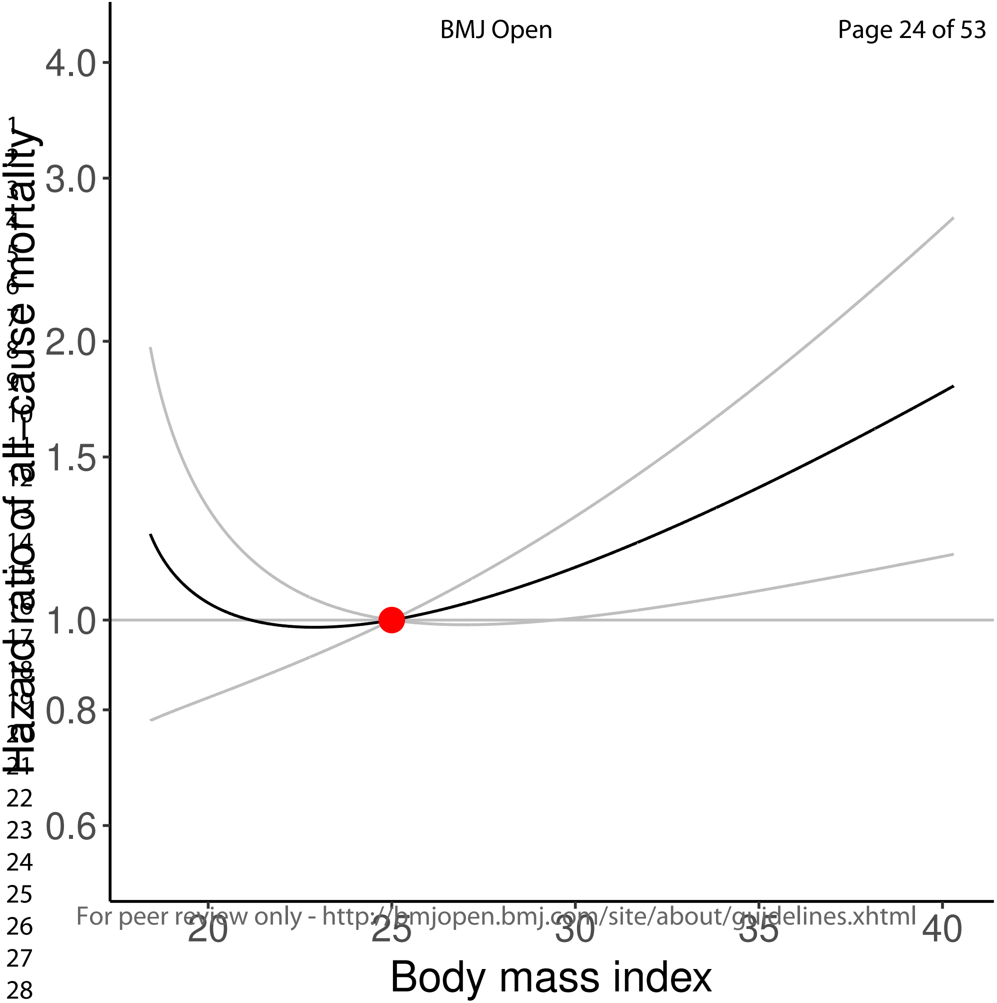
Hazard ratio of all−cause mortality
4.0
3.0
2.0
1.5
1.0
0.8
0.6
20
25
30
35
40
Body mass index

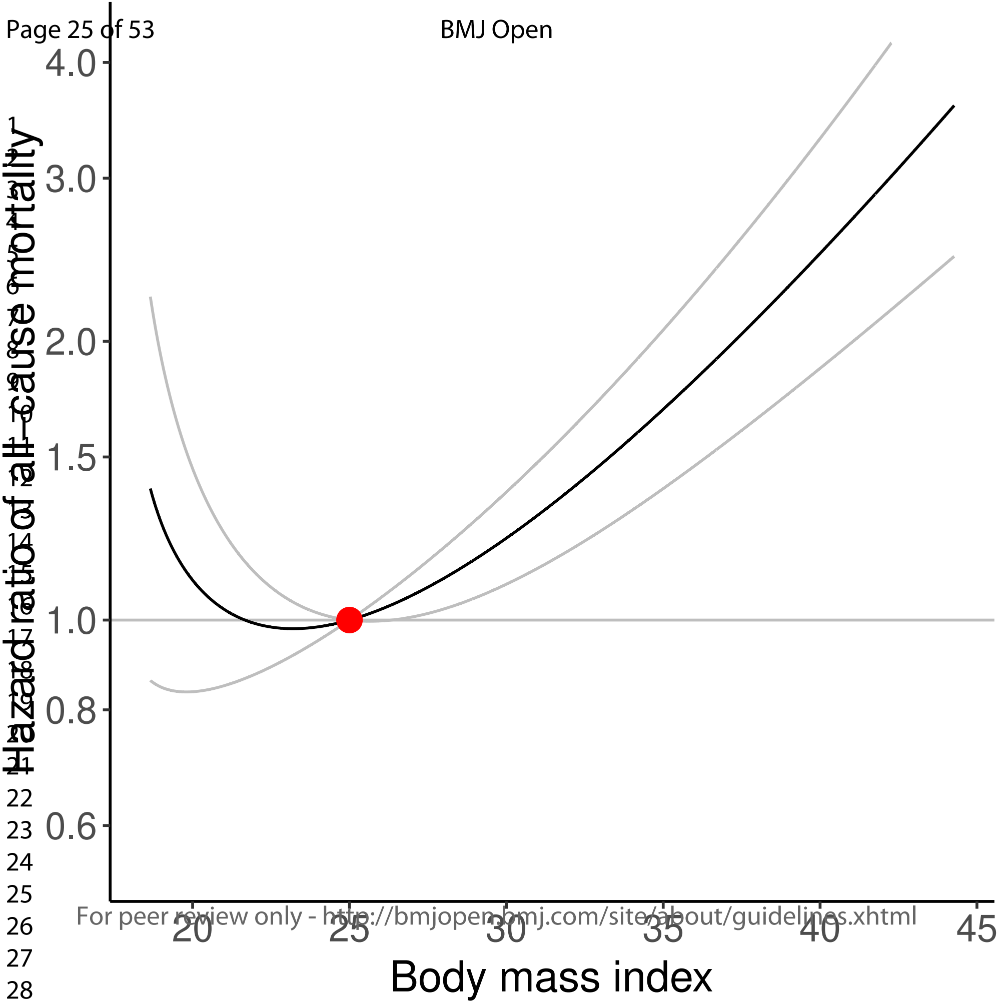
Hazard ratio of all−cause mortality
4.0
3.0
2.0
1.5
1.0
0.8
0.6
20
25
30
35
40
45
Body mass index

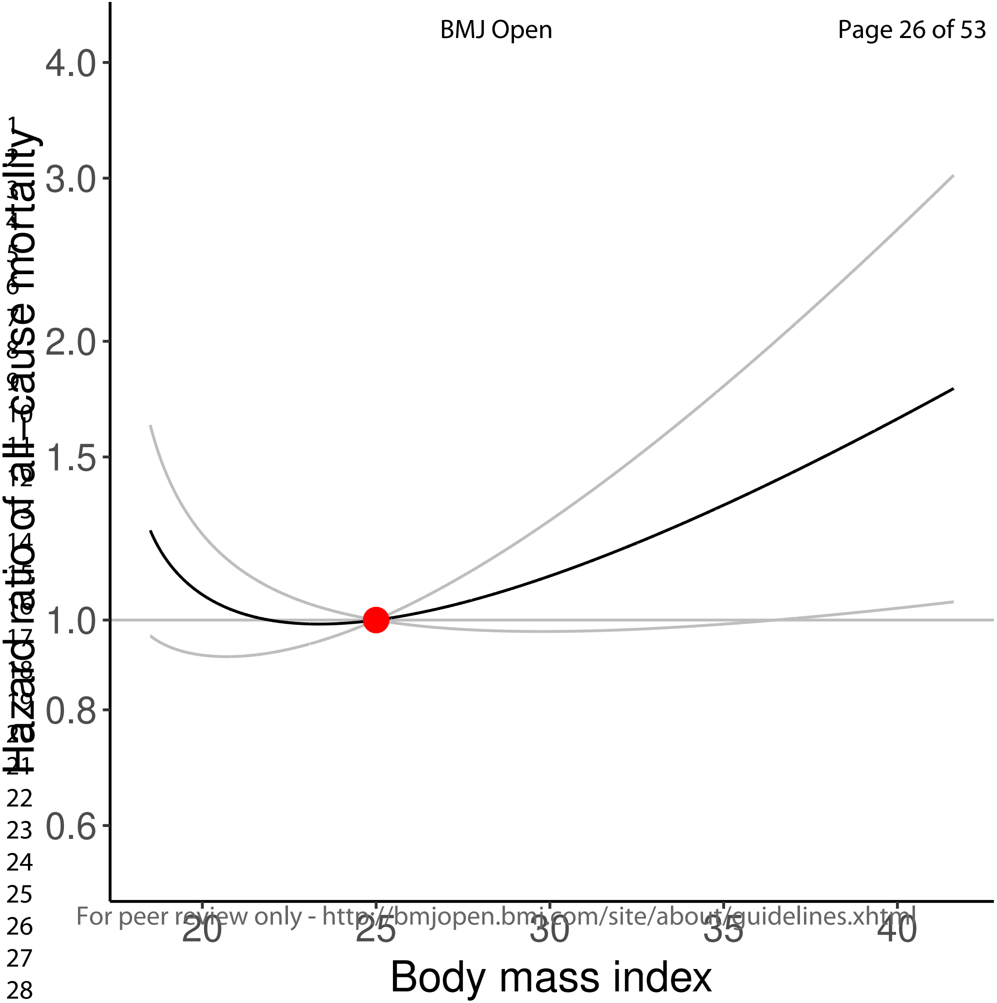

For peer review only - http://bmjopen.bmj.com/site/about/guidelines.xhtml

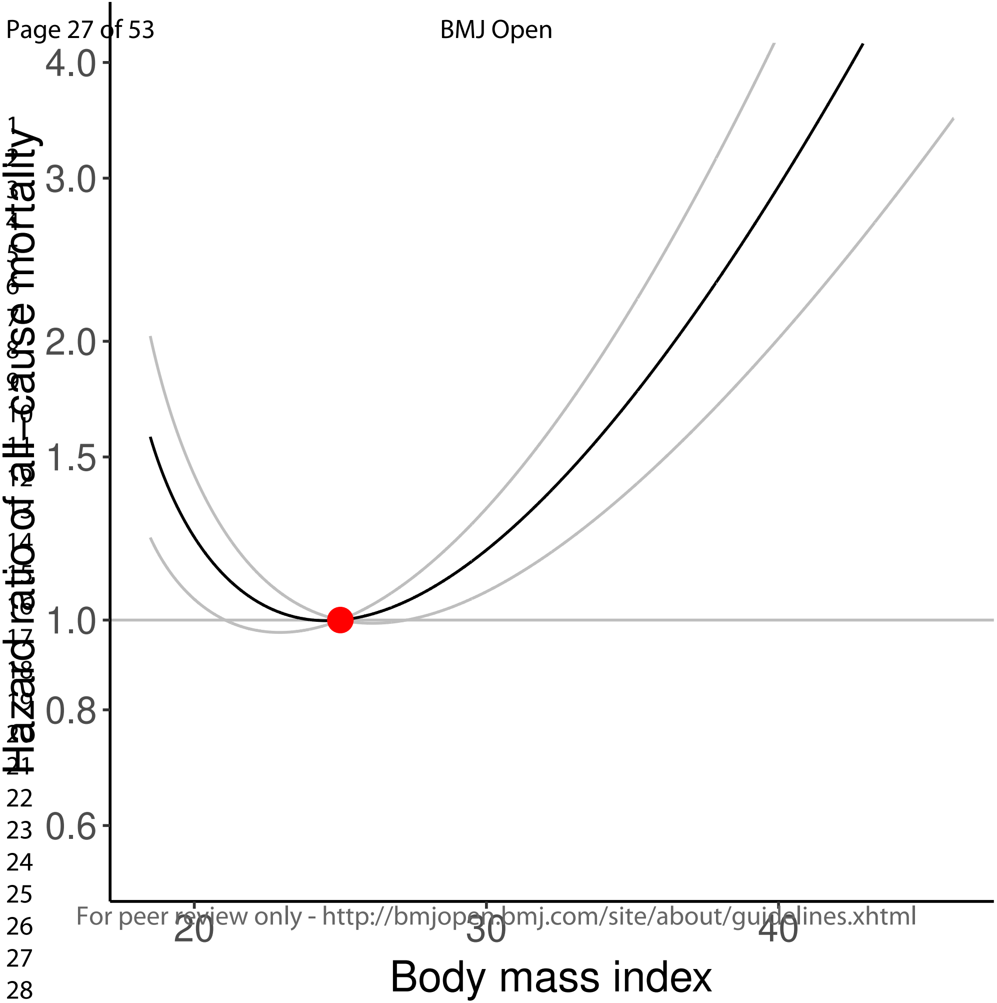
Hazard ratio of all−cause mortality
4.0
3.0
2.0
1.5
1.0
0.8
0.6
20
30
40
Body mass index

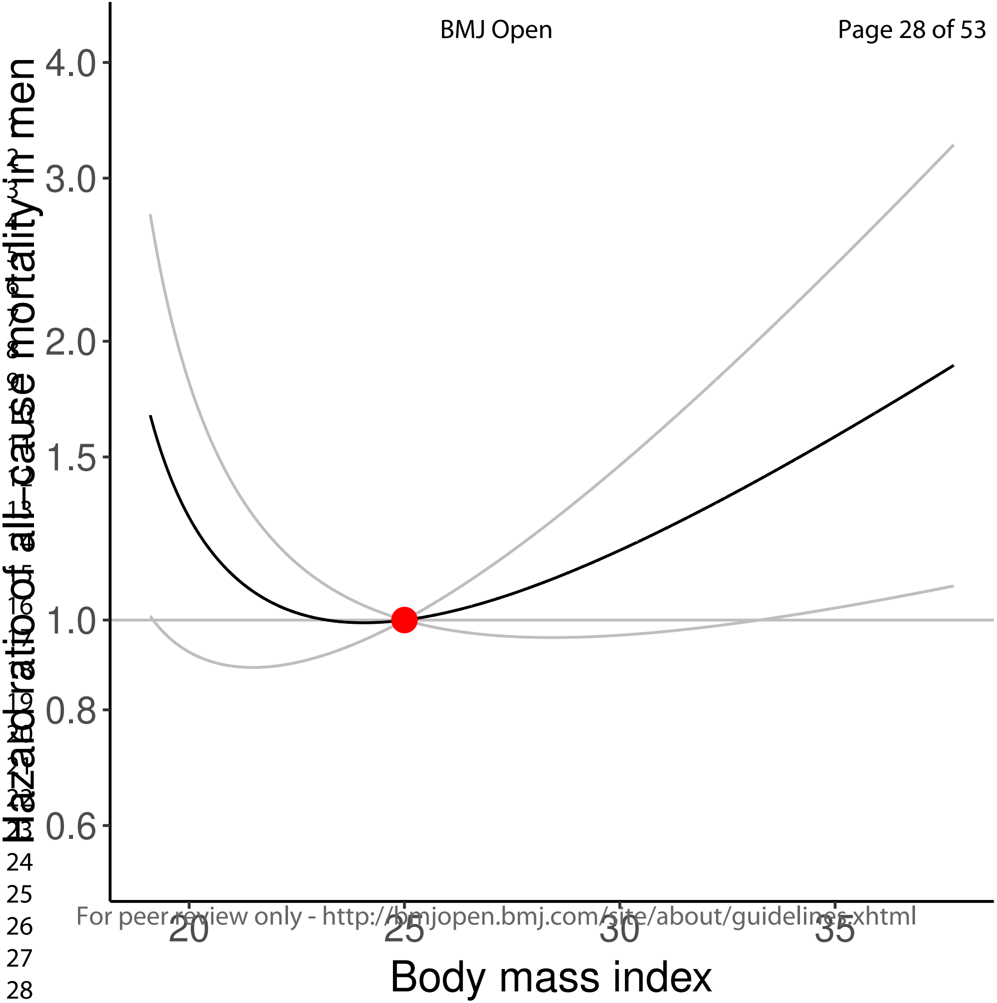
Hazard ratio of all−cause mortality in men
4.0
3.0
2.0
1.5
1.0
0.8
0.6
20
25
30
35
Body mass index

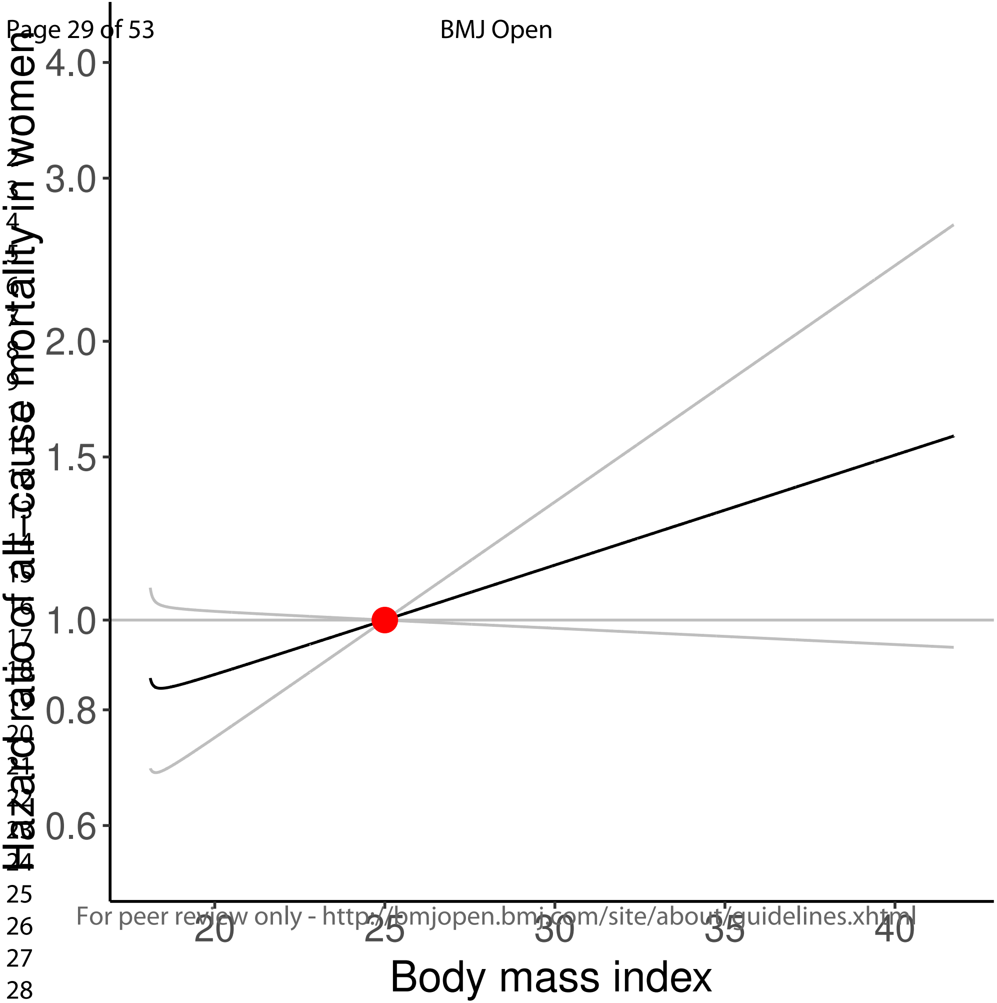
Hazard ratio of all−cause mortality in women
4.0
3.0
2.0
1.5
1.0
0.8
0.6
20
25
30
35
40
Body mass index

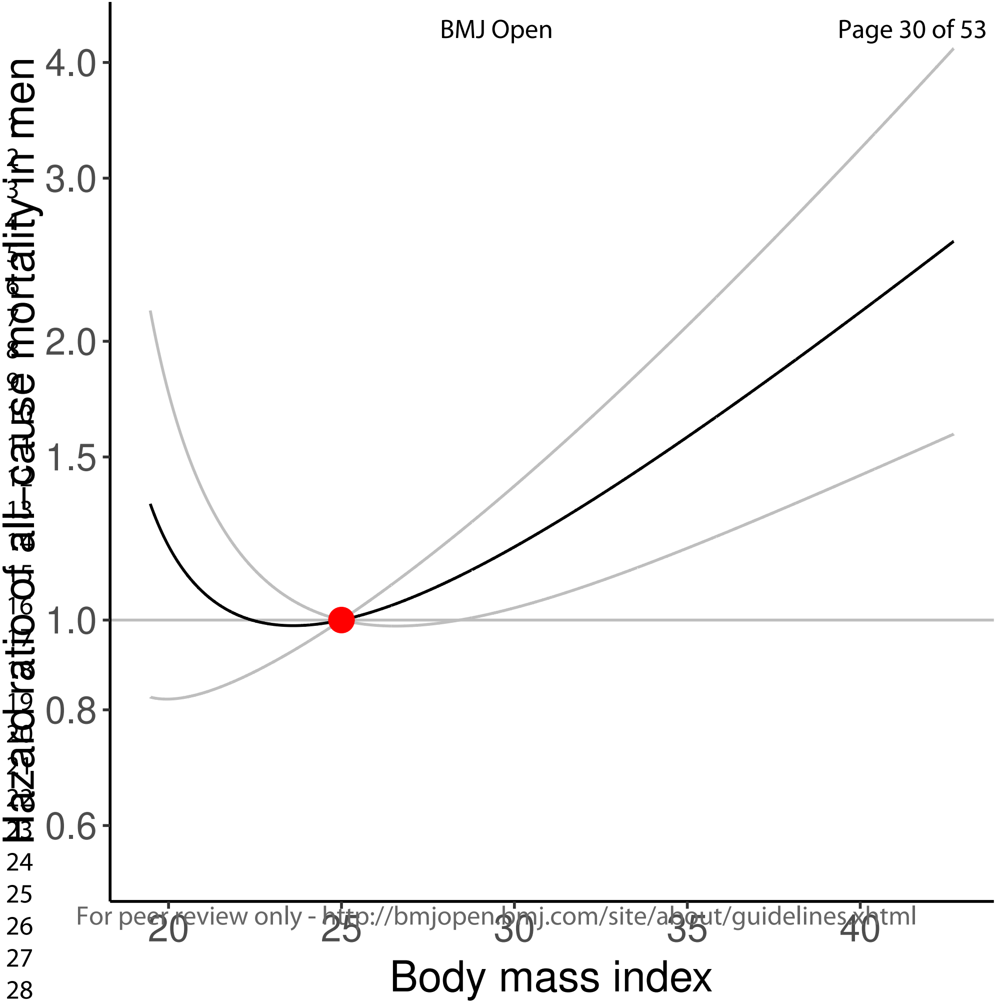
Hazard ratio of all-cause mortality in men
4.0
3.0
2.0
1.5
1.0
0.8
0.6
20
25
30
35
40
Body mass index

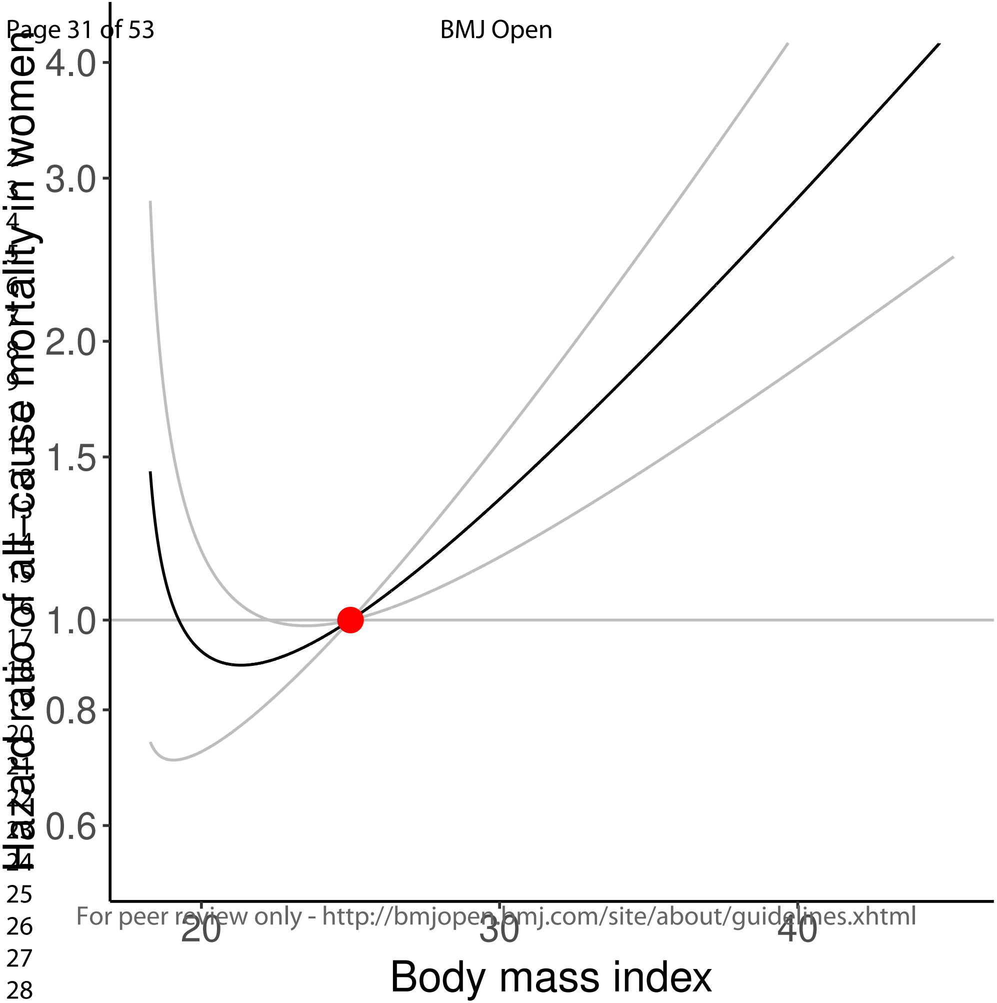
Hazard ratio of all−cause mortality in women
4.0
3.0
2.0
1.5
1.0
0.8
0.6
20
30
40
Body mass index

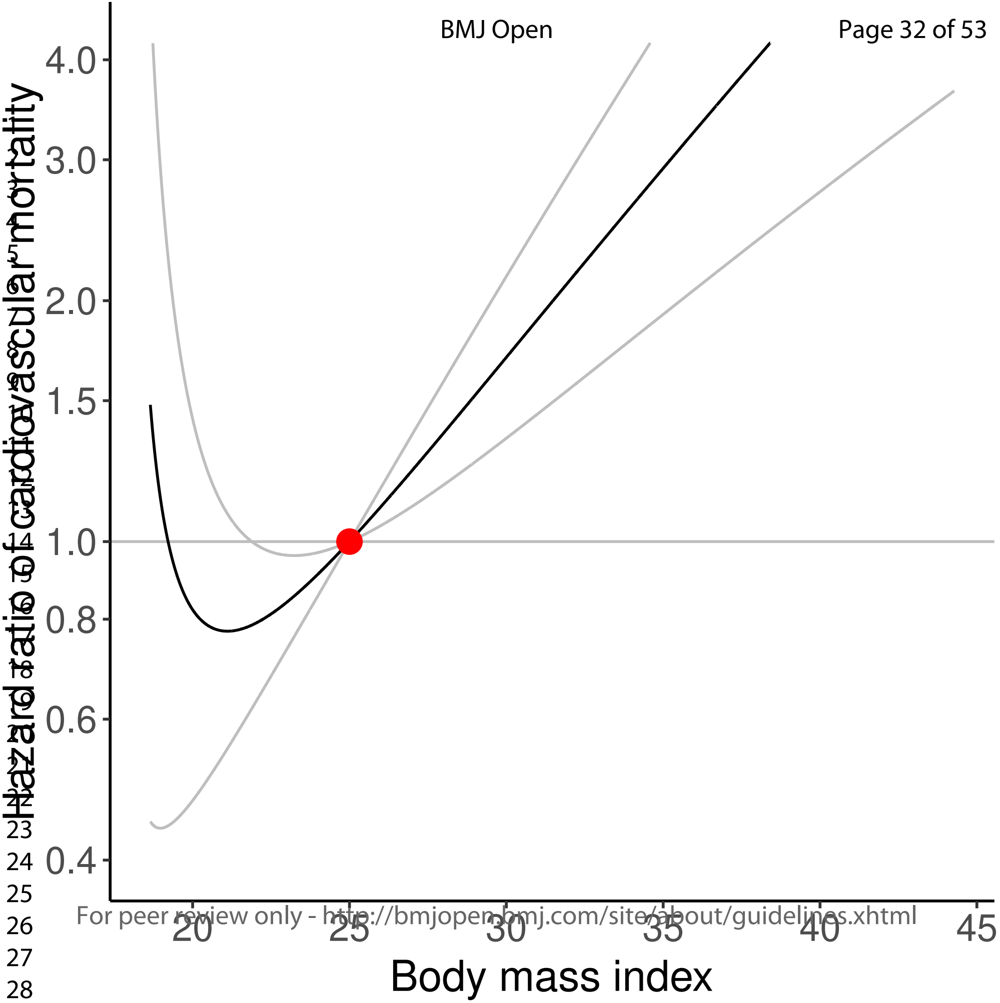
Hazard ratio of cardiovascular mortality
4.0
3.0
2.0
1.5
1.0
0.8
0.6
0.4
20
25
30
35
40
45
Body mass index

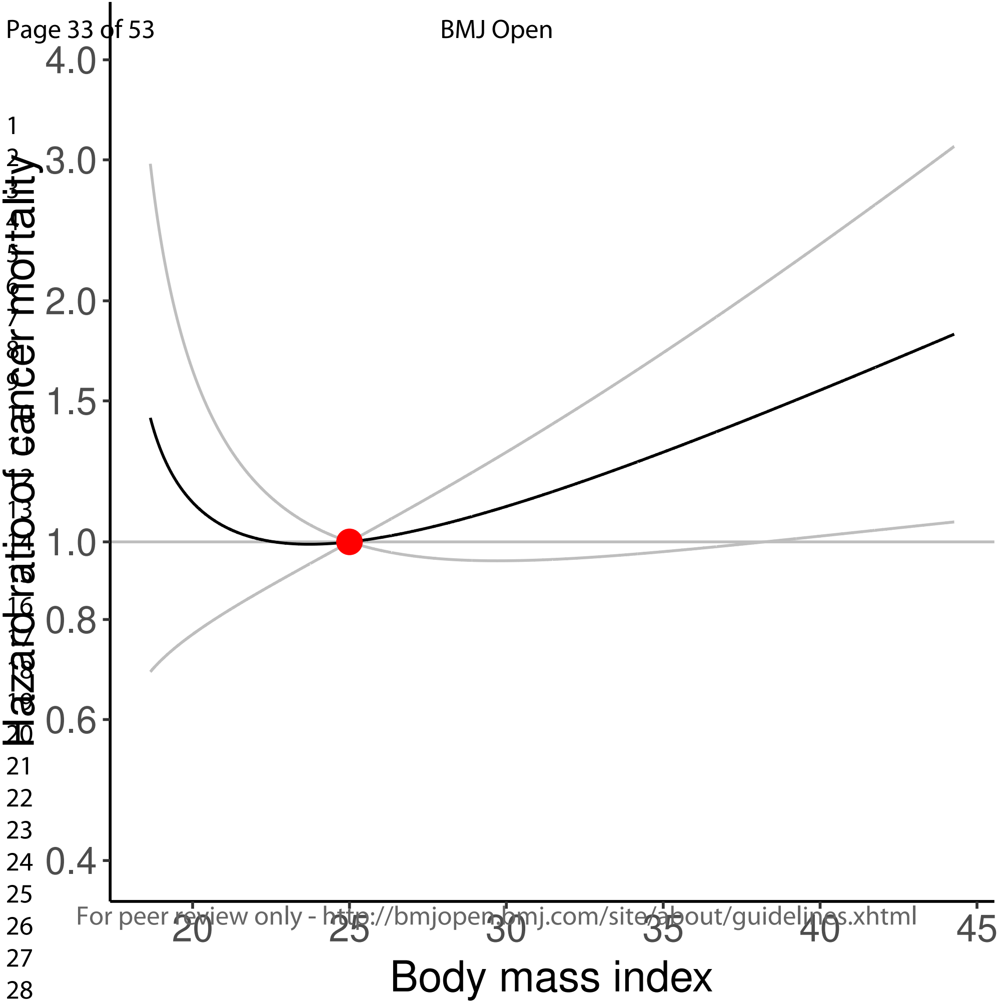

Hazard ratio of cancer mortality
4.0
3.0
2.0
1.5
1.0
0.8
0.6
0.4
20
25
30
35
40
45
Body mass index

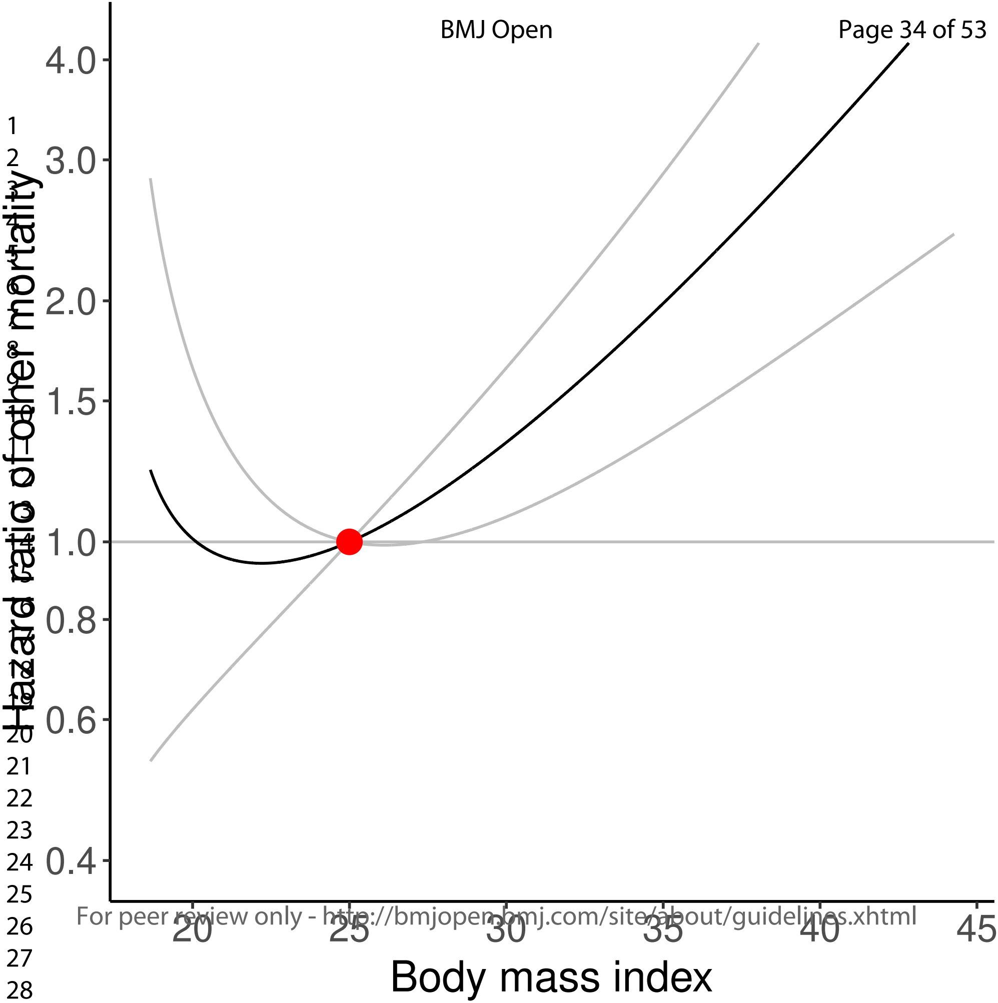
Hazard ratio of other mortality
4.0
3.0
2.0
1.5
1.0
0.8
0.6
0.4
20
25
30
35
40
45
Body mass index

SUPPLEMENTARY MATERIAL

We here perform a simulation study investigating whether adjusting genetic associations for factors that influence selection reduces selection bias within strata in a non-linear Mendelian randomization framework. We generate simulated data for 1,000,000 individuals on a genetic variant *G*, an exposure *X*, an outcome *Y*, and competing risk factors *U* and *V*. For simplicity, all variables are modelled as continuous and normally distributed.

We consider two scenarios. In each scenario, the genetic variant is a valid instrumental variable in the population as a whole, and there is no true effect of the exposure on the outcome. This implies that the genetic variant should not be associated with the outcome. We estimate the association between the genetic variant and the outcome in a selected subset of the population.

In scenario 1, the data-generating model is:

$$G_i \sim Normal(0,1)$$
$$U_i \sim Normal(0,1)$$
$$V_i \sim Normal(0,1)$$
$$X_i \sim Normal(0.2\,G_i + 0.4\,U_i + 0.4V_i, 1)$$
$$Y_i \sim Normal(-0.5\,U_i + 0.2\,V_i, 1)$$

Participants (indexed by the *i* subscript) are selected into the sample with probability $expit(-2 + U_i + X_i)$, where $expit(x) = \frac{\exp(x)}{1+\exp(x)}$ is the inverse of the logistic (logit) function. Here *U* and *V* are both confounders of the relationship between the exposure and outcome, and selection depends on both *U* and *X*. We compare genetic associations with the outcome with and without adjustment for *U*.

In scenario 2, the data-generating model is:

$$G_i \sim Normal(0,1)$$
$$V_i \sim Normal(0,1)$$
$$X_i \sim Normal(0.2\,G_i + 0.4\,V_i, 1)$$
$$U_i \sim Normal(0.3\,X_i, 1)$$
$$Y_i \sim Normal(-0.5\,U_i + 0.2\,V_i, 1)$$

Participants are selected into the sample with probability $expit(-2 + U_i)$. Here *V* is a confounder of the relationship between the exposure and outcome, and *U* is a downstream effect of the exposure *X*, and hence *U* is a collider for *G* and *V*. Selection depends on *U* only. Again, we compare genetic associations with the outcome with and without adjustment for *U*.

In both cases, all normal distributions are independent. We simulate 1000 datasets for each scenario, and compare the mean estimate of the genetic association with the outcome and its empirical Type 1 error rate at a 5% significance level (that is, the proportion of 95% confidence intervals excluding the null). Around 15-20% of the 1,000,000 participants are selected in each simulated dataset.

In scenario 1, the mean association of the genetic variant with the outcome without adjustment for *U* was 0.014 (mean standard error 0.002), and the empirical Type 1 error rate was 100.0%. With adjustment for *U*, the mean association was -0.002 (mean standard error 0.002), and the empirical Type 1 error rate was 17.1%.

For peer review only - http://bmjopen.bmj.com/site/about/guidelines.xhtml

For peer review only

In scenario 2, the mean association of the genetic variant with the outcome without adjustment for *U* was -0.025 (mean standard error 0.003), and the empirical Type 1 error rate was 100.0%. With adjustment for *U*, the mean association was -0.001 (mean standard error 0.003), and the empirical Type 1 error rate was 7.8%.

In both cases, bias reduced considerably on adjustment for the factor influencing selection. In scenario 1, mean bias did not reduce to zero, as selection also depends on the exposure. We note that if selection did not additionally depend on the exposure, there would not be selection bias, as *U* is a confounder not a collider. However, even in this case with imperfect adjustment, the reduction in bias was substantial. In scenario 2, mean bias was indistinguishable from zero in the limited number of simulated datasets considered. Type 1 error rate was slightly higher than the expected 5% rate; this is likely due to the non-linear relationship between the selection factor *U* and the probability of selection.

This simulation study is a deliberately designed to be relatively simple and limited in scope, as we do not believe that any simulation study would be able to prove conclusively that covariate adjustment eliminates selection bias in all cases. Indeed, we do not make that claim; rather we claim that adjustment for the key predictors of selection is likely to ameliorate bias due to selection. There will always be intrinsic uncertainty in practice, as we do not know all the factors that influence selection, and we would be cautious to adjust for too many variables in a Mendelian randomization analysis, particularly for variables that could be mediators on the causal pathway from the exposure to the outcome, or could be colliders (common effects) of the exposure and outcome. As age and sex cannot logically be caused by the exposure, they can be safely adjusted for without risk of these variables being mediators or colliders [S1]. Hence, if age and sex are strong predictors of selection into the sample, then adjustment for age and sex should reduce selection bias in Mendelian randomization investigations.

We hope that any residual bias from selection is low, in line with previous simulation studies that have shown selection effects need to be substantial in magnitude to have non-negligible biasing effects on Mendelian randomization estimates [S2]. However, we acknowledge that this imposes an additional level of uncertainty on the results of a non-linear Mendelian randomization analysis, on top of the existing uncertainty in the Mendelian randomization paradigm due to the validity of the instrumental variable assumptions, and other factors. All methods for making causal inferences from observational data rely on untestable assumptions, and findings should be interpreted with appropriate caution.

For peer review only

SUPPLEMENTARY REFERENCES

S1. Burgess S, Davey Smith G, Davies NM, Dudbridge F, Gill D, Glymour MM, Hartwig FP, Kutalik Z, Holmes MV, Minelli C, Morrison JV. Guidelines for performing Mendelian randomization investigations: update for summer 2023. Wellcome Open Research 2023; 4:186.

S2. Gkatzionis A, Burgess S. Contextualizing selection bias in Mendelian randomization: how bad is it likely to be? International Journal of Epidemiology. 2019; 48(3):691-701.

For peer review only - http://bmjopen.bmj.com/site/about/guidelines.xhtml

Supplementary Table 1: Genetic variants and associations with BMI based on European sex-combined analysis from GIANT [23]. Beta-coefficents are in standard deviation units.

SNP	Effect allele*	Other allele	Beta-coefficient	Standard error of beta	Number of subjects	*P*-value
rs1558902	A	T	0.082	0.003	320 073	7.51E-153
rs6567160	C	T	0.056	0.004	321 958	3.93E-53
rs10938397	G	A	0.040	0.003	320 955	3.21E-38
rs543874	G	A	0.048	0.004	322 008	2.62E-35
rs2207139	G	A	0.045	0.004	322 019	4.13E-29
rs11030104	A	G	0.041	0.004	322 103	5.56E-28
rs3101336	C	T	0.033	0.003	316 872	2.66E-26
rs7138803	A	G	0.032	0.003	322 092	8.15E-24
rs10182181	G	A	0.031	0.003	321 759	8.78E-24
rs3888190	A	C	0.031	0.003	321 930	3.14E-23
rs1516725	C	T	0.045	0.005	320 644	1.89E-22
rs12446632	G	A	0.040	0.005	316 758	1.48E-18
rs2287019	C	T	0.036	0.004	300 921	4.59E-18
rs3817334	T	C	0.026	0.003	321 959	5.15E-17
rs2112347	T	G	0.026	0.003	322 019	6.19E-17
rs12566985	G	A	0.024	0.003	319 282	3.28E-15
rs3810291	A	G	0.028	0.004	296 261	4.81E-15
rs7141420	T	C	0.024	0.003	321 970	1.23E-14
rs13078960	G	T	0.030	0.004	322 135	1.74E-14
rs10968576	G	A	0.025	0.003	322 061	6.61E-14
rs17024393	C	T	0.066	0.009	297 874	7.03E-14
rs657452	A	G	0.023	0.003	313 651	5.48E-13
rs12429545	A	G	0.033	0.005	312 934	1.09E-12
rs12286929	G	A	0.022	0.003	321 903	1.31E-12
rs13107325	T	C	0.048	0.007	321 461	1.83E-12
rs11165643	T	C	0.022	0.003	320 730	2.07E-12
rs7903146	C	T	0.023	0.003	322 130	1.11E-11
rs10132280	C	A	0.023	0.003	321 797	1.14E-11
rs17405819	T	C	0.022	0.003	322 085	2.07E-11
rs1016287	T	C	0.023	0.003	321 969	2.25E-11
rs4256980	G	C	0.021	0.003	320 028	2.90E-11
rs17094222	C	T	0.025	0.004	321 770	5.94E-11
rs12401738	A	G	0.021	0.003	322 070	1.15E-10
rs7599312	G	A	0.022	0.003	322 024	1.17E-10
rs2365389	C	T	0.020	0.003	316 768	1.63E-10
rs205262	G	A	0.022	0.004	315 542	1.75E-10
rs2820292	C	A	0.020	0.003	321 707	1.83E-10
rs12885454	C	A	0.021	0.003	320 823	1.94E-10
rs16851483	T	G	0.048	0.008	233 929	3.55E-10
rs1167827	G	A	0.020	0.003	306 238	6.33E-10
rs758747	T	C	0.023	0.004	308 688	7.47E-10
rs1928295	T	C	0.019	0.003	321 979	7.91E-10
rs9925964	A	G	0.019	0.003	318 385	8.11E-10

For peer review only

rs11126666	A	G	0.021	0.003	321 979	1.33E-09
rs2650492	A	G	0.021	0.004	319 464	1.92E-09
rs6804842	G	A	0.019	0.003	321 463	2.48E-09
rs12940622	G	A	0.018	0.003	322 032	2.49E-09
rs11847697	T	C	0.049	0.008	306 243	3.99E-09
rs4740619	T	C	0.018	0.003	321 887	4.56E-09
rs13191362	A	G	0.028	0.005	321 902	7.34E-09
rs3736485	A	G	0.018	0.003	321 398	7.41E-09
rs17001654	G	C	0.031	0.005	233 722	7.76E-09
rs11191560	C	T	0.031	0.005	321 893	8.45E-09
rs1528435	T	C	0.018	0.003	321 924	1.20E-08
rs2075650	A	G	0.026	0.005	308 408	1.25E-08
rs1000940	G	A	0.019	0.003	321 836	1.28E-08
rs2033529	G	A	0.019	0.003	321 917	1.39E-08
rs11583200	C	T	0.018	0.003	322 095	1.48E-08
rs9400239	C	T	0.019	0.003	321 988	1.61E-08
rs10733682	A	G	0.017	0.003	320 727	1.83E-08
rs11688816	G	A	0.017	0.003	322 051	1.89E-08
rs11057405	G	A	0.031	0.006	314 111	2.02E-08
rs2121279	T	C	0.025	0.004	322 065	2.31E-08
rs29941	G	A	0.018	0.003	321 970	2.41E-08
rs11727676	T	C	0.036	0.006	296 401	2.55E-08
rs3849570	A	C	0.019	0.003	284 339	2.60E-08
rs6477694	C	T	0.017	0.003	322 048	2.67E-08
rs7899106	G	A	0.040	0.007	321 770	2.96E-08
rs2176598	T	C	0.020	0.004	316 848	2.97E-08
rs2245368	C	T	0.032	0.006	205 675	3.19E-08
rs17724992	A	G	0.019	0.004	319 588	3.42E-08
rs7243357	T	G	0.022	0.004	322 107	3.86E-08
rs1808579	C	T	0.017	0.003	322 032	4.17E-08

*BMI-increasing allele

For peer review only

Supplementary Table 2: Distribution of BMI (kg/m^2) in strata defined by the doubly-ranked stratification method

	HUNT			UK Biobank		
Stratum number	Mean	Lower decile	Upper decile	Mean	Lower decile	Upper decile
1	18.4	17.5	19.8	18.6	17.8	20.0
2	19.3	18.2	20.4	19.6	18.5	20.7
3	19.9	18.8	20.8	20.1	19.1	21.1
4	20.3	19.4	21.1	20.5	19.6	21.5
5	20.6	19.7	21.5	20.9	20.0	21.8
6	20.9	20.1	21.7	21.1	20.3	22.0
7	21.1	20.3	21.9	21.4	20.6	22.3
8	21.3	20.5	22.1	21.6	20.8	22.5
9	21.5	20.7	22.3	21.8	21.0	22.7
10	21.7	20.9	22.5	22.0	21.2	22.8
11	21.8	21.1	22.7	22.2	21.4	23.0
12	22.0	21.2	22.8	22.4	21.6	23.2
13	22.1	21.4	22.9	22.5	21.7	23.3
14	22.3	21.5	23.1	22.7	21.9	23.5
15	22.4	21.7	23.2	22.8	22.0	23.7
16	22.5	21.8	23.3	23.0	22.2	23.8
17	22.7	21.9	23.5	23.1	22.3	23.9
18	22.8	22.0	23.6	23.2	22.4	24.1
19	22.9	22.1	23.7	23.4	22.6	24.2
20	23.0	22.2	23.8	23.5	22.7	24.3
21	23.1	22.4	23.9	23.6	22.8	24.4
22	23.2	22.5	24.0	23.7	22.9	24.5
23	23.3	22.6	24.1	23.8	23.0	24.7
24	23.4	22.7	24.2	24.0	23.2	24.8
25	23.6	22.8	24.3	24.1	23.3	24.9
26	23.6	22.9	24.5	24.2	23.4	25.0
27	23.8	23.0	24.5	24.3	23.5	25.2
28	23.8	23.1	24.7	24.4	23.6	25.3
29	23.9	23.1	24.8	24.5	23.7	25.4
30	24.0	23.2	24.8	24.6	23.8	25.5
31	24.1	23.3	25.0	24.7	23.9	25.6
32	24.2	23.4	25.0	24.8	24.0	25.7
33	24.3	23.5	25.1	24.9	24.1	25.8
34	24.4	23.6	25.2	25.0	24.2	25.9
35	24.5	23.7	25.3	25.1	24.3	26.0
36	24.6	23.8	25.4	25.2	24.4	26.1
37	24.7	23.8	25.5	25.3	24.5	26.2
38	24.8	23.9	25.6	25.4	24.6	26.3
39	24.8	24.0	25.7	25.5	24.7	26.4
40	24.9	24.1	25.8	25.6	24.8	26.6
41	25.0	24.2	25.9	25.7	24.9	26.7
42	25.1	24.3	26.0	25.8	25.0	26.8
43	25.2	24.3	26.1	25.9	25.1	26.9
44	25.3	24.4	26.2	26.0	25.1	27.0
45	25.4	24.5	26.3	26.1	25.3	27.1

For peer review only

For peer review only - http://bmjopen.bmj.com/site/about/guidelines.xhtml

46	25.5	24.6	26.4	26.2	25.4	27.2
47	25.6	24.7	26.4	26.3	25.4	27.3
48	25.6	24.8	26.6	26.5	25.5	27.4
49	25.7	24.8	26.7	26.6	25.6	27.5
50	25.8	25.0	26.8	26.7	25.7	27.6
51	25.9	25.0	26.9	26.8	25.8	27.7
52	26.0	25.1	26.9	26.9	25.9	27.8
53	26.1	25.2	27.0	27.0	26.0	28.0
54	26.2	25.3	27.1	27.1	26.1	28.1
55	26.3	25.4	27.2	27.2	26.2	28.2
56	26.4	25.5	27.3	27.3	26.3	28.3
57	26.5	25.5	27.4	27.4	26.4	28.4
58	26.6	25.6	27.5	27.5	26.5	28.6
59	26.7	25.7	27.6	27.6	26.6	28.7
60	26.8	25.8	27.7	27.7	26.7	28.8
61	26.9	25.9	27.9	27.9	26.8	28.9
62	27.0	26.0	27.9	28.0	26.9	29.1
63	27.1	26.1	28.1	28.1	27.0	29.2
64	27.2	26.2	28.2	28.2	27.1	29.3
65	27.3	26.3	28.3	28.3	27.2	29.5
66	27.4	26.4	28.4	28.5	27.3	29.6
67	27.5	26.5	28.6	28.6	27.5	29.8
68	27.6	26.6	28.7	28.7	27.6	29.9
69	27.7	26.7	28.8	28.9	27.7	30.0
70	27.9	26.8	28.9	29.0	27.8	30.2
71	28.0	26.9	29.0	29.1	28.0	30.4
72	28.1	27.0	29.1	29.3	28.1	30.5
73	28.2	27.1	29.4	29.4	28.2	30.7
74	28.3	27.2	29.5	29.6	28.4	30.9
75	28.5	27.4	29.6	29.8	28.5	31.1
76	28.6	27.5	29.8	29.9	28.7	31.2
77	28.7	27.6	29.9	30.1	28.8	31.4
78	28.9	27.8	30.1	30.3	28.9	31.6
79	29.0	27.9	30.3	30.4	29.1	31.8
80	29.2	28.0	30.5	30.6	29.3	32.1
81	29.4	28.2	30.7	30.8	29.4	32.3
82	29.5	28.3	30.9	31.0	29.6	32.6
83	29.7	28.4	31.1	31.3	29.8	32.8
84	29.9	28.6	31.3	31.5	30.0	33.0
85	30.1	28.8	31.5	31.7	30.2	33.3
86	30.3	28.9	31.8	32.0	30.4	33.7
87	30.6	29.1	32.0	32.3	30.6	34.0
88	30.8	29.3	32.3	32.5	30.9	34.4
89	31.0	29.5	32.6	32.9	31.1	34.7
90	31.3	29.7	33.0	33.2	31.4	35.2
91	31.6	30.0	33.3	33.6	31.7	35.6
92	32.0	30.3	33.7	34.0	32.0	36.2
93	32.3	30.6	34.2	34.5	32.4	36.7
94	32.8	30.9	34.8	35.0	32.8	37.4
95	33.3	31.4	35.5	35.7	33.3	38.2
96	33.9	31.8	36.1	36.4	33.9	39.2

For peer review only - http://bmjopen.bmj.com/site/about/guidelines.xhtml

97	34.7	32.4	37.1	37.4	34.6	40.5
98	35.8	33.3	38.7	38.7	35.5	42.2
99	37.3	34.1	40.5	40.5	36.7	44.8
100	40.3	35.6	43.2	44.3	38.8	47.9

We report the mean, lower decile (10th percentile of the distribution), and upper decile (90th percentile of the distribution) of the BMI distribution for participants in each stratum created by the doubly-ranked method. In the first and 100th strata, we instead report the 20th and 80th percentiles of the distribution.

For peer review only

Supplementary Figure 1: Associations between the genetic score and BMI in strata defined by the doubly-ranked method plotted against mean BMI in each stratum for: A) HUNT, B) UK Biobank. Error bars represent 95% confidence intervals.

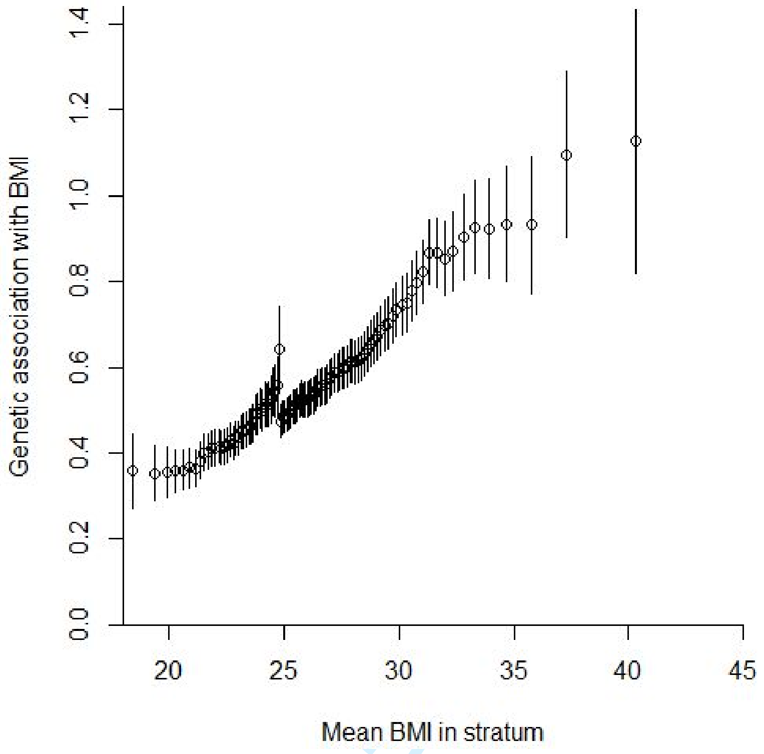

A.

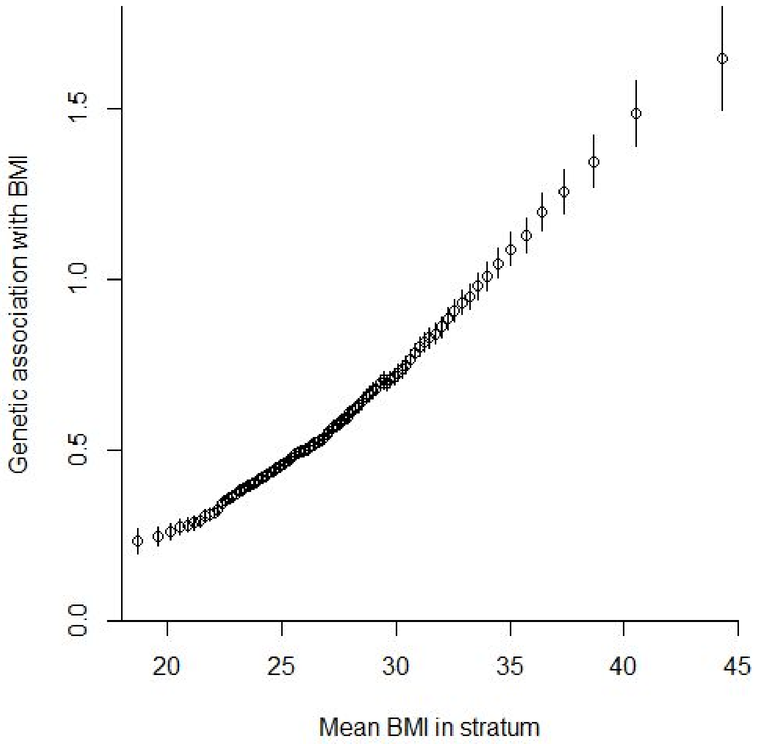

B.

For peer review only - http://bmjopen.bmj.com/site/about/guidelines.xhtml

Supplementary Figure 2: Genetic associations with potential competing risk factors in strata of the HUNT study population

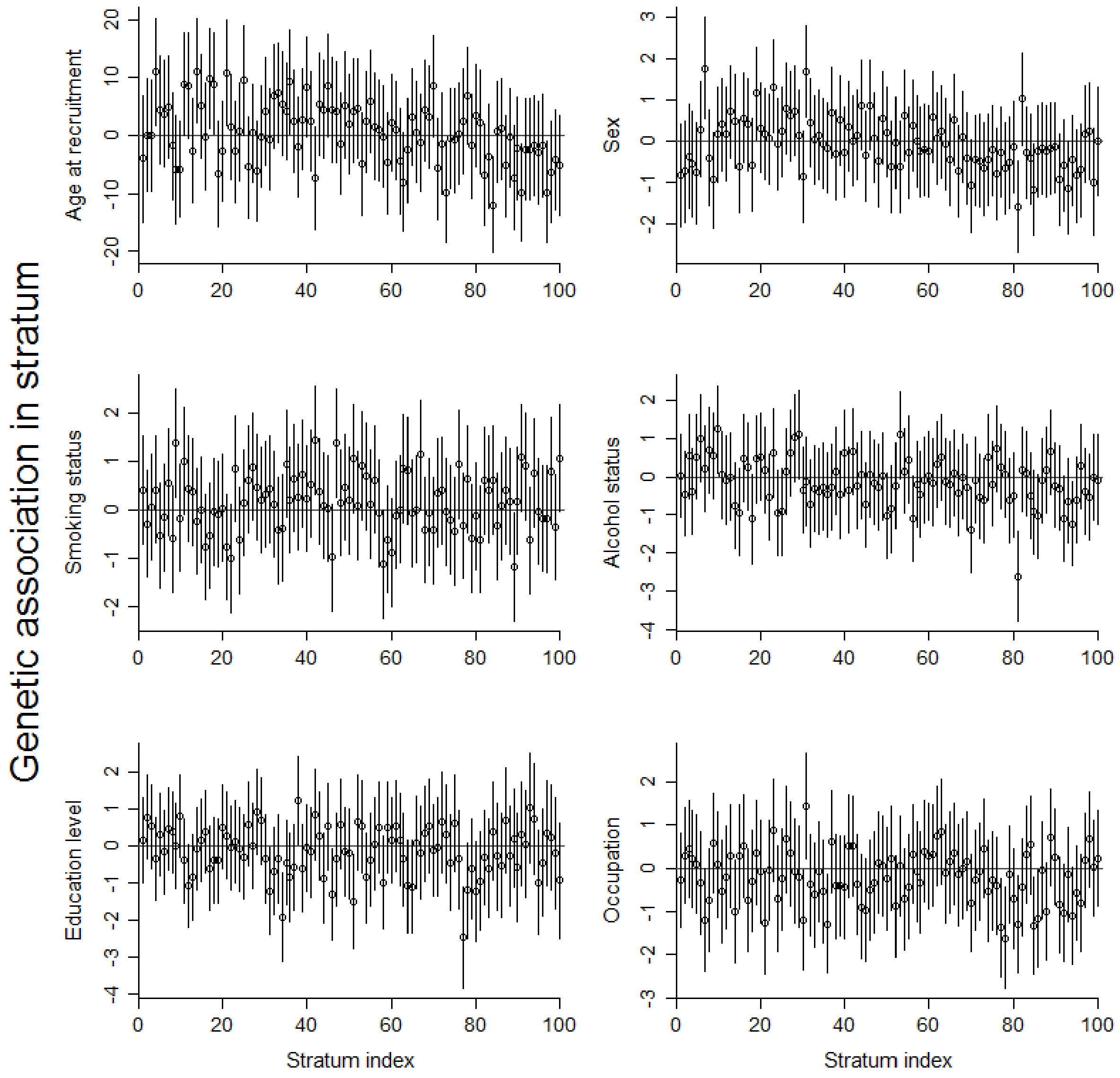

Points represent association estimates from linear (age at recruitment, years) or logistic regression (all other traits). Error bars represent 95% confidence intervals. Stratum 1 has lowest average BMI levels, stratum 100 has highest average BMI levels.

Supplementary Figure 3: Localized average causal effect estimates (stratum-specific estimates) for all-cause mortality plotted against mean BMI in each stratum. Estimates represent hazard ratios per 1 kg/m^2 increase in genetically-predicted BMI. Error bars represent 95% confidence intervals.

A) HUNT, doubly-ranked method:

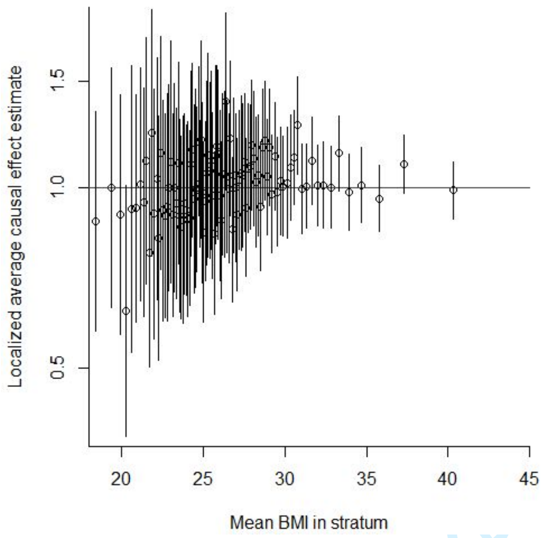

B) UK Biobank, doubly-ranked method:

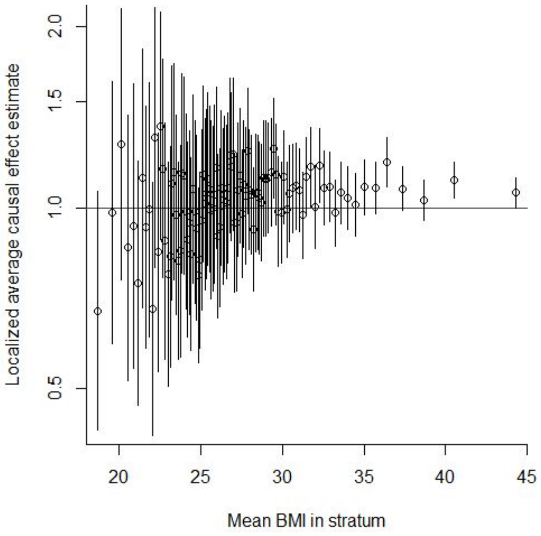

C) HUNT, residual method:

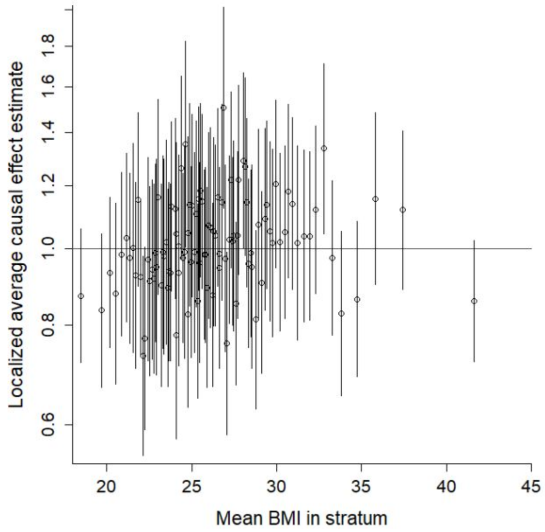

D) UK Biobank, residual method:

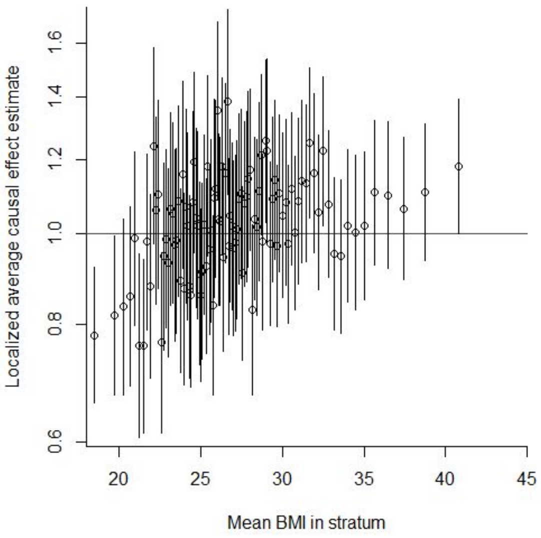

This presentation does not smooth over stratum-specific estimates, hence providing a representation of the causal effect of BMI which is not dependent on the choice of fractional polynomial smoothing. However, stratum-specific estimates are imprecise and variable, as they each only use 1% of the overall sample size.

Supplementary Figure 4: Non-linear Mendelian randomization – dose-response curve between body mass index and all-cause mortality using the doubly-ranked method with inverse-normal rank transformed BMI values in UK Biobank. Grey lines represent 95% confidence intervals. The reference value for BMI was taken as the median of the BMI distribution at 26.7 kg/m^2.

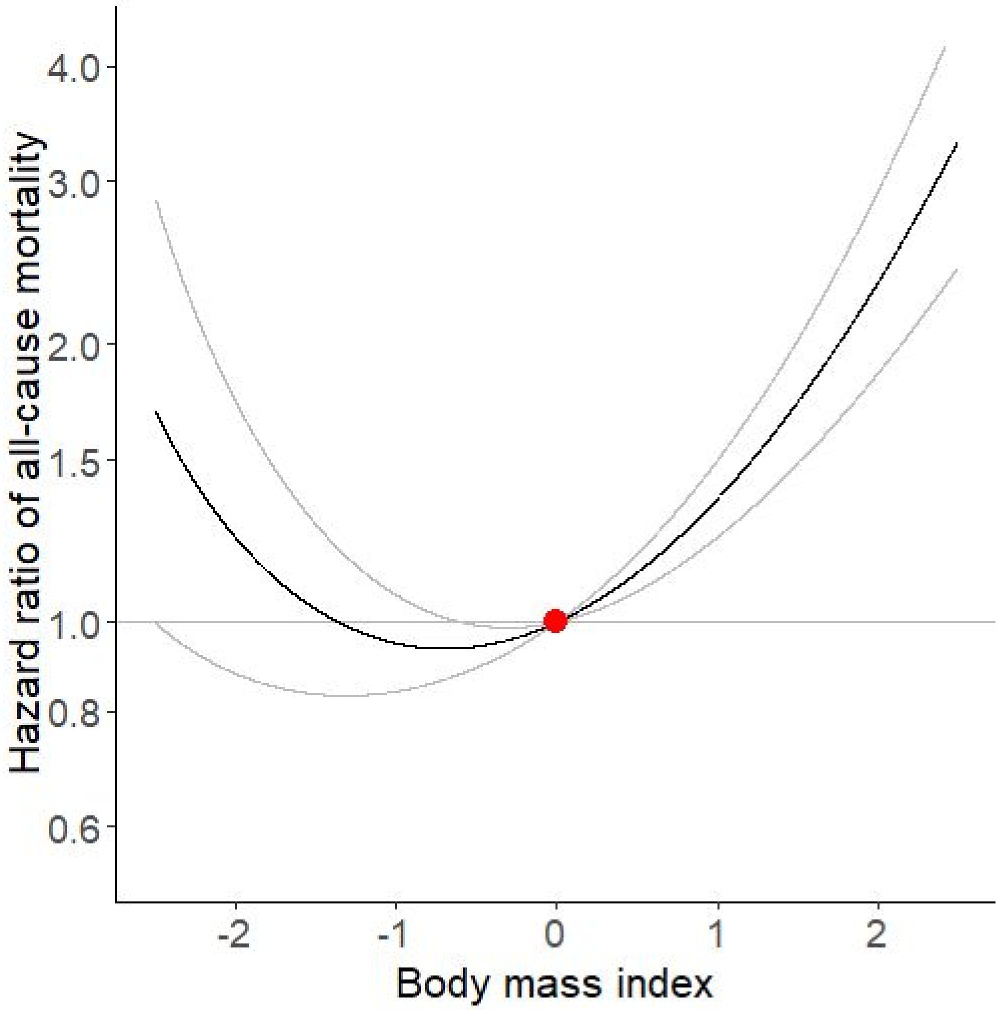

The labels on the horizontal axis represent the number of standard deviations below or above the median of the BMI distribution: for example, -1 represents one standard deviation below the median, and +2 represents two standard deviations above the median.

The reference values are:
-2 = 19.9 kg/m2
-1 = 23.0 kg/m2
0 = 26.7 kg/m2
+1 = 31.6 kg/m2
+2 = 39.2 kg/m2

For peer review only - http://bmjopen.bmj.com/site/about/guidelines.xhtml

Supplementary Figure 5: Localized average causal effect estimates (stratum-specific estimates) for all-cause mortality plotted against mean BMI in each stratum estimated in men and women separately. Estimates represent hazard ratios per 1 kg/m^2 increase in genetically-predicted BMI. Error bars represent 95% confidence intervals.

A) HUNT, men only:

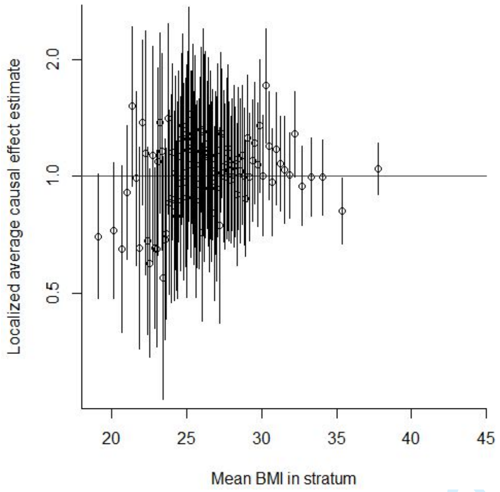

B) HUNT, women only:

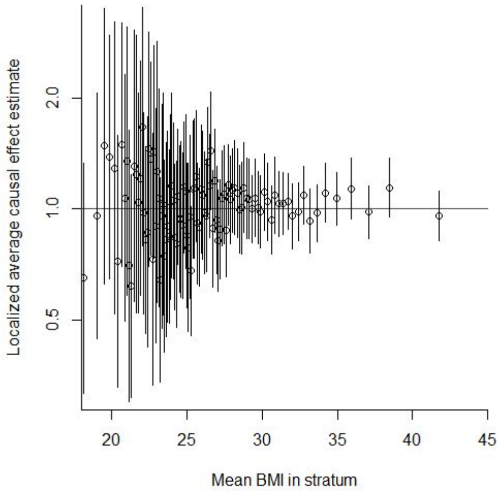

C) UK Biobank, men only:

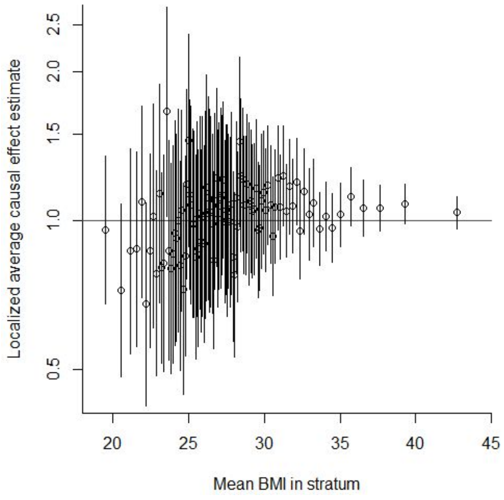

D) UK Biobank, women only:

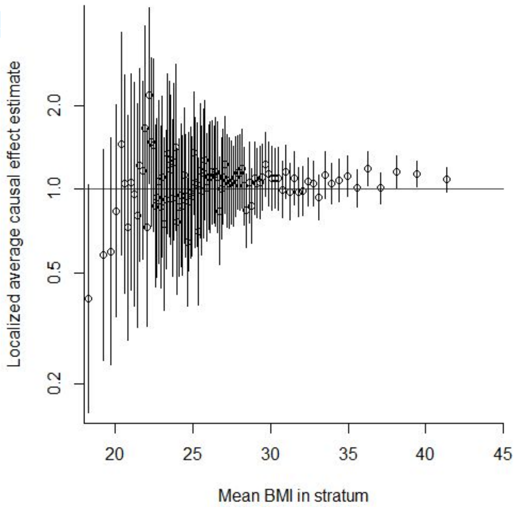

For peer review only - http://bmjopen.bmj.com/site/about/guidelines.xhtml

Supplementary Figure 6: Localized average causal effect estimates (stratum-specific estimates) for cause-specific mortality plotted against mean BMI in each stratum. Estimates represent hazard ratios per 1 kg/m^2 increase in genetically-predicted BMI. Error bars represent 95% confidence intervals.

A: Cardiovascular mortality

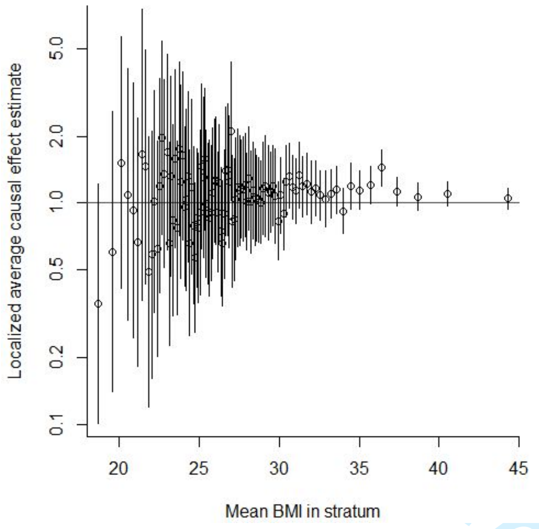

B: Cancer mortality

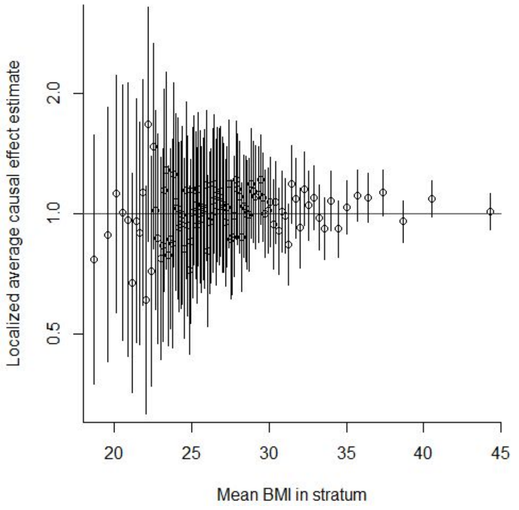

C: Other (non-cardiovascular, non-cancer) mortality

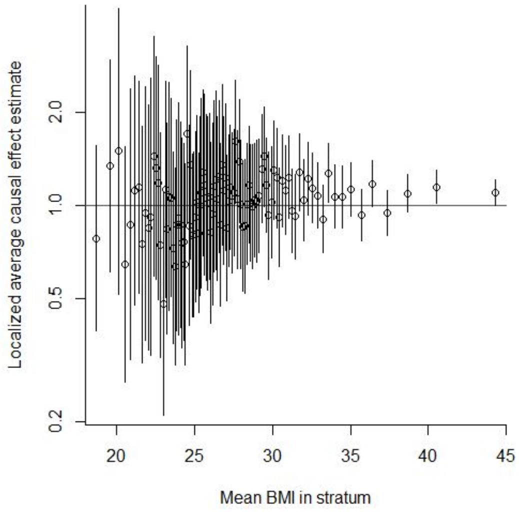

For peer review only - http://bmjopen.bmj.com/site/about/guidelines.xhtml

STROBE-MR checklist of recommended items to address in reports of Mendelian randomization studies[1 2]

Item No.	Section	Checklist item	Page No.	Relevant text from manuscript
1	**TITLE and ABSTRACT**	Indicate Mendelian randomization (MR) as the study's design in the title and/or the abstract if that is a main purpose of the study	1-2	Title: "Body mass index and all-cause mortality in HUNT and UK Biobank studies: revised non-linear Mendelian randomization analyses"<br>Abstract: "**Objectives**: To estimate the shape of the causal relationship between body mass index (BMI) and mortality risk in a Mendelian randomization framework."
	**INTRODUCTION**			
2	**Background**	Explain the scientific background and rationale for the reported study. What is the exposure? Is a potential causal relationship between exposure and outcome plausible? Justify why MR is a helpful method to address the study question	2,4	Abstract: "To estimate the shape of the causal relationship between body mass index (BMI) and mortality risk in a Mendelian randomization framework."<br>Introduction: "The epidemiological relationship between BMI and mortality is complex, with many observational studies conducted in Western countries showing a J-shaped relationship between BMI and mortality risk."<br>"However, such findings may not reflect the causal relationship between BMI and mortality, as observation association are influenced by confounding and reverse causation."
3	**Objectives**	State specific objectives clearly, including pre-specified causal hypotheses (if any). State that MR is a method that, under specific assumptions, intends to estimate causal effects	2,4	Abstract: "To estimate the shape of the causal relationship between body mass index (BMI) and mortality risk in a Mendelian randomization framework."<br>Introduction: "We first investigate whether the constant genetic effect assumption holds for this example, and explore the validity of the instrumental variable assumptions in strata of the population. We then present non-linear Mendelian randomization findings from the doubly-ranked stratification method."<br>"Hence, associations between genetic predictors of BMI and mortality provide insights into the causal effect of BMI on mortality."

For peer review only - http://bmjopen.bmj.com/site/about/guidelines.xhtml

	**METHODS**				
4	**Study design and data sources**		Present key elements of the study design early in the article. Consider including a table listing sources of data for all phases of the study. For each data source contributing to the analysis, describe the following:		
		a)	Setting: Describe the study design and the underlying population, if possible. Describe the setting, locations, and relevant dates, including periods of recruitment, exposure, follow-up, and data collection, when available.	6	See sections "The HUNT study" and "The UK Biobank study" (some of this detail is in reference 19, as indicated: "We present abbreviated descriptions of the datasets included in the analysis; detailed descriptions are in the original paper [19]").
		b)	Participants: Give the eligibility criteria, and the sources and methods of selection of participants. Report the sample size, and whether any power or sample size calculations were carried out prior to the main analysis	6	See sections "The HUNT study" and "The UK Biobank study" (detail is in reference 19: "We present abbreviated descriptions of the datasets included in the analysis; detailed descriptions are in the original paper [19]").
		c)	Describe measurement, quality control and selection of genetic variants	6	See section "SNPs and genetic score used as instrumental variables"
		d)	For each exposure, outcome, and other relevant variables, describe methods of assessment and diagnostic criteria for diseases	NA	See reference 19 for detail.
		e)	Provide details of ethics committee approval and participant informed consent, if relevant	7	See section "Ethical Approval" and "Patient Involvement"
5	**Assumptions**		Explicitly state the three core IV assumptions for the main analysis (relevance, independence and exclusion restriction) as well assumptions for any additional or sensitivity analysis	4,6	Introduction: "According to Mendel's laws of inheritance, genetic variants should be uncorrelated with traits that they do not affect, and hence should be independent of potential confounders"<br>Methods: "We assessed the instrumental variable assumptions by estimating associations between the genetic score and various traits that are competing risk factors in UK Biobank: smoking status (ever versus never), alcohol status (current versus other), education level (post-secondary or higher versus other), occupation (currently employed versus other), age at recruitment, and sex."
6	**Statistical methods: main analysis**		Describe statistical methods and statistics used	6-7	See Section "Statistical analyses".

For peer review only - http://bmjopen.bmj.com/site/about/guidelines.xhtml

		a)	Describe how quantitative variables were handled in the analyses (i.e., scale, units, model)	7	See Section "Statistical analyses".
		b)	Describe how genetic variants were handled in the analyses and, if applicable, how their weights were selected	6	See Section "SNPs and genetic score used as instrumental variables"
		c)	Describe the MR estimator (e.g. two-stage least squares, Wald ratio) and related statistics. Detail the included covariates and, in case of two-sample MR, whether the same covariate set was used for adjustment in the two samples	7	See Section "Statistical analyses":<br>"We then calculated the linear Mendelian randomization estimate, referred to as a localized average causal effect (LACE), in each stratum of the population as a ratio of coefficients: the association of the genetic score with the outcome divided by the associations of the genetic score with the exposure. associations with the exposure (BMI) were obtained from linear regression; associations with the outcome (mortality) were obtained from Cox proportional hazards regression, using age as the timescale. All associations were adjusted for age, age-squared, sex, and centre (for UK Biobank)."
		d)	Explain how missing data were addressed	6	"We excluded participants without data on BMI or genetic variants for BMI, leaving 56,150 individuals for analysis."
		e)	If applicable, indicate how multiple testing was addressed	N/A	We did not account for multiple testing, as the presentation of p-values is not a major aspect of the manuscript.
7	**Assessment of assumptions**		Describe any methods or prior knowledge used to assess the assumptions or justify their validity	6	See Section "SNPs and genetic score used as instrumental variables":<br>"We assessed the instrumental variable assumptions by estimating associations between the genetic score and various traits that are competing risk factors in UK Biobank: smoking status (ever versus never), alcohol status (current versus other), education level (post-secondary versus other), occupations (currently employed versus other), age at recruitment, and sex.
8	**Sensitivity analyses and additional analyses**		Describe any sensitivity analyses or additional analyses performed (e.g. comparison of effect estimates from different approaches, independent replication, bias analytic techniques, validation of instruments, simulations)	7	Sensitivity Analyses: "In brief, first we divided the sample into 100 strata using the doubly-ranked method. For comparison, we also present results from stratification using the residual method for the primary outcome of all-cause mortality."<br>Additional analyses: Testing for linearity:

For peer review only - http://bmjopen.bmj.com/site/about/guidelines.xhtml

					"We report two tests for non-linearity: a linearity test, which assesses whether a non-linear model fits the LACE estimates better than a linear model, and a trend test, which tests for a linear trend amongst the LACE estimates. "
9	**Software and pre-registration**				
		a)	Name statistical software and package(s), including version and settings used	7	"All statistical analyses were performed in R (version 4.3.1), and non-linear Mendelian randomisation analyses were performed using the SUMnlmr package [25]."
		b)	State whether the study protocol and details were pre-registered (as well as when and where)	N/A	The study protocol was not pre-registered.
	**RESULTS**				
10	**Descriptive data**				
		a)	Report the numbers of individuals at each stage of included studies and reasons for exclusion. Consider use of a flow diagram		All numbers provided in Table 1.
		b)	Report summary statistics for phenotypic exposure(s), outcome(s), and other relevant variables (e.g. means, SDs, proportions)		All numbers provided in Table 1.
		c)	If the data sources include meta-analyses of previous studies, provide the assessments of heterogeneity across these studies		N/A – we present results from the HUNT and UK Biobank studies separately, and do not attempt to meta-analyse or otherwise combine results.
		d)	For two-sample MR:<br>i. Provide justification of the similarity of the genetic variant-exposure associations between the exposure and outcome samples<br>ii. Provide information on the number of individuals who overlap between the exposure and outcome studies		N/A – not two-sample MR.
11	**Main results**				
		a)	Report the associations between genetic variant and exposure, and between genetic variant and outcome, preferably on an interpretable scale		Genetic associations with exposure are shown in Supplementary Figure 1.<br>Scaled genetic association with outcome are shown in Supplementary Figures 3, 5, and 6.
		b)	Report MR estimates of the relationship between exposure and outcome, and the measures of uncertainty from the MR analysis, on an interpretable scale, such as odds ratio or relative risk per SD difference		MR estimates are shown directly in Supplementary Figures 3, 5, and 6. Smoothed versions of these associations are shown in Figures 2, 3, and 4.

For peer review only - http://bmjopen.bmj.com/site/about/guidelines.xhtml

		c)	If relevant, consider translating estimates of relative risk into absolute risk for a meaningful time period		N/A
		d)	Consider plots to visualize results (e.g. forest plot, scatterplot of associations between genetic variants and outcome versus between genetic variants and exposure)		N/A
12	**Assessment of assumptions**				
		a)	Report the assessment of the validity of the assumptions	8	See section "Assessment of instrument validity"
		b)	Report any additional statistics (e.g., assessments of heterogeneity across genetic variants, such as I^2, Q statistic or E-value)	6	F statistics are provided in the Methods section under "SNPs and genetic score used as instrumental variables.<br>Nonlinear tests are reported in Figures 2, 3, and 4.
13	**Sensitivity analyses and additional analyses**				
		a)	Report any sensitivity analyses to assess the robustness of the main results to violations of the assumptions		See Figure 2: residual versus ranked stratification.
		b)	Report results from other sensitivity analyses or additional analyses		Figure 2: residual versus ranked stratification<br>Figure 3: results stratified by sex<br>Figure 4: exploring different cause-specific mortality subtypes.
		c)	Report any assessment of direction of causal relationship (e.g., bidirectional MR)		N/A, directionality not considered.
		d)	When relevant, report and compare with estimates from non-MR analyses	11	In Discussion, paragraph beginning "Previous investigations into the non-linear shape of the causal relationship between BMI and mortality have used a variety of approaches." and in particular "Carslake *et al* performed an instrumental variable analysis to investigate the impact of BMI on mortality in the HUNT study, but using offspring BMI as an instrument for the BMI".
		e)	Consider additional plots to visualize results (e.g., leave-one-out analyses)		N/A
	**DISCUSSION**				
14	**Key results**		Summarize key results with reference to study objectives	10	Results are summarized in the Discussion section:

For peer review only - http://bmjopen.bmj.com/site/about/guidelines.xhtml

					"While the curves generally display a J-shaped relationship, evidence for a harmful average effect of decreased BMI on mortality at low BMI levels was limited. Evidence supporting non-linearity from the doubly-ranked method was unconvincing for all mortality outcomes."<br>Also see Abstract / Results.
15	**Limitations**		Discuss limitations of the study, taking into account the validity of the IV assumptions, other sources of potential bias, and imprecision. Discuss both direction and magnitude of any potential bias and any efforts to address them	11-12	Limitations are clearly discussed in the Discussion section, paragraph beginning "However, there are important limitations…" Several potential limitations are discussed, including validity of instruments, pleiotropy, bias due to selection bias, representativeness, and reverse causation.
16	**Interpretation**				
		a)	Meaning: Give a cautious overall interpretation of results in the context of their limitations and in comparison with other studies	10	Interpretation of results is provided in the Discussion section: "In conclusion, non-linear Mendelian randomization analyses using the doubly-ranked stratification method provide strong evidence for harmful effects of increased BMI on mortality above 25 kg/m2. Evidence for a harmful effect of low BMI was only present in some analyses, and where present, only below 20 kg/m2.".<br>Also see Abstract/Conclusion.
		b)	Mechanism: Discuss underlying biological mechanisms that could drive a potential causal relationship between the investigated exposure and the outcome, and whether the gene-environment equivalence assumption is reasonable. Use causal language carefully, clarifying that IV estimates may provide causal effects only under certain assumptions		N/A (mechanisms linking BMI and mortality have been discussed elsewhere at length, and evidence for specific mechanisms is not provided here).
		c)	Clinical relevance: Discuss whether the results have clinical or public policy relevance, and to what extent they inform effect sizes of possible interventions	10	Discussion: "Our findings challenge the "obesity paradox" consensus from observational epidemiology that increases in BMI might reduce risk of mortality for a substantial proportion of the population."
17	**Generalizability**		Discuss the generalizability of the study results (a) to other populations, (b) across other exposure periods/timings, and (c) across other levels of exposure	11	Discussion / Limitations: "Our findings may therefore not be applicable to other populations. "

	**OTHER INFORMATION**			
18	**Funding**	Describe sources of funding and the role of funders in the present study and, if applicable, sources of funding for the databases and original study or studies on which the present study is based	1	See "Funding statement"
19	**Data and data sharing**	Provide the data used to perform all analyses or report where and how the data can be accessed, and reference these sources in the article. Provide the statistical code needed to reproduce the results in the article, or report whether the code is publicly accessible and if so, where	31	See "Data sharing statement"
20	**Conflicts of Interest**	All authors should declare all potential conflicts of interest	1	Provided under "Competing interest statement"

This checklist is copyrighted by the Equator Network under the Creative Commons Attribution 3.0 Unported (CC BY 3.0) license.

1. Skrivankova VW, Richmond RC, Woolf BAR, Yarmolinsky J, Davies NM, Swanson SA, et al. Strengthening the Reporting of Observational Studies in Epidemiology using Mendelian Randomization (STROBE-MR) Statement. JAMA. 2021;under review.

2. Skrivankova VW, Richmond RC, Woolf BAR, Davies NM, Swanson SA, VanderWeele TJ, et al. Strengthening the Reporting of Observational Studies in Epidemiology using Mendelian Randomisation (STROBE-MR): Explanation and Elaboration. BMJ. 2021;375:n2233.

For peer review only

Body mass index and all-cause mortality in HUNT and UK Biobank studies: revised non-linear Mendelian randomization analyses

Journal:	*BMJ Open*
Manuscript ID	bmjopen-2023-081399.R1
Article Type:	Original research
Date Submitted by the Author:	07-Feb-2024
Complete List of Authors:	Burgess, Stephen; University of Cambridge, MRC Biostatistics Unit; University of Cambridge, Cardiovascular Epidemiology Unit, Department of Public Health and Primary Care<br>Sun, Yi-Qian; Norges teknisk-naturvitenskapelige universitet, Department of Clinical and Molecular Medicine (IKOM)<br>Zhou, Ang; University of Cambridge, MRC Biostatistics Unit; University of South Australia, Australian Centre for Precision Health<br>Buck, Christopher; University of Cambridge, MRC Biostatistics Unit<br>Mason, Amy M; University of Cambridge, Department of Public Health and Primary Care<br>Mai, Xiao-Mei; Norwegian University of Science and Technology, Department of Public Health and Nursing
**Primary Subject Heading**:	Public health
Secondary Subject Heading:	Epidemiology, Genetics and genomics, Public health
Keywords:	Obesity, Observational Study, GENETICS, EPIDEMIOLOGY

SCHOLARONE™
Manuscripts

I, the Submitting Author has the right to grant and does grant on behalf of all authors of the Work (as defined in the below author licence), an exclusive licence and/or a non-exclusive licence for contributions from authors who are: i) UK Crown employees; ii) where BMJ has agreed a CC-BY licence shall apply, and/or iii) in accordance with the terms applicable for US Federal Government officers or employees acting as part of their official duties; on a worldwide, perpetual, irrevocable, royalty-free basis to BMJ Publishing Group Ltd ("BMJ") its licensees and where the relevant Journal is co-owned by BMJ to the co-owners of the Journal, to publish the Work in this journal and any other BMJ products and to exploit all rights, as set out in our licence.

The Submitting Author accepts and understands that any supply made under these terms is made by BMJ to the Submitting Author unless you are acting as an employee on behalf of your employer or a postgraduate student of an affiliated institution which is paying any applicable article publishing charge ("APC") for Open Access articles. Where the Submitting Author wishes to make the Work available on an Open Access basis (and intends to pay the relevant APC), the terms of reuse of such Open Access shall be governed by a Creative Commons licence – details of these licences and which Creative Commons licence will apply to this Work are set out in our licence referred to above.

Other than as permitted in any relevant BMJ Author's Self Archiving Policies, I confirm this Work has not been accepted for publication elsewhere, is not being considered for publication elsewhere and does not duplicate material already published. I confirm all authors consent to publication of this Work and authorise the granting of this licence.

For peer review only

Body mass index and all-cause mortality in HUNT and UK Biobank studies: revised non-linear Mendelian randomization analyses

Stephen Burgess[1,2,3], Yi-Qian Sun[4,5,6], Ang Zhou[1,7], Christopher Buck[1], Amy M Mason[2,3], Xiao-Mei Mai[8]

1. Medical Research Council Biostatistics Unit, University of Cambridge, Cambridge, UK.
2. Cardiovascular Epidemiology Unit, Department of Public Health and Primary Care, University of Cambridge, Cambridge, UK.
3. Victor Phillip Dahdaleh Heart and Lung Research Institute, University of Cambridge, Cambridge, UK.
4. Department of Clinical and Molecular Medicine, Faculty of Medicine and Health Science, Norwegian University of Science and Technology, Trondheim, Norway.
5. Department of Pathology, Clinic of Laboratory Medicine, St. Olavs Hospital, Trondheim University Hospital, Trondheim, Norway.
6. Center for Oral Health Services and Research Mid-Norway (TkMidt), Trondheim, Norway.
7. Australian Centre for Precision Health, Unit of Clinical and Health Sciences, University of South Australia, Adelaide, Australia.
8. Department of Public Health and Nursing, Faculty of Medicine and Health Science, Norwegian University of Science and Technology, Trondheim, Norway.

Corresponding author: Stephen Burgess (sb452@medschl.cam.ac.uk)

Word count: 299 (abstract), 4357 (main text).

Key words: Obesity paradox, reverse causation, genetic epidemiology, causal inference, doubly-ranked stratification.

For peer review only - http://bmjopen.bmj.com/site/about/guidelines.xhtml

ABSTRACT

Objectives: To estimate the shape of the causal relationship between body mass index (BMI) and mortality risk in a Mendelian randomization framework.

Design: Mendelian randomization analyses of two prospective population-based cohorts.

Setting: Individuals of European ancestries living in Norway or the United Kingdom (UK).

Participants: 56,150 participants from the Trøndelag Health Study (HUNT) in Norway and 366,385 participants from UK Biobank recruited by postal invitation.

Outcomes: All-cause mortality and cause-specific mortality (cardiovascular, cancer, non-cardiovascular non-cancer).

Results: A previously published non-linear Mendelian randomization analysis of these data using the residual stratification method suggested a J-shaped association between genetically-predicted BMI and mortality outcomes with the lowest mortality risk at a BMI of around 25 kg/m^2. However, the "constant genetic effect" assumption required by this method is violated. The re-analysis of these data using the more reliable doubly-ranked stratification method still indicated a potential J-shaped relationship, but with less certainty as there was less precision in estimates at the lower end of the BMI distribution. Evidence for a harmful effect of reducing BMI at low BMI levels was only present in some analyses, and where present, only below 20 kg/m^2. A harmful effect of increasing BMI for all-cause mortality was evident above 25 kg/m^2, for cardiovascular mortality above 24 kg/m^2, for cancer mortality above 30 kg/m^2, and for non-cardiovascular non-cancer mortality above 26 kg/m^2. In UK Biobank, the association between genetically-predicted BMI and mortality at high BMI levels was stronger in women than in men.

Conclusion: This research challenges findings from previous conventional observational epidemiology and Mendelian randomization investigations that the lowest level of mortality risk is at a BMI level of around 25 kg/m^2. Our results provide evidence that reductions in BMI will only increase mortality risk for a small proportion of the population, and increases in BMI will increase mortality risk for those with BMI above 25 kg/m^2.

For peer review only

For peer review only - http://bmjopen.bmj.com/site/about/guidelines.xhtml

Strengths and limitations of the study

- Mendelian randomization design minimizes bias due to confounding and reverse causation
- Large sample sizes enable powerful analyses even in low body mass index (BMI) individuals
- Validity of the genetic variants as instrumental variables cannot be verified
- Bias due to selection could be non-negligible and could vary across strata
- All estimates are averaged across a stratum of the population; individual effects of raising or lowering BMI may vary between individuals

For peer review only

For peer review only - http://bmjopen.bmj.com/site/about/guidelines.xhtml

INTRODUCTION

Body mass index (BMI) is a convenient and accessible measure of obesity. The epidemiological relationship between BMI and mortality is complex, with many observational studies conducted in Western countries showing a J-shaped relationship between BMI and mortality risk in the general population, such that mortality risk is lowest for those in the upper normal weight (BMI 20.0-24.9)(1-3) or even the overweight (BMI 25.0-29.9)(4-6) category rather than the lower normal weight (BMI 18.5-19.9) or underweight category (BMI <18.5). However, such findings may not reflect the causal relationship between BMI and mortality, as observational associations are influenced by confounding and reverse causation.

Mendelian randomization is an epidemiological technique for the analysis of observational data designed to avoid bias due to confounding and reverse causation(7). Rather than assessing associations between BMI and mortality directly, we assess associations between genetic variants that predict BMI levels and mortality risk(8). This is analogous to the analysis of a randomized controlled trial for weight loss(9), which would assess associations between the randomized intervention and the outcome, rather than associations with measured BMI in trial participants. According to Mendel's laws of inheritance, genetic variants should be uncorrelated with traits that they do not affect, and hence should be independent of potential confounders. As genetic variants are determined at conception, genetic associations should be protected from reverse causation(10). These properties mean that genetic variants are plausible instrumental variables; an instrumental variable is a variable that behaves as if it has been randomized in the population(11). Hence, associations between genetic predictors of BMI and mortality provide insights into the causal effect of BMI on mortality.

Non-linear Mendelian randomization is an extension of standard Mendelian randomization to investigate the shape of the causal relationship between an exposure and an outcome(12). A typical randomized trial estimates an average causal effect, representing the average impact of a population-wide shift in the distribution of an exposure; similarly, a standard Mendelian randomization analysis estimates a population-averaged causal effect(13). A widely used method for non-linear Mendelian randomization stratifies the population based on levels of the exposure, and estimates 'localized average causal effects', representing the average impact of a population-wide shift in the distribution of the exposure for that stratum of the population(13).

However, stratifying the population such that the instrumental variable assumptions still hold in the strata of the population is tricky. Stratifying on the exposure directly would break randomization, as exposure levels are influenced by the instrumental variables. Hence an individual in the lowest stratum of the population defined by the exposure with a genetic predisposition to high values of the exposure would be more likely to have low values of the confounders. This is an example of collider bias(14): the exposure is a common effect of the instrument and exposure—outcome confounders, and so stratification on the exposure leads to a conditional association between the instrument and confounders. The initial proposal for non-linear Mendelian randomization was to stratify on the residual exposure, defined as the residual from regression of the exposure on the instrumental variables(13). The residual exposure is independent of the instrumental variables by construction, and the instrumental variables will be independent of confounders within strata of the residual exposure under a "constant genetic effect" assumption(15). However, if the effect of the genetic instrumental variables on the exposure varies between individuals, then this residual stratification method can lead to severe bias in stratum-specific estimates(16, 17).

For peer review only - http://bmjopen.bmj.com/site/about/guidelines.xhtml

An alternative method, known as the doubly-ranked method, allows the instrumental variable effects on the exposure to vary between individuals provided that a weaker “rank-preserving assumption” holds(18). The rank-preserving assumption states that the ranking of individuals according to their exposure levels would be the same at all levels of the instrumental variable. That is, if the genotype of all individuals were set to any fixed value, the order of individuals according to their exposure levels would be the same. The doubly-ranked method is implemented by first ranking individuals according to their level of the instrument to form pre-strata with similar values of the instrument, and then ranking individuals within each pre-stratum according to their level of the exposure, so that the lowest stratum contains the individual with the lowest exposure value from pre-stratum 1, the individual with the lowest exposure value from pre-stratum 2, the individual with the lowest exposure value from pre-stratum 3, and so on. This method has been shown to be more reliable than the residual method in simulation studies(17, 18).

In a previous paper, we presented evidence for the effect of BMI on mortality from non-linear Mendelian randomization using the residual stratification method in the Norwegian Trøndelag Health (HUNT) and United Kingdom (UK) Biobank studies. A J-shaped association between genetically-predicted BMI and mortality was observed in each study population overall, although a monotonic increasing association (that is, an always-increasing association) was seen in never-smokers(19). Here, we present a re-analysis of the same datasets using the doubly-ranked stratification method. We first investigate whether the constant genetic effect assumption holds for this example, and explore the validity of the instrumental variable assumptions in strata of the population. We then present non-linear Mendelian randomization findings from the doubly-ranked stratification method.

For peer review only - http://bmjopen.bmj.com/site/about/guidelines.xhtml

METHODS

We present abbreviated descriptions of the datasets included in the analysis; detailed descriptions are in the original paper(19).

The HUNT study

We used data from the second wave (1995-1997) of the HUNT study on 65,229 individuals living in the northern part of Trøndelag and over the age of 20 years(20). Participants were followed up until 15 June 2023 or their date of death. The original paper only considered mortality outcomes up to April 2015. By extending the follow-up period, we increase the number of mortality events considered in the analysis from 12,015 to 18,836. We excluded participants without data on BMI or genetic variants for BMI, leaving 56,150 individuals for analysis.

The UK Biobank study

The UK Biobank cohort comprises around 500,000 participants (94% of self-reported European ancestry) aged 40 to 69 years at baseline. They were recruited between 2006-2010 in 22 assessment centres throughout the UK, and followed up until 28 February 2021 or their date of death(21). Again, the original paper only considered mortality outcomes up to February 2016. By extending the follow-up period, we increase the number of mortality events considered in the analysis from 10,344 to 25,021. We performed detailed quality control procedures on UK Biobank participants and on genetic variants as described previously(22). In total, 366,385 unrelated European ancestry participants were included in analyses.

SNPs and genetic score used as instrumental variables

77 single nucleotide polymorphisms (SNPs) were selected as candidate instrumental variables for BMI based on European sex-combined analyses in a genome-wide association study of the Genetic Investigation of ANthropometric Traits (GIANT) consortium(23). Two of these variants (rs12016871 and rs2033732) were not available in the HUNT study, and a further two variants (rs13021737 and rs16951275) were excluded from the analyses due to association with smoking status in the HUNT study. An externally-weighted score was calculated for each individual by multiplying the number of BMI-increasing alleles the individual carries by the variant's association with BMI from the GIANT study (**Supplementary Table 1**), and summing across the remaining 73 variants. Overall, the genetic score explained 2.0% and 1.6% of the variance in BMI in the HUNT and UK Biobank studies respectively, corresponding to F-statistics of 1121 and 5964. As a supplementary analysis, we repeated analyses for all-cause mortality using an extended genetic score comprising 633 variants taken from a more recent genome-wide association study of the GIANT consortium, which included UK Biobank (24). The variant list was taken from Verkouter *et al (25)*, who pruned the initial set of reported variants to exclude correlated variants.

We assessed the instrumental variable assumptions within strata by estimating associations between the genetic score and various traits that are competing risk factors: smoking status (ever versus never), alcohol status (current versus other), education level (post-secondary or higher versus other), occupation (currently employed versus other), age at recruitment, and sex.

Study design

We performed non-linear Mendelian randomization analyses to estimate the shape of the association between genetically-predicted BMI and the outcome. Our primary analysis considered all-cause mortality as the outcome. We also conducted subgroup analyses considering men and

For peer review only - http://bmjopen.bmj.com/site/about/guidelines.xhtml

women separately. In addition, we studied associations with cause-specific mortality events (cardiovascular, cancer, and non-cardiovascular non-cancer) in UK Biobank. These were defined based on International Classification of Diseases, Tenth Revision (ICD-10) codes (see **Supplementary Table 2** for details). We did not perform analyses stratified by smoking status (as presented in the original paper), as smoking status is influenced by BMI(26), and hence this stratification may introduce collider bias.

Statistical analyses

To estimate the non-linear relationship between genetically-predicted BMI and mortality risk, a fractional polynomial method was applied(12). In brief, first we divided the sample into 100 strata using the doubly-ranked method(18). For comparison, we also present results from stratification using the residual method for the primary outcome of all-cause mortality. We then calculated the linear Mendelian randomization estimate, referred to as a localized average causal effect (LACE), in each stratum of the population as a ratio of coefficients: the association of the genetic score with the outcome divided by the association of the genetic score with the exposure. Associations with the exposure (BMI) were obtained from linear regression; associations with the outcome (mortality) were obtained from Cox proportional hazards regression, using age as the timescale. While Cox proportional hazards regression is a semi-parametric method, the estimated non-parametric baseline hazard function is a function of the timescale, not the exposure value. All associations were adjusted for age, age-squared, sex, and centre (for UK Biobank).

We performed meta-regression of the LACE estimates against the mean of the exposure in each stratum in a flexible semiparametric framework using the derivative of fractional polynomial models of degrees 1 and 2. Under the Mendelian randomization assumptions, the slope of the curve represents the average causal effect of the exposure on the outcome for the stratum with that exposure level. A positive causal effect is evident at a particular exposure level when the lower and upper confidence limits for the curve both have a positive slope; and similarly an inverse causal effect is evident if both confidence limits have a negative slope. The reference point in analyses is set to 25 kg/m^2; however, this reference point is arbitrarily chosen, and the key indicator of an increasing or decreasing effect is not whether confidence intervals include or exclude the reference point, but rather whether the slope of the curve is positive or negative at a given exposure value.

We report two tests for non-linearity: a linearity test, which assesses whether a non-linear model fits the LACE estimates better than a linear model, and a trend test, which tests for a linear trend amongst the LACE estimates. Rejection of the linearity test indicates that a linear model is a suboptimal fit to the data; failure to reject means any improvement in fit for the best fitting non-linear model over the linear model is no more than would be expected due to chance alone.

As LACE estimates from the doubly-ranked method can be sensitive to specification of the analytic sample, we repeated analyses 100 times for each dataset omitting a small number of individuals in each iteration (12 individuals were removed at random in each iteration), and then combined estimates across iterations using Rubin's rules.

All statistical analyses were performed in R (version 4.3.1), and non-linear Mendelian randomization analyses were performed using the SUMnlmr package(27).

Patient and public involvement

No patients were involved in setting the research question or the outcome measures, nor were they involved in the design or implementation of the study. No patients were asked to advise on

For peer review only - http://bmjopen.bmj.com/site/about/guidelines.xhtml

interpretation or writing up of results. There are no specific plans to disseminate the results of the research to study participants or the relevant patient community.

For peer review only

For peer review only - http://bmjopen.bmj.com/site/about/guidelines.xhtml

RESULTS

Study populations

Baseline characteristics of the participants are provided in **Table 1**. Genetic associations with BMI in strata of the population defined by the doubly-ranked method are displayed in **Supplementary Figure 1**, and the BMI distribution in strata of the population is given in **Supplementary Table 3**. Per 1 standard deviation increase in the genetic score, BMI was 0.358 kg/m^2 greater in the lowest percentile of participants in the HUNT study, and 1.126 kg/m^2 greater in the highest percentile. Equivalent values for UK Biobank were 0.232 kg/m^2 in the lowest percentile and 1.645 kg/m^2 in the highest percentile. It is clear that the genetic associations with BMI differ strongly at different levels of BMI, and so the assumption required for the residual stratification method is violated. We note that similar estimates of the genetic associations with the exposure from the residual stratification method are unreliable, as these estimates from the residual method are similar even if the genetic effect on the exposure varies in the population(18).

Assessment of instrument validity

Associations with traits that are competing risk factors in strata of UK Biobank are displayed in **Figure 1**. As previously observed in the literature for other exposures(28), there are clear patterns in genetic associations with age and sex across strata. While there is no overall association of the genetic score with age or sex, there are associations in several of the strata.

As it is logically impossible that autosomal genetic variants can affect either age or sex, and as age and sex are typically measured without error, these associations must represent the effect of selection into the UK Biobank sample. While concerning, as we adjust for age and sex in our Mendelian randomization analyses, these associations will not lead to strong bias in our causal estimates (see **Supplementary Material** for a simulation study investigation into bias mitigation by adjustment for confounders that are predictors of selection). For other traits, both the magnitude of associations and patterns in associations are less strong, particularly for education level and occupation, which are important measures of social class. For alcohol status and smoking status, it is impossible to distinguish between genetic associations that reflect pleiotropic or selection effects and those that reflect a causal effect of the exposure (sometimes called “vertical pleiotropy”); the latter do not violate the instrumental variable assumptions. Hence both the associations and any pattern of associations may reflect violations of the instrumental variable assumptions or selection bias, but they could also reflect downstream effects of BMI which vary across strata.

Corresponding associations in the HUNT study are shown in **Supplementary Figure 2**. While there are some patterns in the stratum associations with age and sex, these are less pronounced than for UK Biobank, potentially due to differential selection being less strong in the HUNT study.

Comparison of results from residual and doubly-ranked methods

The shape of the association between genetically-predicted BMI and all-cause mortality is displayed in **Figure 2**, and stratum-specific LACE estimates are provided in **Supplementary Figure 3**. We note that **Figure 2** and **Supplementary Figure 3** differ in their presentation of these data; **Figure 2** plots the estimated association between genetically-predicted BMI values and mortality risk with respect to the reference exposure value, whereas **Supplementary Figure 3** plots the estimated effect of the exposure on mortality risk in each stratum. A smoothed version of the plot in **Supplementary Figure 3** would represent the derivative of the curve shown in **Figure 2**.

For peer review only - http://bmjopen.bmj.com/site/about/guidelines.xhtml

When stratifying the population using the residual method, there is a distinct J-shaped relationship between BMI and mortality, particularly in the UK Biobank study, with minimum risk at a BMI level of around 25 kg/m^2. However, as discussed above, results from the residual stratification method are unreliable here. In contrast, when stratifying using the doubly-ranked method, the relationship between BMI and mortality is far more uncertain at the lower end of the BMI distribution. In the HUNT study, the association is mildly J-shaped, with a null slope up to a BMI of around 28 kg/m^2, and a positive slope above this level. For UK Biobank, there is some indication of an upturn in mortality risk at very low levels of BMI (the slope of the curve is negative below 20 kg/m^2), but otherwise the relationship is flat (and compatible with a null effect) up to a BMI of around 25 kg/m^2, and has an increasing positive slope above this level. The linearity and trend tests for the residual method indicate evidence supporting non-linearity for UK Biobank ($p_{linearity} = 5\times10^{-4}$, $p_{trend} = 2\times10^{-5}$). In contrast, these tests for the more reliable doubly-ranked method do not provide compelling evidence supporting non-linearity (**Figure 2**).

Similar results were observed when inverse-normal rank transforming the exposure (**Supplementary Figure 4**). When dividing the population into 50 strata, results were similar in both studies. When dividing into 200 strata, results were similar for UK Biobank, but differed for the HUNT study, as the best-fitting curve was monotone with no J-shaped upturn at the lower end of the BMI distribution (**Supplementary Figure 5**). When using the extended genetic score (633 variants) in UK Biobank, a pattern of results similar to the main analysis was observed, but with narrower confidence intervals as the extended score explains more variance in BMI (**Supplementary Figure 6**). The was still some indication of a J-shaped curve, although minimum mortality risk was at a BMI level of 20-21 kg/m^2, and evidence for an upturn in risk at low BMI levels was weaker.

Sex-stratified analyses

Sex-stratified analyses from the doubly-ranked method are presented in **Figure 3**, and stratum-specific LACE estimates are provided in **Supplementary Figure 7**. In each case, there is some indication of a J-shaped relationship, but an upturn in mortality risk is only evident below 20 kg/m^2 (below 22 kg/m^2 for men in the HUNT study). Otherwise, estimates are generally compatible with the null up to a BMI of 25 kg/m^2, and positive above this level. Evidence for a harmful effect of increases in BMI for both men and women is weaker in the HUNT study, although a harmful effect above 30 kg/m^2 is evident for men. The slope above 25 kg/m^2 is steeper for women than men in UK Biobank. The linearity and trend tests do not provide compelling evidence supporting non-linearity in any analysis (**Figure 3**).

Cause-specific mortality

Analyses investigating cause-specific mortality from the doubly-ranked method in UK Biobank are presented in **Figure 4**, and stratum-specific LACE estimates are provided in **Supplementary Figure 8**. Again, there is some indication of a J-shaped relationship for each outcome. This is most pronounced for cardiovascular mortality, and less evident for cancer and non-cardiovascular non-cancer mortality. An upturn in risk at low BMI levels was only evident for cardiovascular mortality, and only below 20 kg/m^2. Otherwise, the relationship was compatible with the null up to around 24 kg/m^2 for cardiovascular mortality, 26 kg/m^2 for non-cardiovascular non-cancer mortality, and 30 kg/m^2 for cancer mortality, and positive above this level. The slope above 25 kg/m^2 is steepest for cardiovascular mortality, then non-cardiovascular non-cancer, and finally for cancer mortality. The linearity and trend tests do not provide any evidence supporting non-linearity for any outcome, indicating that a non-linear model does not fit the data significantly better than a linear model for any outcome (**Figure 4**).

For peer review only - http://bmjopen.bmj.com/site/about/guidelines.xhtml

DISCUSSION

In this manuscript, we have investigated the shape of associations between genetically-predicted BMI and mortality outcomes to provide an indication of the causal effect of BMI on mortality risk at different levels of BMI using non-linear Mendelian randomization. While the curves generally display a J-shaped relationship, evidence for a harmful average effect of decreased BMI on mortality at low BMI levels was limited. Evidence supporting non-linearity from statistical hypothesis tests for the doubly-ranked method was unconvincing for all mortality outcomes. In UK Biobank, the slope representing the estimated effect of BMI on mortality risk above 25 kg/m^2 was sharper for women than for men, and sharpest for cardiovascular mortality compared with cancer mortality and non-cardiovascular non-cancer mortality. There was evidence for a harmful effect of high BMI on all mortality subtypes.

Our findings challenge "obesity paradox" results from observational epidemiology and previous Mendelian randomization analyses using the residual method, which suggest that increases in BMI might reduce risk of mortality for a substantial proportion of the general population. While the minimum of the estimated curve for all-cause mortality was at a BMI of around 23 kg/m^2 in UK Biobank, the curve was near flat from 22-24 kg/m^2, and was positive from 25 kg/m^2 upwards. Evidence for a harmful effect of decreased BMI at low levels of BMI (as indicated by negative slopes for both the upper and lower confidence limits of the BMI—mortality curve) was only present in some analyses, and only below 20 kg/m^2 when present. Amongst cause-specific mortality outcomes, this was only evident for cardiovascular mortality. In the HUNT study, there was no strong evidence for an effect of BMI on all-cause mortality at low BMI levels in overall analyses, and evidence for a harmful effect of increased BMI above around 28 kg/m^2. A potential reason for the discrepancy is that UK Biobank only contains participants from the age of 40 upwards, whereas the HUNT study includes younger individuals. Hence underweight strata in UK Biobank are more likely to comprise primarily of older, frailer individuals, whereas underweight strata in the HUNT study include more healthy, slim individuals.

Our investigation provides further empirical evidence that the assumptions required for the residual stratification method for non-linear Mendelian randomization are likely to be violated in practice, and that results from the residual method can be substantially different to those from the doubly-ranked method(17). The ratio between the genetic association with BMI in the highest versus lowest percentile group was 3.1 in the HUNT study and 7.1 in UK Biobank, whereas the assumptions required by the residual method imply that these associations should be constant across the distribution of the exposure. Particularly in UK Biobank, the residual method provided strong evidence for non-linearity in the effect of BMI on mortality risk, giving a J-shaped relationship with the minimum risk level at around 25 kg/m^2. This is similar to the confounded association observed in traditional observational studies(2, 3). In contrast, the doubly-ranked method provided little evidence for non-linearity. While there was some indication of a J-shaped curve in all analyses, estimates at the lower end of the BMI distribution were imprecise. One reason for this is that genetic associations with BMI were weaker at low levels of BMI in the doubly-ranked method, and so stratum-specific Mendelian randomization estimates are less precise in these strata.

Previous investigations into the non-linear shape of the causal relationship between BMI and mortality have used a variety of approaches. Wade *et al* used the residual stratification method for non-linear Mendelian randomization in UK Biobank(29), which showed a J-shaped relationship similar to the one we observe here. Jenkins *et al* investigated associations between a genetic score for BMI and mortality in UK Biobank(5), considering analyses in the full dataset, and restricted to those with a pre-existing morbidity condition. While they did not observe evidence for non-linearity,

For peer review only - http://bmjopen.bmj.com/site/about/guidelines.xhtml

the genetic score only explains a small proportion of variance in BMI, and so substantial non-linearity may not be expected on this scale. The difference between average BMI levels at the lowest versus highest decile of the genetic score is less than 2 kg/m^2. Sulc *et al* investigated the effect of BMI on life expectancy (estimated as the mean of parental lifespan) in a Mendelian randomization framework using a polynomial approach similar to the residual method(30), except that instead of stratifying on the residual exposure, this approach constructs a polynomial function of the residual exposure. They showed a linear effect of increased BMI on reduced life expectancy, with no indication of non-linearity. However, this approach also has been shown in simulations to be vulnerable to variation in genetic effects on the exposure(18). Carslake *et al* performed an instrumental variable analysis to investigate the impact of BMI on mortality in the HUNT study(31), but using offspring BMI as an instrument for the BMI of study participants. This analysis did not provide evidence for a harmful effect of having low BMI. However, offspring BMI is unlikely to satisfy the instrumental variable assumptions for the exposure of participant BMI. A similar analysis was performed in the 1958 British birth cohort, and a similar conclusion was reached(32). Blond *et al* also performed an instrumental variable analysis using offspring BMI as an instrument for the BMI of study participants in the Copenhagen School Health Records Register(33), showing evidence of positive effects of increases in BMI on mortality at both high and low BMI levels. Finally, Kjøllesdal et al performed an instrumental variable analysis using early adulthood BMI as an instrument for the midlife BMI of study participants in various Norwegian health surveys(34), showing mild J-shaped associations in the relationship between BMI and mortality, but far less pronounced than from observational analyses of the same dataset.

Overall, there is general consensus from different approaches that the adverse relationship of low BMI with mortality is exaggerated in observational analyses. However, variation between these results indicates the importance of modelling assumptions, both for the identification of causal effects, and for the modelling of non-linear relationships. Indeed, in our investigations, if we had used fractional polynomials of degree 1 rather than degree 2, then we would have observed monotonic increasing relationships of BMI with all outcomes. This is because the simpler degree 1 models are dominated by behaviour at the upper end of the BMI distribution, where instruments are strongest and most mortality outcomes occur. The simpler degree 1 polynomials smooth over imprecise negative estimates at the lower end of the BMI distribution, and so do not allow uncertainty in the shape of the distribution (particularly at its lower end) to be accurately reflected.

Our analysis has several strengths, but also limitations. The Mendelian randomization design minimizes bias from confounding and reverse causation. The large sample sizes of the HUNT and UK Biobank studies enable powerful analyses, even in strata of the population with low BMI levels. These sample sizes also enable a fine stratification of individuals, meaning that outcome associations can be assessed in strata only consisting of low BMI (<20 kg/m^2) individuals.

However, there are important limitations, which should lead to caution in the interpretation of our findings. As with all Mendelian randomization analyses, findings are dependent on the validity of the genetic variants as instrumental variables. While we were able to show null associations of the genetic score with some traits, it is never possible to prove the validity of the instrumental variable assumptions beyond all doubt. To reduce the scope for population stratification, our analyses were limited to European ancestry participants. Our findings may therefore not be applicable to other populations. Further, recruitment into UK Biobank is dependent on age, sex, and other factors, which leads to bias in Mendelian randomization estimates. While the effect of moderate selection on Mendelian randomization estimates is often slight(35), the extent of selection bias may differ between strata, leading to differential bias across stratum-specific estimates(28). It is likely that age

For peer review only - http://bmjopen.bmj.com/site/about/guidelines.xhtml

and sex are the strongest predictors of study participants, and differential genetic associations with these traits were observed in UK Biobank. For other traits, patterns in genetic associations across strata were less evident, and may reflect downstream effects of BMI rather than instrument invalidity due to pleiotropy or selection bias. As the HUNT study focused on a specific geographic area of Norway, it achieved much higher recruitment rates (around 70%, compared with 5% in UK Biobank), and so should be less affected by selection bias. This was corroborated by our analyses, as genetic associations in strata were weaker in the HUNT study. While genetic associations are protected from reverse causation, strata membership is determined by BMI levels, and hence could be subject to reverse causation. In particular, low BMI strata could contain a large proportion of individuals whose BMI levels are low because of comorbidity. However, genetic associations should still provide a reliable guide as to the extent and direction of any causal effect of BMI in these strata. Finally, all estimates represent averages across strata of the population; individual effects of raising or lowering BMI may vary between individuals. Differences in estimates for different strata may reflect the changing composition of the strata, as the characteristics of those with BMI less than 20 kg/m^2 are likely to be different to those with higher BMI. A non-linear curve may reflect a combination of different effects in different subgroups of the population, rather than that the relationship between BMI and mortality risk is non-linear for any individual in the population(36).

Conclusions

In conclusion, non-linear Mendelian randomization analyses using the doubly-ranked stratification method provide strong evidence for harmful effects of increased BMI on mortality above 25 kg/m^2. Evidence for a harmful effect of low BMI was only present in some analyses, and where present, only below 20 kg/m^2.

For peer review only

Table 1: Baseline characteristics of study participants in HUNT and UK Biobank studies

	HUNT	UK Biobank
Number of participants (n)	56,150	366,385
Men (%)	47.1	45.9
Age at baseline in years (SD)	49.6 (16.6)	56.7 (8.0)
Number of deaths (n)	18,836	25,021
- Cardiovascular mortality events (n)	-	5212
- Cancer mortality events (n)	-	12,880
- Other (non-cardiovascular non-cancer) events (n)	-	6692
BMI in kg/m^2 (SD)	26.3 (4.1)	27.4 (4.8)
Median follow-up years	18.5	7.0
Ever-smokers (%)	55.9	46.1

BMI: body mass index; SD: standard deviation; Data are given as number of subjects (n), percentage (%), median, or mean (standard deviation). For 237 deaths in UK Biobank, sufficient data were not available to classify the event as cardiovascular, cancer, or non-cardiovascular non-cancer.

For peer review only

FIGURE CAPTIONS

Figure 1: Genetic associations with potential competing risk factors in strata of the UK Biobank population

Figure 2: Non-linear Mendelian randomization – dose-response curve between body mass index and all-cause mortality. Using the doubly-ranked method: A) HUNT, B) UK Biobank; using the residual method (unreliable when the genetic effect on the exposure varies): C) HUNT, D) UK Biobank. Gradient at each point of the curve is the localized average causal effect. Grey lines represent 95% confidence intervals. The reference value for BMI was taken as 25 kg/m^2.

Figure 3: Non-linear Mendelian randomization – dose-response curve between body mass index and all-cause mortality in men and women respectively using doubly-ranked method. A) HUNT, men only; B) HUNT, women only; C) UK Biobank, men only; D) UK Biobank, women only. Gradient at each point of the curve is the localized average causal effect. Grey lines represent 95% confidence intervals. The reference value for BMI was taken as 25 kg/m^2.

Figure 4: Non-linear Mendelian randomization in UK Biobank – dose-response curve between body mass index and cause-specific mortality using doubly-ranked method. A) Cardiovascular disease mortality; B) Cancer mortality. C) Mortality due to other causes (non-cardiovascular, non-cancer). Gradient at each point of the curve is the localized average causal effect. Grey lines represent 95% confidence intervals. The reference value for BMI was taken as 25 kg/m^2.

For peer review only - http://bmjopen.bmj.com/site/about/guidelines.xhtml

Contributor statement:

SB, YQS, and XMM conceptualized the project. SB and YQS performed formal analysis. All authors contributed to the methodology and interpretation of findings. YQS and AMM helped with data curation. SB and XMM supervised the project. SB wrote the initial manuscript draft. All authors contributed to the drafting and editing of the manuscript. All authors have approved of the final version of the manuscript.

Competing interest statement: None declared.

Funding statement: This work was supported by the Wellcome Trust (grant number 225790/Z/22/Z), the United Kingdom Research and Innovation Medical Research Council (grant number MC_UU_00002/7), the British Heart Foundation (RG/18/13/33946) and the National Institute for Health and Care Research Cambridge Biomedical Research Centre (BRC-1215-20014; NIHR203312). YQS was supported by a researcher grant from The Liaison Committee for Education, Research and Innovation in Central Norway (project ID 2018/42794). AMM is funded by the National Institute for Health and Care Research Blood and Transplant Research Unit in Donor Health and Behaviour (NIHR203337). The views expressed are those of the authors and not necessarily those of the NIHR, NHSBT or the Department of Health and Social Care.

Data sharing statement: Data from the HUNT and UK Biobank studies are available on application to any bona fide scientific researcher. Summary statistics for genetic associations with the exposure and outcome in strata for UK Biobank are available at http://doi.org/10.6084/m9.figshare.25041686.

Ethical approval

The HUNT study has ethical approval from The Regional Committee for Medical Research in Norway (REK 2015/78). Approval for individual projects is regulated in conjunction with The Norwegian Social Science Data Services (NSD). The UK Biobank study has ethical approval from the North West Multi-centre Research Ethics Committee (16/NW/0274). This research has been conducted using the UK Biobank Resource under Application Number 7439.

Acknowledgments

The Trøndelag Health Study (HUNT) is a collaboration between the HUNT Research Centre (Faculty of Medicine and Health Sciences, NTNU, Norwegian University of Science and Technology), Trøndelag County Council, Central Norway Regional Health Authority and the Norwegian Institute of Public Health.

For peer review only

REFERENCES

1. Aune D, Sen A, Prasad M, Norat T, Janszky I, Tonstad S, et al. BMI and all cause mortality: systematic review and non-linear dose-response meta-analysis of 230 cohort studies with 3.74 million deaths among 30.3 million participants. BMJ. 2016;353:i2156.
2. Bhaskaran K, dos-Santos-Silva I, Leon DA, Douglas IJ, Smeeth L. Association of BMI with overall and cause-specific mortality: a population-based cohort study of 3.6 million adults in the UK. The Lancet Diabetes & Endocrinology. 2018;6(12):944-53.
3. Global BMI Mortality Collaboration, Di Angelantonio E, Bhupathiraju Sh N, Wormser D, Gao P, Kaptoge S, et al. Body-mass index and all-cause mortality: individual-participant-data meta-analysis of 239 prospective studies in four continents. Lancet. 2016;388(10046):776-86.
4. Flegal KM, Kit BK, Orpana H, Graubard BI. Association of All-Cause Mortality With Overweight and Obesity Using Standard Body Mass Index Categories: A Systematic Review and Meta-analysis. JAMA. 2013;309(1):71-82.
5. Jenkins DA, Wade KH, Carslake D, Bowden J, Sattar N, Loos RJF, et al. Estimating the causal effect of BMI on mortality risk in people with heart disease, diabetes and cancer using Mendelian randomization. International journal of cardiology. 2021;330:214-20.
6. Visaria A, Setoguchi S. Body mass index and all-cause mortality in a 21st century U.S. population: A National Health Interview Survey analysis. PLOS ONE. 2023;18(7):e0287218.
7. Davey Smith G, Ebrahim S. 'Mendelian randomization': can genetic epidemiology contribute to understanding environmental determinants of disease? International Journal of Epidemiology. 2003;32(1):1-22.
8. Lawlor DA, Harbord RM, Sterne JA, Timpson N, Davey Smith G. Mendelian randomization: using genes as instruments for making causal inferences in epidemiology. Statistics in Medicine. 2008;27(8):1133-63.
9. Thanassoulis G, O'Donnell CJ. Mendelian randomization: nature's randomized trial in the post-genome era. JAMA. 2009;301(22):2386-8.
10. Burgess S, Swanson SA, Labrecque JA. Are Mendelian randomization investigations immune from bias due to reverse causation? European Journal of Epidemiology. 2021;36(3):253-7.
11. Martens EP, Pestman WR, de Boer A, Belitser SV, Klungel OH. Instrumental variables: application and limitations. Epidemiology. 2006:260-7.
12. Staley JR, Burgess S. Semiparametric methods for estimation of a nonlinear exposure-outcome relationship using instrumental variables with application to Mendelian randomization. Genet Epidemiol. 2017;41(4):341-52.
13. Burgess S, Davies NM, Thompson SG. Instrumental variable analysis with a nonlinear exposure-outcome relationship. Epidemiology. 2014;25(6):877-85.
14. Cole SR, Platt RW, Schisterman EF, Chu H, Westreich D, Richardson D, Poole C. Illustrating bias due to conditioning on a collider. International Journal of Epidemiology. 2010;39(2):417-20.
15. Small DS. Commentary: Interpretation and Sensitivity Analysis for the Localized Average Causal Effect Curve. Epidemiology. 2014;25(6).
16. Davey Smith G. Mendelian randomisation and vitamin D: the importance of model assumptions. The Lancet Diabetes & Endocrinology. 2023;11(1):14.
17. Burgess S. Violation of the constant genetic effect assumption can result in biased estimates for non-linear Mendelian randomization. Human Heredity. 2022(In Press).
18. Tian H, Mason AM, Liu C, Burgess S. Relaxing parametric assumptions for non-linear Mendelian randomization using a doubly-ranked stratification method. PLOS Genetics. 2023;19(6):e1010823.
19. Sun Y-Q, Burgess S, Staley JR, Wood AM, Bell S, Kaptoge SK, et al. Body mass index and all cause mortality in HUNT and UK Biobank studies: linear and non-linear mendelian randomisation analyses. British Medical Journal. 2019;364:l1042.
20. Krokstad S, Langhammer A, Hveem K, Holmen TL, Midthjell K, Stene TR, et al. Cohort Profile: The HUNT Study, Norway. International Journal of Epidemiology. 2013;42(4):968-77.

For peer review only - http://bmjopen.bmj.com/site/about/guidelines.xhtml

21. Sudlow C, Gallacher J, Allen N, Beral V, Burton P, Danesh J, et al. UK Biobank: an open access resource for identifying the causes of a wide range of complex diseases of middle and old age. PLOS Medicine. 2015;12(3):e1001779.
22. Astle WJ, Elding H, Jiang T, Allen D, Ruklisa D, Mann AL, et al. The allelic landscape of human blood cell trait variation and links to common complex disease. Cell. 2016;167(5):1415-29.
23. Locke AE, Kahali B, Berndt SI, Justice AE, Pers TH, Day FR, et al. Genetic studies of body mass index yield new insights for obesity biology. Nature. 2015;518(7538):197-206.
24. Yengo L, Sidorenko J, Kemper KE, Zheng Z, Wood AR, Weedon MN, et al. Meta-analysis of genome-wide association studies for height and body mass index in ~700000 individuals of European ancestry. Human Molecular Genetics. 2018;27(20):3641-9.
25. Verkouter I, de Mutsert R, Smit RAJ, Trompet S, Rosendaal FR, van Heemst D, et al. The contribution of tissue-grouped BMI-associated gene sets to cardiometabolic-disease risk: a Mendelian randomization study. International Journal of Epidemiology. 2020;49(4):1246-56.
26. Carreras-Torres R, Johansson M, Haycock PC, Relton CL, Davey Smith G, Brennan P, Martin RM. Role of obesity in smoking behaviour: Mendelian randomisation study in UK Biobank. BMJ. 2018;361:k1767.
27. Mason AM, Burgess S. Software Application Profile: SUMnlmr, an R package that facilitates flexible and reproducible non-linear Mendelian randomization analyses. International Journal of Epidemiology. 2022;51(6):2014-9.
28. Hamilton FW, Hughes DA, Spiller W, Tilling K, Davey Smith G. Non-linear mendelian randomization: evaluation of biases using negative controls with a focus on BMI and Vitamin D. medRxiv. 2023:2023.08.21.23293658.
29. Wade KH, Carslake D, Sattar N, Davey Smith G, Timpson NJ. BMI and Mortality in UK Biobank: Revised Estimates Using Mendelian Randomization. Obesity. 2018;26(11):1796-806.
30. Sulc J, Sjaarda J, Kutalik Z. Polynomial Mendelian randomization reveals non-linear causal effects for obesity-related traits. HGG Advances. 2022;3(3):100124.
31. Carslake D, Davey Smith G, Gunnell D, Davies N, Nilsen TIL, Romundstad P. Confounding by ill health in the observed association between BMI and mortality: evidence from the HUNT Study using offspring BMI as an instrument. International Journal of Epidemiology. 2018;47(3):760-70.
32. Hyppönen E, Carslake D, Berry DJ, Power C, Davey Smith G. Estimating the influence of body mass index (BMI) on mortality using offspring BMI as an instrumental variable. International Journal of Obesity. 2022;46(1):77-84.
33. Blond K, Carslake D, Gjærde LK, Vistisen D, Sørensen TIA, Smith GD, Baker JL. Instrumental variable analysis using offspring BMI in childhood as an indicator of parental BMI in relation to mortality. Scientific Reports. 2021;11(1):22408.
34. Kjøllesdal MKR, Davey Smith G, Ariansen I, Kinge JM, Degerud E, Næss Ø. The association between BMI and mortality using early adulthood BMI as an instrumental variable for midlife BMI. Scientific Reports. 2018;8(1):11499.
35. Gkatzionis A, Burgess S. Contextualizing selection bias in Mendelian randomization: how bad is it likely to be? International Journal of Epidemiology. 2018;48(3):691-701.
36. Small DS. Commentary: Interpretation and sensitivity analysis for the localized average causal effect curve. Epidemiology. 2014;25(6):886-8.

For peer review only - http://bmjopen.bmj.com/site/about/guidelines.xhtml

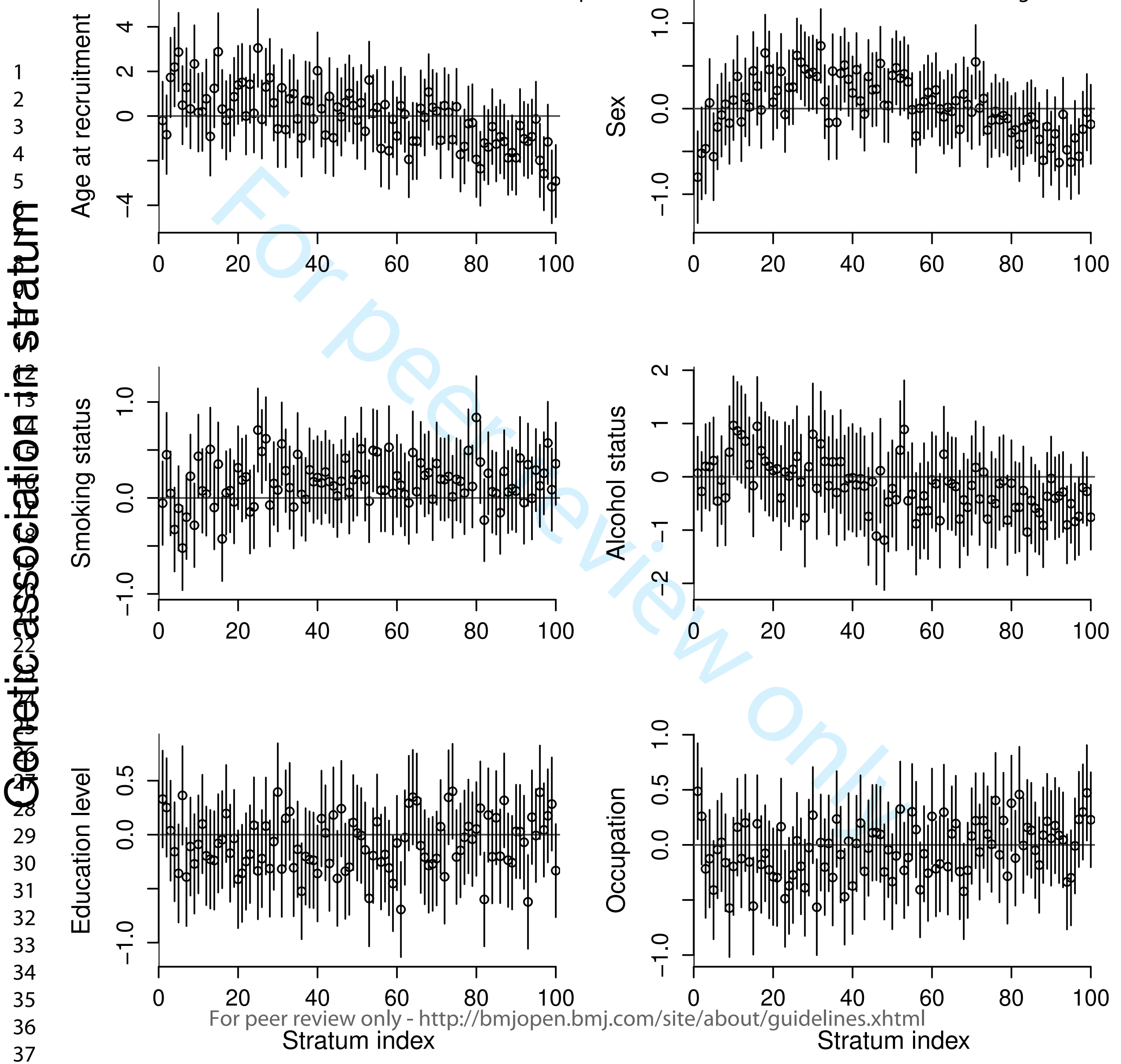

For peer review only

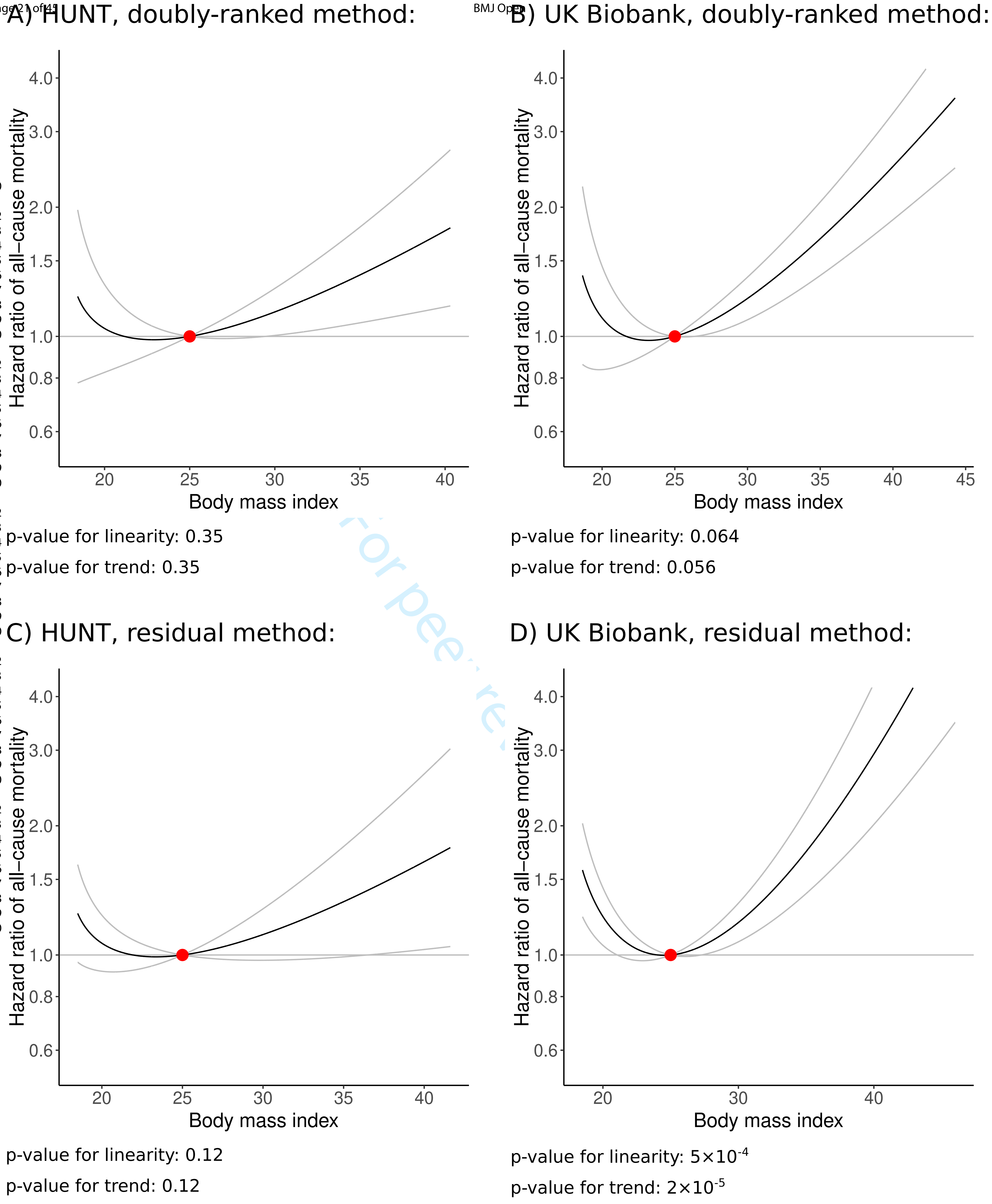
A) HUNT, doubly-ranked method:
Hazard ratio of all−cause mortality
4.0
3.0
2.0
1.5
1.0
0.8
0.6
20
25
30
35
40
Body mass index
p-value for linearity: 0.35
p-value for trend: 0.35
B) UK Biobank, doubly-ranked method:
Hazard ratio of all−cause mortality
Body mass index
45
p-value for linearity: 0.064
p-value for trend: 0.056
C) HUNT, residual method:
Hazard ratio of all−cause mortality
Body mass index
p-value for linearity: 0.12
p-value for trend: 0.12
D) UK Biobank, residual method:
Hazard ratio of all−cause mortality
Body mass index
p-value for linearity: 5×10-4
p-value for trend: 2×10-5

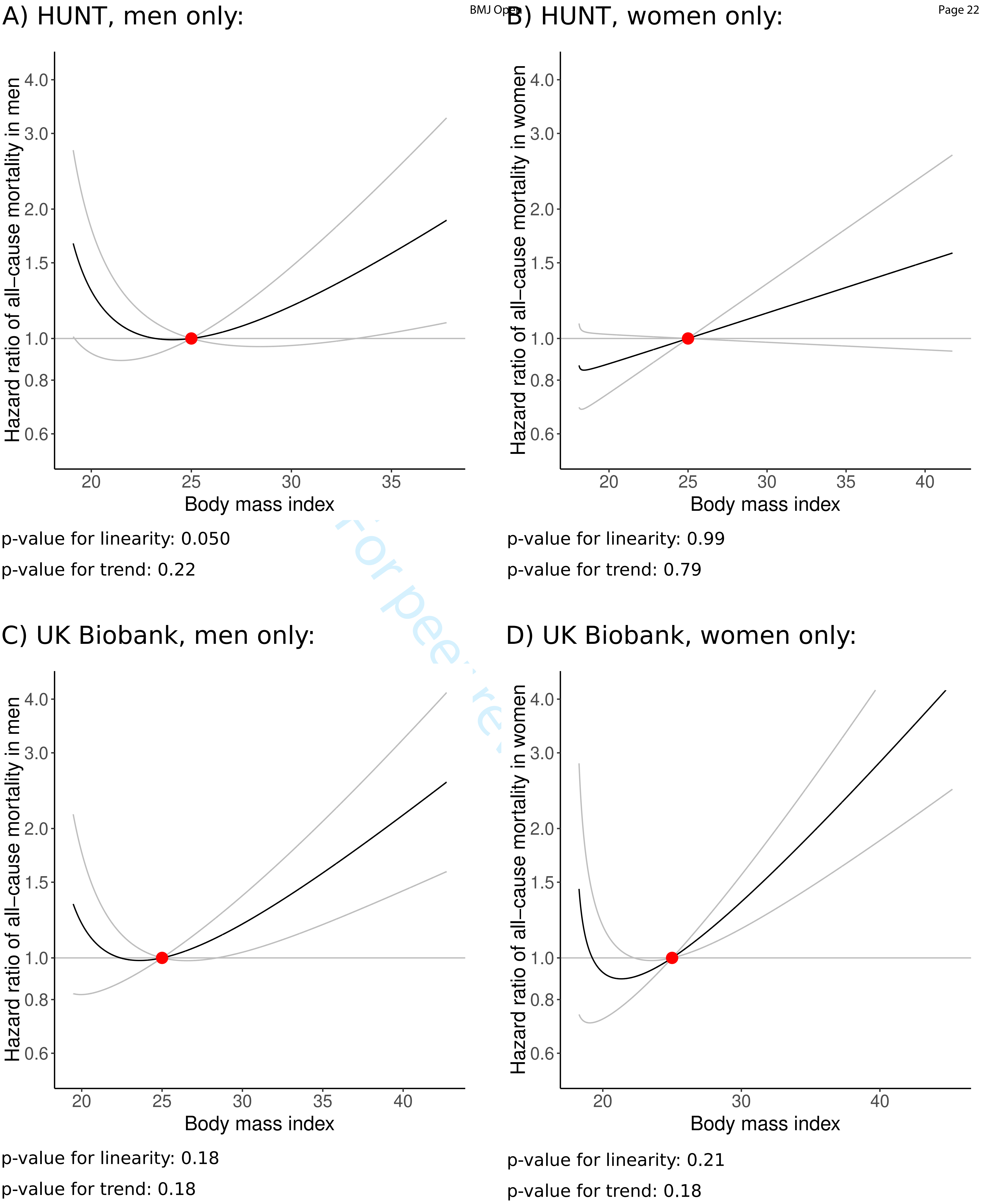
A) HUNT, men only:
Hazard ratio of all–cause mortality in men
4.0
3.0
2.0
1.5
1.0
0.8
0.6
20
25
30
35
Body mass index
p-value for linearity: 0.050
p-value for trend: 0.22
B) HUNT, women only:
Hazard ratio of all–cause mortality in women
4.0
3.0
2.0
1.5
1.0
0.8
0.6
20
25
30
35
40
Body mass index
p-value for linearity: 0.99
p-value for trend: 0.79
C) UK Biobank, men only:
Hazard ratio of all–cause mortality in men
4.0
3.0
2.0
1.5
1.0
0.8
0.6
20
25
30
35
40
Body mass index
p-value for linearity: 0.18
p-value for trend: 0.18
D) UK Biobank, women only:
Hazard ratio of all–cause mortality in women
4.0
3.0
2.0
1.5
1.0
0.8
0.6
20
30
40
Body mass index
p-value for linearity: 0.21
p-value for trend: 0.18

A) Cardiovascular mortality:

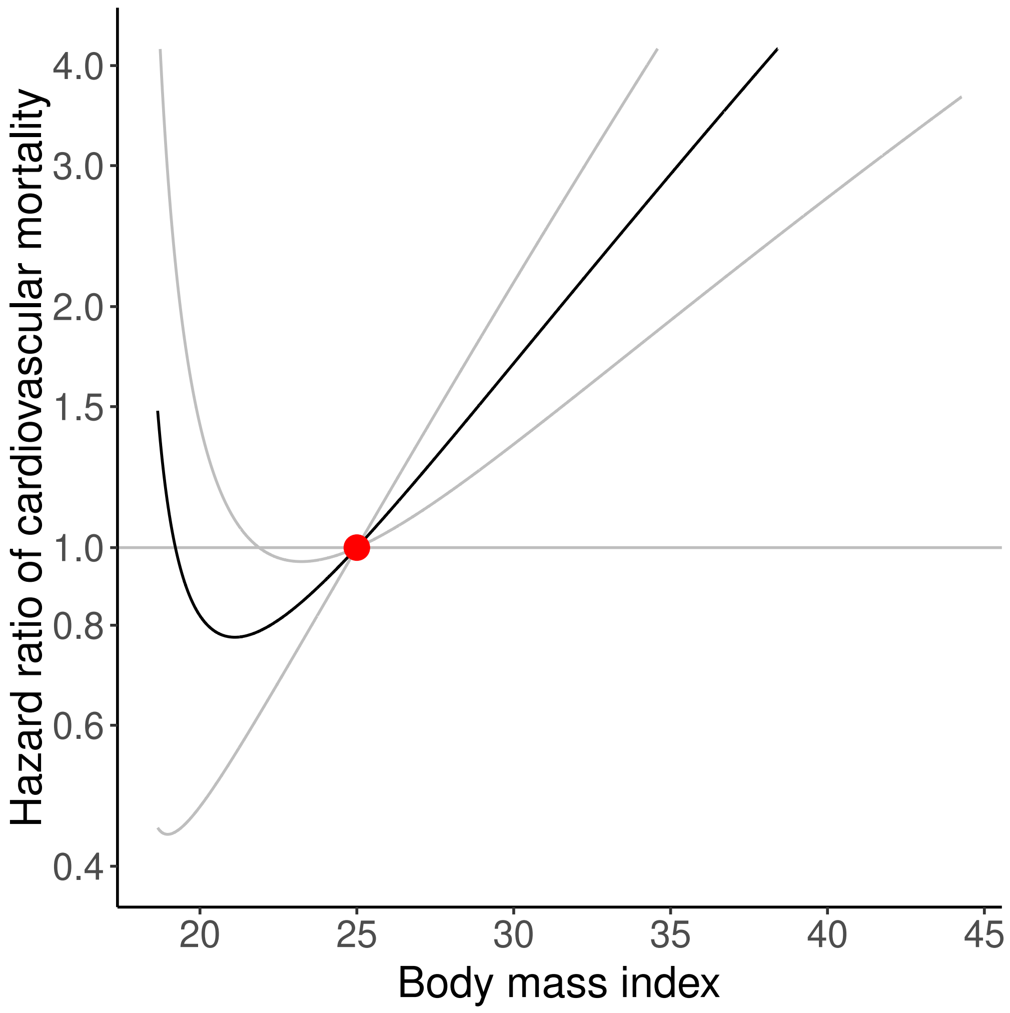

p-value for linearity: 0.99

p-value for trend: 0.92

B) Cancer mortality:

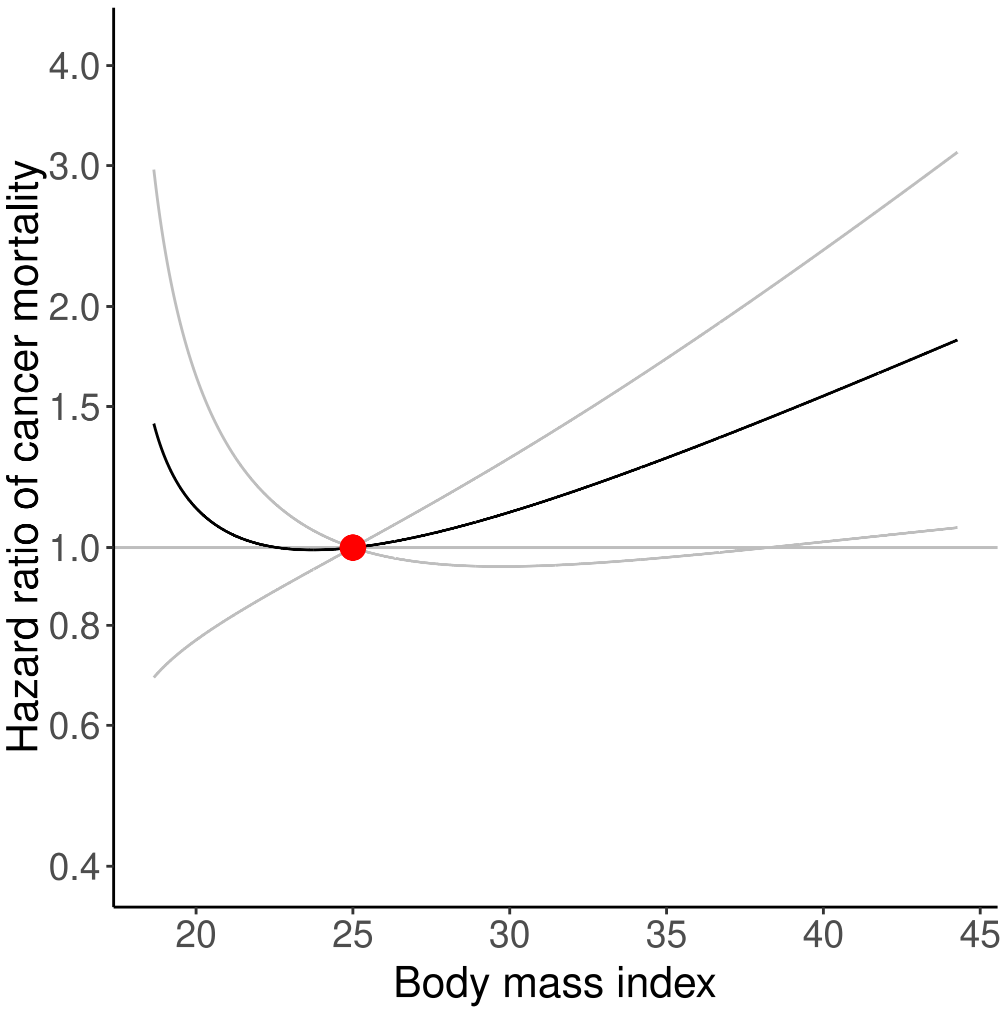

p-value for linearity: 0.39

p-value for trend: 0.38

C) Other (non-cardiovascular, non-cancer mortality):

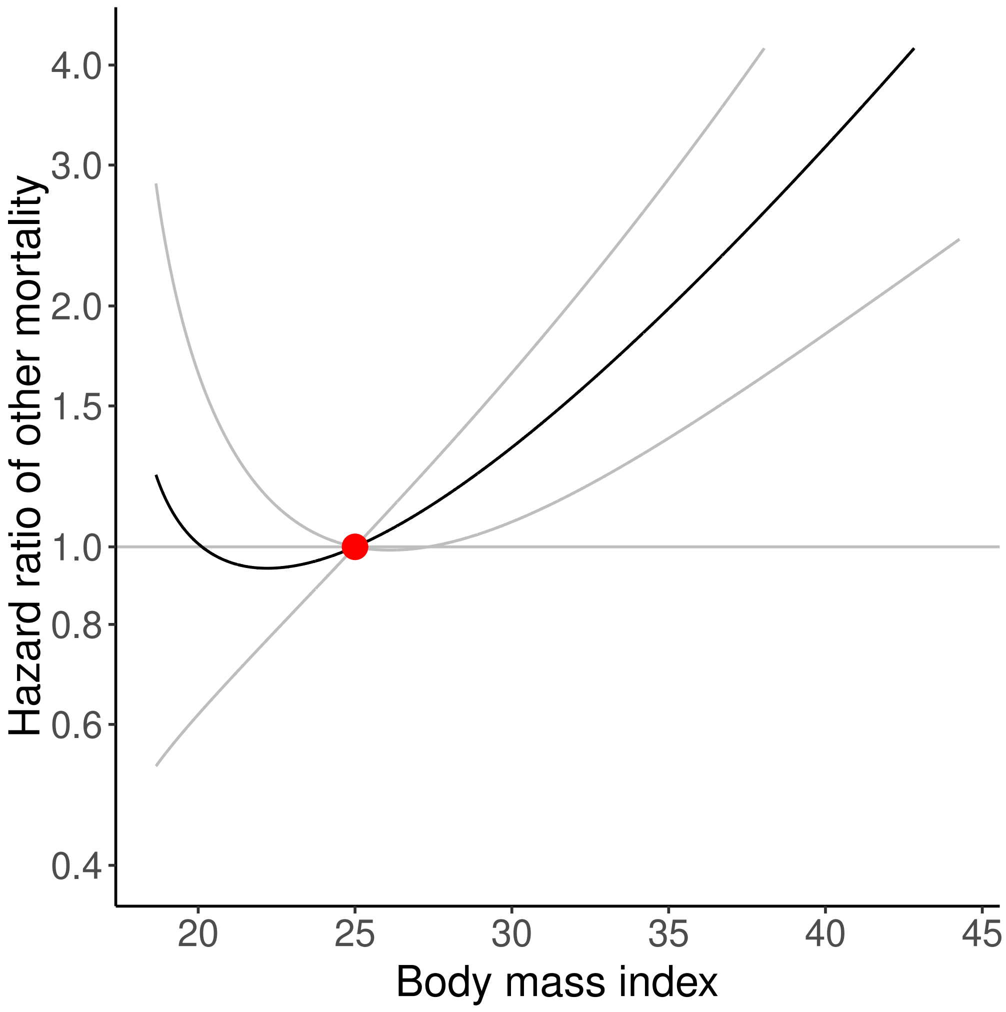

p-value for linearity: 0.20

p-value for trend: 0.19

SUPPLEMENTARY MATERIAL

We here perform a simulation study investigating whether adjusting genetic associations for factors that influence selection reduces selection bias within strata in a non-linear Mendelian randomization framework. We generate simulated data for 1,000,000 individuals on a genetic variant *G*, an exposure *X*, an outcome *Y*, and competing risk factors *U* and *V*. For simplicity, all variables are modelled as continuous and normally distributed.

We consider two scenarios. In each scenario, the genetic variant is a valid instrumental variable in the population as a whole, and there is no true effect of the exposure on the outcome. This implies that the genetic variant should not be associated with the outcome. We estimate the association between the genetic variant and the outcome in a selected subset of the population.

In scenario 1, the data-generating model is:

$$G_i \sim Normal(0,1)$$
$$U_i \sim Normal(0,1)$$
$$V_i \sim Normal(0,1)$$
$$X_i \sim Normal(0.2\ G_i + 0.4\ U_i + 0.4V_i, 1)$$
$$Y_i \sim Normal(-0.5\ U_i + 0.2\ V_i, 1)$$

Participants (indexed by the *i* subscript) are selected into the sample with probability $expit(-2 + U_i + X_i)$, where $expit(x) = \frac{\exp(x)}{1+\exp(x)}$ is the inverse of the logistic (logit) function. Here *U* and *V* are both confounders of the relationship between the exposure and outcome, and selection depends on both *U* and *X*. We compare genetic associations with the outcome with and without adjustment for *U*.

In scenario 2, the data-generating model is:

$$G_i \sim Normal(0,1)$$
$$V_i \sim Normal(0,1)$$
$$X_i \sim Normal(0.2\ G_i + 0.8\ V_i, 1)$$
$$U_i \sim Normal(X_i, 1)$$
$$Y_i \sim Normal(0.5\ V_i, 1)$$

Participants are selected into the sample with probability $expit(-2 + U_i)$. Here *V* is a confounder (common cause) of the relationship between the exposure and outcome, and *U* is a downstream effect of the exposure *X*, and hence *U* is a collider (common effect) of *G* and *V*. Selection depends on *U* only. Again, we compare genetic associations with the outcome with and without adjustment for *U*.

In both cases, all normal distributions are independent. We simulate 1000 datasets for each scenario, and compare the mean estimate of the genetic association with the outcome and its empirical Type 1 error rate at a 5% significance level (that is, the proportion of 95% confidence intervals excluding the null). Around 15-20% of the 1,000,000 participants are selected in each simulated dataset.

In scenario 1, the mean association of the genetic variant with the outcome without adjustment for *U* was 0.014 (mean standard error 0.002), and the empirical Type 1 error rate was 100.0%. With adjustment for *U*, the mean association was -0.002 (mean standard error 0.002), and the empirical Type 1 error rate was 17.1%.

In scenario 2, the mean association of the genetic variant with the outcome without adjustment for *U* was -0.010 (mean standard error 0.002), and the empirical Type 1 error rate was 97.9%. With

For peer review only - http://bmjopen.bmj.com/site/about/guidelines.xhtml

For peer review only

adjustment for *U*, the mean association was -0.030 (mean standard error 0.002), and the empirical Type 1 error rate was 100.0%.

In scenario 1, bias reduced considerably on adjustment for the factor influencing selection. Mean bias did not reduce to zero, as selection also depends on the exposure. We note that if selection did not additionally depend on the exposure, there would not be selection bias, as *U* is a confounder not a collider. However, even in this case with imperfect adjustment, the reduction in bias was substantial. In scenario 2, there was bias without adjustment for the collider *U*, but mean bias sharpened (got worse) on adjustment for *U*. Scenario 1 is more likely to be representative of the situation we see in the paper, as sex and age cannot be consequences of the exposure, but are likely to be confounders.

This simulation study is deliberately designed to be relatively simple and limited in scope, as we do not believe that any simulation study would be able to prove conclusively that covariate adjustment eliminates selection bias in all cases where selection depends on a confounder. Indeed, we do not make that claim; rather we claim that adjustment for confounders that are key predictors of selection can ameliorate bias due to selection. There will always be intrinsic uncertainty in practice, as we do not know all the factors that influence selection, and we would be cautious to adjust for too many variables in a Mendelian randomization analysis, particularly for variables that could be mediators on the causal pathway from the exposure to the outcome, or could be colliders. As age and sex cannot logically be caused by the exposure, they can be safely adjusted for without risk of these variables being mediators or colliders [S1]. Hence, if age and sex are strong predictors of selection into the sample, then adjustment for age and sex should reduce selection bias in Mendelian randomization investigations.

We believe that any residual bias from selection is likely to be low, in line with previous simulation studies that have shown selection effects need to be substantial in magnitude to have non-negligible biasing effects on Mendelian randomization estimates [S2]. However, we acknowledge that this imposes an additional level of uncertainty on the results of a non-linear Mendelian randomization analysis, on top of the existing uncertainty in the Mendelian randomization paradigm due to the validity of the instrumental variable assumptions, and other factors. All methods for making causal inferences from observational data rely on untestable assumptions, and findings should be interpreted with appropriate caution.

SUPPLEMENTARY REFERENCES

S1. Burgess S, Davey Smith G, Davies NM, Dudbridge F, Gill D, Glymour MM, Hartwig FP, Kutalik Z, Holmes MV, Minelli C, Morrison JV, *et al*. Guidelines for performing Mendelian randomization investigations: update for summer 2023. Wellcome Open Research 2023; 4:186.

S2. Gkatzionis A, Burgess S. Contextualizing selection bias in Mendelian randomization: how bad is it likely to be? International Journal of Epidemiology. 2019; 48(3):691-701.

For peer review only - http://bmjopen.bmj.com/site/about/guidelines.xhtml

Supplementary Table 1: Genetic variants and associations with BMI based on European sex-combined analysis from GIANT [23]. Beta-coefficents are in standard deviation units.

SNP	Effect allele*	Other allele	Beta-coefficient	Standard error of beta	Number of subjects	*P*-value
rs1558902	A	T	0.082	0.003	320 073	7.51E-153
rs6567160	C	T	0.056	0.004	321 958	3.93E-53
rs10938397	G	A	0.040	0.003	320 955	3.21E-38
rs543874	G	A	0.048	0.004	322 008	2.62E-35
rs2207139	G	A	0.045	0.004	322 019	4.13E-29
rs11030104	A	G	0.041	0.004	322 103	5.56E-28
rs3101336	C	T	0.033	0.003	316 872	2.66E-26
rs7138803	A	G	0.032	0.003	322 092	8.15E-24
rs10182181	G	A	0.031	0.003	321 759	8.78E-24
rs3888190	A	C	0.031	0.003	321 930	3.14E-23
rs1516725	C	T	0.045	0.005	320 644	1.89E-22
rs12446632	G	A	0.040	0.005	316 758	1.48E-18
rs2287019	C	T	0.036	0.004	300 921	4.59E-18
rs3817334	T	C	0.026	0.003	321 959	5.15E-17
rs2112347	T	G	0.026	0.003	322 019	6.19E-17
rs12566985	G	A	0.024	0.003	319 282	3.28E-15
rs3810291	A	G	0.028	0.004	296 261	4.81E-15
rs7141420	T	C	0.024	0.003	321 970	1.23E-14
rs13078960	G	T	0.030	0.004	322 135	1.74E-14
rs10968576	G	A	0.025	0.003	322 061	6.61E-14
rs17024393	C	T	0.066	0.009	297 874	7.03E-14
rs657452	A	G	0.023	0.003	313 651	5.48E-13
rs12429545	A	G	0.033	0.005	312 934	1.09E-12
rs12286929	G	A	0.022	0.003	321 903	1.31E-12
rs13107325	T	C	0.048	0.007	321 461	1.83E-12
rs11165643	T	C	0.022	0.003	320 730	2.07E-12
rs7903146	C	T	0.023	0.003	322 130	1.11E-11
rs10132280	C	A	0.023	0.003	321 797	1.14E-11
rs17405819	T	C	0.022	0.003	322 085	2.07E-11
rs1016287	T	C	0.023	0.003	321 969	2.25E-11
rs4256980	G	C	0.021	0.003	320 028	2.90E-11
rs17094222	C	T	0.025	0.004	321 770	5.94E-11
rs12401738	A	G	0.021	0.003	322 070	1.15E-10
rs7599312	G	A	0.022	0.003	322 024	1.17E-10
rs2365389	C	T	0.020	0.003	316 768	1.63E-10
rs205262	G	A	0.022	0.004	315 542	1.75E-10
rs2820292	C	A	0.020	0.003	321 707	1.83E-10
rs12885454	C	A	0.021	0.003	320 823	1.94E-10
rs16851483	T	G	0.048	0.008	233 929	3.55E-10
rs1167827	G	A	0.020	0.003	306 238	6.33E-10
rs758747	T	C	0.023	0.004	308 688	7.47E-10
rs1928295	T	C	0.019	0.003	321 979	7.91E-10
rs9925964	A	G	0.019	0.003	318 385	8.11E-10

For peer review only

rs11126666	A	G	0.021	0.003	321 979	1.33E-09
rs2650492	A	G	0.021	0.004	319 464	1.92E-09
rs6804842	G	A	0.019	0.003	321 463	2.48E-09
rs12940622	G	A	0.018	0.003	322 032	2.49E-09
rs11847697	T	C	0.049	0.008	306 243	3.99E-09
rs4740619	T	C	0.018	0.003	321 887	4.56E-09
rs13191362	A	G	0.028	0.005	321 902	7.34E-09
rs3736485	A	G	0.018	0.003	321 398	7.41E-09
rs17001654	G	C	0.031	0.005	233 722	7.76E-09
rs11191560	C	T	0.031	0.005	321 893	8.45E-09
rs1528435	T	C	0.018	0.003	321 924	1.20E-08
rs2075650	A	G	0.026	0.005	308 408	1.25E-08
rs1000940	G	A	0.019	0.003	321 836	1.28E-08
rs2033529	G	A	0.019	0.003	321 917	1.39E-08
rs11583200	C	T	0.018	0.003	322 095	1.48E-08
rs9400239	C	T	0.019	0.003	321 988	1.61E-08
rs10733682	A	G	0.017	0.003	320 727	1.83E-08
rs11688816	G	A	0.017	0.003	322 051	1.89E-08
rs11057405	G	A	0.031	0.006	314 111	2.02E-08
rs2121279	T	C	0.025	0.004	322 065	2.31E-08
rs29941	G	A	0.018	0.003	321 970	2.41E-08
rs11727676	T	C	0.036	0.006	296 401	2.55E-08
rs3849570	A	C	0.019	0.003	284 339	2.60E-08
rs6477694	C	T	0.017	0.003	322 048	2.67E-08
rs7899106	G	A	0.040	0.007	321 770	2.96E-08
rs2176598	T	C	0.020	0.004	316 848	2.97E-08
rs2245368	C	T	0.032	0.006	205 675	3.19E-08
rs17724992	A	G	0.019	0.004	319 588	3.42E-08
rs7243357	T	G	0.022	0.004	322 107	3.86E-08
rs1808579	C	T	0.017	0.003	322 032	4.17E-08

*BMI-increasing allele

For peer review only

Supplementary Table 2: ICD-10 codes for cause-specific mortality outcomes

Cause-specific mortality	ICD-10 codes
Cardiovascular	G45, I01, I03-I82, I87, I95-I99, F01, Q20-Q28, R96
Cancer	C00-C97, D00-D48
Non-cardiovascular non-cancer	All others

For peer review only

Supplementary Table 3: Distribution of BMI (kg/m^2) in strata defined by the doubly-ranked stratification method

	HUNT			UK Biobank		
Stratum number	Mean	Lower decile	Upper decile	Mean	Lower decile	Upper decile
1	18.4	17.5	19.8	18.6	17.8	20.0
2	19.3	18.2	20.4	19.6	18.5	20.7
3	19.9	18.8	20.8	20.1	19.1	21.1
4	20.3	19.4	21.1	20.5	19.6	21.5
5	20.6	19.7	21.5	20.9	20.0	21.8
6	20.9	20.1	21.7	21.1	20.3	22.0
7	21.1	20.3	21.9	21.4	20.6	22.3
8	21.3	20.5	22.1	21.6	20.8	22.5
9	21.5	20.7	22.3	21.8	21.0	22.7
10	21.7	20.9	22.5	22.0	21.2	22.8
11	21.8	21.1	22.7	22.2	21.4	23.0
12	22.0	21.2	22.8	22.4	21.6	23.2
13	22.1	21.4	22.9	22.5	21.7	23.3
14	22.3	21.5	23.1	22.7	21.9	23.5
15	22.4	21.7	23.2	22.8	22.0	23.7
16	22.5	21.8	23.3	23.0	22.2	23.8
17	22.7	21.9	23.5	23.1	22.3	23.9
18	22.8	22.0	23.6	23.2	22.4	24.1
19	22.9	22.1	23.7	23.4	22.6	24.2
20	23.0	22.2	23.8	23.5	22.7	24.3
21	23.1	22.4	23.9	23.6	22.8	24.4
22	23.2	22.5	24.0	23.7	22.9	24.5
23	23.3	22.6	24.1	23.8	23.0	24.7
24	23.4	22.7	24.2	24.0	23.2	24.8
25	23.6	22.8	24.3	24.1	23.3	24.9
26	23.6	22.9	24.5	24.2	23.4	25.0
27	23.8	23.0	24.5	24.3	23.5	25.2
28	23.8	23.1	24.7	24.4	23.6	25.3
29	23.9	23.1	24.8	24.5	23.7	25.4
30	24.0	23.2	24.8	24.6	23.8	25.5
31	24.1	23.3	25.0	24.7	23.9	25.6
32	24.2	23.4	25.0	24.8	24.0	25.7
33	24.3	23.5	25.1	24.9	24.1	25.8
34	24.4	23.6	25.2	25.0	24.2	25.9
35	24.5	23.7	25.3	25.1	24.3	26.0
36	24.6	23.8	25.4	25.2	24.4	26.1
37	24.7	23.8	25.5	25.3	24.5	26.2
38	24.8	23.9	25.6	25.4	24.6	26.3
39	24.8	24.0	25.7	25.5	24.7	26.4
40	24.9	24.1	25.8	25.6	24.8	26.6
41	25.0	24.2	25.9	25.7	24.9	26.7
42	25.1	24.3	26.0	25.8	25.0	26.8
43	25.2	24.3	26.1	25.9	25.1	26.9
44	25.3	24.4	26.2	26.0	25.1	27.0
45	25.4	24.5	26.3	26.1	25.3	27.1

For peer review only

46	25.5	24.6	26.4	26.2	25.4	27.2
47	25.6	24.7	26.4	26.3	25.4	27.3
48	25.6	24.8	26.6	26.5	25.5	27.4
49	25.7	24.8	26.7	26.6	25.6	27.5
50	25.8	25.0	26.8	26.7	25.7	27.6
51	25.9	25.0	26.9	26.8	25.8	27.7
52	26.0	25.1	26.9	26.9	25.9	27.8
53	26.1	25.2	27.0	27.0	26.0	28.0
54	26.2	25.3	27.1	27.1	26.1	28.1
55	26.3	25.4	27.2	27.2	26.2	28.2
56	26.4	25.5	27.3	27.3	26.3	28.3
57	26.5	25.5	27.4	27.4	26.4	28.4
58	26.6	25.6	27.5	27.5	26.5	28.6
59	26.7	25.7	27.6	27.6	26.6	28.7
60	26.8	25.8	27.7	27.7	26.7	28.8
61	26.9	25.9	27.9	27.9	26.8	28.9
62	27.0	26.0	27.9	28.0	26.9	29.1
63	27.1	26.1	28.1	28.1	27.0	29.2
64	27.2	26.2	28.2	28.2	27.1	29.3
65	27.3	26.3	28.3	28.3	27.2	29.5
66	27.4	26.4	28.4	28.5	27.3	29.6
67	27.5	26.5	28.6	28.6	27.5	29.8
68	27.6	26.6	28.7	28.7	27.6	29.9
69	27.7	26.7	28.8	28.9	27.7	30.0
70	27.9	26.8	28.9	29.0	27.8	30.2
71	28.0	26.9	29.0	29.1	28.0	30.4
72	28.1	27.0	29.1	29.3	28.1	30.5
73	28.2	27.1	29.4	29.4	28.2	30.7
74	28.3	27.2	29.5	29.6	28.4	30.9
75	28.5	27.4	29.6	29.8	28.5	31.1
76	28.6	27.5	29.8	29.9	28.7	31.2
77	28.7	27.6	29.9	30.1	28.8	31.4
78	28.9	27.8	30.1	30.3	28.9	31.6
79	29.0	27.9	30.3	30.4	29.1	31.8
80	29.2	28.0	30.5	30.6	29.3	32.1
81	29.4	28.2	30.7	30.8	29.4	32.3
82	29.5	28.3	30.9	31.0	29.6	32.6
83	29.7	28.4	31.1	31.3	29.8	32.8
84	29.9	28.6	31.3	31.5	30.0	33.0
85	30.1	28.8	31.5	31.7	30.2	33.3
86	30.3	28.9	31.8	32.0	30.4	33.7
87	30.6	29.1	32.0	32.3	30.6	34.0
88	30.8	29.3	32.3	32.5	30.9	34.4
89	31.0	29.5	32.6	32.9	31.1	34.7
90	31.3	29.7	33.0	33.2	31.4	35.2
91	31.6	30.0	33.3	33.6	31.7	35.6
92	32.0	30.3	33.7	34.0	32.0	36.2
93	32.3	30.6	34.2	34.5	32.4	36.7
94	32.8	30.9	34.8	35.0	32.8	37.4
95	33.3	31.4	35.5	35.7	33.3	38.2
96	33.9	31.8	36.1	36.4	33.9	39.2

For peer review only

For peer review only - http://bmjopen.bmj.com/site/about/guidelines.xhtml

97	34.7	32.4	37.1	37.4	34.6	40.5
98	35.8	33.3	38.7	38.7	35.5	42.2
99	37.3	34.1	40.5	40.5	36.7	44.8
100	40.3	35.6	43.2	44.3	38.8	47.9

We report the mean, lower decile (10th percentile of the distribution), and upper decile (90th percentile of the distribution) of the BMI distribution for participants in each stratum created by the doubly-ranked method. In the first and 100th strata, we instead report the 20th and 80th percentiles of the distribution.

For peer review only

Supplementary Figure 1: Associations between the genetic score and BMI in strata defined by the doubly-ranked method plotted against mean BMI in each stratum for: A) HUNT, B) UK Biobank. Error bars represent 95% confidence intervals.

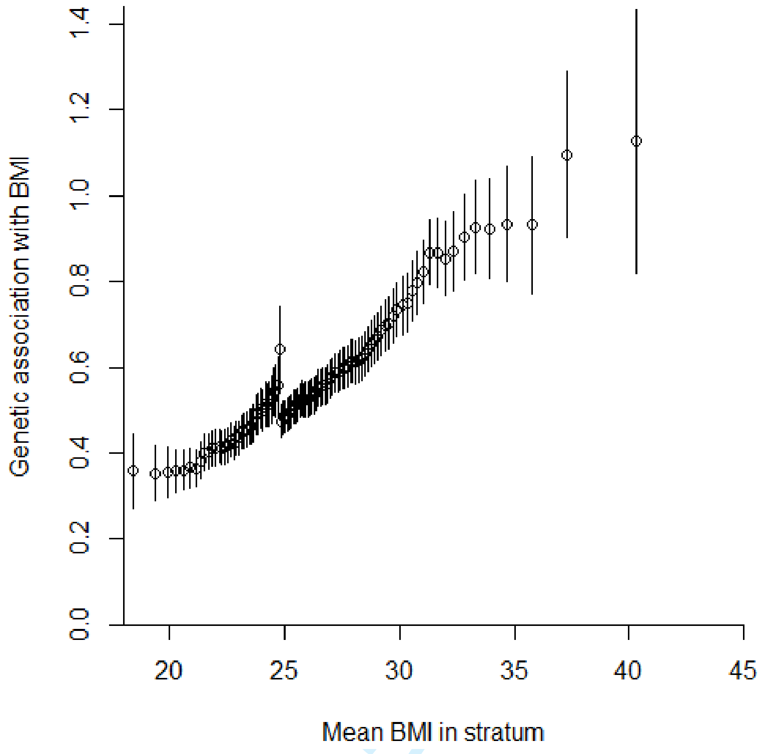

A.

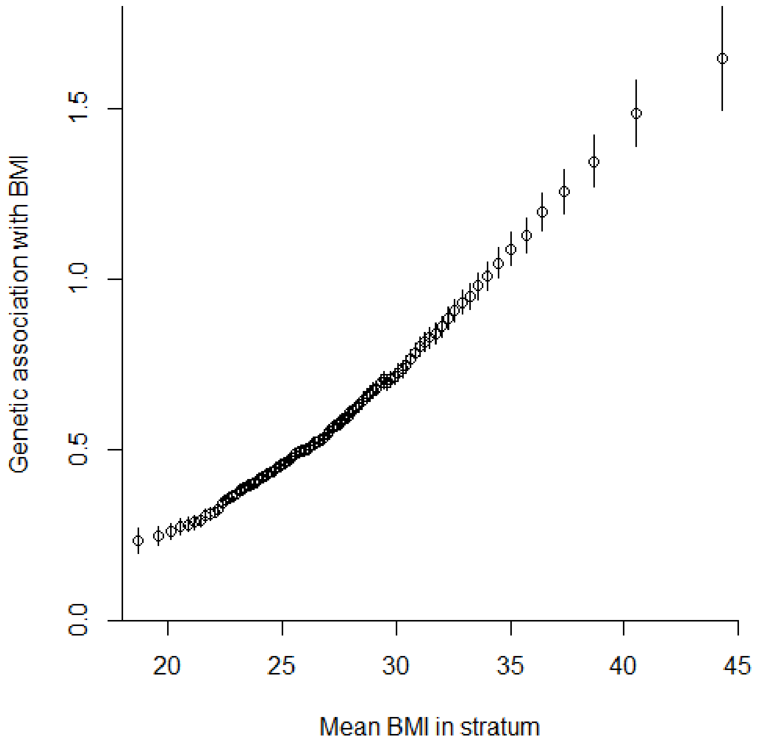

B.

Supplementary Figure 2: Genetic associations with potential competing risk factors in strata of the HUNT study population

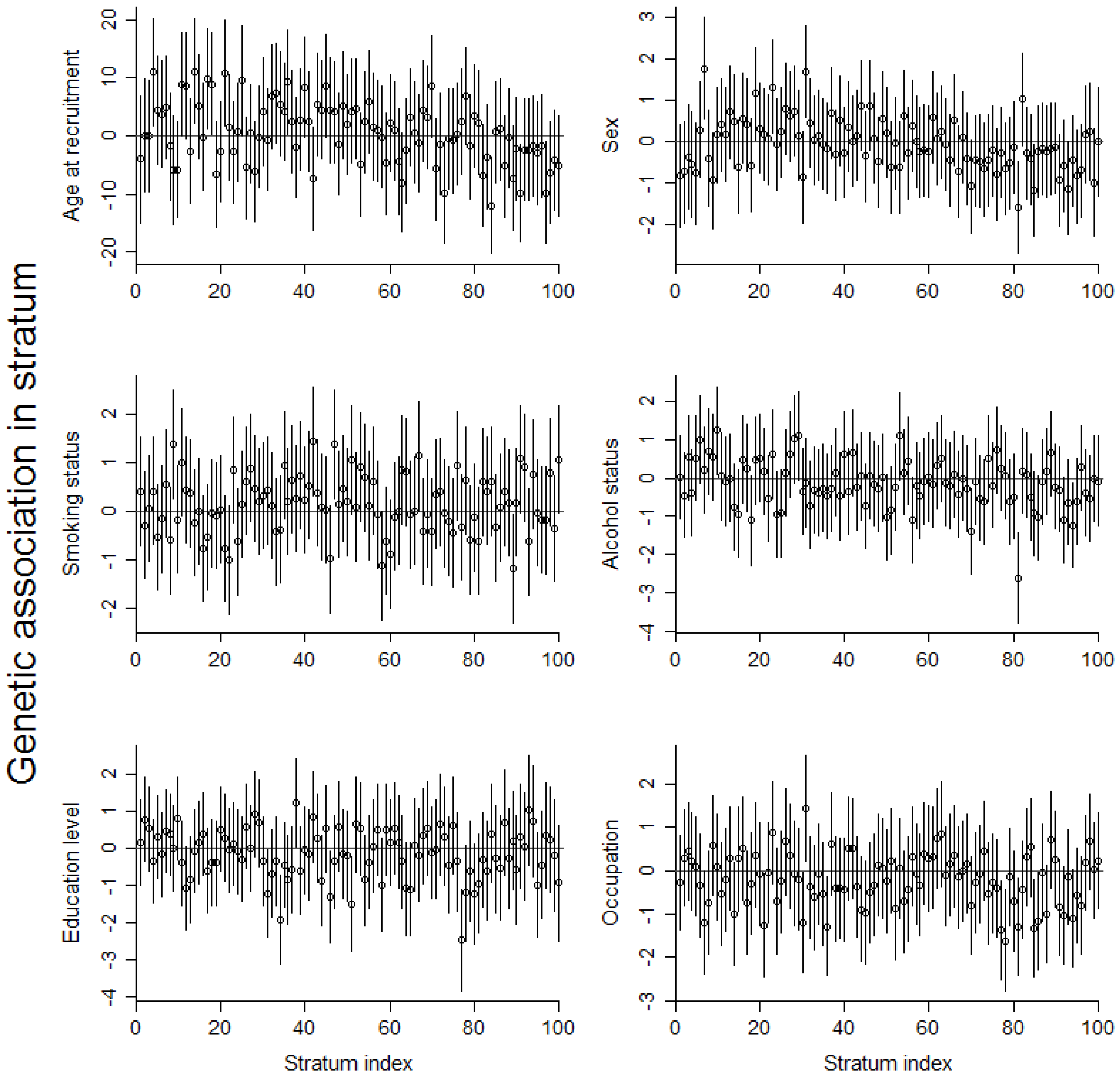

Points represent association estimates from linear (age at recruitment, years) or logistic regression (all other traits). Error bars represent 95% confidence intervals. Stratum 1 has lowest average BMI levels, stratum 100 has highest average BMI levels.

For peer review only - http://bmjopen.bmj.com/site/about/guidelines.xhtml

Supplementary Figure 3: Localized average causal effect estimates (stratum-specific estimates) for all-cause mortality plotted against mean BMI in each stratum. Estimates represent hazard ratios per 1 kg/m^2 increase in genetically-predicted BMI. Error bars represent 95% confidence intervals.

A) HUNT, doubly-ranked method:

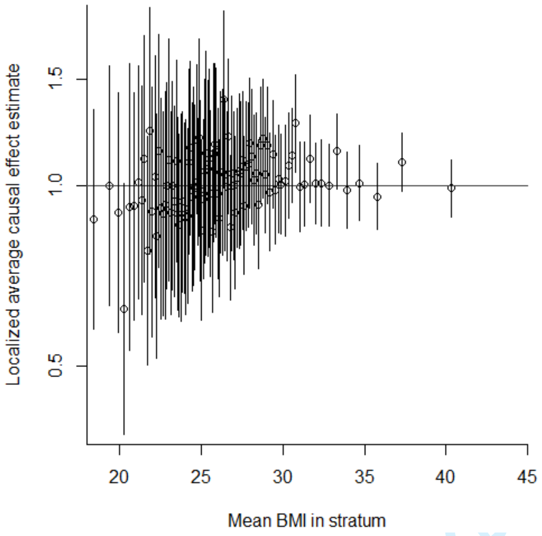

B) UK Biobank, doubly-ranked method:

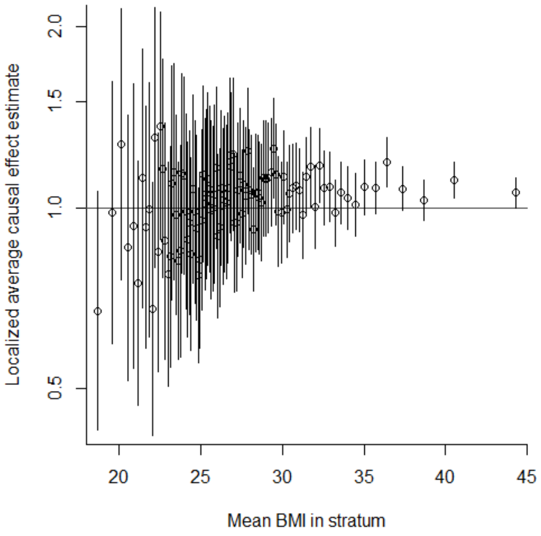

C) HUNT, residual method:

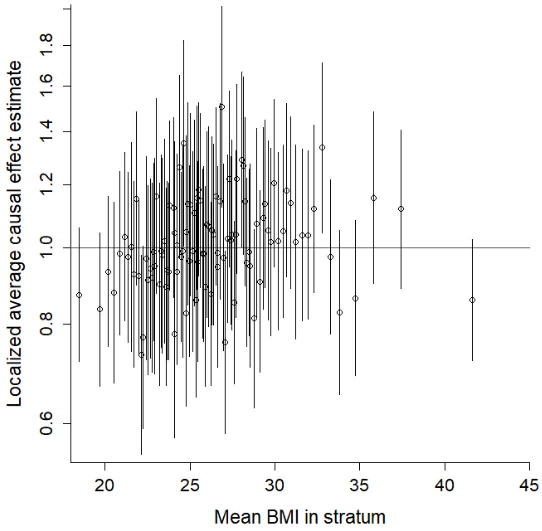

D) UK Biobank, residual method:

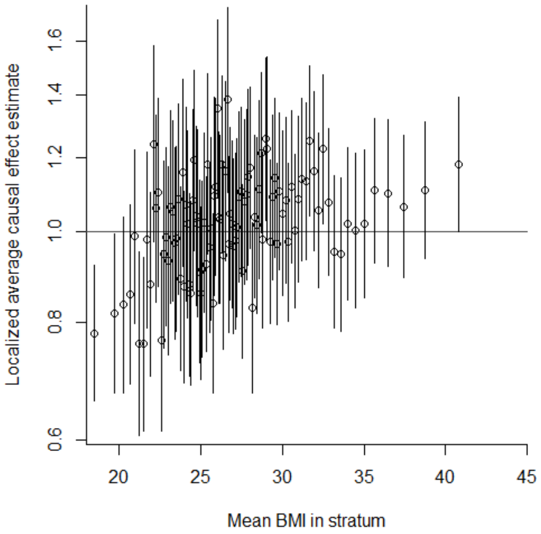

This presentation does not smooth over stratum-specific estimates, hence providing a representation of the causal effect of BMI which is not dependent on the choice of fractional polynomial smoothing. However, stratum-specific estimates are imprecise and variable, as they each only use 1% of the overall sample size. These plots differ from the presentation in the main text Figures: here, we plot the stratum-specific Mendelian randomization estimates, which reflect the slope of the curves in the main text figures.

Supplementary Figure 4: Non-linear Mendelian randomization – dose-response curve between body mass index and all-cause mortality using the doubly-ranked method with inverse-normal rank transformed BMI values in UK Biobank. Grey lines represent 95% confidence intervals. The reference value for BMI was taken as the median of the BMI distribution at 26.7 kg/m^2.

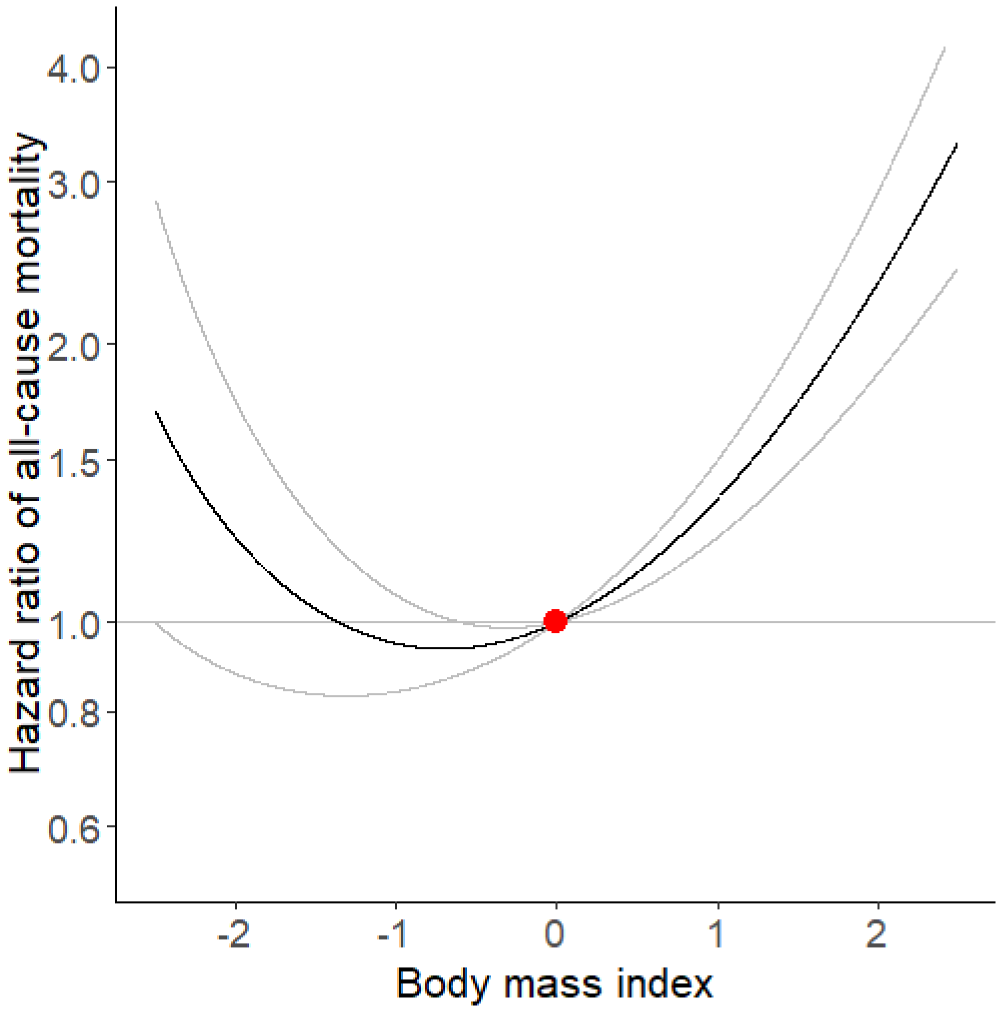

The labels on the horizontal axis represent the number of standard deviations below or above the median of the BMI distribution: for example, -1 represents one standard deviation below the median, and +2 represents two standard deviations above the median.

The reference values are:
-2 = 19.9 kg/m^2
-1 = 23.0 kg/m^2
0 = 26.7 kg/m^2
+1 = 31.6 kg/m^2
+2 = 39.2 kg/m^2

For peer review only - http://bmjopen.bmj.com/site/about/guidelines.xhtml

Supplementary Figure 5: Non-linear Mendelian randomization – dose-response curve between body mass index and all-cause mortality estimated using the doubly-ranked method: A) HUNT, B) UK Biobank dividing into 50 strata; C) HUNT, D) UK Biobank dividing into 200 strata. Gradient at each point of the curve is the localized average causal effect. Grey lines represent 95% confidence intervals. The reference value for BMI was taken as 25 kg/m^2.

A) HUNT, 50 strata:

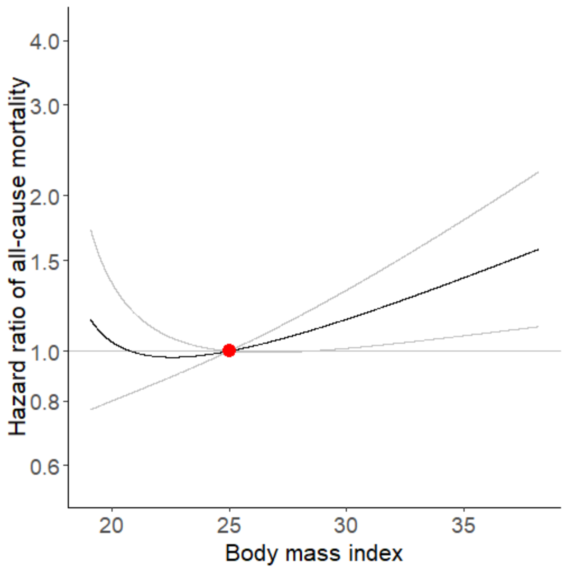

p-value for linearity: 0.50
p-value for trend: 0.49

B) UK Biobank, 50 strata:

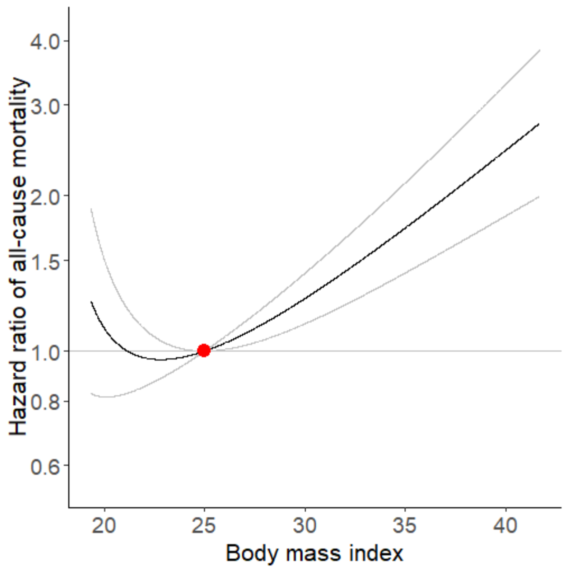

p-value for linearity: 0.10
p-value for trend: 0.09

C) HUNT, 200 strata:

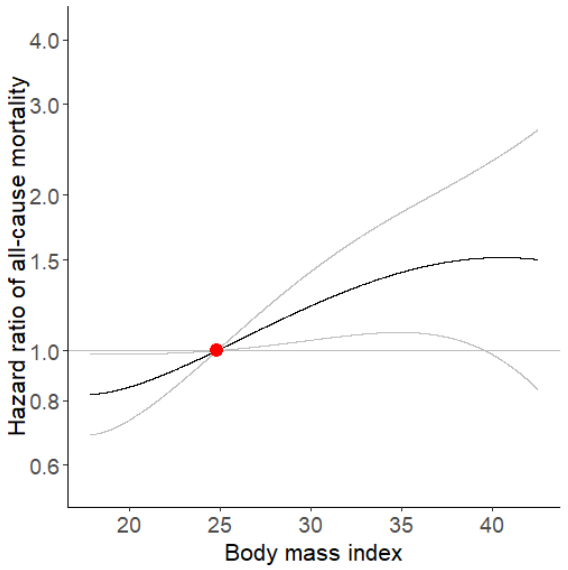

p-value for linearity: 0.99
p-value for trend: 0.88

D) UK Biobank, 200 strata:

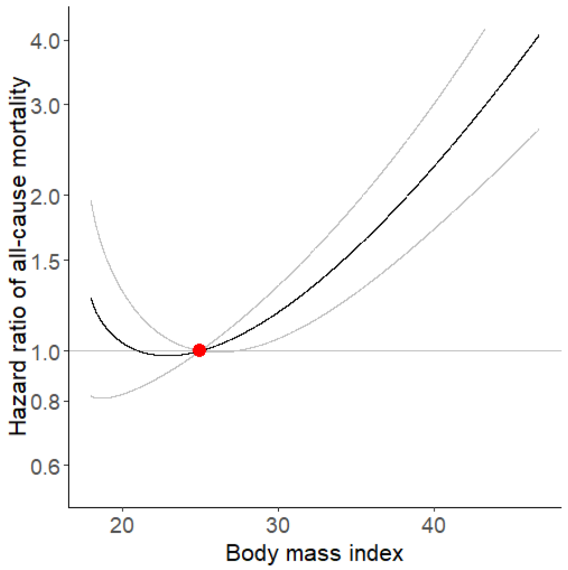

p-value for linearity: 0.28
p-value for trend: 0.16

For peer review only - http://bmjopen.bmj.com/site/about/guidelines.xhtml

Supplementary Figure 6: Non-linear Mendelian randomization – dose-response curve between body mass index and all-cause mortality estimated using the doubly-ranked method and the extended genetic score in UK Biobank. Gradient at each point of the curve is the localized average causal effect. Grey lines represent 95% confidence intervals. The reference value for BMI was taken as 25 kg/m^2.

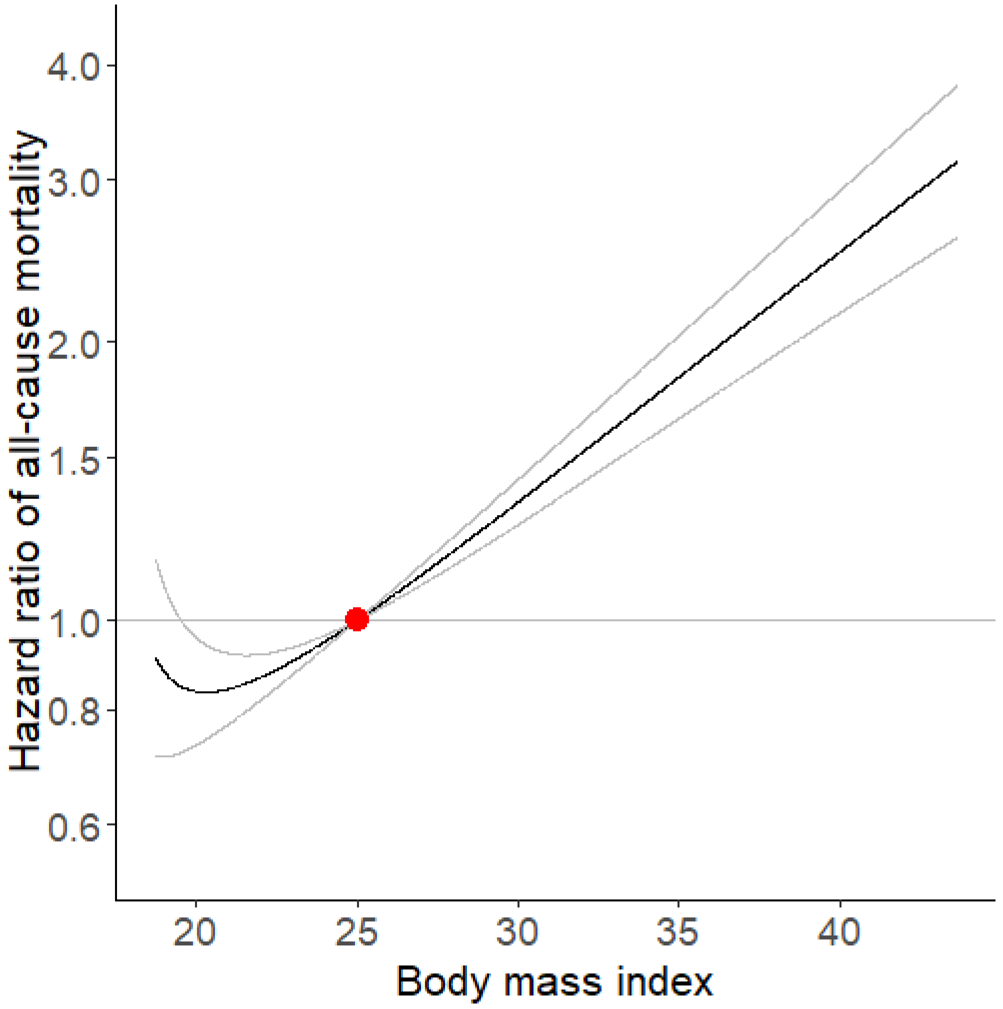

p-value for linearity: 0.99
p-value for trend: 0.32

Supplementary Figure 7: Localized average causal effect estimates (stratum-specific estimates) for all-cause mortality plotted against mean BMI in each stratum estimated in men and women separately. Estimates represent hazard ratios per 1 kg/m^2 increase in genetically-predicted BMI. Error bars represent 95% confidence intervals.

A) HUNT, men only:

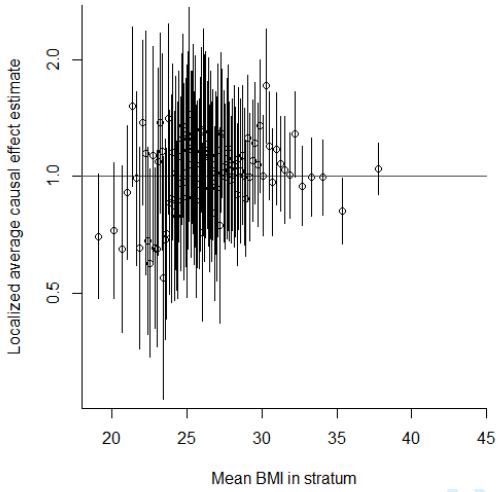

B) HUNT, women only:

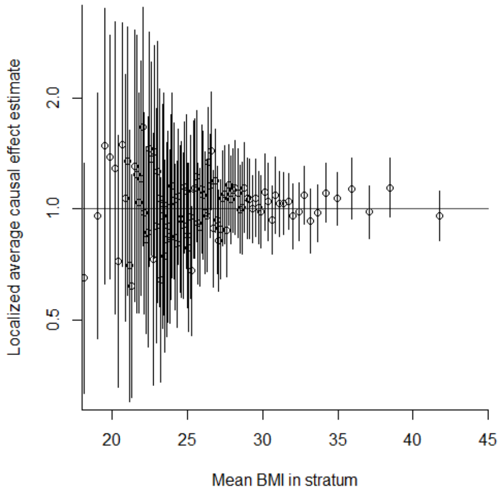

C) UK Biobank, men only:

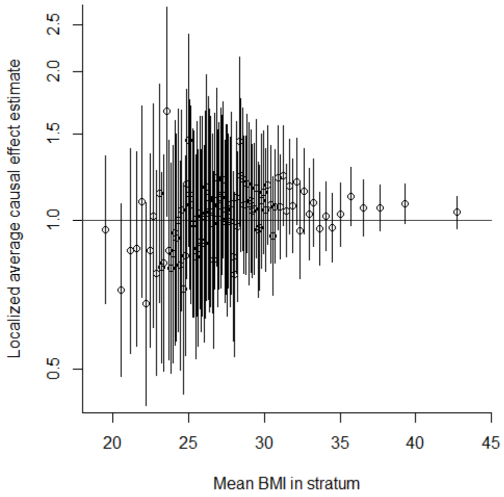

D) UK Biobank, women only:

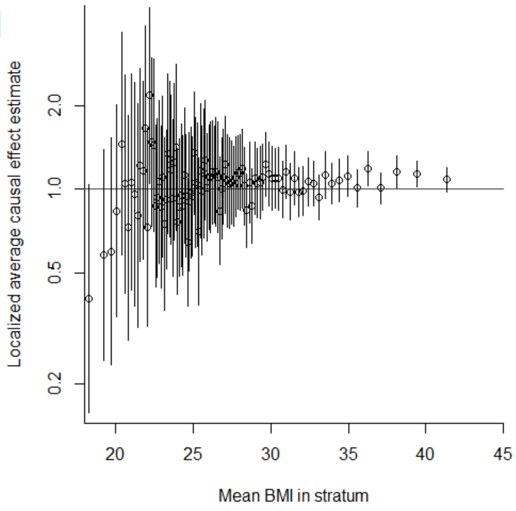

For peer review only - http://bmjopen.bmj.com/site/about/guidelines.xhtml

Supplementary Figure 8: Localized average causal effect estimates (stratum-specific estimates) for cause-specific mortality plotted against mean BMI in each stratum. Estimates represent hazard ratios per 1 kg/m^2 increase in genetically-predicted BMI. Error bars represent 95% confidence intervals.

A: Cardiovascular mortality

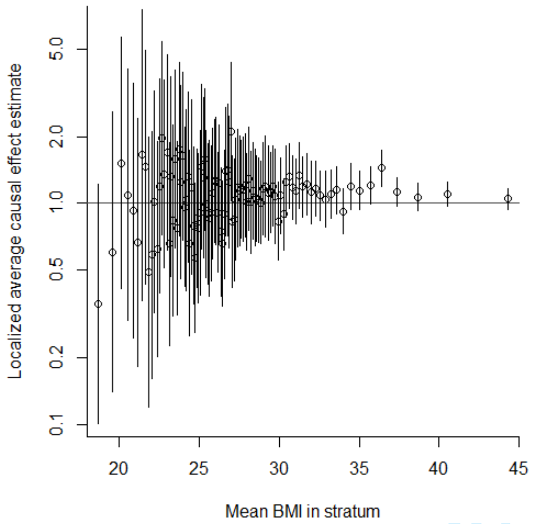

B: Cancer mortality

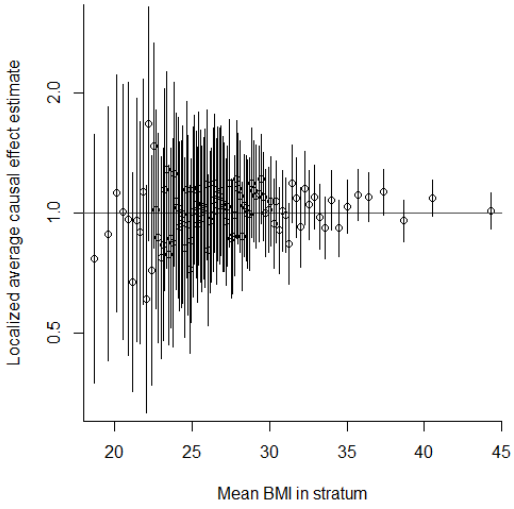

C: Other (non-cardiovascular, non-cancer) mortality

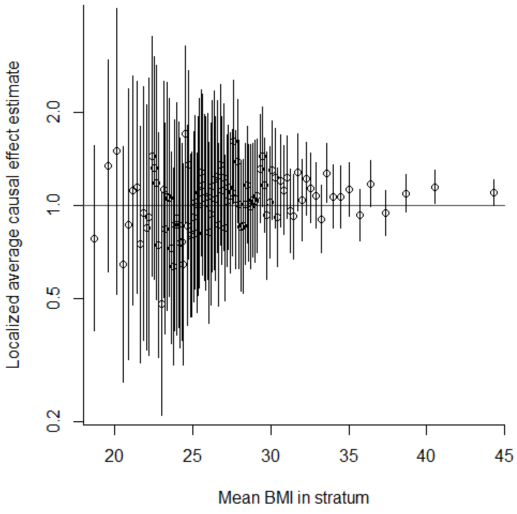

For peer review only - http://bmjopen.bmj.com/site/about/guidelines.xhtml

STROBE-MR checklist of recommended items to address in reports of Mendelian randomization studies[1 2]

Item No.	Section	Checklist item	Page No.	Relevant text from manuscript
1	**TITLE and ABSTRACT**	Indicate Mendelian randomization (MR) as the study's design in the title and/or the abstract if that is a main purpose of the study	1-2	Title: "Body mass index and all-cause mortality in HUNT and UK Biobank studies: revised non-linear Mendelian randomization analyses"<br>Abstract: "**Objectives**: To estimate the shape of the causal relationship between body mass index (BMI) and mortality risk in a Mendelian randomization framework."
	**INTRODUCTION**			
2	**Background**	Explain the scientific background and rationale for the reported study. What is the exposure? Is a potential causal relationship between exposure and outcome plausible? Justify why MR is a helpful method to address the study question	2,4	Abstract: "To estimate the shape of the causal relationship between body mass index (BMI) and mortality risk in a Mendelian randomization framework."<br>Introduction: "The epidemiological relationship between BMI and mortality is complex, with many observational studies conducted in Western countries showing a J-shaped relationship between BMI and mortality risk."<br>"However, such findings may not reflect the causal relationship between BMI and mortality, as observation association are influenced by confounding and reverse causation."
3	**Objectives**	State specific objectives clearly, including pre-specified causal hypotheses (if any). State that MR is a method that, under specific assumptions, intends to estimate causal effects	2,4	Abstract: "To estimate the shape of the causal relationship between body mass index (BMI) and mortality risk in a Mendelian randomization framework."<br>Introduction: "We first investigate whether the constant genetic effect assumption holds for this example, and explore the validity of the instrumental variable assumptions in strata of the population. We then present non-linear Mendelian randomization findings from the doubly-ranked stratification method."<br>"Hence, associations between genetic predictors of BMI and mortality provide insights into the causal effect of BMI on mortality."

For peer review only - http://bmjopen.bmj.com/site/about/guidelines.xhtml

	**METHODS**				
4	**Study design and data sources**		Present key elements of the study design early in the article. Consider including a table listing sources of data for all phases of the study. For each data source contributing to the analysis, describe the following:		
		a)	Setting: Describe the study design and the underlying population, if possible. Describe the setting, locations, and relevant dates, including periods of recruitment, exposure, follow-up, and data collection, when available.	6	See sections "The HUNT study" and "The UK Biobank study" (some of this detail is in reference 19, as indicated: "We present abbreviated descriptions of the datasets included in the analysis; detailed descriptions are in the original paper [19]").
		b)	Participants: Give the eligibility criteria, and the sources and methods of selection of participants. Report the sample size, and whether any power or sample size calculations were carried out prior to the main analysis	6	See sections "The HUNT study" and "The UK Biobank study" (detail is in reference 19: "We present abbreviated descriptions of the datasets included in the analysis; detailed descriptions are in the original paper [19]").
		c)	Describe measurement, quality control and selection of genetic variants	6	See section "SNPs and genetic score used as instrumental variables"
		d)	For each exposure, outcome, and other relevant variables, describe methods of assessment and diagnostic criteria for diseases	NA	See reference 19 for detail.
		e)	Provide details of ethics committee approval and participant informed consent, if relevant	7	See section "Ethical Approval" and "Patient Involvement"
5	**Assumptions**		Explicitly state the three core IV assumptions for the main analysis (relevance, independence and exclusion restriction) as well assumptions for any additional or sensitivity analysis	4,6	Introduction: "According to Mendel's laws of inheritance, genetic variants should be uncorrelated with traits that they do not affect, and hence should be independent of potential confounders"<br>Methods: "We assessed the instrumental variable assumptions by estimating associations between the genetic score and various traits that are competing risk factors in UK Biobank: smoking status (ever versus never), alcohol status (current versus other), education level (post-secondary or higher versus other), occupation (currently employed versus other), age at recruitment, and sex."
6	**Statistical methods: main analysis**		Describe statistical methods and statistics used	6-7	See Section "Statistical analyses".

For peer review only - http://bmjopen.bmj.com/site/about/guidelines.xhtml

		a)	Describe how quantitative variables were handled in the analyses (i.e., scale, units, model)	7	See Section "Statistical analyses".
		b)	Describe how genetic variants were handled in the analyses and, if applicable, how their weights were selected	6	See Section "SNPs and genetic score used as instrumental variables"
		c)	Describe the MR estimator (e.g. two-stage least squares, Wald ratio) and related statistics. Detail the included covariates and, in case of two-sample MR, whether the same covariate set was used for adjustment in the two samples	7	See Section "Statistical analyses":<br>"We then calculated the linear Mendelian randomization estimate, referred to as a localized average causal effect (LACE), in each stratum of the population as a ratio of coefficients: the association of the genetic score with the outcome divided by the associations of the genetic score with the exposure. associations with the exposure (BMI) were obtained from linear regression; associations with the outcome (mortality) were obtained from Cox proportional hazards regression, using age as the timescale. All associations were adjusted for age, age-squared, sex, and centre (for UK Biobank)."
		d)	Explain how missing data were addressed	6	"We excluded participants without data on BMI or genetic variants for BMI, leaving 56,150 individuals for analysis."
		e)	If applicable, indicate how multiple testing was addressed	N/A	We did not account for multiple testing, as the presentation of p-values is not a major aspect of the manuscript.
7	**Assessment of assumptions**		Describe any methods or prior knowledge used to assess the assumptions or justify their validity	6	See Section "SNPs and genetic score used as instrumental variables":<br>"We assessed the instrumental variable assumptions by estimating associations between the genetic score and various traits that are competing risk factors in UK Biobank: smoking status (ever versus never), alcohol status (current versus other), education level (post-secondary versus other), occupations (currently employed versus other), age at recruitment, and sex.
8	**Sensitivity analyses and additional analyses**		Describe any sensitivity analyses or additional analyses performed (e.g. comparison of effect estimates from different approaches, independent replication, bias analytic techniques, validation of instruments, simulations)	7	Sensitivity Analyses: "In brief, first we divided the sample into 100 strata using the doubly-ranked method. For comparison, we also present results from stratification using the residual method for the primary outcome of all-cause mortality."<br>Additional analyses: Testing for linearity:

For peer review only - http://bmjopen.bmj.com/site/about/guidelines.xhtml

					"We report two tests for non-linearity: a linearity test, which assesses whether a non-linear model fits the LACE estimates better than a linear model, and a trend test, which tests for a linear trend amongst the LACE estimates. "
9	**Software and pre-registration**				
		a)	Name statistical software and package(s), including version and settings used	7	"All statistical analyses were performed in R (version 4.3.1), and non-linear Mendelian randomisation analyses were performed using the SUMnlmr package [25]."
		b)	State whether the study protocol and details were pre-registered (as well as when and where)	N/A	The study protocol was not pre-registered.
	**RESULTS**				
10	**Descriptive data**				
		a)	Report the numbers of individuals at each stage of included studies and reasons for exclusion. Consider use of a flow diagram		All numbers provided in Table 1.
		b)	Report summary statistics for phenotypic exposure(s), outcome(s), and other relevant variables (e.g. means, SDs, proportions)		All numbers provided in Table 1.
		c)	If the data sources include meta-analyses of previous studies, provide the assessments of heterogeneity across these studies		N/A – we present results from the HUNT and UK Biobank studies separately, and do not attempt to meta-analyse or otherwise combine results.
		d)	For two-sample MR:<br>i. Provide justification of the similarity of the genetic variant-exposure associations between the exposure and outcome samples<br>ii. Provide information on the number of individuals who overlap between the exposure and outcome studies		N/A – not two-sample MR.
11	**Main results**				
		a)	Report the associations between genetic variant and exposure, and between genetic variant and outcome, preferably on an interpretable scale		Genetic associations with exposure are shown in Supplementary Figure 1.<br>Scaled genetic association with outcome are shown in Supplementary Figures 3, 5, and 6.
		b)	Report MR estimates of the relationship between exposure and outcome, and the measures of uncertainty from the MR analysis, on an interpretable scale, such as odds ratio or relative risk per SD difference		MR estimates are shown directly in Supplementary Figures 3, 5, and 6. Smoothed versions of these associations are shown in Figures 2, 3, and 4.

For peer review only - http://bmjopen.bmj.com/site/about/guidelines.xhtml

		c)	If relevant, consider translating estimates of relative risk into absolute risk for a meaningful time period		N/A
		d)	Consider plots to visualize results (e.g. forest plot, scatterplot of associations between genetic variants and outcome versus between genetic variants and exposure)		N/A
12	**Assessment of assumptions**				
		a)	Report the assessment of the validity of the assumptions	8	See section "Assessment of instrument validity"
		b)	Report any additional statistics (e.g., assessments of heterogeneity across genetic variants, such as I^2, Q statistic or E-value)	6	F statistics are provided in the Methods section under "SNPs and genetic score used as instrumental variables.<br>Nonlinear tests are reported in Figures 2, 3, and 4.
13	**Sensitivity analyses and additional analyses**				
		a)	Report any sensitivity analyses to assess the robustness of the main results to violations of the assumptions		See Figure 2: residual versus ranked stratification.
		b)	Report results from other sensitivity analyses or additional analyses		Figure 2: residual versus ranked stratification<br>Figure 3: results stratified by sex<br>Figure 4: exploring different cause-specific mortality subtypes.
		c)	Report any assessment of direction of causal relationship (e.g., bidirectional MR)		N/A, directionality not considered.
		d)	When relevant, report and compare with estimates from non-MR analyses	11	In Discussion, paragraph beginning "Previous investigations into the non-linear shape of the causal relationship between BMI and mortality have used a variety of approaches." and in particular "Carslake *et al* performed an instrumental variable analysis to investigate the impact of BMI on mortality in the HUNT study, but using offspring BMI as an instrument for the BMI".
		e)	Consider additional plots to visualize results (e.g., leave-one-out analyses)		N/A
	**DISCUSSION**				
14	**Key results**		Summarize key results with reference to study objectives	10	Results are summarized in the Discussion section:

For peer review only - http://bmjopen.bmj.com/site/about/guidelines.xhtml

					"While the curves generally display a J-shaped relationship, evidence for a harmful average effect of decreased BMI on mortality at low BMI levels was limited. Evidence supporting non-linearity from the doubly-ranked method was unconvincing for all mortality outcomes."<br>Also see Abstract / Results.
15	**Limitations**		Discuss limitations of the study, taking into account the validity of the IV assumptions, other sources of potential bias, and imprecision. Discuss both direction and magnitude of any potential bias and any efforts to address them	11-12	Limitations are clearly discussed in the Discussion section, paragraph beginning "However, there are important limitations…" Several potential limitations are discussed, including validity of instruments, pleiotropy, bias due to selection bias, representativeness, and reverse causation.
16	**Interpretation**				
		a)	Meaning: Give a cautious overall interpretation of results in the context of their limitations and in comparison with other studies	10	Interpretation of results is provided in the Discussion section: "In conclusion, non-linear Mendelian randomization analyses using the doubly-ranked stratification method provide strong evidence for harmful effects of increased BMI on mortality above 25 kg/m2. Evidence for a harmful effect of low BMI was only present in some analyses, and where present, only below 20 kg/m2.".<br>Also see Abstract/Conclusion.
		b)	Mechanism: Discuss underlying biological mechanisms that could drive a potential causal relationship between the investigated exposure and the outcome, and whether the gene-environment equivalence assumption is reasonable. Use causal language carefully, clarifying that IV estimates may provide causal effects only under certain assumptions		N/A (mechanisms linking BMI and mortality have been discussed elsewhere at length, and evidence for specific mechanisms is not provided here).
		c)	Clinical relevance: Discuss whether the results have clinical or public policy relevance, and to what extent they inform effect sizes of possible interventions	10	Discussion: "Our findings challenge the "obesity paradox" consensus from observational epidemiology that increases in BMI might reduce risk of mortality for a substantial proportion of the population."
17	**Generalizability**		Discuss the generalizability of the study results (a) to other populations, (b) across other exposure periods/timings, and (c) across other levels of exposure	11	Discussion / Limitations: "Our findings may therefore not be applicable to other populations. "

For peer review only - http://bmjopen.bmj.com/site/about/guidelines.xhtml

	**OTHER INFORMATION**			
18	**Funding**	Describe sources of funding and the role of funders in the present study and, if applicable, sources of funding for the databases and original study or studies on which the present study is based	1	See “Funding statement”
19	**Data and data sharing**	Provide the data used to perform all analyses or report where and how the data can be accessed, and reference these sources in the article. Provide the statistical code needed to reproduce the results in the article, or report whether the code is publicly accessible and if so, where	31	See “Data sharing statement”
20	**Conflicts of Interest**	All authors should declare all potential conflicts of interest	1	Provided under “Competing interest statement”

This checklist is copyrighted by the Equator Network under the Creative Commons Attribution 3.0 Unported (CC BY 3.0) license.

1. Skrivankova VW, Richmond RC, Woolf BAR, Yarmolinsky J, Davies NM, Swanson SA, et al. Strengthening the Reporting of Observational Studies in Epidemiology using Mendelian Randomization (STROBE-MR) Statement. JAMA. 2021;under review.

2. Skrivankova VW, Richmond RC, Woolf BAR, Davies NM, Swanson SA, VanderWeele TJ, et al. Strengthening the Reporting of Observational Studies in Epidemiology using Mendelian Randomisation (STROBE-MR): Explanation and Elaboration. BMJ. 2021;375:n2233.

For peer review only - http://bmjopen.bmj.com/site/about/guidelines.xhtml

For peer review only

Body mass index and all-cause mortality in HUNT and UK Biobank studies: revised non-linear Mendelian randomization analyses

Journal:	*BMJ Open*
Manuscript ID	bmjopen-2023-081399.R2
Article Type:	Original research
Date Submitted by the Author:	11-Mar-2024

Complete List of Authors:	Burgess, Stephen; University of Cambridge, MRC Biostatistics Unit; University of Cambridge, Cardiovascular Epidemiology Unit, Department of Public Health and Primary Care<br>Sun, Yi-Qian; Norges teknisk-naturvitenskapelige universitet, Department of Clinical and Molecular Medicine (IKOM)<br>Zhou, Ang; University of Cambridge, MRC Biostatistics Unit; University of South Australia, Australian Centre for Precision Health<br>Buck, Christopher; University of Cambridge, MRC Biostatistics Unit<br>Mason, Amy M; University of Cambridge, Department of Public Health and Primary Care<br>Mai, Xiao-Mei; Norwegian University of Science and Technology, Department of Public Health and Nursing

**Primary Subject Heading**:	Public health
Secondary Subject Heading:	Epidemiology, Genetics and genomics, Public health
Keywords:	Obesity, Observational Study, GENETICS, EPIDEMIOLOGY

BMJ

I, the Submitting Author has the right to grant and does grant on behalf of all authors of the Work (as defined in the below author licence), an exclusive licence and/or a non-exclusive licence for contributions from authors who are: i) UK Crown employees; ii) where BMJ has agreed a CC-BY licence shall apply, and/or iii) in accordance with the terms applicable for US Federal Government officers or employees acting as part of their official duties; on a worldwide, perpetual, irrevocable, royalty-free basis to BMJ Publishing Group Ltd ("BMJ") its licensees and where the relevant Journal is co-owned by BMJ to the co-owners of the Journal, to publish the Work in this journal and any other BMJ products and to exploit all rights, as set out in our licence.

The Submitting Author accepts and understands that any supply made under these terms is made by BMJ to the Submitting Author unless you are acting as an employee on behalf of your employer or a postgraduate student of an affiliated institution which is paying any applicable article publishing charge ("APC") for Open Access articles. Where the Submitting Author wishes to make the Work available on an Open Access basis (and intends to pay the relevant APC), the terms of reuse of such Open Access shall be governed by a Creative Commons licence – details of these licences and which Creative Commons licence will apply to this Work are set out in our licence referred to above.

Other than as permitted in any relevant BMJ Author's Self Archiving Policies, I confirm this Work has not been accepted for publication elsewhere, is not being considered for publication elsewhere and does not duplicate material already published. I confirm all authors consent to publication of this Work and authorise the granting of this licence.

For peer review only

Body mass index and all-cause mortality in HUNT and UK Biobank studies: revised non-linear Mendelian randomization analyses

Stephen Burgess[1,2,3], Yi-Qian Sun[4,5,6], Ang Zhou[1,7], Christopher Buck[1], Amy M Mason[2,3], Xiao-Mei Mai[8]

1. Medical Research Council Biostatistics Unit, University of Cambridge, Cambridge, UK.
2. Cardiovascular Epidemiology Unit, Department of Public Health and Primary Care, University of Cambridge, Cambridge, UK.
3. Victor Phillip Dahdaleh Heart and Lung Research Institute, University of Cambridge, Cambridge, UK.
4. Department of Clinical and Molecular Medicine, Faculty of Medicine and Health Science, Norwegian University of Science and Technology, Trondheim, Norway.
5. Department of Pathology, Clinic of Laboratory Medicine, St. Olavs Hospital, Trondheim University Hospital, Trondheim, Norway.
6. Center for Oral Health Services and Research Mid-Norway (TkMidt), Trondheim, Norway.
7. Australian Centre for Precision Health, Unit of Clinical and Health Sciences, University of South Australia, Adelaide, Australia.
8. Department of Public Health and Nursing, Faculty of Medicine and Health Science, Norwegian University of Science and Technology, Trondheim, Norway.

Corresponding author: Stephen Burgess (sb452@medschl.cam.ac.uk)

Word count: 299 (abstract), 4357 (main text).

Key words: Obesity paradox, reverse causation, genetic epidemiology, causal inference, doubly-ranked stratification.

For peer review only

ABSTRACT

Objectives: To estimate the shape of the causal relationship between body mass index (BMI) and mortality risk in a Mendelian randomization framework.

Design: Mendelian randomization analyses of two prospective population-based cohorts.

Setting: Individuals of European ancestries living in Norway or the United Kingdom (UK).

Participants: 56,150 participants from the Trøndelag Health Study (HUNT) in Norway and 366,385 participants from UK Biobank recruited by postal invitation.

Outcomes: All-cause mortality and cause-specific mortality (cardiovascular, cancer, non-cardiovascular non-cancer).

Results: A previously published non-linear Mendelian randomization analysis of these data using the residual stratification method suggested a J-shaped association between genetically-predicted BMI and mortality outcomes with the lowest mortality risk at a BMI of around 25 kg/m^2. However, the "constant genetic effect" assumption required by this method is violated. The re-analysis of these data using the more reliable doubly-ranked stratification method still indicated a potential J-shaped relationship, but with much less certainty as there was less precision in estimates at the lower end of the BMI distribution. Evidence for a harmful effect of reducing BMI at low BMI levels was only present in some analyses, and where present, only below 20 kg/m^2. A harmful effect of increasing BMI for all-cause mortality was evident above 25 kg/m^2, for cardiovascular mortality above 24 kg/m^2, for cancer mortality above 30 kg/m^2, and for non-cardiovascular non-cancer mortality above 26 kg/m^2. In UK Biobank, the association between genetically-predicted BMI and mortality at high BMI levels was stronger in women than in men.

Conclusion: This research challenges findings from previous conventional observational epidemiology and Mendelian randomization investigations that the lowest level of mortality risk is at a BMI level of around 25 kg/m^2. Our results provide evidence that reductions in BMI will only increase mortality risk for a small proportion of the population, and increases in BMI will increase mortality risk for those with BMI above 25 kg/m^2.

For peer review only - http://bmjopen.bmj.com/site/about/guidelines.xhtml

Strengths and limitations of the study

- Mendelian randomization design minimizes bias due to confounding and reverse causation
- Large sample sizes enable powerful analyses even in low body mass index (BMI) individuals
- Validity of the genetic variants as instrumental variables cannot be verified
- Bias due to selection could be non-negligible and could vary across strata
- All estimates are averaged across a stratum of the population; individual effects of raising or lowering BMI may vary between individuals

For peer review only

INTRODUCTION

Body mass index (BMI) is a convenient and accessible measure of obesity. The epidemiological relationship between BMI and mortality is complex, with many observational studies conducted in Western countries showing a J-shaped relationship between BMI and mortality risk in the general population, such that mortality risk is lowest for those in the upper normal weight (BMI 20.0-24.9 kg/m^2)(1-3) or even the overweight (BMI 25.0-29.9 kg/m^2)(4-6) category rather than the lower normal weight (BMI 18.5-19.9 kg/m^2) or underweight category (BMI <18.5 kg/m^2). However, such findings may not reflect the causal relationship between BMI and mortality, as observational associations are influenced by confounding and reverse causation.

Mendelian randomization is an epidemiological technique for the analysis of observational data designed to avoid bias due to confounding and reverse causation(7). Rather than assessing associations between BMI and mortality directly, we assess associations between genetic variants that predict BMI levels and mortality risk(8). This is analogous to the analysis of a randomized controlled trial for weight loss(9), which would assess associations between the randomized intervention and the outcome, rather than associations with measured BMI in trial participants. According to Mendel's laws of inheritance, genetic variants should be uncorrelated with traits that they do not affect, and hence should be independent of potential confounders. As genetic variants are determined at conception, genetic associations should be protected from reverse causation(10). These properties mean that genetic variants are plausible instrumental variables; an instrumental variable is a variable that behaves as if it has been randomized in the population(11). Hence, associations between genetic predictors of BMI and mortality provide insights into the causal effect of BMI on mortality.

Non-linear Mendelian randomization is an extension of standard Mendelian randomization to investigate the shape of the causal relationship between an exposure and an outcome(12). A typical randomized trial estimates an average causal effect, representing the average impact of a population-wide shift in the distribution of an exposure; similarly, a standard Mendelian randomization analysis estimates a population-averaged causal effect(13). A widely used method for non-linear Mendelian randomization stratifies the population based on levels of the exposure, and estimates 'localized average causal effects', representing the average impact of a population-wide shift in the distribution of the exposure for that stratum of the population(13).

However, stratifying the population such that the instrumental variable assumptions still hold in the strata of the population is tricky. Stratifying on the exposure directly would break randomization, as exposure levels are influenced by the instrumental variables. Hence an individual in the lowest stratum of the population defined by the exposure with a genetic predisposition to high values of the exposure would be more likely to have low values of the confounders. This is an example of collider bias(14): the exposure is a common effect of the instrument and exposure—outcome confounders, and so stratification on the exposure leads to a conditional association between the instrument and confounders. The initial proposal for non-linear Mendelian randomization was to stratify on the residual exposure, defined as the residual from regression of the exposure on the instrumental variables(13). The residual exposure is independent of the instrumental variables by construction, and the instrumental variables will be independent of confounders within strata of the residual exposure under a "constant genetic effect" assumption(15). However, if the effect of the genetic instrumental variables on the exposure varies between individuals, then this residual stratification method can lead to severe bias in stratum-specific estimates(16, 17).

For peer review only - http://bmjopen.bmj.com/site/about/guidelines.xhtml

An alternative method, known as the doubly-ranked method, allows the instrumental variable effects on the exposure to vary between individuals provided that a weaker “rank-preserving assumption” holds(18). The rank-preserving assumption states that the ranking of individuals according to their exposure levels would be the same at all levels of the instrumental variable. That is, if the genotype of all individuals were set to any fixed value, the order of individuals according to their exposure levels would be the same. The doubly-ranked method is implemented by first ranking individuals according to their level of the instrument to form pre-strata with similar values of the instrument, and then ranking individuals within each pre-stratum according to their level of the exposure, so that the lowest stratum contains the individual with the lowest exposure value from pre-stratum 1, the individual with the lowest exposure value from pre-stratum 2, the individual with the lowest exposure value from pre-stratum 3, and so on. This method has been shown to be more reliable than the residual method in simulation studies(17, 18).

In a previous paper, we presented evidence for the effect of BMI on mortality from non-linear Mendelian randomization using the residual stratification method in the Norwegian Trøndelag Health (HUNT) and United Kingdom (UK) Biobank studies. A J-shaped association between genetically-predicted BMI and mortality was observed in each study population overall, although a monotonic increasing association (that is, an always-increasing association) was seen in never-smokers(19). Here, we present a re-analysis of the same datasets using the doubly-ranked stratification method. We first investigate whether the constant genetic effect assumption holds for this example, and explore the validity of the instrumental variable assumptions in strata of the population. We then present non-linear Mendelian randomization findings from the doubly-ranked stratification method.

For peer review only - http://bmjopen.bmj.com/site/about/guidelines.xhtml

METHODS

We present abbreviated descriptions of the datasets included in the analysis; detailed descriptions are in the original paper(19).

The HUNT study

We used data from the second wave (1995-1997) of the HUNT study on 65,229 individuals living in the northern part of Trøndelag and over the age of 20 years(20). Participants were followed up until 15 June 2023 or their date of death. The original paper only considered mortality outcomes up to April 2015. By extending the follow-up period, we increase the number of mortality events considered in the analysis from 12,015 to 18,836. We excluded participants without data on BMI or genetic variants for BMI, leaving 56,150 individuals for analysis.

The UK Biobank study

The UK Biobank cohort comprises around 500,000 participants (94% of self-reported European ancestry) aged 40 to 69 years at baseline. They were recruited between 2006-2010 in 22 assessment centres throughout the UK, and followed up until 28 February 2021 or their date of death(21). Again, the original paper only considered mortality outcomes up to February 2016. By extending the follow-up period, we increase the number of mortality events considered in the analysis from 10,344 to 25,021. We performed detailed quality control procedures on UK Biobank participants and on genetic variants as described previously(22). In total, 366,385 unrelated European ancestry participants were included in analyses.

SNPs and genetic score used as instrumental variables

77 single nucleotide polymorphisms (SNPs) were selected as candidate instrumental variables for BMI based on European sex-combined analyses in a genome-wide association study of the Genetic Investigation of ANthropometric Traits (GIANT) consortium(23). Two of these variants (rs12016871 and rs2033732) were not available in the HUNT study, and a further two variants (rs13021737 and rs16951275) were excluded from the analyses due to association with smoking status in the HUNT study. An externally-weighted score was calculated for each individual by multiplying the number of BMI-increasing alleles the individual carries by the variant's association with BMI from the GIANT study (**Supplementary Table 1**), and summing across the remaining 73 variants. Overall, the genetic score explained 2.0% and 1.6% of the variance in BMI in the HUNT and UK Biobank studies respectively, corresponding to F-statistics of 1121 and 5964. As a supplementary analysis, we repeated analyses for all-cause mortality using an extended genetic score comprising 633 variants taken from a more recent genome-wide association study of the GIANT consortium, which included UK Biobank (24). The variant list was taken from Verkouter *et al (25)*, who pruned the initial set of reported variants to exclude correlated variants.

We assessed the instrumental variable assumptions within strata by estimating associations between the genetic score and various traits that are competing risk factors: smoking status (ever versus never), alcohol status (current versus other), education level (post-secondary or higher versus other), occupation (currently employed versus other), age at recruitment, and sex.

Study design

We performed non-linear Mendelian randomization analyses to estimate the shape of the association between genetically-predicted BMI and the outcome. Our primary analysis considered all-cause mortality as the outcome. We also conducted subgroup analyses considering men and

For peer review only - http://bmjopen.bmj.com/site/about/guidelines.xhtml

women separately. In addition, we studied associations with cause-specific mortality events (cardiovascular, cancer, and non-cardiovascular non-cancer) in UK Biobank. These were defined based on International Classification of Diseases, Tenth Revision (ICD-10) codes (see **Supplementary Table 2** for details). We did not perform analyses stratified by smoking status (as presented in the original paper), as smoking status is influenced by BMI(26), and hence this stratification may introduce collider bias.

Statistical analyses

To estimate the non-linear relationship between genetically-predicted BMI and mortality risk, a fractional polynomial method was applied(12). In brief, first we divided the sample into 100 strata using the doubly-ranked method(18). For comparison, we also present results from stratification using the residual method for the primary outcome of all-cause mortality. We then calculated the linear Mendelian randomization estimate, referred to as a localized average causal effect (LACE), in each stratum of the population as a ratio of coefficients: the association of the genetic score with the outcome divided by the association of the genetic score with the exposure. Associations with the exposure (BMI) were obtained from linear regression; associations with the outcome (mortality) were obtained from Cox proportional hazards regression, using age as the timescale. While Cox proportional hazards regression is a semi-parametric method, the estimated non-parametric baseline hazard function is a function of the timescale, not the exposure value. All associations were adjusted for age, age-squared, sex, and centre (for UK Biobank).

We performed meta-regression of the LACE estimates against the mean of the exposure in each stratum in a flexible semiparametric framework using the derivative of fractional polynomial models of degrees 1 and 2. Under the Mendelian randomization assumptions, the slope of the curve represents the average causal effect of the exposure on the outcome for the stratum with that exposure level. A positive causal effect is evident at a particular exposure level when the lower and upper confidence limits for the curve both have a positive slope; and similarly an inverse causal effect is evident if both confidence limits have a negative slope. The reference point in analyses is set to 25 kg/m^2; however, this reference point is arbitrarily chosen, and the key indicator of an increasing or decreasing effect is not whether confidence intervals include or exclude the reference point, but rather whether the slope of the curve is positive or negative at a given exposure value.

We report two tests for non-linearity: a linearity test, which assesses whether a non-linear model fits the LACE estimates better than a linear model, and a trend test, which tests for a linear trend amongst the LACE estimates. Rejection of the linearity test indicates that a linear model is a suboptimal fit to the data; failure to reject means any improvement in fit for the best fitting non-linear model over the linear model is no more than would be expected due to chance alone.

As LACE estimates from the doubly-ranked method can be sensitive to specification of the analytic sample, we repeated analyses 100 times for each dataset omitting a small number of individuals in each iteration (12 individuals were removed at random in each iteration), and then combined estimates across iterations using Rubin's rules.

All statistical analyses were performed in R (version 4.3.1), and non-linear Mendelian randomization analyses were performed using the SUMnlmr package(27).

Patient and public involvement

No patients were involved in setting the research question or the outcome measures, nor were they involved in the design or implementation of the study. No patients were asked to advise on

For peer review only - http://bmjopen.bmj.com/site/about/guidelines.xhtml

interpretation or writing up of results. There are no specific plans to disseminate the results of the research to study participants or the relevant patient community.

For peer review only

For peer review only - http://bmjopen.bmj.com/site/about/guidelines.xhtml

RESULTS

Study populations

Baseline characteristics of the participants are provided in **Table 1**. Genetic associations with BMI in strata of the population defined by the doubly-ranked method are displayed in **Supplementary Figure 1**, and the BMI distribution in strata of the population is given in **Supplementary Table 3**. Per 1 standard deviation increase in the genetic score, BMI was 0.358 kg/m^2 greater in the lowest percentile of participants in the HUNT study, and 1.126 kg/m^2 greater in the highest percentile. Equivalent values for UK Biobank were 0.232 kg/m^2 in the lowest percentile and 1.645 kg/m^2 in the highest percentile. It is clear that the genetic associations with BMI differ strongly at different levels of BMI, and so the assumption required for the residual stratification method is violated. We note that similar estimates of the genetic associations with the exposure from the residual stratification method are unreliable, as these estimates from the residual method are similar even if the genetic effect on the exposure varies in the population(18).

Assessment of instrument validity

Associations with traits that are competing risk factors in strata of UK Biobank are displayed in **Figure 1**. As previously observed in the literature for other exposures(28), there are clear patterns in genetic associations with age and sex across strata. While there is no overall association of the genetic score with age or sex, there are associations in several of the strata.

As it is logically impossible that autosomal genetic variants can affect either age or sex, and as age and sex are typically measured without error, these associations must represent the effect of selection into the UK Biobank sample. While concerning, as we adjust for age and sex in our Mendelian randomization analyses, these associations will not lead to strong bias in our causal estimates (see **Supplementary Material** for a simulation study investigation into bias mitigation by adjustment for confounders that are predictors of selection). For other traits, both the magnitude of associations and patterns in associations are less strong, particularly for education level and occupation, which are important measures of social class. For alcohol status and smoking status, it is impossible to distinguish between genetic associations that reflect pleiotropic or selection effects and those that reflect a causal effect of the exposure (sometimes called "vertical pleiotropy"); the latter do not violate the instrumental variable assumptions. Hence both the associations and any pattern of associations may reflect violations of the instrumental variable assumptions or selection bias, but they could also reflect downstream effects of BMI which vary across strata.

Corresponding associations in the HUNT study are shown in **Supplementary Figure 2**. While there are some patterns in the stratum associations with age and sex, these are less pronounced than for UK Biobank, potentially due to differential selection being less strong in the HUNT study.

Comparison of results from residual and doubly-ranked methods

The shape of the association between genetically-predicted BMI and all-cause mortality is displayed in **Figure 2**, and stratum-specific LACE estimates are provided in **Supplementary Figure 3**. We note that **Figure 2** and **Supplementary Figure 3** differ in their presentation of these data; **Figure 2** plots the estimated association between genetically-predicted BMI values and mortality risk with respect to the reference exposure value, whereas **Supplementary Figure 3** plots the estimated effect of the exposure on mortality risk in each stratum. A smoothed version of the log transformed estimates in **Supplementary Figure 3** would represent the derivative of the curve shown in **Figure 2**.

For peer review only - http://bmjopen.bmj.com/site/about/guidelines.xhtml

When stratifying the population using the residual method, there is a distinct J-shaped relationship between BMI and mortality, particularly in the UK Biobank study, with minimum risk at a BMI level of around 25 kg/m^2. However, as discussed above, results from the residual stratification method are unreliable here. In contrast, when stratifying using the doubly-ranked method, the relationship between BMI and mortality is far more uncertain at the lower end of the BMI distribution. In the HUNT study, the association is mildly J-shaped, with a null slope up to a BMI of around 28 kg/m^2, and a positive slope above this level. For UK Biobank, there is some indication of an upturn in mortality risk at very low levels of BMI (the slope of the curve is negative below 20 kg/m^2), but otherwise the relationship is flat (and compatible with a null effect) up to a BMI of around 25 kg/m^2, and has an increasing positive slope above this level. The linearity and trend tests for the residual method indicate evidence supporting non-linearity for UK Biobank ($p_{linearity} = 5\times10^{-4}$, $p_{trend} = 2\times10^{-5}$). In contrast, these tests for the more reliable doubly-ranked method do not provide compelling evidence supporting non-linearity (**Figure 2**).

Similar results were observed when inverse-normal rank transforming the exposure (**Supplementary Figure 4**). When dividing the population into 50 strata, results were similar in both studies. When dividing into 200 strata, results were similar for UK Biobank, but differed for the HUNT study, as the best-fitting curve was monotone with no J-shaped upturn at the lower end of the BMI distribution (**Supplementary Figure 5**). When using the extended genetic score (633 variants) in UK Biobank, a pattern of results similar to the main analysis was observed, but with narrower confidence intervals as the extended score explains more variance in BMI (**Supplementary Figure 6**). There was still some indication of a J-shaped curve, although minimum mortality risk was at a BMI level of 20-21 kg/m^2, and evidence for an upturn in risk at low BMI levels was weaker.

Sex-stratified analyses

Sex-stratified analyses from the doubly-ranked method are presented in **Figure 3**, and stratum-specific LACE estimates are provided in **Supplementary Figure 7**. In each case, there is some indication of a J-shaped relationship, but an upturn in mortality risk is only evident below 20 kg/m^2 (below 22 kg/m^2 for men in the HUNT study). Otherwise, estimates are generally compatible with the null up to a BMI of 25 kg/m^2, and positive above this level. Evidence for a harmful effect of increases in BMI for both men and women is weaker in the HUNT study, although a harmful effect above 30 kg/m^2 is evident for men. The slope above 25 kg/m^2 is steeper for women than men in UK Biobank. The linearity and trend tests do not provide compelling evidence supporting non-linearity in any analysis (**Figure 3**).

Cause-specific mortality

Analyses investigating cause-specific mortality from the doubly-ranked method in UK Biobank are presented in **Figure 4**, and stratum-specific LACE estimates are provided in **Supplementary Figure 8**. Again, there is some indication of a J-shaped relationship for each outcome. This is most pronounced for cardiovascular mortality, and less evident for cancer and non-cardiovascular non-cancer mortality. An upturn in risk at low BMI levels was only evident for cardiovascular mortality, and only below 20 kg/m^2. Otherwise, the relationship was compatible with the null up to around 24 kg/m^2 for cardiovascular mortality, 26 kg/m^2 for non-cardiovascular non-cancer mortality, and 30 kg/m^2 for cancer mortality, and positive above this level. The slope above 25 kg/m^2 is steepest for cardiovascular mortality, then non-cardiovascular non-cancer, and finally for cancer mortality. The linearity and trend tests do not provide any evidence supporting non-linearity for any outcome, indicating that a non-linear model does not fit the data significantly better than a linear model for any outcome (**Figure 4**).

For peer review only - http://bmjopen.bmj.com/site/about/guidelines.xhtml

DISCUSSION

In this manuscript, we have investigated the shape of associations between genetically-predicted BMI and mortality outcomes to provide an indication of the causal effect of BMI on mortality risk at different levels of BMI using non-linear Mendelian randomization. While the curves generally display a J-shaped relationship, evidence for a harmful average effect of decreased BMI on mortality at low BMI levels was limited. Evidence supporting non-linearity from statistical hypothesis tests for the doubly-ranked method was unconvincing for all mortality outcomes. In UK Biobank, the slope representing the estimated effect of BMI on mortality risk above 25 kg/m^2 was sharper for women than for men, and sharpest for cardiovascular mortality compared with cancer mortality and non-cardiovascular non-cancer mortality. There was evidence for a harmful effect of high BMI on all mortality subtypes.

Our findings challenge "obesity paradox" results from observational epidemiology and previous Mendelian randomization analyses using the residual method, which suggest that increases in BMI might reduce risk of mortality for a substantial proportion of the general population. While the minimum of the estimated curve for all-cause mortality was at a BMI of around 23 kg/m^2 in UK Biobank, the curve was near flat from 22-24 kg/m^2, and was positive from 25 kg/m^2 upwards. Evidence for a harmful effect of decreased BMI at low levels of BMI (as indicated by negative slopes for both the upper and lower confidence limits of the BMI—mortality curve) was only present in some analyses, and only below 20 kg/m^2 when present. Amongst cause-specific mortality outcomes, this was only evident for cardiovascular mortality. In the HUNT study, there was no strong evidence for an effect of BMI on all-cause mortality at low BMI levels in overall analyses, and evidence for a harmful effect of increased BMI above around 28 kg/m^2. A potential reason for the discrepancy is that UK Biobank only contains participants from the age of 40 upwards, whereas the HUNT study includes younger individuals. Hence underweight strata in UK Biobank are more likely to comprise primarily of older, frailer individuals, whereas underweight strata in the HUNT study include more healthy, slim individuals.

Our investigation provides further empirical evidence that the assumptions required for the residual stratification method for non-linear Mendelian randomization are likely to be violated in practice, and that results from the residual method can be substantially different to those from the doubly-ranked method(17). The ratio between the genetic association with BMI in the highest versus lowest percentile group was 3.1 in the HUNT study and 7.1 in UK Biobank, whereas the assumptions required by the residual method imply that these associations should be constant across the distribution of the exposure. Particularly in UK Biobank, the residual method provided strong evidence for non-linearity in the effect of BMI on mortality risk, giving a J-shaped relationship with the minimum risk level at around 25 kg/m^2. This is similar to the confounded association observed in traditional observational studies(2, 3). In contrast, the doubly-ranked method provided little evidence for non-linearity; formal statistical tests for non-linearity did not reject the null hypothesis of linearity in any analysis. While there was some indication of a J-shaped curve in the main analyses, estimates at the lower end of the BMI distribution were imprecise. One reason for this is that genetic associations with BMI were weaker at low levels of BMI in the doubly-ranked method, and so stratum-specific Mendelian randomization estimates are less precise in these strata. In some supplementary analyses using different numbers of strata, evidence for a J-shaped relationship in the HUNT dataset was absent, and the J-shape was less pronounced when using an extended genetic score in UK Biobank.

Previous investigations into the non-linear shape of the causal relationship between BMI and mortality have used a variety of approaches. Wade *et al* used the residual stratification method for

For peer review only - http://bmjopen.bmj.com/site/about/guidelines.xhtml

non-linear Mendelian randomization in UK Biobank(29), which showed a J-shaped relationship similar to the one we observe here. Jenkins *et al* investigated associations between a genetic score for BMI and mortality in UK Biobank(5), considering analyses in the full dataset, and restricted to those with a pre-existing morbidity condition. While they did not observe evidence for non-linearity, the genetic score only explains a small proportion of variance in BMI, and so substantial non-linearity may not be expected on this scale. The difference between average BMI levels at the lowest versus highest decile of the genetic score is less than 2 kg/m^2. Sulc *et al* investigated the effect of BMI on life expectancy (estimated as the mean of parental lifespan) in a Mendelian randomization framework using a polynomial approach similar to the residual method(30), except that instead of stratifying on the residual exposure, this approach constructs a polynomial function of the residual exposure. They showed a linear effect of increased BMI on reduced life expectancy, with no indication of non-linearity. However, this approach also has been shown in simulations to be vulnerable to variation in genetic effects on the exposure(18). Carslake *et al* performed an instrumental variable analysis to investigate the impact of BMI on mortality in the HUNT study(31), but using offspring BMI as an instrument for the BMI of study participants. This analysis did not provide evidence for a harmful effect of having low BMI. However, offspring BMI is unlikely to satisfy the instrumental variable assumptions for the exposure of participant BMI. A similar analysis was performed in the 1958 British birth cohort, and a similar conclusion was reached(32). Blond *et al* also performed an instrumental variable analysis using offspring BMI as an instrument for the BMI of study participants in the Copenhagen School Health Records Register(33), showing evidence of positive effects of increases in BMI on mortality at both high and low BMI levels. Finally, Kjøllesdal et al performed an instrumental variable analysis using early adulthood BMI as an instrument for the midlife BMI of study participants in various Norwegian health surveys(34), showing mild J-shaped associations in the relationship between BMI and mortality, but far less pronounced than from observational analyses of the same dataset.

Overall, there is general consensus from different approaches that the adverse relationship of low BMI with mortality is exaggerated in observational analyses. However, variation between these results indicates the importance of modelling assumptions, both for the identification of causal effects, and for the modelling of non-linear relationships. Indeed, in our investigations, if we had used fractional polynomials of degree 1 rather than degree 2, then we would have observed monotonic increasing relationships of BMI with all outcomes. This is because the simpler degree 1 models are dominated by behaviour at the upper end of the BMI distribution, where instruments are strongest and most mortality outcomes occur. The simpler degree 1 polynomials smooth over imprecise negative estimates at the lower end of the BMI distribution, and so do not allow uncertainty in the shape of the distribution (particularly at its lower end) to be accurately reflected.

Our analysis has several strengths, but also limitations. The Mendelian randomization design minimizes bias from confounding and reverse causation. The large sample sizes of the HUNT and UK Biobank studies enable powerful analyses, even in strata of the population with low BMI levels. These sample sizes also enable a fine stratification of individuals, meaning that outcome associations can be assessed in strata only consisting of low BMI (<20 kg/m^2) individuals.

However, there are important limitations, which should lead to caution in the interpretation of our findings. As with all Mendelian randomization analyses, findings are dependent on the validity of the genetic variants as instrumental variables. While we were able to show null associations of the genetic score with some traits, it is never possible to prove the validity of the instrumental variable assumptions beyond all doubt. To reduce the scope for population stratification, our analyses were limited to European ancestry participants. Our findings may therefore not be applicable to other

For peer review only - http://bmjopen.bmj.com/site/about/guidelines.xhtml

populations. Further, recruitment into UK Biobank is dependent on age, sex, and other factors, which leads to bias in Mendelian randomization estimates. While the effect of moderate selection on Mendelian randomization estimates is often slight(35), the extent of selection bias may differ between strata, leading to differential bias across stratum-specific estimates(28). It is likely that age and sex are the strongest predictors of study participants, and differential genetic associations with these traits were observed in UK Biobank. For other traits, patterns in genetic associations across strata were less evident, and may reflect downstream effects of BMI rather than instrument invalidity due to pleiotropy or selection bias. As the HUNT study focused on a specific geographic area of Norway, it achieved much higher recruitment rates (around 70%, compared with 5% in UK Biobank), and so should be less affected by selection bias. This was corroborated by our analyses, as genetic associations in strata were weaker in the HUNT study. While genetic associations are protected from reverse causation, strata membership is determined by BMI levels, and hence could be subject to reverse causation. In particular, low BMI strata could contain a large proportion of individuals whose BMI levels are low because of comorbidity. However, genetic associations should still provide a reliable guide as to the extent and direction of any causal effect of BMI in these strata. Finally, all estimates represent averages across strata of the population; individual effects of raising or lowering BMI may vary between individuals. Differences in estimates for different strata may reflect the changing composition of the strata, as the characteristics of those with BMI less than 20 kg/m^2 are likely to be different to those with higher BMI. A non-linear curve may reflect a combination of different effects in different subgroups of the population, rather than that the relationship between BMI and mortality risk is non-linear for any individual in the population(36).

Conclusions

In conclusion, non-linear Mendelian randomization analyses using the doubly-ranked stratification method provide strong evidence for harmful effects of increased BMI on mortality above 25 kg/m^2. Evidence for a harmful effect of low BMI was only present in some analyses, and where present, only below 20 kg/m^2.

For peer review only - http://bmjopen.bmj.com/site/about/guidelines.xhtml

Table 1: Baseline characteristics of study participants in HUNT and UK Biobank studies

	HUNT	UK Biobank
Number of participants (n)	56,150	366,385
Men (%)	47.1	45.9
Age at baseline in years (SD)	49.6 (16.6)	56.7 (8.0)
Number of deaths (n)	18,836	25,021
- Cardiovascular mortality events (n)	-	5212
- Cancer mortality events (n)	-	12,880
- Other (non-cardiovascular non-cancer) events (n)	-	6692
BMI in kg/m^2 (SD)	26.3 (4.1)	27.4 (4.8)
Median follow-up years	26.5	12.0
Ever-smokers (%)	55.9	46.1

BMI: body mass index; SD: standard deviation; Data are given as number of subjects (n), percentage (%), median, or mean (standard deviation). For 237 deaths in UK Biobank, sufficient data were not available to classify the event as cardiovascular, cancer, or non-cardiovascular non-cancer.

For peer review only

For peer review only - http://bmjopen.bmj.com/site/about/guidelines.xhtml

FIGURE CAPTIONS

Figure 1: Genetic associations with potential competing risk factors in strata of the UK Biobank population

Figure 2: Non-linear Mendelian randomization – dose-response curve between body mass index and all-cause mortality. Using the doubly-ranked method: A) HUNT, B) UK Biobank; using the residual method (unreliable when the genetic effect on the exposure varies): C) HUNT, D) UK Biobank. Gradient at each point of the curve is the localized average causal effect. Grey lines represent 95% confidence intervals. The reference value for BMI was taken as 25 kg/m^2.

Figure 3: Non-linear Mendelian randomization – dose-response curve between body mass index and all-cause mortality in men and women respectively using doubly-ranked method. A) HUNT, men only; B) HUNT, women only; C) UK Biobank, men only; D) UK Biobank, women only. Gradient at each point of the curve is the localized average causal effect. Grey lines represent 95% confidence intervals. The reference value for BMI was taken as 25 kg/m^2.

Figure 4: Non-linear Mendelian randomization in UK Biobank – dose-response curve between body mass index and cause-specific mortality using doubly-ranked method. A) Cardiovascular disease mortality; B) Cancer mortality. C) Mortality due to other causes (non-cardiovascular, non-cancer). Gradient at each point of the curve is the localized average causal effect. Grey lines represent 95% confidence intervals. The reference value for BMI was taken as 25 kg/m^2.

For peer review only

For peer review only - http://bmjopen.bmj.com/site/about/guidelines.xhtml

Contributor statement:

SB, YQS, and XMM conceptualized the project. SB and YQS performed formal analysis. SB, YQS, AZ, CB, AMM, and XMM contributed to the methodology and interpretation of findings. YQS and AMM helped with data curation. SB and XMM supervised the project. SB wrote the initial manuscript draft. All authors (SB, YQS, AZ, CB, AMM, and XMM) contributed to the drafting and editing of the manuscript. All authors (SB, YQS, AZ, CB, AMM, and XMM) have approved of the final version of the manuscript.

Competing interest statement: None declared.

Funding statement: This work was supported by the Wellcome Trust (grant number 225790/Z/22/Z), the United Kingdom Research and Innovation Medical Research Council (grant number MC_UU_00002/7), the British Heart Foundation (RG/18/13/33946) and the National Institute for Health and Care Research Cambridge Biomedical Research Centre (BRC-1215-20014; NIHR203312). YQS was supported by a researcher grant from The Liaison Committee for Education, Research and Innovation in Central Norway (project ID 2018/42794). AMM is funded by the National Institute for Health and Care Research Blood and Transplant Research Unit in Donor Health and Behaviour (NIHR203337). The views expressed are those of the authors and not necessarily those of the NIHR, NHSBT or the Department of Health and Social Care.

Data sharing statement: Data from the HUNT and UK Biobank studies are available on application to any bona fide scientific researcher. Summary statistics for genetic associations with the exposure and outcome in strata for UK Biobank are available at http://doi.org/10.6084/m9.figshare.25041686.

Ethical approval

The HUNT study has ethical approval from The Regional Committee for Medical Research in Norway (REK 2015/78). Approval for individual projects is regulated in conjunction with The Norwegian Social Science Data Services (NSD). The UK Biobank study has ethical approval from the North West Multi-centre Research Ethics Committee (16/NW/0274). This research has been conducted using the UK Biobank Resource under Application Number 7439.

Acknowledgments

The Trøndelag Health Study (HUNT) is a collaboration between the HUNT Research Centre (Faculty of Medicine and Health Sciences, NTNU, Norwegian University of Science and Technology), Trøndelag County Council, Central Norway Regional Health Authority and the Norwegian Institute of Public Health.

For peer review only

REFERENCES

1. Aune D, Sen A, Prasad M, Norat T, Janszky I, Tonstad S, et al. BMI and all cause mortality: systematic review and non-linear dose-response meta-analysis of 230 cohort studies with 3.74 million deaths among 30.3 million participants. BMJ. 2016;353:i2156.
2. Bhaskaran K, dos-Santos-Silva I, Leon DA, Douglas IJ, Smeeth L. Association of BMI with overall and cause-specific mortality: a population-based cohort study of 3.6 million adults in the UK. The Lancet Diabetes & Endocrinology. 2018;6(12):944-53.
3. Global BMI Mortality Collaboration, Di Angelantonio E, Bhupathiraju Sh N, Wormser D, Gao P, Kaptoge S, et al. Body-mass index and all-cause mortality: individual-participant-data meta-analysis of 239 prospective studies in four continents. Lancet. 2016;388(10046):776-86.
4. Flegal KM, Kit BK, Orpana H, Graubard BI. Association of All-Cause Mortality With Overweight and Obesity Using Standard Body Mass Index Categories: A Systematic Review and Meta-analysis. JAMA. 2013;309(1):71-82.
5. Jenkins DA, Wade KH, Carslake D, Bowden J, Sattar N, Loos RJF, et al. Estimating the causal effect of BMI on mortality risk in people with heart disease, diabetes and cancer using Mendelian randomization. International journal of cardiology. 2021;330:214-20.
6. Visaria A, Setoguchi S. Body mass index and all-cause mortality in a 21st century U.S. population: A National Health Interview Survey analysis. PLOS ONE. 2023;18(7):e0287218.
7. Davey Smith G, Ebrahim S. 'Mendelian randomization': can genetic epidemiology contribute to understanding environmental determinants of disease? International Journal of Epidemiology. 2003;32(1):1-22.
8. Lawlor DA, Harbord RM, Sterne JA, Timpson N, Davey Smith G. Mendelian randomization: using genes as instruments for making causal inferences in epidemiology. Statistics in Medicine. 2008;27(8):1133-63.
9. Thanassoulis G, O'Donnell CJ. Mendelian randomization: nature's randomized trial in the post-genome era. JAMA. 2009;301(22):2386-8.
10. Burgess S, Swanson SA, Labrecque JA. Are Mendelian randomization investigations immune from bias due to reverse causation? European Journal of Epidemiology. 2021;36(3):253-7.
11. Martens EP, Pestman WR, de Boer A, Belitser SV, Klungel OH. Instrumental variables: application and limitations. Epidemiology. 2006:260-7.
12. Staley JR, Burgess S. Semiparametric methods for estimation of a nonlinear exposure-outcome relationship using instrumental variables with application to Mendelian randomization. Genet Epidemiol. 2017;41(4):341-52.
13. Burgess S, Davies NM, Thompson SG. Instrumental variable analysis with a nonlinear exposure-outcome relationship. Epidemiology. 2014;25(6):877-85.
14. Cole SR, Platt RW, Schisterman EF, Chu H, Westreich D, Richardson D, Poole C. Illustrating bias due to conditioning on a collider. International Journal of Epidemiology. 2010;39(2):417-20.
15. Small DS. Commentary: Interpretation and Sensitivity Analysis for the Localized Average Causal Effect Curve. Epidemiology. 2014;25(6).
16. Davey Smith G. Mendelian randomisation and vitamin D: the importance of model assumptions. The Lancet Diabetes & Endocrinology. 2023;11(1):14.
17. Burgess S. Violation of the constant genetic effect assumption can result in biased estimates for non-linear Mendelian randomization. Human Heredity. 2022(In Press).
18. Tian H, Mason AM, Liu C, Burgess S. Relaxing parametric assumptions for non-linear Mendelian randomization using a doubly-ranked stratification method. PLOS Genetics. 2023;19(6):e1010823.
19. Sun Y-Q, Burgess S, Staley JR, Wood AM, Bell S, Kaptoge SK, et al. Body mass index and all cause mortality in HUNT and UK Biobank studies: linear and non-linear mendelian randomisation analyses. British Medical Journal. 2019;364:l1042.
20. Krokstad S, Langhammer A, Hveem K, Holmen TL, Midthjell K, Stene TR, et al. Cohort Profile: The HUNT Study, Norway. International Journal of Epidemiology. 2013;42(4):968-77.

For peer review only - http://bmjopen.bmj.com/site/about/guidelines.xhtml

21. Sudlow C, Gallacher J, Allen N, Beral V, Burton P, Danesh J, et al. UK Biobank: an open access resource for identifying the causes of a wide range of complex diseases of middle and old age. PLOS Medicine. 2015;12(3):e1001779.
22. Astle WJ, Elding H, Jiang T, Allen D, Ruklisa D, Mann AL, et al. The allelic landscape of human blood cell trait variation and links to common complex disease. Cell. 2016;167(5):1415-29.
23. Locke AE, Kahali B, Berndt SI, Justice AE, Pers TH, Day FR, et al. Genetic studies of body mass index yield new insights for obesity biology. Nature. 2015;518(7538):197-206.
24. Yengo L, Sidorenko J, Kemper KE, Zheng Z, Wood AR, Weedon MN, et al. Meta-analysis of genome-wide association studies for height and body mass index in ~700000 individuals of European ancestry. Human Molecular Genetics. 2018;27(20):3641-9.
25. Verkouter I, de Mutsert R, Smit RAJ, Trompet S, Rosendaal FR, van Heemst D, et al. The contribution of tissue-grouped BMI-associated gene sets to cardiometabolic-disease risk: a Mendelian randomization study. International Journal of Epidemiology. 2020;49(4):1246-56.
26. Carreras-Torres R, Johansson M, Haycock PC, Relton CL, Davey Smith G, Brennan P, Martin RM. Role of obesity in smoking behaviour: Mendelian randomisation study in UK Biobank. BMJ. 2018;361:k1767.
27. Mason AM, Burgess S. Software Application Profile: SUMnlmr, an R package that facilitates flexible and reproducible non-linear Mendelian randomization analyses. International Journal of Epidemiology. 2022;51(6):2014-9.
28. Hamilton FW, Hughes DA, Spiller W, Tilling K, Davey Smith G. Non-linear mendelian randomization: evaluation of biases using negative controls with a focus on BMI and Vitamin D. medRxiv. 2023:2023.08.21.23293658.
29. Wade KH, Carslake D, Sattar N, Davey Smith G, Timpson NJ. BMI and Mortality in UK Biobank: Revised Estimates Using Mendelian Randomization. Obesity. 2018;26(11):1796-806.
30. Sulc J, Sjaarda J, Kutalik Z. Polynomial Mendelian randomization reveals non-linear causal effects for obesity-related traits. HGG Advances. 2022;3(3):100124.
31. Carslake D, Davey Smith G, Gunnell D, Davies N, Nilsen TIL, Romundstad P. Confounding by ill health in the observed association between BMI and mortality: evidence from the HUNT Study using offspring BMI as an instrument. International Journal of Epidemiology. 2018;47(3):760-70.
32. Hyppönen E, Carslake D, Berry DJ, Power C, Davey Smith G. Estimating the influence of body mass index (BMI) on mortality using offspring BMI as an instrumental variable. International Journal of Obesity. 2022;46(1):77-84.
33. Blond K, Carslake D, Gjærde LK, Vistisen D, Sørensen TIA, Smith GD, Baker JL. Instrumental variable analysis using offspring BMI in childhood as an indicator of parental BMI in relation to mortality. Scientific Reports. 2021;11(1):22408.
34. Kjøllesdal MKR, Davey Smith G, Ariansen I, Kinge JM, Degerud E, Næss Ø. The association between BMI and mortality using early adulthood BMI as an instrumental variable for midlife BMI. Scientific Reports. 2018;8(1):11499.
35. Gkatzionis A, Burgess S. Contextualizing selection bias in Mendelian randomization: how bad is it likely to be? International Journal of Epidemiology. 2018;48(3):691-701.
36. Small DS. Commentary: Interpretation and sensitivity analysis for the localized average causal effect curve. Epidemiology. 2014;25(6):886-8.

For peer review only - http://bmjopen.bmj.com/site/about/guidelines.xhtml

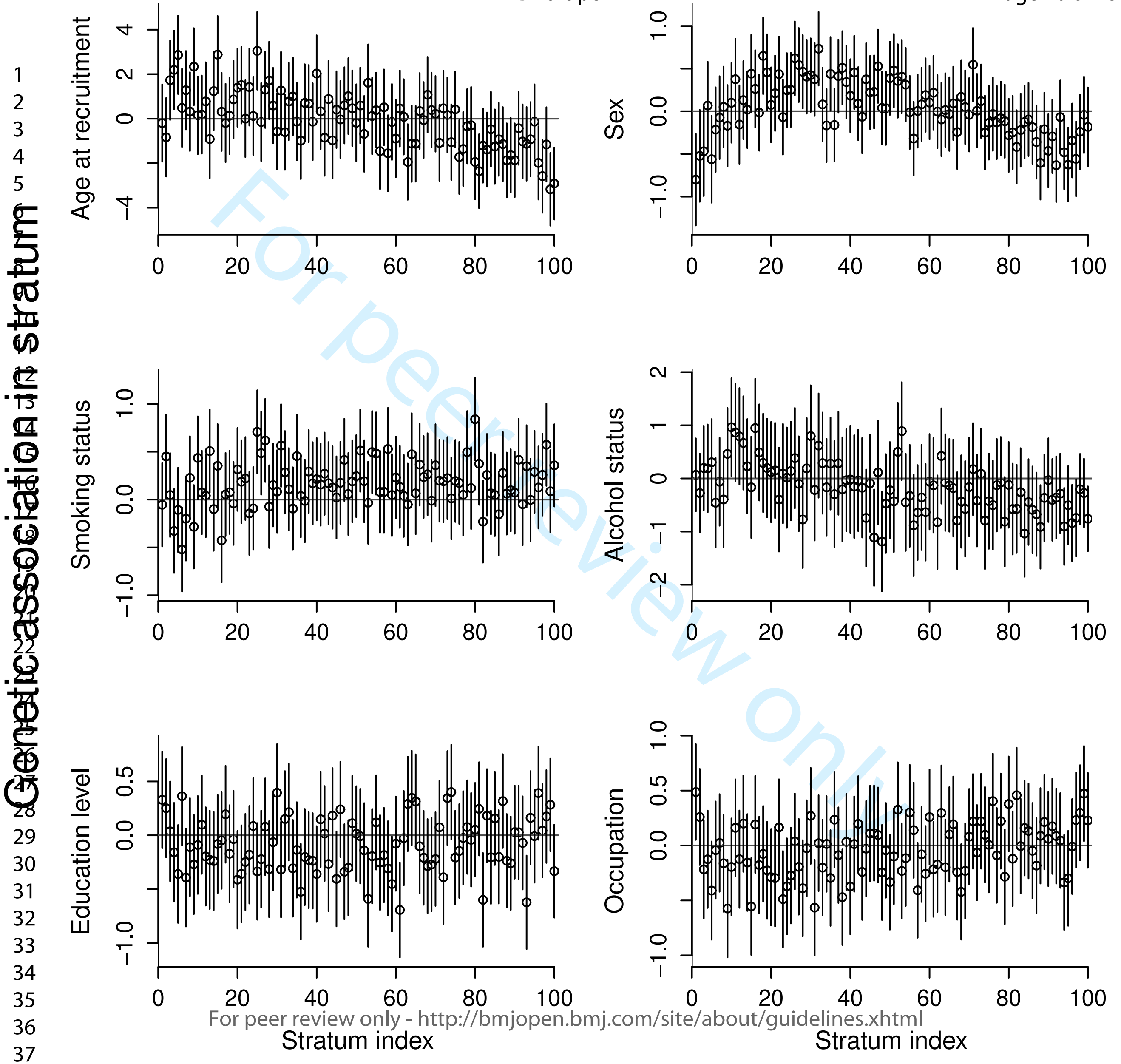

For peer review only

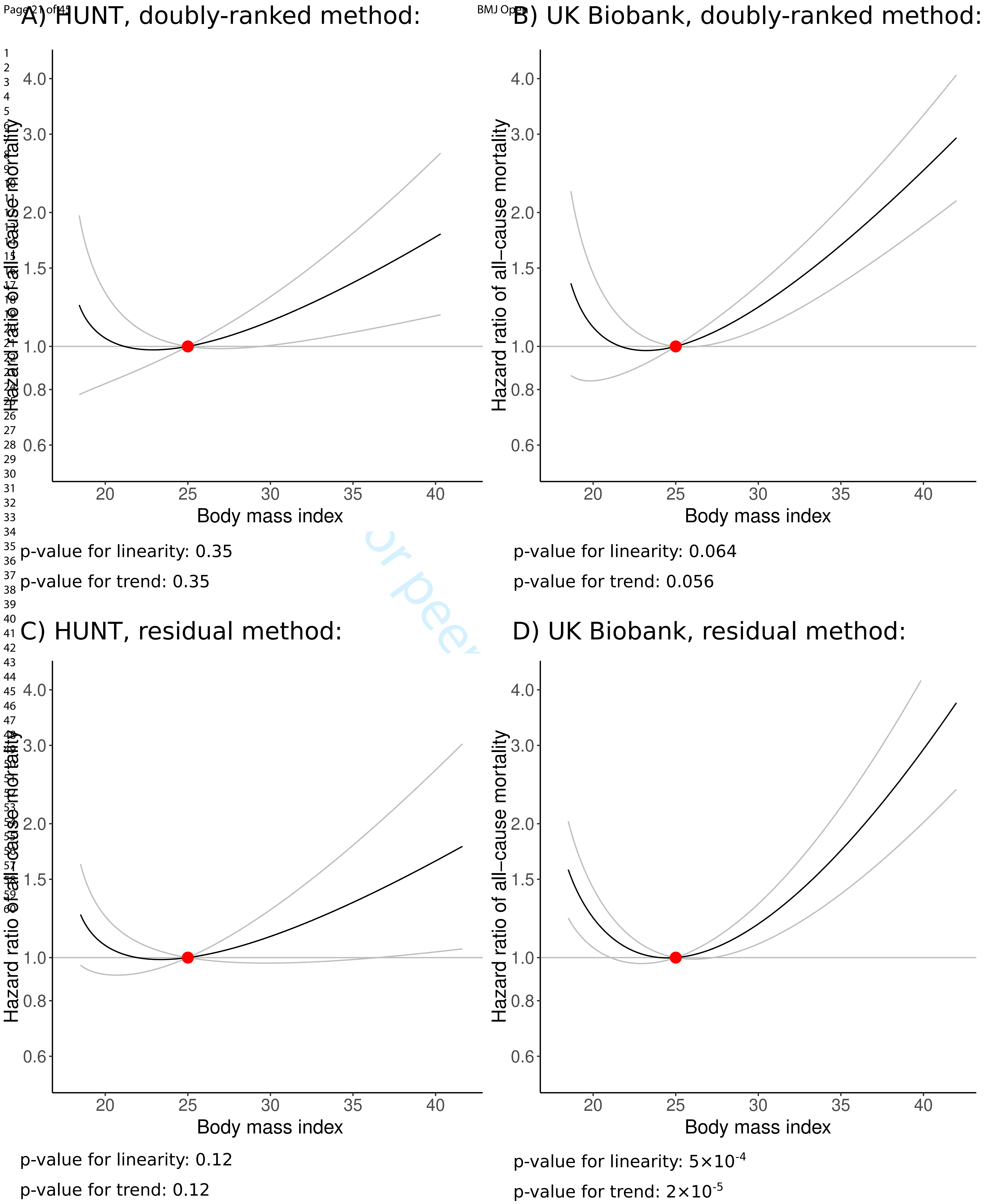
A) HUNT, doubly-ranked method:
B) UK Biobank, doubly-ranked method:
Hazard ratio of all−cause mortality
4.0
3.0
2.0
1.5
1.0
0.8
0.6
20
25
30
35
40
Body mass index
p-value for linearity: 0.35
p-value for trend: 0.35
p-value for linearity: 0.064
p-value for trend: 0.056
C) HUNT, residual method:
D) UK Biobank, residual method:
p-value for linearity: 0.12
p-value for trend: 0.12
p-value for linearity: 5×10-4
p-value for trend: 2×10-5

A) HUNT, men only:

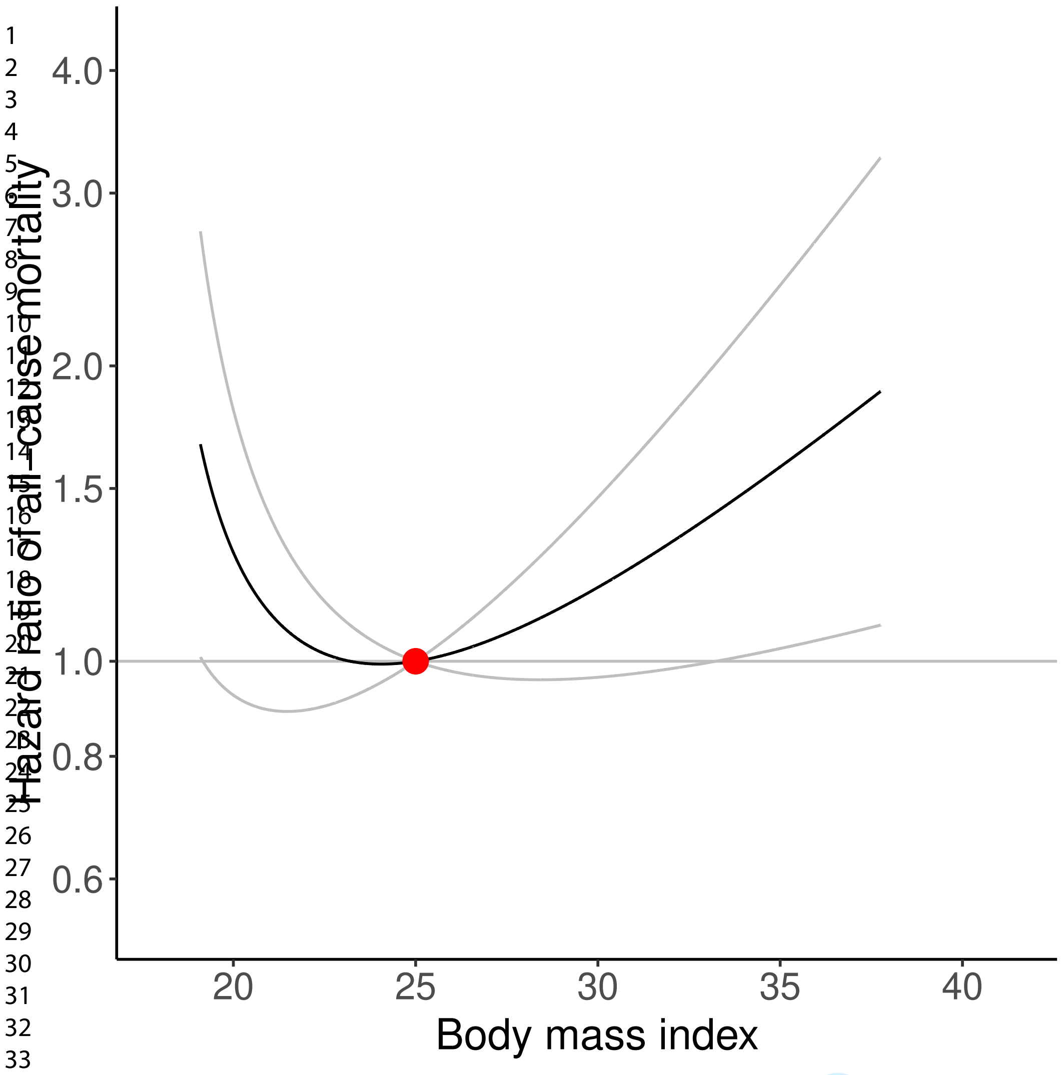

p-value for linearity: 0.050

p-value for trend: 0.22

B) HUNT, women only:

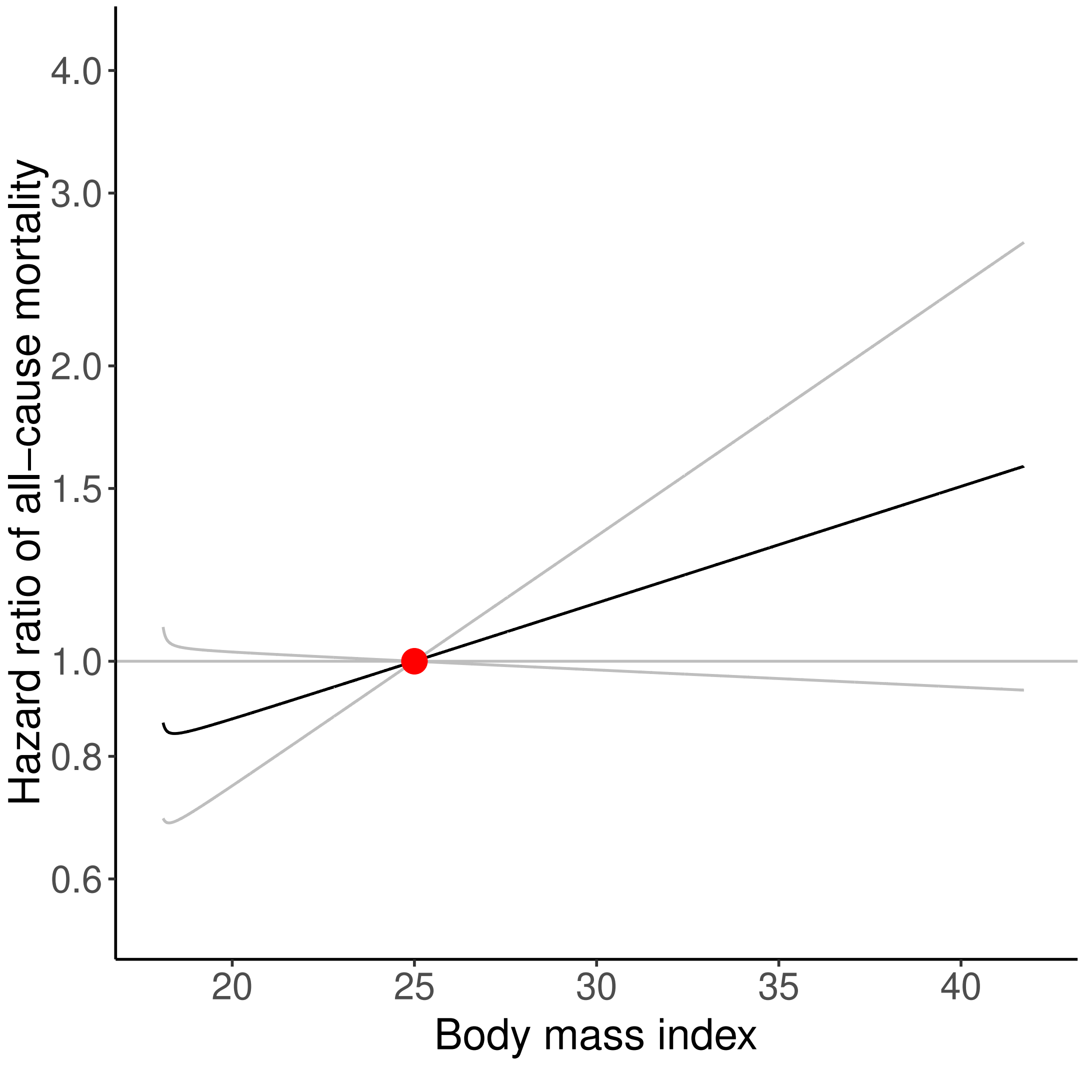

p-value for linearity: 0.99

p-value for trend: 0.79

C) UK Biobank, men only:

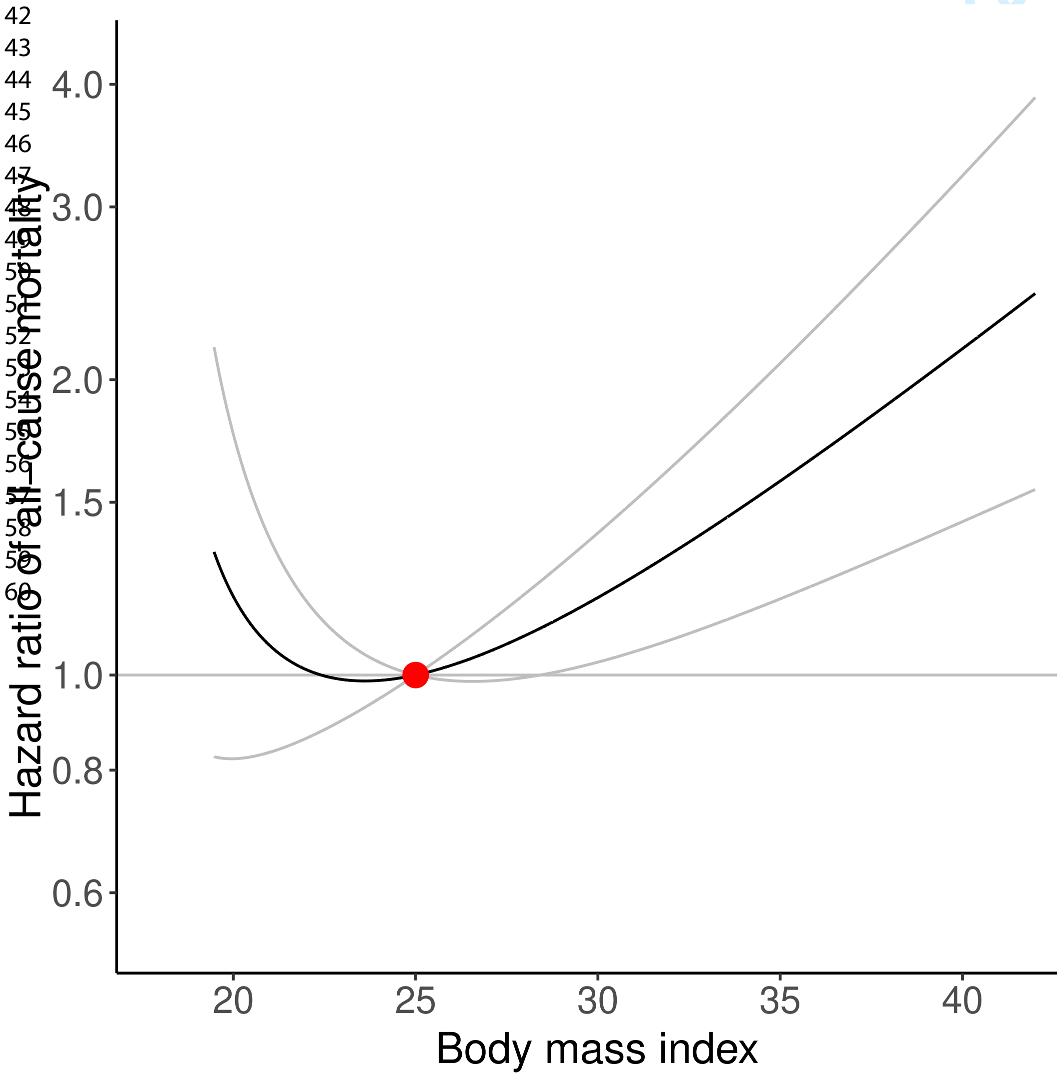

p-value for linearity: 0.18

p-value for trend: 0.18

D) UK Biobank, women only:

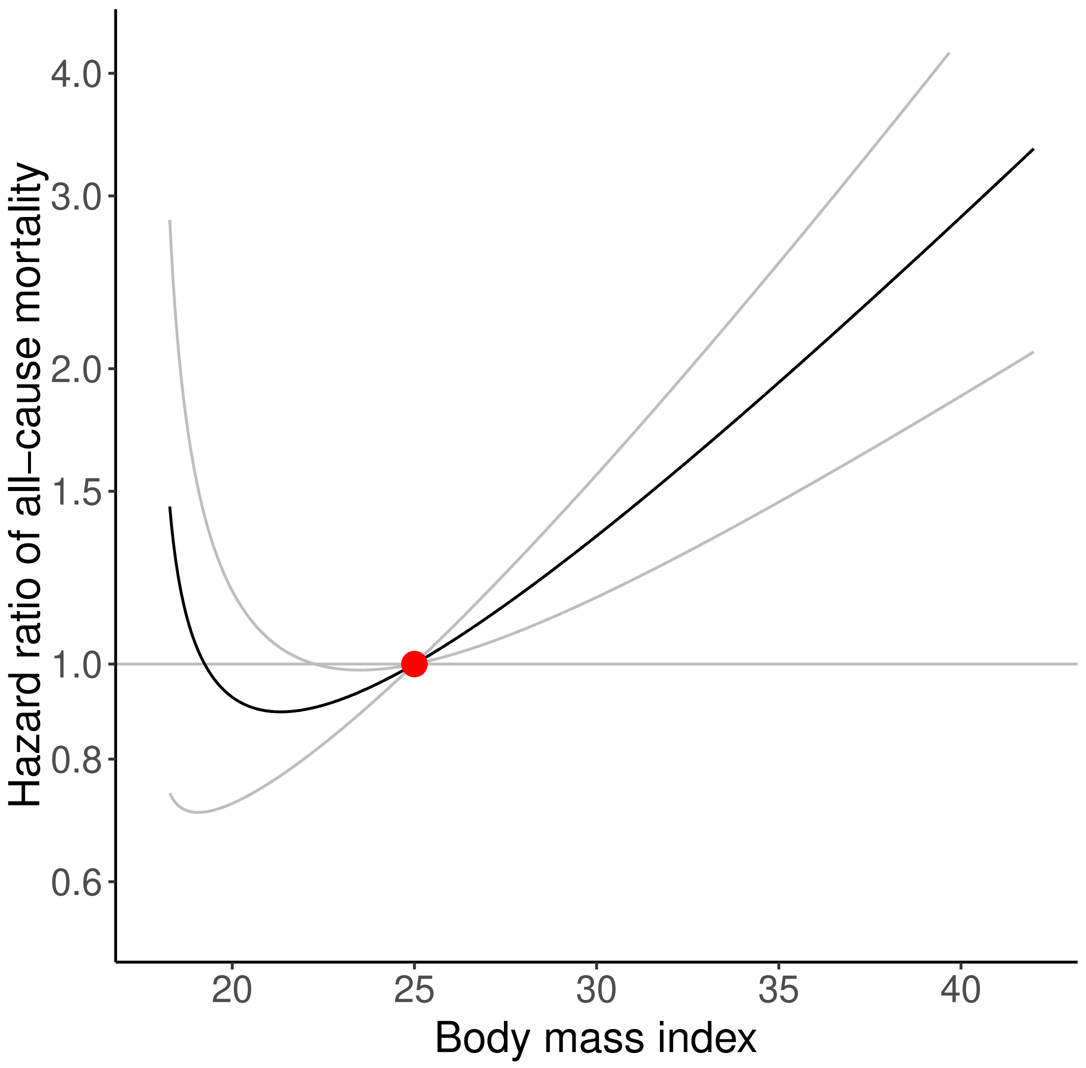

p-value for linearity: 0.21

p-value for trend: 0.18

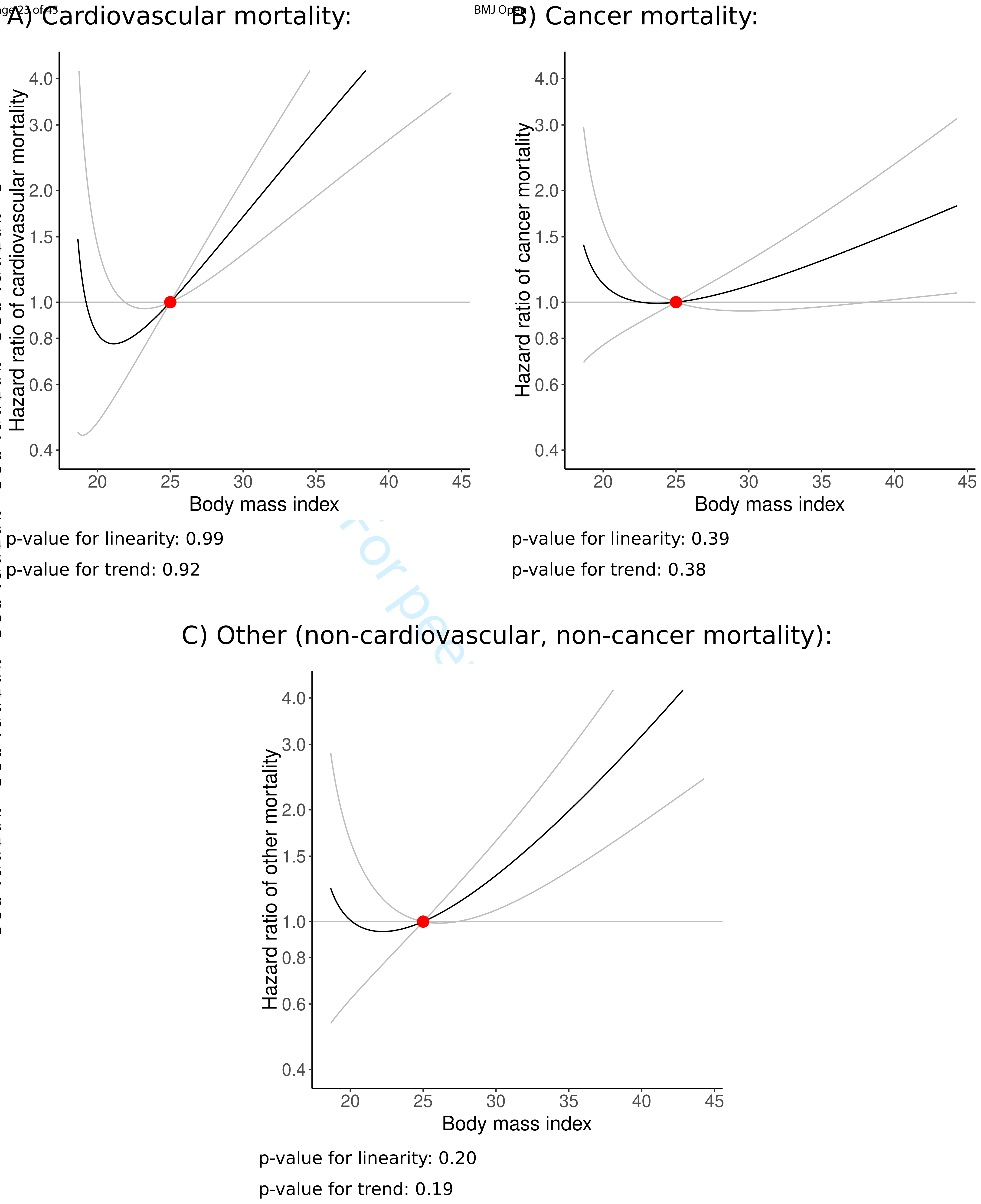
A) Cardiovascular mortality:
Hazard ratio of cardiovascular mortality
4.0
3.0
2.0
1.5
1.0
0.8
0.6
0.4
20
25
30
35
40
45
Body mass index
p-value for linearity: 0.99
p-value for trend: 0.92
B) Cancer mortality:
Hazard ratio of cancer mortality
4.0
3.0
2.0
1.5
1.0
0.8
0.6
0.4
20
25
30
35
40
45
Body mass index
p-value for linearity: 0.39
p-value for trend: 0.38
C) Other (non-cardiovascular, non-cancer mortality):
Hazard ratio of other mortality
4.0
3.0
2.0
1.5
1.0
0.8
0.6
0.4
20
25
30
35
40
45
Body mass index
p-value for linearity: 0.20
p-value for trend: 0.19

SUPPLEMENTARY MATERIAL

We here perform a simulation study investigating whether adjusting genetic associations for factors that influence selection reduces selection bias within strata in a non-linear Mendelian randomization framework. We generate simulated data for 1,000,000 individuals on a genetic variant *G*, an exposure *X*, an outcome *Y*, and competing risk factors *U* and *V*. For simplicity, all variables are modelled as continuous and normally distributed.

We consider two scenarios. In each scenario, the genetic variant is a valid instrumental variable in the population as a whole, and there is no true effect of the exposure on the outcome. This implies that the genetic variant should not be associated with the outcome. We estimate the association between the genetic variant and the outcome in a selected subset of the population.

In scenario 1, the data-generating model is:

$$G_i \sim Normal(0,1)$$
$$U_i \sim Normal(0,1)$$
$$V_i \sim Normal(0,1)$$
$$X_i \sim Normal(0.2\, G_i + 0.4\, U_i + 0.4V_i, 1)$$
$$Y_i \sim Normal(-0.5\, U_i + 0.2\, V_i, 1)$$

Participants (indexed by the *i* subscript) are selected into the sample with probability $expit(-2 + U_i + X_i)$, where $expit(x) = \frac{\exp(x)}{1+\exp(x)}$ is the inverse of the logistic (logit) function. Here *U* and *V* are both confounders of the relationship between the exposure and outcome, and selection depends on both *U* and *X*. We compare genetic associations with the outcome with and without adjustment for *U*.

In scenario 2, the data-generating model is:

$$G_i \sim Normal(0,1)$$
$$V_i \sim Normal(0,1)$$
$$X_i \sim Normal(0.2\, G_i + 0.8\, V_i, 1)$$
$$U_i \sim Normal(X_i, 1)$$
$$Y_i \sim Normal(0.5\, V_i, 1)$$

Participants are selected into the sample with probability $expit(-2 + U_i)$. Here *V* is a confounder (common cause) of the relationship between the exposure and outcome, and *U* is a downstream effect of the exposure *X*, and hence *U* is a collider (common effect) of *G* and *V*. Selection depends on *U* only. Again, we compare genetic associations with the outcome with and without adjustment for *U*.

In both cases, all normal distributions are independent. We simulate 1000 datasets for each scenario, and compare the mean estimate of the genetic association with the outcome and its empirical Type 1 error rate at a 5% significance level (that is, the proportion of 95% confidence intervals excluding the null). Around 15-20% of the 1,000,000 participants are selected in each simulated dataset.

In scenario 1, the mean association of the genetic variant with the outcome without adjustment for *U* was 0.014 (mean standard error 0.002), and the empirical Type 1 error rate was 100.0%. With adjustment for *U*, the mean association was -0.002 (mean standard error 0.002), and the empirical Type 1 error rate was 17.1%.

In scenario 2, the mean association of the genetic variant with the outcome without adjustment for *U* was -0.010 (mean standard error 0.002), and the empirical Type 1 error rate was 97.9%. With

For peer review only - http://bmjopen.bmj.com/site/about/guidelines.xhtml

adjustment for *U*, the mean association was -0.030 (mean standard error 0.002), and the empirical Type 1 error rate was 100.0%.

In scenario 1, bias reduced considerably on adjustment for the factor influencing selection. Mean bias did not reduce to zero, as selection also depends on the exposure. We note that if selection did not additionally depend on the exposure, there would not be selection bias, as *U* is a confounder not a collider. However, even in this case with imperfect adjustment, the reduction in bias was substantial. In scenario 2, there was bias without adjustment for the collider *U*, but mean bias sharpened (got worse) on adjustment for *U*. Scenario 1 is more likely to be representative of the situation we see in the paper, as sex and age cannot be consequences of the exposure, but are likely to be confounders.

This simulation study is deliberately designed to be relatively simple and limited in scope, as we do not believe that any simulation study would be able to prove conclusively that covariate adjustment eliminates selection bias in all cases where selection depends on a confounder. Indeed, we do not make that claim; rather we claim that adjustment for confounders that are key predictors of selection can ameliorate bias due to selection. There will always be intrinsic uncertainty in practice, as we do not know all the factors that influence selection, and we would be cautious to adjust for too many variables in a Mendelian randomization analysis, particularly for variables that could be mediators on the causal pathway from the exposure to the outcome, or could be colliders. As age and sex cannot logically be caused by the exposure, they can be safely adjusted for without risk of these variables being mediators or colliders [S1]. Hence, if age and sex are strong predictors of selection into the sample, then adjustment for age and sex should reduce selection bias in Mendelian randomization investigations.

We believe that any residual bias from selection is likely to be low, in line with previous simulation studies that have shown selection effects need to be substantial in magnitude to have non-negligible biasing effects on Mendelian randomization estimates [S2]. However, we acknowledge that this imposes an additional level of uncertainty on the results of a non-linear Mendelian randomization analysis, on top of the existing uncertainty in the Mendelian randomization paradigm due to the validity of the instrumental variable assumptions, and other factors. All methods for making causal inferences from observational data rely on untestable assumptions, and findings should be interpreted with appropriate caution.

SUPPLEMENTARY REFERENCES

S1. Burgess S, Davey Smith G, Davies NM, Dudbridge F, Gill D, Glymour MM, Hartwig FP, Kutalik Z, Holmes MV, Minelli C, Morrison JV, *et al*. Guidelines for performing Mendelian randomization investigations: update for summer 2023. Wellcome Open Research 2023; 4:186.

S2. Gkatzionis A, Burgess S. Contextualizing selection bias in Mendelian randomization: how bad is it likely to be? International Journal of Epidemiology. 2019; 48(3):691-701.

For peer review only - http://bmjopen.bmj.com/site/about/guidelines.xhtml

Supplementary Table 1: Genetic variants and associations with BMI based on European sex-combined analysis from GIANT [23]. Beta-coefficents are in standard deviation units.

SNP	Effect allele*	Other allele	Beta-coefficient	Standard error of beta	Number of subjects	*P*-value
rs1558902	A	T	0.082	0.003	320 073	7.51E-153
rs6567160	C	T	0.056	0.004	321 958	3.93E-53
rs10938397	G	A	0.040	0.003	320 955	3.21E-38
rs543874	G	A	0.048	0.004	322 008	2.62E-35
rs2207139	G	A	0.045	0.004	322 019	4.13E-29
rs11030104	A	G	0.041	0.004	322 103	5.56E-28
rs3101336	C	T	0.033	0.003	316 872	2.66E-26
rs7138803	A	G	0.032	0.003	322 092	8.15E-24
rs10182181	G	A	0.031	0.003	321 759	8.78E-24
rs3888190	A	C	0.031	0.003	321 930	3.14E-23
rs1516725	C	T	0.045	0.005	320 644	1.89E-22
rs12446632	G	A	0.040	0.005	316 758	1.48E-18
rs2287019	C	T	0.036	0.004	300 921	4.59E-18
rs3817334	T	C	0.026	0.003	321 959	5.15E-17
rs2112347	T	G	0.026	0.003	322 019	6.19E-17
rs12566985	G	A	0.024	0.003	319 282	3.28E-15
rs3810291	A	G	0.028	0.004	296 261	4.81E-15
rs7141420	T	C	0.024	0.003	321 970	1.23E-14
rs13078960	G	T	0.030	0.004	322 135	1.74E-14
rs10968576	G	A	0.025	0.003	322 061	6.61E-14
rs17024393	C	T	0.066	0.009	297 874	7.03E-14
rs657452	A	G	0.023	0.003	313 651	5.48E-13
rs12429545	A	G	0.033	0.005	312 934	1.09E-12
rs12286929	G	A	0.022	0.003	321 903	1.31E-12
rs13107325	T	C	0.048	0.007	321 461	1.83E-12
rs11165643	T	C	0.022	0.003	320 730	2.07E-12
rs7903146	C	T	0.023	0.003	322 130	1.11E-11
rs10132280	C	A	0.023	0.003	321 797	1.14E-11
rs17405819	T	C	0.022	0.003	322 085	2.07E-11
rs1016287	T	C	0.023	0.003	321 969	2.25E-11
rs4256980	G	C	0.021	0.003	320 028	2.90E-11
rs17094222	C	T	0.025	0.004	321 770	5.94E-11
rs12401738	A	G	0.021	0.003	322 070	1.15E-10
rs7599312	G	A	0.022	0.003	322 024	1.17E-10
rs2365389	C	T	0.020	0.003	316 768	1.63E-10
rs205262	G	A	0.022	0.004	315 542	1.75E-10
rs2820292	C	A	0.020	0.003	321 707	1.83E-10
rs12885454	C	A	0.021	0.003	320 823	1.94E-10
rs16851483	T	G	0.048	0.008	233 929	3.55E-10
rs1167827	G	A	0.020	0.003	306 238	6.33E-10
rs758747	T	C	0.023	0.004	308 688	7.47E-10
rs1928295	T	C	0.019	0.003	321 979	7.91E-10
rs9925964	A	G	0.019	0.003	318 385	8.11E-10

For peer review only

rs11126666	A	G	0.021	0.003	321 979	1.33E-09
rs2650492	A	G	0.021	0.004	319 464	1.92E-09
rs6804842	G	A	0.019	0.003	321 463	2.48E-09
rs12940622	G	A	0.018	0.003	322 032	2.49E-09
rs11847697	T	C	0.049	0.008	306 243	3.99E-09
rs4740619	T	C	0.018	0.003	321 887	4.56E-09
rs13191362	A	G	0.028	0.005	321 902	7.34E-09
rs3736485	A	G	0.018	0.003	321 398	7.41E-09
rs17001654	G	C	0.031	0.005	233 722	7.76E-09
rs11191560	C	T	0.031	0.005	321 893	8.45E-09
rs1528435	T	C	0.018	0.003	321 924	1.20E-08
rs2075650	A	G	0.026	0.005	308 408	1.25E-08
rs1000940	G	A	0.019	0.003	321 836	1.28E-08
rs2033529	G	A	0.019	0.003	321 917	1.39E-08
rs11583200	C	T	0.018	0.003	322 095	1.48E-08
rs9400239	C	T	0.019	0.003	321 988	1.61E-08
rs10733682	A	G	0.017	0.003	320 727	1.83E-08
rs11688816	G	A	0.017	0.003	322 051	1.89E-08
rs11057405	G	A	0.031	0.006	314 111	2.02E-08
rs2121279	T	C	0.025	0.004	322 065	2.31E-08
rs29941	G	A	0.018	0.003	321 970	2.41E-08
rs11727676	T	C	0.036	0.006	296 401	2.55E-08
rs3849570	A	C	0.019	0.003	284 339	2.60E-08
rs6477694	C	T	0.017	0.003	322 048	2.67E-08
rs7899106	G	A	0.040	0.007	321 770	2.96E-08
rs2176598	T	C	0.020	0.004	316 848	2.97E-08
rs2245368	C	T	0.032	0.006	205 675	3.19E-08
rs17724992	A	G	0.019	0.004	319 588	3.42E-08
rs7243357	T	G	0.022	0.004	322 107	3.86E-08
rs1808579	C	T	0.017	0.003	322 032	4.17E-08

*BMI-increasing allele

For peer review only

Supplementary Table 2: ICD-10 codes for cause-specific mortality outcomes

Cause-specific mortality	ICD-10 codes
Cardiovascular	G45, I01, I03-I82, I87, I95-I99, F01, Q20-Q28, R96
Cancer	C00-C97, D00-D48
Non-cardiovascular non-cancer	All others

For peer review only

Supplementary Table 3: Distribution of BMI (kg/m^2) in strata defined by the doubly-ranked stratification method

Stratum number	HUNT Mean	HUNT Lower decile	HUNT Upper decile	UK Biobank Mean	UK Biobank Lower decile	UK Biobank Upper decile
1	18.4	17.5	19.8	18.6	17.8	20.0
2	19.3	18.2	20.4	19.6	18.5	20.7
3	19.9	18.8	20.8	20.1	19.1	21.1
4	20.3	19.4	21.1	20.5	19.6	21.5
5	20.6	19.7	21.5	20.9	20.0	21.8
6	20.9	20.1	21.7	21.1	20.3	22.0
7	21.1	20.3	21.9	21.4	20.6	22.3
8	21.3	20.5	22.1	21.6	20.8	22.5
9	21.5	20.7	22.3	21.8	21.0	22.7
10	21.7	20.9	22.5	22.0	21.2	22.8
11	21.8	21.1	22.7	22.2	21.4	23.0
12	22.0	21.2	22.8	22.4	21.6	23.2
13	22.1	21.4	22.9	22.5	21.7	23.3
14	22.3	21.5	23.1	22.7	21.9	23.5
15	22.4	21.7	23.2	22.8	22.0	23.7
16	22.5	21.8	23.3	23.0	22.2	23.8
17	22.7	21.9	23.5	23.1	22.3	23.9
18	22.8	22.0	23.6	23.2	22.4	24.1
19	22.9	22.1	23.7	23.4	22.6	24.2
20	23.0	22.2	23.8	23.5	22.7	24.3
21	23.1	22.4	23.9	23.6	22.8	24.4
22	23.2	22.5	24.0	23.7	22.9	24.5
23	23.3	22.6	24.1	23.8	23.0	24.7
24	23.4	22.7	24.2	24.0	23.2	24.8
25	23.6	22.8	24.3	24.1	23.3	24.9
26	23.6	22.9	24.5	24.2	23.4	25.0
27	23.8	23.0	24.5	24.3	23.5	25.2
28	23.8	23.1	24.7	24.4	23.6	25.3
29	23.9	23.1	24.8	24.5	23.7	25.4
30	24.0	23.2	24.8	24.6	23.8	25.5
31	24.1	23.3	25.0	24.7	23.9	25.6
32	24.2	23.4	25.0	24.8	24.0	25.7
33	24.3	23.5	25.1	24.9	24.1	25.8
34	24.4	23.6	25.2	25.0	24.2	25.9
35	24.5	23.7	25.3	25.1	24.3	26.0
36	24.6	23.8	25.4	25.2	24.4	26.1
37	24.7	23.8	25.5	25.3	24.5	26.2
38	24.8	23.9	25.6	25.4	24.6	26.3
39	24.8	24.0	25.7	25.5	24.7	26.4
40	24.9	24.1	25.8	25.6	24.8	26.6
41	25.0	24.2	25.9	25.7	24.9	26.7
42	25.1	24.3	26.0	25.8	25.0	26.8
43	25.2	24.3	26.1	25.9	25.1	26.9
44	25.3	24.4	26.2	26.0	25.1	27.0
45	25.4	24.5	26.3	26.1	25.3	27.1

For peer review only

46	25.5	24.6	26.4	26.2	25.4	27.2
47	25.6	24.7	26.4	26.3	25.4	27.3
48	25.6	24.8	26.6	26.5	25.5	27.4
49	25.7	24.8	26.7	26.6	25.6	27.5
50	25.8	25.0	26.8	26.7	25.7	27.6
51	25.9	25.0	26.9	26.8	25.8	27.7
52	26.0	25.1	26.9	26.9	25.9	27.8
53	26.1	25.2	27.0	27.0	26.0	28.0
54	26.2	25.3	27.1	27.1	26.1	28.1
55	26.3	25.4	27.2	27.2	26.2	28.2
56	26.4	25.5	27.3	27.3	26.3	28.3
57	26.5	25.5	27.4	27.4	26.4	28.4
58	26.6	25.6	27.5	27.5	26.5	28.6
59	26.7	25.7	27.6	27.6	26.6	28.7
60	26.8	25.8	27.7	27.7	26.7	28.8
61	26.9	25.9	27.9	27.9	26.8	28.9
62	27.0	26.0	27.9	28.0	26.9	29.1
63	27.1	26.1	28.1	28.1	27.0	29.2
64	27.2	26.2	28.2	28.2	27.1	29.3
65	27.3	26.3	28.3	28.3	27.2	29.5
66	27.4	26.4	28.4	28.5	27.3	29.6
67	27.5	26.5	28.6	28.6	27.5	29.8
68	27.6	26.6	28.7	28.7	27.6	29.9
69	27.7	26.7	28.8	28.9	27.7	30.0
70	27.9	26.8	28.9	29.0	27.8	30.2
71	28.0	26.9	29.0	29.1	28.0	30.4
72	28.1	27.0	29.1	29.3	28.1	30.5
73	28.2	27.1	29.4	29.4	28.2	30.7
74	28.3	27.2	29.5	29.6	28.4	30.9
75	28.5	27.4	29.6	29.8	28.5	31.1
76	28.6	27.5	29.8	29.9	28.7	31.2
77	28.7	27.6	29.9	30.1	28.8	31.4
78	28.9	27.8	30.1	30.3	28.9	31.6
79	29.0	27.9	30.3	30.4	29.1	31.8
80	29.2	28.0	30.5	30.6	29.3	32.1
81	29.4	28.2	30.7	30.8	29.4	32.3
82	29.5	28.3	30.9	31.0	29.6	32.6
83	29.7	28.4	31.1	31.3	29.8	32.8
84	29.9	28.6	31.3	31.5	30.0	33.0
85	30.1	28.8	31.5	31.7	30.2	33.3
86	30.3	28.9	31.8	32.0	30.4	33.7
87	30.6	29.1	32.0	32.3	30.6	34.0
88	30.8	29.3	32.3	32.5	30.9	34.4
89	31.0	29.5	32.6	32.9	31.1	34.7
90	31.3	29.7	33.0	33.2	31.4	35.2
91	31.6	30.0	33.3	33.6	31.7	35.6
92	32.0	30.3	33.7	34.0	32.0	36.2
93	32.3	30.6	34.2	34.5	32.4	36.7
94	32.8	30.9	34.8	35.0	32.8	37.4
95	33.3	31.4	35.5	35.7	33.3	38.2
96	33.9	31.8	36.1	36.4	33.9	39.2

For peer review only

97	34.7	32.4	37.1	37.4	34.6	40.5
98	35.8	33.3	38.7	38.7	35.5	42.2
99	37.3	34.1	40.5	40.5	36.7	44.8
100	40.3	35.6	43.2	44.3	38.8	47.9

We report the mean, lower decile (10th percentile of the distribution), and upper decile (90th percentile of the distribution) of the BMI distribution for participants in each stratum created by the doubly-ranked method. In the first and 100th strata, we instead report the 20th and 80th percentiles of the distribution.

For peer review only

Supplementary Figure 1: Associations between the genetic score and BMI in strata defined by the doubly-ranked method plotted against mean BMI in each stratum for: A) HUNT, B) UK Biobank. Error bars represent 95% confidence intervals.

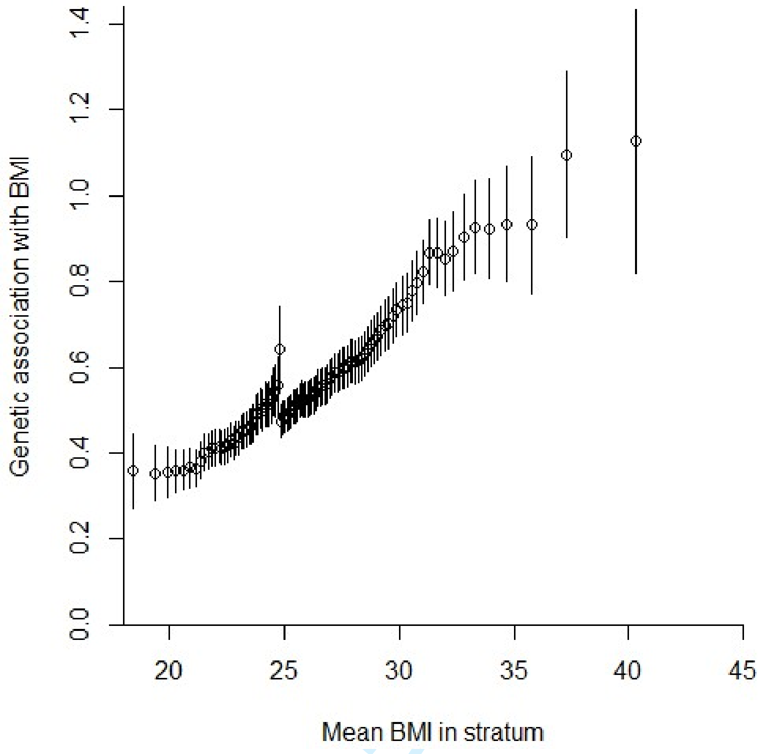

A.

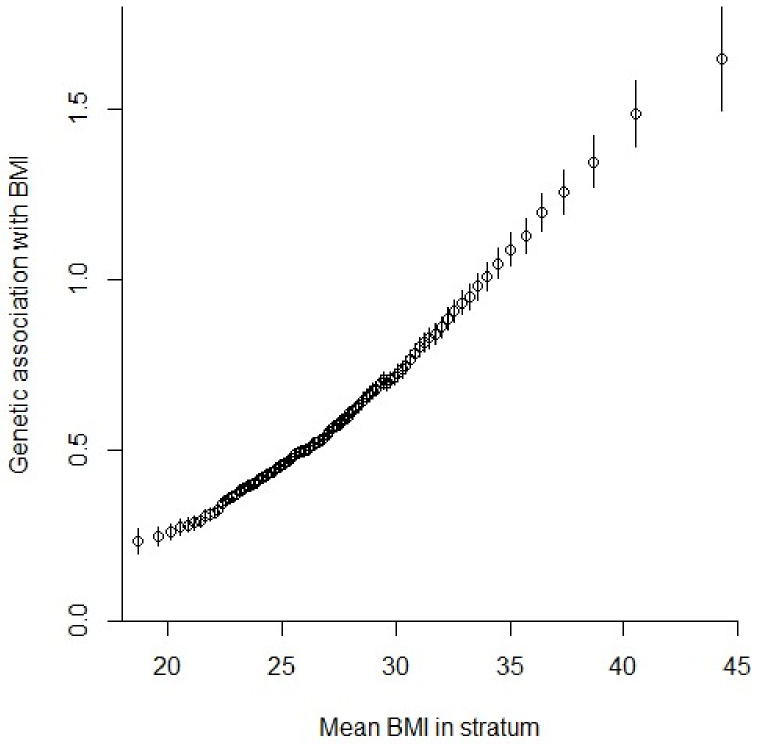

B.

For peer review only - http://bmjopen.bmj.com/site/about/guidelines.xhtml

Supplementary Figure 2: Genetic associations with potential competing risk factors in strata of the HUNT study population

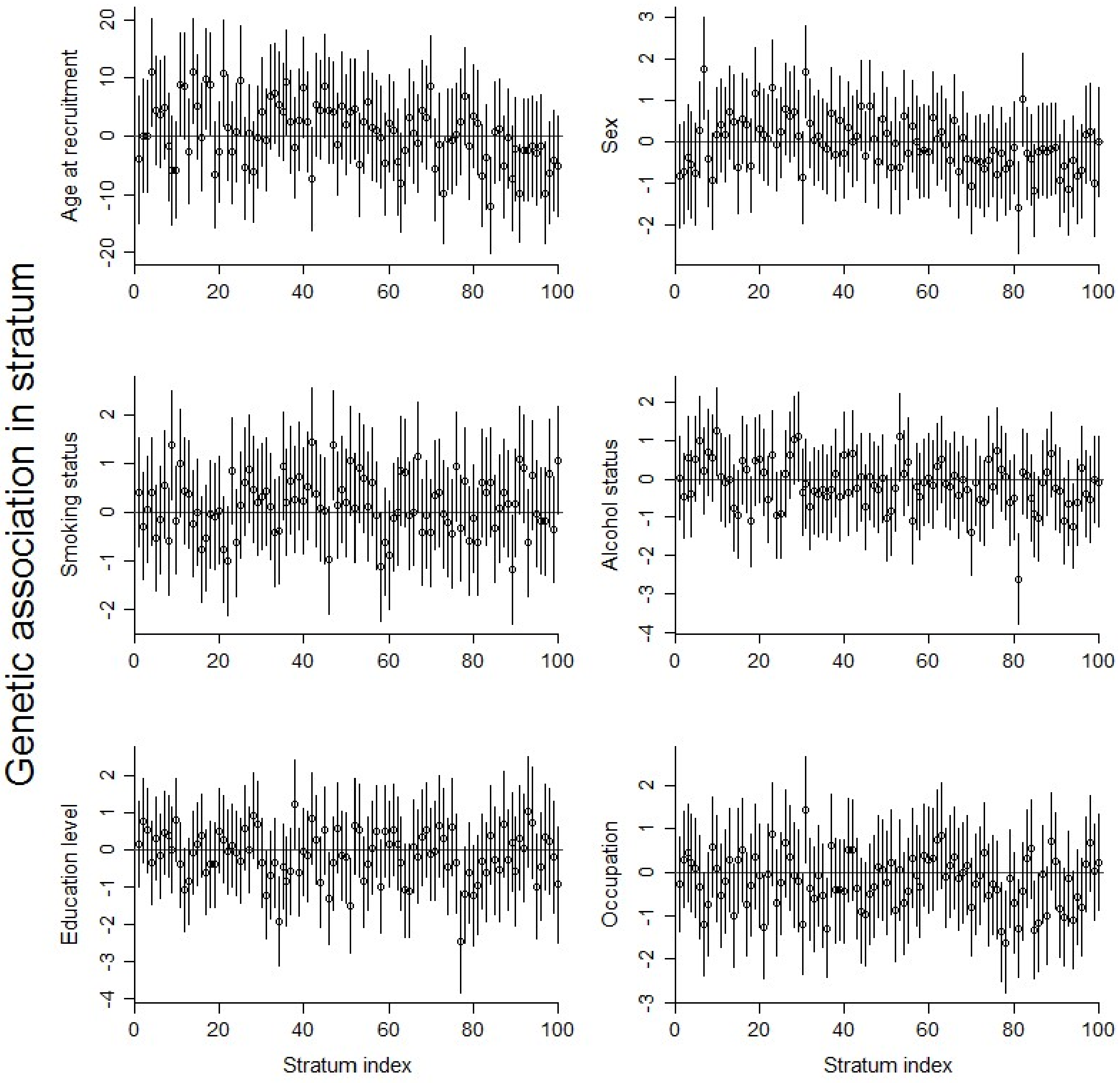

Points represent association estimates from linear (age at recruitment, years) or logistic regression (all other traits). Error bars represent 95% confidence intervals. Stratum 1 has lowest average BMI levels, stratum 100 has highest average BMI levels.

For peer review only - http://bmjopen.bmj.com/site/about/guidelines.xhtml

Supplementary Figure 3: Localized average causal effect estimates (stratum-specific estimates) for all-cause mortality plotted against mean BMI in each stratum. Estimates represent hazard ratios per 1 kg/m^2 increase in genetically-predicted BMI. Error bars represent 95% confidence intervals.

A) HUNT, doubly-ranked method:

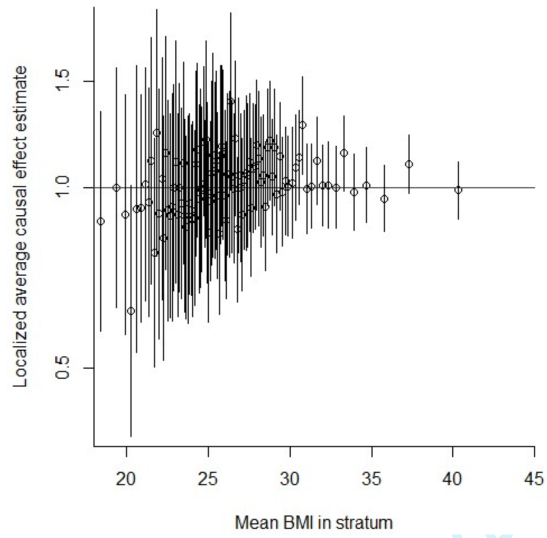

B) UK Biobank, doubly-ranked method:

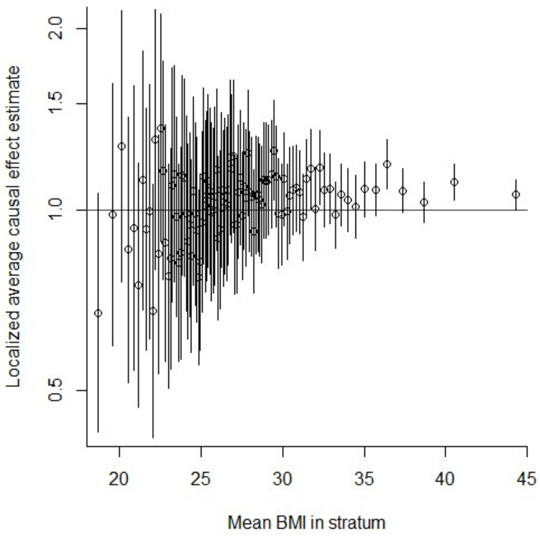

C) HUNT, residual method:

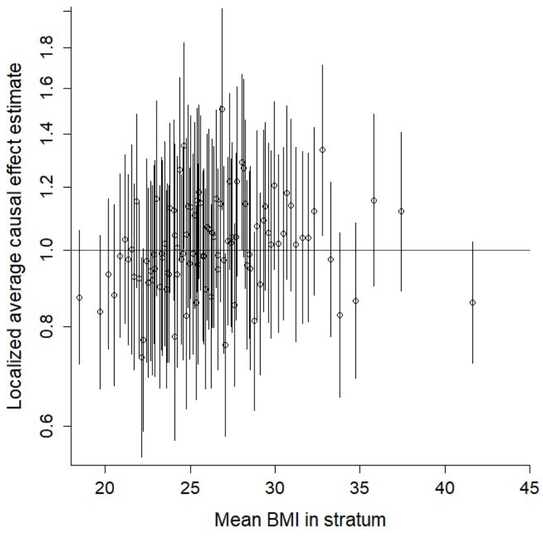

D) UK Biobank, residual method:

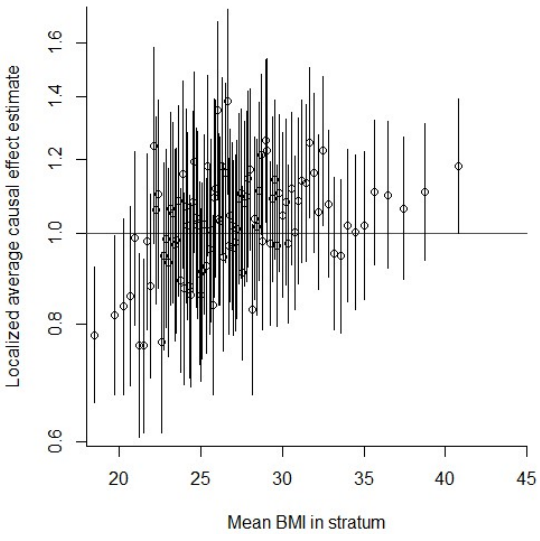

This presentation does not smooth over stratum-specific estimates, hence providing a representation of the causal effect of BMI which is not dependent on the choice of fractional polynomial smoothing. However, stratum-specific estimates are imprecise and variable, as they each only use 1% of the overall sample size. These plots differ from the presentation in the main text Figures: here, we plot the stratum-specific Mendelian randomization estimates, which (when log-transformed) reflect the slope of the curves in the main text figures.

For peer review only - http://bmjopen.bmj.com/site/about/guidelines.xhtml

Supplementary Figure 4: Non-linear Mendelian randomization – dose-response curve between body mass index and all-cause mortality using the doubly-ranked method with inverse-normal rank transformed BMI values in UK Biobank. Grey lines represent 95% confidence intervals. The reference value for BMI was taken as the median of the BMI distribution at 26.7 kg/m^2.

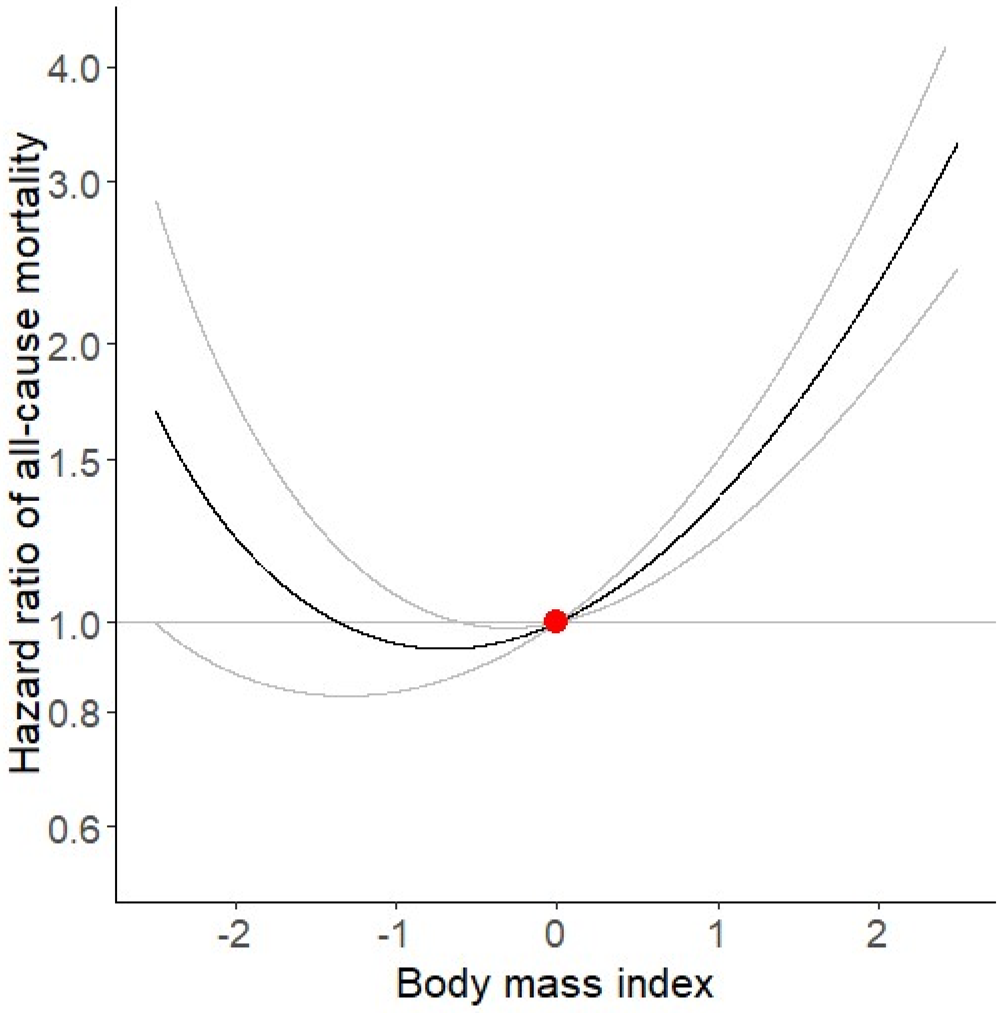

The labels on the horizontal axis represent the number of standard deviations below or above the median of the BMI distribution: for example, -1 represents one standard deviation below the median, and +2 represents two standard deviations above the median.

The reference values are:
-2 = 19.9 kg/m^2
-1 = 23.0 kg/m^2
0 = 26.7 kg/m^2
+1 = 31.6 kg/m^2
+2 = 39.2 kg/m^2

For peer review only - http://bmjopen.bmj.com/site/about/guidelines.xhtml

Supplementary Figure 5: Non-linear Mendelian randomization – dose-response curve between body mass index and all-cause mortality estimated using the doubly-ranked method: A) HUNT, B) UK Biobank dividing into 50 strata; C) HUNT, D) UK Biobank dividing into 200 strata. Gradient at each point of the curve is the localized average causal effect. Grey lines represent 95% confidence intervals. The reference value for BMI was taken as 25 kg/m^2.

A) HUNT, 50 strata:

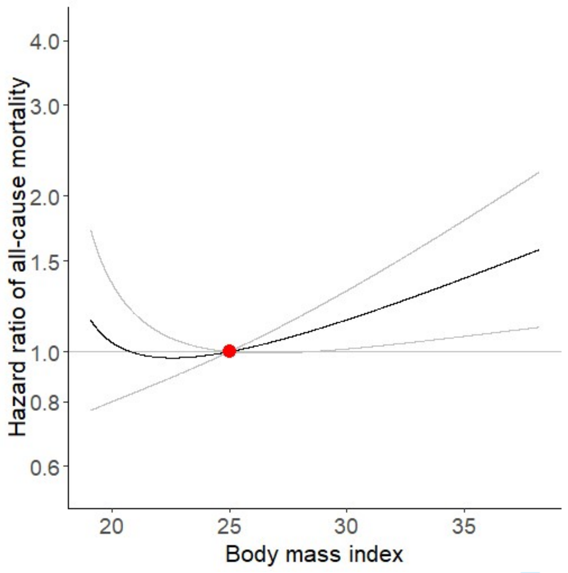

p-value for linearity: 0.50
p-value for trend: 0.49

B) UK Biobank, 50 strata:

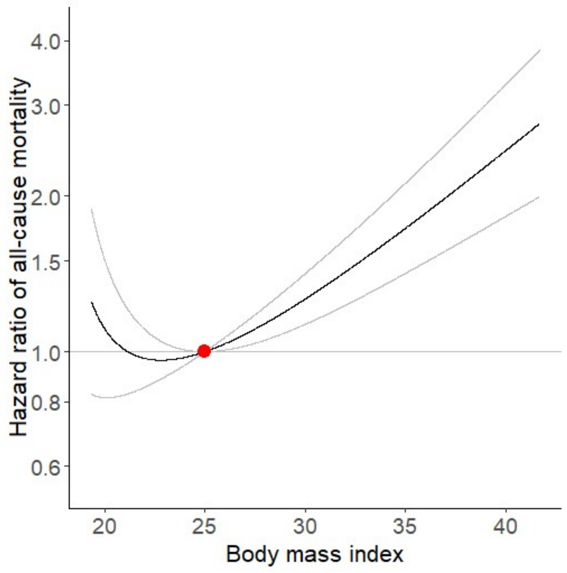

p-value for linearity: 0.10
p-value for trend: 0.09

C) HUNT, 200 strata:

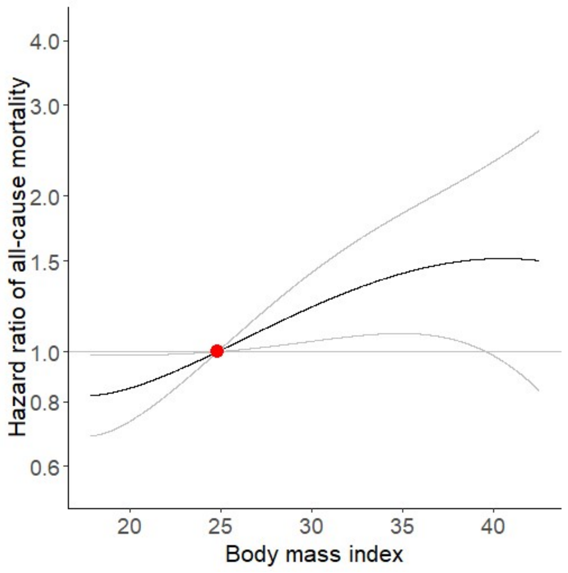

p-value for linearity: 0.99
p-value for trend: 0.88

D) UK Biobank, 200 strata:

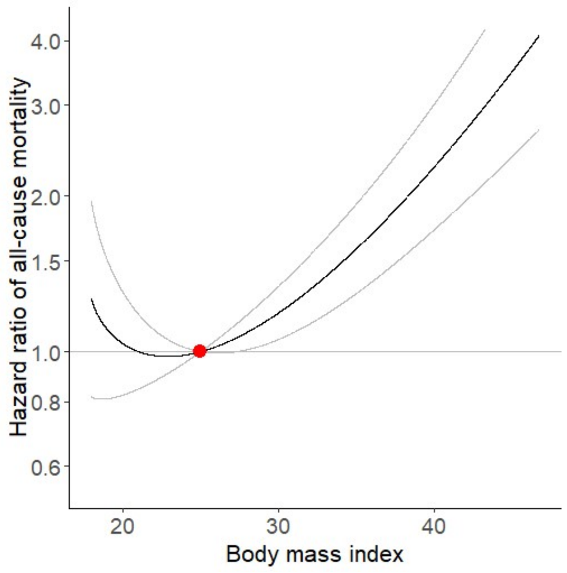

p-value for linearity: 0.28
p-value for trend: 0.16

For peer review only - http://bmjopen.bmj.com/site/about/guidelines.xhtml

Supplementary Figure 6: Non-linear Mendelian randomization – dose-response curve between body mass index and all-cause mortality estimated using the doubly-ranked method and the extended genetic score in UK Biobank. Gradient at each point of the curve is the localized average causal effect. Grey lines represent 95% confidence intervals. The reference value for BMI was taken as 25 kg/m^2.

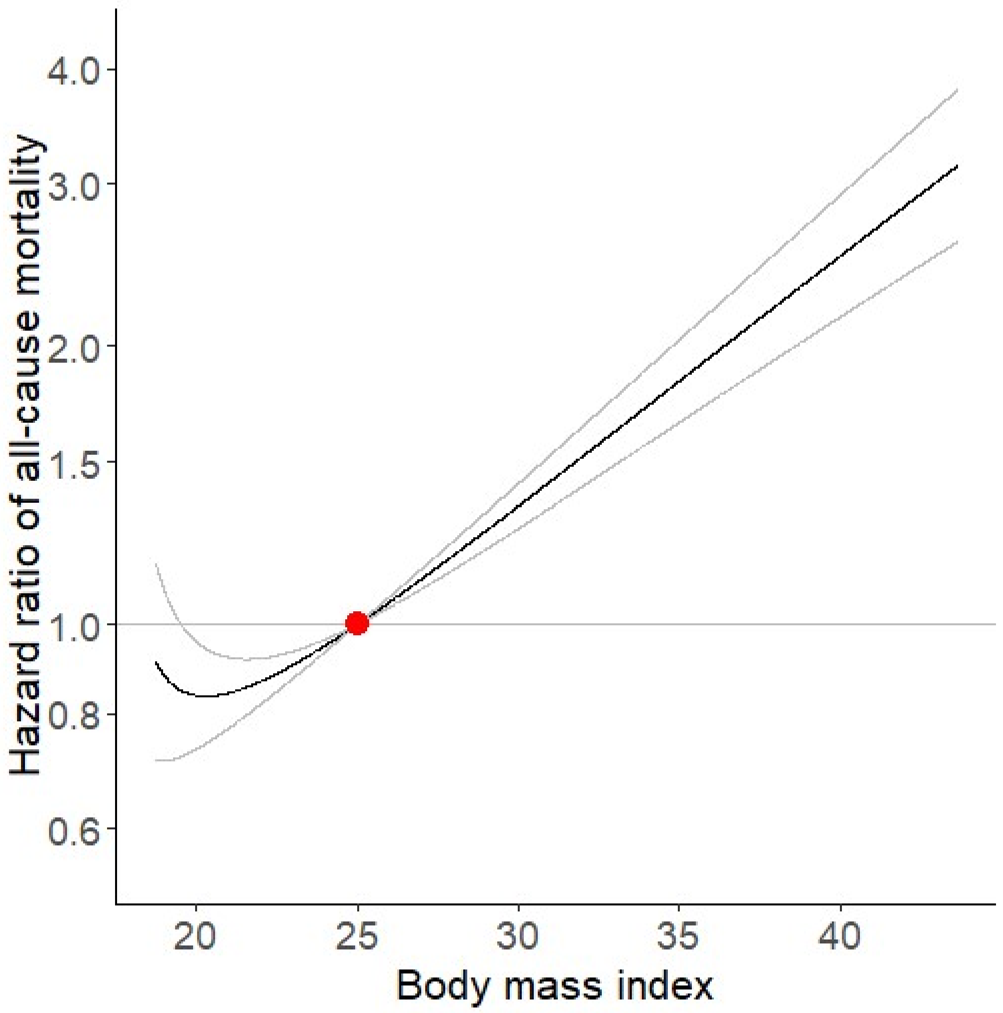

p-value for linearity: 0.99
p-value for trend: 0.32

Supplementary Figure 7: Localized average causal effect estimates (stratum-specific estimates) for all-cause mortality plotted against mean BMI in each stratum estimated in men and women separately. Estimates represent hazard ratios per 1 kg/m^2 increase in genetically-predicted BMI. Error bars represent 95% confidence intervals.

A) HUNT, men only:

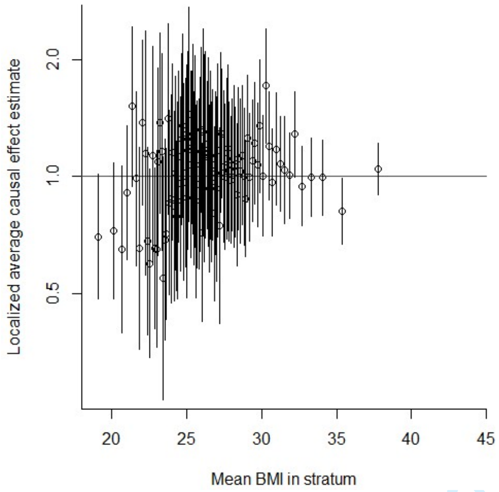

B) HUNT, women only:

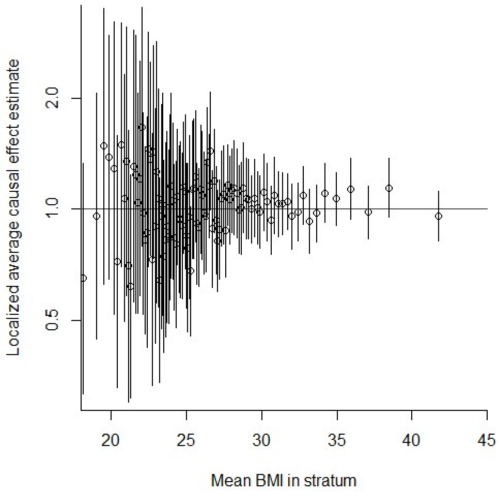

C) UK Biobank, men only:

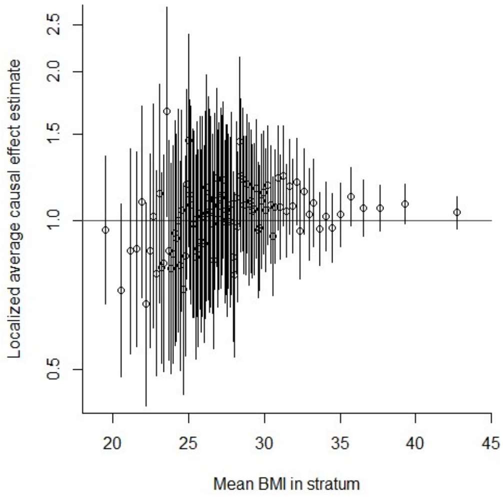

D) UK Biobank, women only:

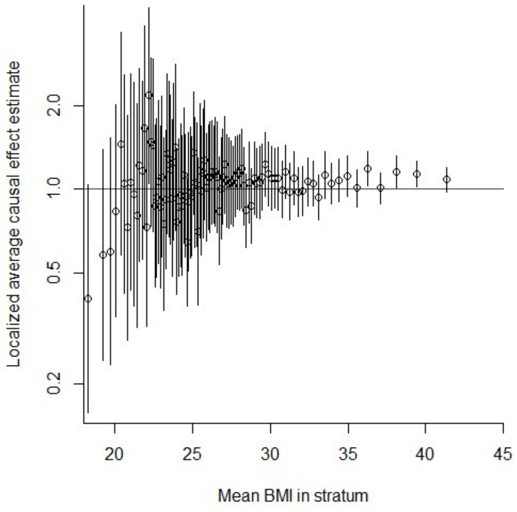

For peer review only - http://bmjopen.bmj.com/site/about/guidelines.xhtml

Supplementary Figure 8: Localized average causal effect estimates (stratum-specific estimates) for cause-specific mortality plotted against mean BMI in each stratum. Estimates represent hazard ratios per 1 kg/m^2 increase in genetically-predicted BMI. Error bars represent 95% confidence intervals.

A: Cardiovascular mortality

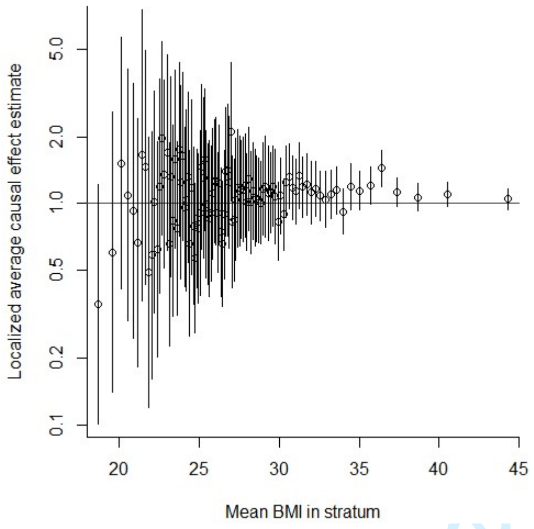

B: Cancer mortality

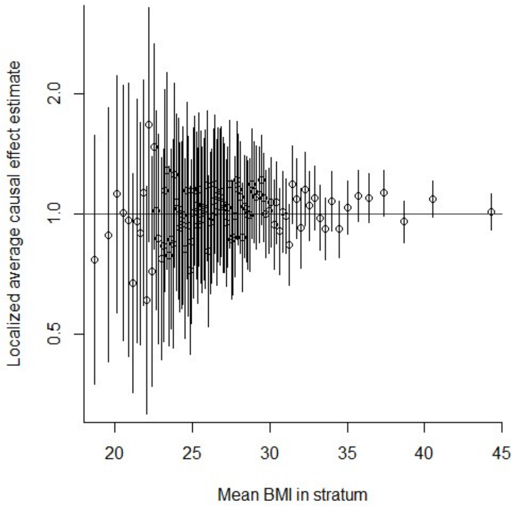

C: Other (non-cardiovascular, non-cancer) mortality

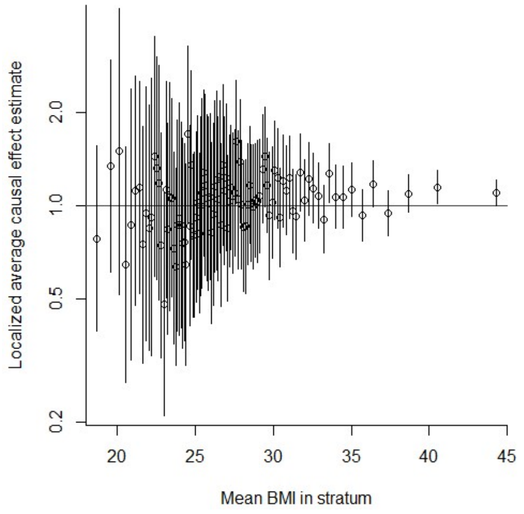

For peer review only - http://bmjopen.bmj.com/site/about/guidelines.xhtml

STROBE-MR checklist of recommended items to address in reports of Mendelian randomization studies[1 2]

Item No.	Section	Checklist item	Page No.	Relevant text from manuscript
1	**TITLE and ABSTRACT**	Indicate Mendelian randomization (MR) as the study's design in the title and/or the abstract if that is a main purpose of the study	1-2	Title: "Body mass index and all-cause mortality in HUNT and UK Biobank studies: revised non-linear Mendelian randomization analyses"<br>Abstract: "**Objectives**: To estimate the shape of the causal relationship between body mass index (BMI) and mortality risk in a Mendelian randomization framework."
	**INTRODUCTION**			
2	**Background**	Explain the scientific background and rationale for the reported study. What is the exposure? Is a potential causal relationship between exposure and outcome plausible? Justify why MR is a helpful method to address the study question	2,4	Abstract: "To estimate the shape of the causal relationship between body mass index (BMI) and mortality risk in a Mendelian randomization framework."<br>Introduction: "The epidemiological relationship between BMI and mortality is complex, with many observational studies conducted in Western countries showing a J-shaped relationship between BMI and mortality risk."<br>"However, such findings may not reflect the causal relationship between BMI and mortality, as observation association are influenced by confounding and reverse causation."
3	**Objectives**	State specific objectives clearly, including pre-specified causal hypotheses (if any). State that MR is a method that, under specific assumptions, intends to estimate causal effects	2,4	Abstract: "To estimate the shape of the causal relationship between body mass index (BMI) and mortality risk in a Mendelian randomization framework."<br>Introduction: "We first investigate whether the constant genetic effect assumption holds for this example, and explore the validity of the instrumental variable assumptions in strata of the population. We then present non-linear Mendelian randomization findings from the doubly-ranked stratification method."<br>"Hence, associations between genetic predictors of BMI and mortality provide insights into the causal effect of BMI on mortality."

For peer review only - http://bmjopen.bmj.com/site/about/guidelines.xhtml

	**METHODS**			
4	**Study design and data sources**	Present key elements of the study design early in the article. Consider including a table listing sources of data for all phases of the study. For each data source contributing to the analysis, describe the following:		
	a)	Setting: Describe the study design and the underlying population, if possible. Describe the setting, locations, and relevant dates, including periods of recruitment, exposure, follow-up, and data collection, when available.	6	See sections "The HUNT study" and "The UK Biobank study" (some of this detail is in reference 19, as indicated: "We present abbreviated descriptions of the datasets included in the analysis; detailed descriptions are in the original paper [19]").
	b)	Participants: Give the eligibility criteria, and the sources and methods of selection of participants. Report the sample size, and whether any power or sample size calculations were carried out prior to the main analysis	6	See sections "The HUNT study" and "The UK Biobank study" (detail is in reference 19: "We present abbreviated descriptions of the datasets included in the analysis; detailed descriptions are in the original paper [19]").
	c)	Describe measurement, quality control and selection of genetic variants	6	See section "SNPs and genetic score used as instrumental variables"
	d)	For each exposure, outcome, and other relevant variables, describe methods of assessment and diagnostic criteria for diseases	NA	See reference 19 for detail.
	e)	Provide details of ethics committee approval and participant informed consent, if relevant	7	See section "Ethical Approval" and "Patient Involvement"
5	**Assumptions**	Explicitly state the three core IV assumptions for the main analysis (relevance, independence and exclusion restriction) as well assumptions for any additional or sensitivity analysis	4,6	Introduction: "According to Mendel's laws of inheritance, genetic variants should be uncorrelated with traits that they do not affect, and hence should be independent of potential confounders"<br>Methods: "We assessed the instrumental variable assumptions by estimating associations between the genetic score and various traits that are competing risk factors in UK Biobank: smoking status (ever versus never), alcohol status (current versus other), education level (post-secondary or higher versus other), occupation (currently employed versus other), age at recruitment, and sex."
6	**Statistical methods: main analysis**	Describe statistical methods and statistics used	6-7	See Section "Statistical analyses".

For peer review only - http://bmjopen.bmj.com/site/about/guidelines.xhtml

		a)	Describe how quantitative variables were handled in the analyses (i.e., scale, units, model)	7	See Section “Statistical analyses”.
		b)	Describe how genetic variants were handled in the analyses and, if applicable, how their weights were selected	6	See Section “SNPs and genetic score used as instrumental variables”
		c)	Describe the MR estimator (e.g. two-stage least squares, Wald ratio) and related statistics. Detail the included covariates and, in case of two-sample MR, whether the same covariate set was used for adjustment in the two samples	7	See Section “Statistical analyses”:<br>“We then calculated the linear Mendelian randomization estimate, referred to as a localized average causal effect (LACE), in each stratum of the population as a ratio of coefficients: the association of the genetic score with the outcome divided by the associations of the genetic score with the exposure. associations with the exposure (BMI) were obtained from linear regression; associations with the outcome (mortality) were obtained from Cox proportional hazards regression, using age as the timescale. All associations were adjusted for age, age-squared, sex, and centre (for UK Biobank).”
		d)	Explain how missing data were addressed	6	“We excluded participants without data on BMI or genetic variants for BMI, leaving 56,150 individuals for analysis.”
		e)	If applicable, indicate how multiple testing was addressed	N/A	We did not account for multiple testing, as the presentation of p-values is not a major aspect of the manuscript.
7	**Assessment of assumptions**		Describe any methods or prior knowledge used to assess the assumptions or justify their validity	6	See Section “SNPs and genetic score used as instrumental variables”:<br>“We assessed the instrumental variable assumptions by estimating associations between the genetic score and various traits that are competing risk factors in UK Biobank: smoking status (ever versus never), alcohol status (current versus other), education level (post-secondary versus other), occupations (currently employed versus other), age at recruitment, and sex.
8	**Sensitivity analyses and additional analyses**		Describe any sensitivity analyses or additional analyses performed (e.g. comparison of effect estimates from different approaches, independent replication, bias analytic techniques, validation of instruments, simulations)	7	Sensitivity Analyses: “In brief, first we divided the sample into 100 strata using the doubly-ranked method. For comparison, we also present results from stratification using the residual method for the primary outcome of all-cause mortality.”<br>Additional analyses: Testing for linearity:

For peer review only - http://bmjopen.bmj.com/site/about/guidelines.xhtml

					"We report two tests for non-linearity: a linearity test, which assesses whether a non-linear model fits the LACE estimates better than a linear model, and a trend test, which tests for a linear trend amongst the LACE estimates. "
9	**Software and pre-registration**				
		a)	Name statistical software and package(s), including version and settings used	7	"All statistical analyses were performed in R (version 4.3.1), and non-linear Mendelian randomisation analyses were performed using the SUMnlmr package [25]."
		b)	State whether the study protocol and details were pre-registered (as well as when and where)	N/A	The study protocol was not pre-registered.
	**RESULTS**				
10	**Descriptive data**				
		a)	Report the numbers of individuals at each stage of included studies and reasons for exclusion. Consider use of a flow diagram		All numbers provided in Table 1.
		b)	Report summary statistics for phenotypic exposure(s), outcome(s), and other relevant variables (e.g. means, SDs, proportions)		All numbers provided in Table 1.
		c)	If the data sources include meta-analyses of previous studies, provide the assessments of heterogeneity across these studies		N/A – we present results from the HUNT and UK Biobank studies separately, and do not attempt to meta-analyse or otherwise combine results.
		d)	For two-sample MR:<br>i. Provide justification of the similarity of the genetic variant-exposure associations between the exposure and outcome samples<br>ii. Provide information on the number of individuals who overlap between the exposure and outcome studies		N/A – not two-sample MR.
11	**Main results**				
		a)	Report the associations between genetic variant and exposure, and between genetic variant and outcome, preferably on an interpretable scale		Genetic associations with exposure are shown in Supplementary Figure 1.<br>Scaled genetic association with outcome are shown in Supplementary Figures 3, 5, and 6.
		b)	Report MR estimates of the relationship between exposure and outcome, and the measures of uncertainty from the MR analysis, on an interpretable scale, such as odds ratio or relative risk per SD difference		MR estimates are shown directly in Supplementary Figures 3, 5, and 6. Smoothed versions of these associations are shown in Figures 2, 3, and 4.

For peer review only - http://bmjopen.bmj.com/site/about/guidelines.xhtml

		c)	If relevant, consider translating estimates of relative risk into absolute risk for a meaningful time period		N/A
		d)	Consider plots to visualize results (e.g. forest plot, scatterplot of associations between genetic variants and outcome versus between genetic variants and exposure)		N/A
12	**Assessment of assumptions**				
		a)	Report the assessment of the validity of the assumptions	8	See section "Assessment of instrument validity"
		b)	Report any additional statistics (e.g., assessments of heterogeneity across genetic variants, such as I^2, Q statistic or E-value)	6	F statistics are provided in the Methods section under "SNPs and genetic score used as instrumental variables.<br>Nonlinear tests are reported in Figures 2, 3, and 4.
13	**Sensitivity analyses and additional analyses**				
		a)	Report any sensitivity analyses to assess the robustness of the main results to violations of the assumptions		See Figure 2: residual versus ranked stratification.
		b)	Report results from other sensitivity analyses or additional analyses		Figure 2: residual versus ranked stratification<br>Figure 3: results stratified by sex<br>Figure 4: exploring different cause-specific mortality subtypes.
		c)	Report any assessment of direction of causal relationship (e.g., bidirectional MR)		N/A, directionality not considered.
		d)	When relevant, report and compare with estimates from non-MR analyses	11	In Discussion, paragraph beginning "Previous investigations into the non-linear shape of the causal relationship between BMI and mortality have used a variety of approaches." and in particular "Carslake *et al* performed an instrumental variable analysis to investigate the impact of BMI on mortality in the HUNT study, but using offspring BMI as an instrument for the BMI".
		e)	Consider additional plots to visualize results (e.g., leave-one-out analyses)		N/A
	**DISCUSSION**				
14	**Key results**		Summarize key results with reference to study objectives	10	Results are summarized in the Discussion section:

For peer review only - http://bmjopen.bmj.com/site/about/guidelines.xhtml

					"While the curves generally display a J-shaped relationship, evidence for a harmful average effect of decreased BMI on mortality at low BMI levels was limited. Evidence supporting non-linearity from the doubly-ranked method was unconvincing for all mortality outcomes."<br>Also see Abstract / Results.
15	**Limitations**		Discuss limitations of the study, taking into account the validity of the IV assumptions, other sources of potential bias, and imprecision. Discuss both direction and magnitude of any potential bias and any efforts to address them	11-12	Limitations are clearly discussed in the Discussion section, paragraph beginning "However, there are important limitations…" Several potential limitations are discussed, including validity of instruments, pleiotropy, bias due to selection bias, representativeness, and reverse causation.
16	**Interpretation**				
		a)	Meaning: Give a cautious overall interpretation of results in the context of their limitations and in comparison with other studies	10	Interpretation of results is provided in the Discussion section: "In conclusion, non-linear Mendelian randomization analyses using the doubly-ranked stratification method provide strong evidence for harmful effects of increased BMI on mortality above 25 kg/m2. Evidence for a harmful effect of low BMI was only present in some analyses, and where present, only below 20 kg/m2.".<br>Also see Abstract/Conclusion.
		b)	Mechanism: Discuss underlying biological mechanisms that could drive a potential causal relationship between the investigated exposure and the outcome, and whether the gene-environment equivalence assumption is reasonable. Use causal language carefully, clarifying that IV estimates may provide causal effects only under certain assumptions		N/A (mechanisms linking BMI and mortality have been discussed elsewhere at length, and evidence for specific mechanisms is not provided here).
		c)	Clinical relevance: Discuss whether the results have clinical or public policy relevance, and to what extent they inform effect sizes of possible interventions	10	Discussion: "Our findings challenge the "obesity paradox" consensus from observational epidemiology that increases in BMI might reduce risk of mortality for a substantial proportion of the population."
17	**Generalizability**		Discuss the generalizability of the study results (a) to other populations, (b) across other exposure periods/timings, and (c) across other levels of exposure	11	Discussion / Limitations: "Our findings may therefore not be applicable to other populations. "

For peer review only

	**OTHER INFORMATION**			
18	**Funding**	Describe sources of funding and the role of funders in the present study and, if applicable, sources of funding for the databases and original study or studies on which the present study is based	1	See "Funding statement"
19	**Data and data sharing**	Provide the data used to perform all analyses or report where and how the data can be accessed, and reference these sources in the article. Provide the statistical code needed to reproduce the results in the article, or report whether the code is publicly accessible and if so, where	31	See "Data sharing statement"
20	**Conflicts of Interest**	All authors should declare all potential conflicts of interest	1	Provided under "Competing interest statement"

This checklist is copyrighted by the Equator Network under the Creative Commons Attribution 3.0 Unported (CC BY 3.0) license.

1. Skrivankova VW, Richmond RC, Woolf BAR, Yarmolinsky J, Davies NM, Swanson SA, et al. Strengthening the Reporting of Observational Studies in Epidemiology using Mendelian Randomization (STROBE-MR) Statement. JAMA. 2021;under review.

2. Skrivankova VW, Richmond RC, Woolf BAR, Davies NM, Swanson SA, VanderWeele TJ, et al. Strengthening the Reporting of Observational Studies in Epidemiology using Mendelian Randomisation (STROBE-MR): Explanation and Elaboration. BMJ. 2021;375:n2233.

For peer review only - http://bmjopen.bmj.com/site/about/guidelines.xhtml

BMJ Open

Body mass index and all-cause mortality in HUNT and UK Biobank studies: revised non-linear Mendelian randomization analyses

Journal:	*BMJ Open*
Manuscript ID	bmjopen-2023-081399.R3
Article Type:	Original research
Date Submitted by the Author:	01-May-2024
Complete List of Authors:	Burgess, Stephen; University of Cambridge, MRC Biostatistics Unit; University of Cambridge, Cardiovascular Epidemiology Unit, Department of Public Health and Primary Care<br>Sun, Yi-Qian; Norges teknisk-naturvitenskapelige universitet, Department of Clinical and Molecular Medicine (IKOM)<br>Zhou, Ang; University of Cambridge, MRC Biostatistics Unit; University of South Australia, Australian Centre for Precision Health<br>Buck, Christopher; University of Cambridge, MRC Biostatistics Unit<br>Mason, Amy M; University of Cambridge, Department of Public Health and Primary Care<br>Mai, Xiao-Mei; Norwegian University of Science and Technology, Department of Public Health and Nursing
**Primary Subject Heading**:	Public health
Secondary Subject Heading:	Epidemiology, Genetics and genomics, Public health
Keywords:	Obesity, Observational Study, GENETICS, EPIDEMIOLOGY

SCHOLARONE™
Manuscripts

I, the Submitting Author has the right to grant and does grant on behalf of all authors of the Work (as defined in the below author licence), an exclusive licence and/or a non-exclusive licence for contributions from authors who are: i) UK Crown employees; ii) where BMJ has agreed a CC-BY licence shall apply, and/or iii) in accordance with the terms applicable for US Federal Government officers or employees acting as part of their official duties; on a worldwide, perpetual, irrevocable, royalty-free basis to BMJ Publishing Group Ltd ("BMJ") its licensees and where the relevant Journal is co-owned by BMJ to the co-owners of the Journal, to publish the Work in this journal and any other BMJ products and to exploit all rights, as set out in our licence.

The Submitting Author accepts and understands that any supply made under these terms is made by BMJ to the Submitting Author unless you are acting as an employee on behalf of your employer or a postgraduate student of an affiliated institution which is paying any applicable article publishing charge ("APC") for Open Access articles. Where the Submitting Author wishes to make the Work available on an Open Access basis (and intends to pay the relevant APC), the terms of reuse of such Open Access shall be governed by a Creative Commons licence – details of these licences and which Creative Commons licence will apply to this Work are set out in our licence referred to above.

Other than as permitted in any relevant BMJ Author's Self Archiving Policies, I confirm this Work has not been accepted for publication elsewhere, is not being considered for publication elsewhere and does not duplicate material already published. I confirm all authors consent to publication of this Work and authorise the granting of this licence.

For peer review only

Body mass index and all-cause mortality in HUNT and UK Biobank studies: revised non-linear Mendelian randomization analyses

Stephen Burgess[1,2,3], Yi-Qian Sun[4,5,6], Ang Zhou[1,7], Christopher Buck[1], Amy M Mason[2,3], Xiao-Mei Mai[8]

1. Medical Research Council Biostatistics Unit, University of Cambridge, Cambridge, UK.
2. Cardiovascular Epidemiology Unit, Department of Public Health and Primary Care, University of Cambridge, Cambridge, UK.
3. Victor Phillip Dahdaleh Heart and Lung Research Institute, University of Cambridge, Cambridge, UK.
4. Department of Clinical and Molecular Medicine, Faculty of Medicine and Health Science, Norwegian University of Science and Technology, Trondheim, Norway.
5. Department of Pathology, Clinic of Laboratory Medicine, St. Olavs Hospital, Trondheim University Hospital, Trondheim, Norway.
6. Center for Oral Health Services and Research Mid-Norway (TkMidt), Trondheim, Norway.
7. Australian Centre for Precision Health, Unit of Clinical and Health Sciences, University of South Australia, Adelaide, Australia.
8. Department of Public Health and Nursing, Faculty of Medicine and Health Science, Norwegian University of Science and Technology, Trondheim, Norway.

Corresponding author: Stephen Burgess (sb452@medschl.cam.ac.uk)

Word count: 299 (abstract), 4357 (main text).

Key words: Obesity paradox, reverse causation, genetic epidemiology, causal inference, doubly-ranked stratification.

For peer review only

For peer review only - http://bmjopen.bmj.com/site/about/guidelines.xhtml

ABSTRACT

Objectives: To estimate the shape of the causal relationship between body mass index (BMI) and mortality risk in a Mendelian randomization framework.

Design: Mendelian randomization analyses of two prospective population-based cohorts.

Setting: Individuals of European ancestries living in Norway or the United Kingdom (UK).

Participants: 56,150 participants from the Trøndelag Health Study (HUNT) in Norway and 366,385 participants from UK Biobank recruited by postal invitation.

Outcomes: All-cause mortality and cause-specific mortality (cardiovascular, cancer, non-cardiovascular non-cancer).

Results: A previously published non-linear Mendelian randomization analysis of these data using the residual stratification method suggested a J-shaped association between genetically-predicted BMI and mortality outcomes with the lowest mortality risk at a BMI of around 25 kg/m^2. However, the "constant genetic effect" assumption required by this method is violated. The re-analysis of these data using the more reliable doubly-ranked stratification method provided some indication of a J-shaped relationship, but with much less certainty as there was less precision in estimates at the lower end of the BMI distribution. Evidence for a harmful effect of reducing BMI at low BMI levels was only present in some analyses, and where present, only below 20 kg/m^2. A harmful effect of increasing BMI for all-cause mortality was evident above 25 kg/m^2, for cardiovascular mortality above 24 kg/m^2, for cancer mortality above 30 kg/m^2, and for non-cardiovascular non-cancer mortality above 26 kg/m^2. In UK Biobank, the association between genetically-predicted BMI and mortality at high BMI levels was stronger in women than in men.

Conclusion: This research challenges findings from previous conventional observational epidemiology and Mendelian randomization investigations that the lowest level of mortality risk is at a BMI level of around 25 kg/m^2. Our results provide some evidence that reductions in BMI will increase mortality risk for a small proportion of the population, and clear evidence that increases in BMI will increase mortality risk for those with BMI above 25 kg/m^2.

For peer review only

For peer review only - http://bmjopen.bmj.com/site/about/guidelines.xhtml

Strengths and limitations of the study

- Mendelian randomization design minimizes bias due to confounding and reverse causation
- Large sample sizes enable powerful analyses even in low body mass index (BMI) individuals
- Validity of the genetic variants as instrumental variables cannot be verified
- Bias due to selection could be non-negligible and could vary across strata
- All estimates are averaged across a stratum of the population; individual effects of raising or lowering BMI may vary between individuals

For peer review only

For peer review only - http://bmjopen.bmj.com/site/about/guidelines.xhtml

INTRODUCTION

Body mass index (BMI) is a convenient and accessible measure of obesity. The epidemiological relationship between BMI and mortality is complex, with many observational studies conducted in Western countries showing a J-shaped relationship between BMI and mortality risk in the general population, such that mortality risk is lowest for those in the upper normal weight (BMI 20.0-24.9 kg/m^2)(1-3) or even the overweight (BMI 25.0-29.9 kg/m^2)(4-6) category rather than the lower normal weight (BMI 18.5-19.9 kg/m^2) or underweight category (BMI <18.5 kg/m^2). However, such findings may not reflect the causal relationship between BMI and mortality, as observational associations are influenced by confounding and reverse causation.

Mendelian randomization is an epidemiological technique for the analysis of observational data designed to avoid bias due to confounding and reverse causation(7). Rather than assessing associations between BMI and mortality directly, we assess associations between genetic variants that predict BMI levels and mortality risk(8). This is analogous to the analysis of a randomized controlled trial for weight loss(9), which would assess associations between the randomized intervention and the outcome, rather than associations with measured BMI in trial participants. According to Mendel's laws of inheritance, genetic variants should be uncorrelated with traits that they do not affect, and hence should be independent of potential confounders. As genetic variants are determined at conception, genetic associations should be protected from reverse causation(10). These properties mean that genetic variants are plausible instrumental variables; an instrumental variable is a variable that behaves as if it has been randomized in the population(11). Hence, associations between genetic predictors of BMI and mortality provide insights into the causal effect of BMI on mortality.

Non-linear Mendelian randomization is an extension of standard Mendelian randomization to investigate the shape of the causal relationship between an exposure and an outcome(12). A typical randomized trial estimates an average causal effect, representing the average impact of a population-wide shift in the distribution of an exposure; similarly, a standard Mendelian randomization analysis estimates a population-averaged causal effect(13). A widely used method for non-linear Mendelian randomization stratifies the population based on levels of the exposure, and estimates 'localized average causal effects', representing the average impact of a population-wide shift in the distribution of the exposure for that stratum of the population(13).

However, stratifying the population such that the instrumental variable assumptions still hold in the strata of the population is tricky. Stratifying on the exposure directly would break randomization, as exposure levels are influenced by the instrumental variables. Hence an individual in the lowest stratum of the population defined by the exposure with a genetic predisposition to high values of the exposure would be more likely to have low values of the confounders. This is an example of collider bias(14): the exposure is a common effect of the instrument and exposure—outcome confounders, and so stratification on the exposure leads to a conditional association between the instrument and confounders. The initial proposal for non-linear Mendelian randomization was to stratify on the residual exposure, defined as the residual from regression of the exposure on the instrumental variables(13). The residual exposure is independent of the instrumental variables by construction, and the instrumental variables will be independent of confounders within strata of the residual exposure under a "constant genetic effect" assumption(15). However, if the effect of the genetic instrumental variables on the exposure varies between individuals, then this residual stratification method can lead to severe bias in stratum-specific estimates(16, 17).

For peer review only - http://bmjopen.bmj.com/site/about/guidelines.xhtml

An alternative method, known as the doubly-ranked method, allows the instrumental variable effects on the exposure to vary between individuals provided that a weaker “rank-preserving assumption” holds(18). The rank-preserving assumption states that the ranking of individuals according to their exposure levels would be the same at all levels of the instrumental variable. That is, if the genotype of all individuals were set to any fixed value, the order of individuals according to their exposure levels would be the same. The doubly-ranked method is implemented by first ranking individuals according to their level of the instrument to form pre-strata with similar values of the instrument, and then ranking individuals within each pre-stratum according to their level of the exposure, so that the lowest stratum contains the individual with the lowest exposure value from pre-stratum 1, the individual with the lowest exposure value from pre-stratum 2, the individual with the lowest exposure value from pre-stratum 3, and so on. This method has been shown to be more reliable than the residual method in simulation studies(17, 18).

In a previous paper, we presented evidence for the effect of BMI on mortality from non-linear Mendelian randomization using the residual stratification method in the Norwegian Trøndelag Health (HUNT) and United Kingdom (UK) Biobank studies. A J-shaped association between genetically-predicted BMI and mortality was observed in each study population overall, although a monotonic increasing association (that is, an always-increasing association) was seen in never-smokers(19). Here, we present a re-analysis of the same datasets using the doubly-ranked stratification method. We first investigate whether the constant genetic effect assumption holds for this example, and explore the validity of the instrumental variable assumptions in strata of the population. We then present non-linear Mendelian randomization findings from the doubly-ranked stratification method.

For peer review only

METHODS

We present abbreviated descriptions of the datasets included in the analysis; detailed descriptions are in the original paper(19).

The HUNT study

We used data from the second wave (1995-1997) of the HUNT study on 65,229 individuals living in the northern part of Trøndelag and over the age of 20 years(20). Participants were followed up until 15 June 2023 or their date of death. The original paper only considered mortality outcomes up to April 2015. By extending the follow-up period, we increase the number of mortality events considered in the analysis from 12,015 to 18,836. We excluded participants without data on BMI or genetic variants for BMI, leaving 56,150 individuals for analysis.

The UK Biobank study

The UK Biobank cohort comprises around 500,000 participants (94% of self-reported European ancestry) aged 40 to 69 years at baseline. They were recruited between 2006-2010 in 22 assessment centres throughout the UK, and followed up until 28 February 2021 or their date of death(21). Again, the original paper only considered mortality outcomes up to February 2016. By extending the follow-up period, we increase the number of mortality events considered in the analysis from 10,344 to 25,021. We performed detailed quality control procedures on UK Biobank participants and on genetic variants as described previously(22). In total, 366,385 unrelated European ancestry participants were included in analyses.

SNPs and genetic score used as instrumental variables

77 single nucleotide polymorphisms (SNPs) were selected as candidate instrumental variables for BMI based on European sex-combined analyses in a genome-wide association study of the Genetic Investigation of ANthropometric Traits (GIANT) consortium(23). Two of these variants (rs12016871 and rs2033732) were not available in the HUNT study, and a further two variants (rs13021737 and rs16951275) were excluded from the analyses due to association with smoking status in the HUNT study. An externally-weighted score was calculated for each individual by multiplying the number of BMI-increasing alleles the individual carries by the variant's association with BMI from the GIANT study (**Supplementary Table 1**), and summing across the remaining 73 variants. Overall, the genetic score explained 2.0% and 1.6% of the variance in BMI in the HUNT and UK Biobank studies respectively, corresponding to F-statistics of 1121 and 5964. As a supplementary analysis, we repeated analyses for all-cause mortality using an extended genetic score comprising 633 variants taken from a more recent genome-wide association study of the GIANT consortium, which included UK Biobank (24). The variant list was taken from Verkouter *et al (25)*, who pruned the initial set of reported variants to exclude correlated variants.

We assessed the instrumental variable assumptions within strata by estimating associations between the genetic score and various traits that are competing risk factors: smoking status (ever versus never), alcohol status (current versus other), education level (post-secondary or higher versus other), occupation (currently employed versus other), age at recruitment, and sex.

Study design

We performed non-linear Mendelian randomization analyses to estimate the shape of the association between genetically-predicted BMI and the outcome. Our primary analysis considered all-cause mortality as the outcome. We also conducted subgroup analyses considering men and

For peer review only - http://bmjopen.bmj.com/site/about/guidelines.xhtml

women separately. In addition, we studied associations with cause-specific mortality events (cardiovascular, cancer, and non-cardiovascular non-cancer) in UK Biobank. These were defined based on International Classification of Diseases, Tenth Revision (ICD-10) codes (see **Supplementary Table 2** for details). We did not perform analyses stratified by smoking status (as presented in the original paper), as smoking status is influenced by BMI(26), and hence this stratification may introduce collider bias.

Statistical analyses

To estimate the non-linear relationship between genetically-predicted BMI and mortality risk, a fractional polynomial method was applied(12). In brief, first we divided the sample into 100 strata using the doubly-ranked method(18). For comparison, we also present results from stratification using the residual method for the primary outcome of all-cause mortality. We then calculated the linear Mendelian randomization estimate, referred to as a localized average causal effect (LACE), in each stratum of the population as a ratio of coefficients: the association of the genetic score with the outcome divided by the association of the genetic score with the exposure. Associations with the exposure (BMI) were obtained from linear regression; associations with the outcome (mortality) were obtained from Cox proportional hazards regression, using age as the timescale. While Cox proportional hazards regression is a semi-parametric method, the estimated non-parametric baseline hazard function is a function of the timescale, not the exposure value. All associations were adjusted for age, age-squared, sex, and centre (for UK Biobank).

We performed meta-regression of the LACE estimates against the mean of the exposure in each stratum in a flexible semiparametric framework using the derivative of fractional polynomial models of degrees 1 and 2. Under the Mendelian randomization assumptions, the slope of the curve represents the average causal effect of the exposure on the outcome for the stratum with that exposure level. A positive causal effect is evident at a particular exposure level when the lower and upper confidence limits for the curve both have a positive slope; and similarly an inverse causal effect is evident if both confidence limits have a negative slope. The reference point in analyses is set to 25 kg/m^2; however, this reference point is arbitrarily chosen, and the key indicator of an increasing or decreasing effect is not whether confidence intervals include or exclude the reference point, but rather whether the slope of the curve is positive or negative at a given exposure value.

We report two tests for non-linearity: a linearity test, which assesses whether a non-linear model fits the LACE estimates better than a linear model, and a trend test, which tests for a linear trend amongst the LACE estimates. Rejection of the linearity test indicates that a linear model is a suboptimal fit to the data; failure to reject means any improvement in fit for the best fitting non-linear model over the linear model is no more than would be expected due to chance alone.

As LACE estimates from the doubly-ranked method can be sensitive to specification of the analytic sample, we repeated analyses 100 times for each dataset omitting a small number of individuals in each iteration (12 individuals were removed at random in each iteration), and then combined estimates across iterations using Rubin's rules.

All statistical analyses were performed in R (version 4.3.1), and non-linear Mendelian randomization analyses were performed using the SUMnlmr package(27).

Patient and public involvement

No patients were involved in setting the research question or the outcome measures, nor were they involved in the design or implementation of the study. No patients were asked to advise on

For peer review only - http://bmjopen.bmj.com/site/about/guidelines.xhtml

interpretation or writing up of results. There are no specific plans to disseminate the results of the research to study participants or the relevant patient community.

For peer review only

RESULTS

Study populations

Baseline characteristics of the participants are provided in **Table 1**. Genetic associations with BMI in strata of the population defined by the doubly-ranked method are displayed in **Supplementary Figure 1**, and the BMI distribution in strata of the population is given in **Supplementary Table 3**. Per 1 standard deviation increase in the genetic score, BMI was 0.358 kg/m^2 greater in the lowest percentile of participants in the HUNT study, and 1.126 kg/m^2 greater in the highest percentile. Equivalent values for UK Biobank were 0.232 kg/m^2 in the lowest percentile and 1.645 kg/m^2 in the highest percentile. It is clear that the genetic associations with BMI differ strongly at different levels of BMI, and so the assumption required for the residual stratification method is violated. We note that similar estimates of the genetic associations with the exposure from the residual stratification method are unreliable, as these estimates from the residual method are similar even if the genetic effect on the exposure varies in the population(18).

Assessment of instrument validity

Associations with traits that are competing risk factors in strata of UK Biobank are displayed in **Figure 1**. As previously observed in the literature for other exposures(28), there are clear patterns in genetic associations with age and sex across strata. While there is no overall association of the genetic score with age or sex, there are associations in several of the strata.

As it is logically impossible that autosomal genetic variants can affect either age or sex, and as age and sex are typically measured without error, these associations must represent the effect of selection into the UK Biobank sample. While concerning, as we adjust for age and sex in our Mendelian randomization analyses, these associations will not lead to strong bias in our causal estimates (see **Supplementary Material** for a simulation study investigation into bias mitigation by adjustment for confounders that are predictors of selection). For other traits, both the magnitude of associations and patterns in associations are less strong, particularly for education level and occupation, which are important measures of social class. For alcohol status and smoking status, it is impossible to distinguish between genetic associations that reflect pleiotropic or selection effects and those that reflect a causal effect of the exposure (sometimes called "vertical pleiotropy"); the latter do not violate the instrumental variable assumptions. Hence both the associations and any pattern of associations may reflect violations of the instrumental variable assumptions or selection bias, but they could also reflect downstream effects of BMI which vary across strata.

Corresponding associations in the HUNT study are shown in **Supplementary Figure 2**. While there are some patterns in the stratum associations with age and sex, these are less pronounced than for UK Biobank, potentially due to differential selection being less strong in the HUNT study.

Comparison of results from residual and doubly-ranked methods

The shape of the association between genetically-predicted BMI and all-cause mortality is displayed in **Figure 2**, and stratum-specific LACE estimates are provided in **Supplementary Figure 3**. We note that **Figure 2** and **Supplementary Figure 3** differ in their presentation of these data; **Figure 2** plots the estimated association between genetically-predicted BMI values and mortality risk with respect to the reference exposure value, whereas **Supplementary Figure 3** plots the estimated effect of the exposure on mortality risk in each stratum. A smoothed version of the log transformed estimates in **Supplementary Figure 3** would represent the derivative of the curve shown in **Figure 2**.

For peer review only - http://bmjopen.bmj.com/site/about/guidelines.xhtml

When stratifying the population using the residual method, there is a distinct J-shaped relationship between BMI and mortality, particularly in the UK Biobank study, with minimum risk at a BMI level of around 25 kg/m^2. However, as discussed above, results from the residual stratification method are unreliable here. In contrast, when stratifying using the doubly-ranked method, the relationship between BMI and mortality is far more uncertain at the lower end of the BMI distribution. In the HUNT study, the association is mildly J-shaped, with a null slope up to a BMI of around 28 kg/m^2, and a positive slope above this level. For UK Biobank, there is some indication of an upturn in mortality risk at very low levels of BMI (the slope of the curve is negative below 20 kg/m^2), but otherwise the relationship is flat (and compatible with a null effect) up to a BMI of around 25 kg/m^2, and has an increasing positive slope above this level. The linearity and trend tests for the residual method indicate evidence supporting non-linearity for UK Biobank ($p_{linearity} = 5\times10^{-4}$, $p_{trend} = 2\times10^{-5}$). In contrast, these tests for the more reliable doubly-ranked method do not provide compelling evidence supporting non-linearity (**Figure 2**).

Similar results were observed when inverse-normal rank transforming the exposure (**Supplementary Figure 4**). When dividing the population into 50 strata, results were similar in both studies. When dividing into 200 strata, results were similar for UK Biobank, but differed for the HUNT study, as the best-fitting curve was monotone with no J-shaped upturn at the lower end of the BMI distribution (**Supplementary Figure 5**). When using the extended genetic score (633 variants) in UK Biobank, a pattern of results similar to the main analysis was observed, but with narrower confidence intervals as the extended score explains more variance in BMI (**Supplementary Figure 6**). There was still some indication of a J-shaped curve, although minimum mortality risk was at a BMI level of 20-21 kg/m^2, and evidence for an upturn in risk at low BMI levels was weaker.

Sex-stratified analyses

Sex-stratified analyses from the doubly-ranked method are presented in **Figure 3**, and stratum-specific LACE estimates are provided in **Supplementary Figure 7**. In each case, there is some indication of a J-shaped relationship, but an upturn in mortality risk is only evident below 20 kg/m^2 (below 22 kg/m^2 for men in the HUNT study). Otherwise, estimates are generally compatible with the null up to a BMI of 25 kg/m^2, and positive above this level. Evidence for a harmful effect of increases in BMI for both men and women is weaker in the HUNT study, although a harmful effect above 30 kg/m^2 is evident for men. The slope above 25 kg/m^2 is steeper for women than men in UK Biobank. The linearity and trend tests do not provide compelling evidence supporting non-linearity in any analysis (**Figure 3**).

Cause-specific mortality

Analyses investigating cause-specific mortality from the doubly-ranked method in UK Biobank are presented in **Figure 4**, and stratum-specific LACE estimates are provided in **Supplementary Figure 8**. Again, there is some indication of a J-shaped relationship for each outcome. This is most pronounced for cardiovascular mortality, and less evident for cancer and non-cardiovascular non-cancer mortality. An upturn in risk at low BMI levels was only evident for cardiovascular mortality, and only below 20 kg/m^2. Otherwise, the relationship was compatible with the null up to around 24 kg/m^2 for cardiovascular mortality, 26 kg/m^2 for non-cardiovascular non-cancer mortality, and 30 kg/m^2 for cancer mortality, and positive above this level. The slope above 25 kg/m^2 is steepest for cardiovascular mortality, then non-cardiovascular non-cancer, and finally for cancer mortality. The linearity and trend tests do not provide any evidence supporting non-linearity for any outcome, indicating that a non-linear model does not fit the data significantly better than a linear model for any outcome (**Figure 4**).

For peer review only - http://bmjopen.bmj.com/site/about/guidelines.xhtml

DISCUSSION

In this manuscript, we have investigated the shape of associations between genetically-predicted BMI and mortality outcomes to provide an indication of the causal effect of BMI on mortality risk at different levels of BMI using non-linear Mendelian randomization. While the curves display some signs of a J-shaped relationship, evidence for a harmful average effect of decreased BMI on mortality at low BMI levels was limited. Evidence supporting non-linearity from statistical hypothesis tests for the doubly-ranked method was unconvincing for all mortality outcomes. In UK Biobank, the slope representing the estimated effect of BMI on mortality risk above 25 kg/m^2 was sharper for women than for men, and sharpest for cardiovascular mortality compared with cancer mortality and non-cardiovascular non-cancer mortality. There was evidence for a harmful effect of high BMI on all mortality subtypes.

Our findings challenge "obesity paradox" results from observational epidemiology and previous Mendelian randomization analyses using the residual method, which suggest that increases in BMI might reduce risk of mortality for a substantial proportion of the general population. While the minimum of the estimated curve for all-cause mortality was at a BMI of around 23 kg/m^2 in UK Biobank, the curve was near flat from 22-24 kg/m^2, and was positive from 25 kg/m^2 upwards. Evidence for a harmful effect of decreased BMI at low levels of BMI (as indicated by negative slopes for both the upper and lower confidence limits of the BMI—mortality curve) was only present in some analyses, and only below 20 kg/m^2 when present. Amongst cause-specific mortality outcomes, this was only evident for cardiovascular mortality. In the HUNT study, there was no strong evidence for an effect of BMI on all-cause mortality at low BMI levels in overall analyses, and evidence for a harmful effect of increased BMI above around 28 kg/m^2. A potential reason for the discrepancy is that UK Biobank only contains participants from the age of 40 upwards, whereas the HUNT study includes younger individuals. Hence underweight strata in UK Biobank are more likely to comprise primarily of older, frailer individuals, whereas underweight strata in the HUNT study include more healthy, slim individuals.

Our investigation provides further empirical evidence that the assumptions required for the residual stratification method for non-linear Mendelian randomization are likely to be violated in practice, and that results from the residual method can be substantially different to those from the doubly-ranked method(17). The ratio between the genetic association with BMI in the highest versus lowest percentile group was 3.1 in the HUNT study and 7.1 in UK Biobank, whereas the assumptions required by the residual method imply that these associations should be constant across the distribution of the exposure. Particularly in UK Biobank, the residual method provided strong evidence for non-linearity in the effect of BMI on mortality risk, giving a J-shaped relationship with the minimum risk level at around 25 kg/m^2. This is similar to the confounded association observed in traditional observational studies(2, 3). In contrast, the doubly-ranked method provided little evidence for non-linearity; formal statistical tests for non-linearity did not reject the null hypothesis of linearity in any analysis. While there was some indication of a J-shaped curve in the main analyses, estimates at the lower end of the BMI distribution were imprecise. One reason for this is that genetic associations with BMI were weaker at low levels of BMI in the doubly-ranked method, and so stratum-specific Mendelian randomization estimates are less precise in these strata. In some supplementary analyses using different numbers of strata, evidence for a J-shaped relationship in the HUNT dataset was absent, and the J-shape was less pronounced when using an extended genetic score in UK Biobank.

Previous investigations into the non-linear shape of the causal relationship between BMI and mortality have used a variety of approaches. Wade *et al* used the residual stratification method for

For peer review only - http://bmjopen.bmj.com/site/about/guidelines.xhtml

non-linear Mendelian randomization in UK Biobank(29), which showed a J-shaped relationship similar to the one we observe here. Jenkins *et al* investigated associations between a genetic score for BMI and mortality in UK Biobank(5), considering analyses in the full dataset, and restricted to those with a pre-existing morbidity condition. While they did not observe evidence for non-linearity, the genetic score only explains a small proportion of variance in BMI, and so substantial non-linearity may not be expected on this scale. The difference between average BMI levels at the lowest versus highest decile of the genetic score is less than 2 kg/m^2. Sulc *et al* investigated the effect of BMI on life expectancy (estimated as the mean of parental lifespan) in a Mendelian randomization framework using a polynomial approach similar to the residual method(30), except that instead of stratifying on the residual exposure, this approach constructs a polynomial function of the residual exposure. They showed a linear effect of increased BMI on reduced life expectancy, with no indication of non-linearity. However, this approach also has been shown in simulations to be vulnerable to variation in genetic effects on the exposure(18). Carslake *et al* performed an instrumental variable analysis to investigate the impact of BMI on mortality in the HUNT study(31), but using offspring BMI as an instrument for the BMI of study participants. This analysis did not provide evidence for a harmful effect of having low BMI. However, offspring BMI is unlikely to satisfy the instrumental variable assumptions for the exposure of participant BMI. A similar analysis was performed in the 1958 British birth cohort, and a similar conclusion was reached(32). Blond *et al* also performed an instrumental variable analysis using offspring BMI as an instrument for the BMI of study participants in the Copenhagen School Health Records Register(33), showing evidence of positive effects of increases in BMI on mortality at both high and low BMI levels. Finally, Kjøllesdal et al performed an instrumental variable analysis using early adulthood BMI as an instrument for the midlife BMI of study participants in various Norwegian health surveys(34), showing mild J-shaped associations in the relationship between BMI and mortality, but far less pronounced than from observational analyses of the same dataset.

Overall, there is general consensus from different approaches that the adverse relationship of low BMI with mortality is exaggerated in observational analyses. However, variation between these results indicates the importance of modelling assumptions, both for the identification of causal effects, and for the modelling of non-linear relationships. Indeed, in our investigations, if we had used fractional polynomials of degree 1 rather than degree 2, then we would have observed monotonic increasing relationships of BMI with all outcomes. This is because the simpler degree 1 models are dominated by behaviour at the upper end of the BMI distribution, where instruments are strongest and most mortality outcomes occur. The simpler degree 1 polynomials smooth over imprecise negative estimates at the lower end of the BMI distribution, and so do not allow uncertainty in the shape of the distribution (particularly at its lower end) to be accurately reflected.

Our analysis has several strengths, but also limitations. The Mendelian randomization design minimizes bias from confounding and reverse causation. The large sample sizes of the HUNT and UK Biobank studies enable powerful analyses, even in strata of the population with low BMI levels. These sample sizes also enable a fine stratification of individuals, meaning that outcome associations can be assessed in strata only consisting of low BMI (<20 kg/m^2) individuals.

However, there are important limitations, which should lead to caution in the interpretation of our findings. As with all Mendelian randomization analyses, findings are dependent on the validity of the genetic variants as instrumental variables. While we were able to show null associations of the genetic score with some traits, it is never possible to prove the validity of the instrumental variable assumptions beyond all doubt. To reduce the scope for population stratification, our analyses were limited to European ancestry participants. Our findings may therefore not be applicable to other

For peer review only - http://bmjopen.bmj.com/site/about/guidelines.xhtml

populations. Further, recruitment into UK Biobank is dependent on age, sex, and other factors, which leads to bias in Mendelian randomization estimates. While the effect of moderate selection on Mendelian randomization estimates is often slight(35), the extent of selection bias may differ between strata, leading to differential bias across stratum-specific estimates(28). It is likely that age and sex are the strongest predictors of study participants, and differential genetic associations with these traits were observed in UK Biobank. For other traits, patterns in genetic associations across strata were less evident, and may reflect downstream effects of BMI rather than instrument invalidity due to pleiotropy or selection bias. As the HUNT study focused on a specific geographic area of Norway, it achieved much higher recruitment rates (around 70%, compared with 5% in UK Biobank), and so should be less affected by selection bias. This was corroborated by our analyses, as genetic associations in strata were weaker in the HUNT study. While genetic associations are protected from reverse causation, strata membership is determined by BMI levels, and hence could be subject to reverse causation. In particular, low BMI strata could contain a large proportion of individuals whose BMI levels are low because of comorbidity. However, genetic associations should still provide a reliable guide as to the extent and direction of any causal effect of BMI in these strata. Finally, all estimates represent averages across strata of the population; individual effects of raising or lowering BMI may vary between individuals. Differences in estimates for different strata may reflect the changing composition of the strata, as the characteristics of those with BMI less than 20 kg/m^2 are likely to be different to those with higher BMI. A non-linear curve may reflect a combination of different effects in different subgroups of the population, rather than that the relationship between BMI and mortality risk is non-linear for any individual in the population(36).

Conclusions

In conclusion, non-linear Mendelian randomization analyses using the doubly-ranked stratification method provide strong evidence for harmful effects of increased BMI on mortality above 25 kg/m^2. Evidence for a harmful effect of low BMI was only present in some analyses, and where present, only below 20 kg/m^2.

For peer review only - http://bmjopen.bmj.com/site/about/guidelines.xhtml

Table 1: Baseline characteristics of study participants in HUNT and UK Biobank studies

	HUNT	UK Biobank
Number of participants (n)	56,150	366,385
Men (%)	47.1	45.9
Age at baseline in years (SD)	49.6 (16.6)	56.7 (8.0)
Number of deaths (n)	18,836	25,021
- Cardiovascular mortality events (n)	-	5212
- Cancer mortality events (n)	-	12,880
- Other (non-cardiovascular non-cancer) events (n)	-	6692
BMI in kg/m^2 (SD)	26.3 (4.1)	27.4 (4.8)
Median follow-up years	26.5	12.0
Ever-smokers (%)	55.9	46.1

BMI: body mass index; SD: standard deviation; Data are given as number of subjects (n), percentage (%), median, or mean (standard deviation). For 237 deaths in UK Biobank, sufficient data were not available to classify the event as cardiovascular, cancer, or non-cardiovascular non-cancer.

For peer review only

For peer review only - http://bmjopen.bmj.com/site/about/guidelines.xhtml

FIGURE CAPTIONS

Figure 1: Genetic associations with potential competing risk factors in strata of the UK Biobank population

Figure 2: Non-linear Mendelian randomization – dose-response curve between body mass index and all-cause mortality. Using the doubly-ranked method: A) HUNT, B) UK Biobank; using the residual method (unreliable when the genetic effect on the exposure varies): C) HUNT, D) UK Biobank. Gradient at each point of the curve is the localized average causal effect. Grey lines represent 95% confidence intervals. The reference value for BMI was taken as 25 kg/m^2.

Figure 3: Non-linear Mendelian randomization – dose-response curve between body mass index and all-cause mortality in men and women respectively using doubly-ranked method. A) HUNT, men only; B) HUNT, women only; C) UK Biobank, men only; D) UK Biobank, women only. Gradient at each point of the curve is the localized average causal effect. Grey lines represent 95% confidence intervals. The reference value for BMI was taken as 25 kg/m^2.

Figure 4: Non-linear Mendelian randomization in UK Biobank – dose-response curve between body mass index and cause-specific mortality using doubly-ranked method. A) Cardiovascular disease mortality; B) Cancer mortality. C) Mortality due to other causes (non-cardiovascular, non-cancer). Gradient at each point of the curve is the localized average causal effect. Grey lines represent 95% confidence intervals. The reference value for BMI was taken as 25 kg/m^2.

For peer review only

Contributor statement:

SB, YQS, and XMM conceptualized the project. SB and YQS performed formal analysis. SB, YQS, AZ, CB, AMM, and XMM contributed to the methodology and interpretation of findings. YQS and AMM helped with data curation. SB and XMM supervised the project. SB wrote the initial manuscript draft. All authors (SB, YQS, AZ, CB, AMM, and XMM) contributed to the drafting and editing of the manuscript. All authors (SB, YQS, AZ, CB, AMM, and XMM) have approved of the final version of the manuscript.

Competing interest statement: None declared.

Funding statement: This work was supported by the Wellcome Trust (grant number 225790/Z/22/Z), the United Kingdom Research and Innovation Medical Research Council (grant number MC_UU_00002/7), the British Heart Foundation (RG/18/13/33946) and the National Institute for Health and Care Research Cambridge Biomedical Research Centre (BRC-1215-20014; NIHR203312). YQS was supported by a researcher grant from The Liaison Committee for Education, Research and Innovation in Central Norway (project ID 2018/42794). AMM is funded by the National Institute for Health and Care Research Blood and Transplant Research Unit in Donor Health and Behaviour (NIHR203337). The views expressed are those of the authors and not necessarily those of the NIHR, NHSBT or the Department of Health and Social Care.

Data sharing statement: Data from the HUNT and UK Biobank studies are available on application to any bona fide scientific researcher. Summary statistics for genetic associations with the exposure and outcome in strata for UK Biobank are available at http://doi.org/10.6084/m9.figshare.25041686.

Ethical approval

The HUNT study has ethical approval from The Regional Committee for Medical Research in Norway (REK 2015/78). Approval for individual projects is regulated in conjunction with The Norwegian Social Science Data Services (NSD). The UK Biobank study has ethical approval from the North West Multi-centre Research Ethics Committee (16/NW/0274). This research has been conducted using the UK Biobank Resource under Application Number 7439.

Acknowledgments

The Trøndelag Health Study (HUNT) is a collaboration between the HUNT Research Centre (Faculty of Medicine and Health Sciences, NTNU, Norwegian University of Science and Technology), Trøndelag County Council, Central Norway Regional Health Authority and the Norwegian Institute of Public Health.

For peer review only

REFERENCES

1. Aune D, Sen A, Prasad M, Norat T, Janszky I, Tonstad S, et al. BMI and all cause mortality: systematic review and non-linear dose-response meta-analysis of 230 cohort studies with 3.74 million deaths among 30.3 million participants. BMJ. 2016;353:i2156.
2. Bhaskaran K, dos-Santos-Silva I, Leon DA, Douglas IJ, Smeeth L. Association of BMI with overall and cause-specific mortality: a population-based cohort study of 3.6 million adults in the UK. The Lancet Diabetes & Endocrinology. 2018;6(12):944-53.
3. Global BMI Mortality Collaboration, Di Angelantonio E, Bhupathiraju Sh N, Wormser D, Gao P, Kaptoge S, et al. Body-mass index and all-cause mortality: individual-participant-data meta-analysis of 239 prospective studies in four continents. Lancet. 2016;388(10046):776-86.
4. Flegal KM, Kit BK, Orpana H, Graubard BI. Association of All-Cause Mortality With Overweight and Obesity Using Standard Body Mass Index Categories: A Systematic Review and Meta-analysis. JAMA. 2013;309(1):71-82.
5. Jenkins DA, Wade KH, Carslake D, Bowden J, Sattar N, Loos RJF, et al. Estimating the causal effect of BMI on mortality risk in people with heart disease, diabetes and cancer using Mendelian randomization. International journal of cardiology. 2021;330:214-20.
6. Visaria A, Setoguchi S. Body mass index and all-cause mortality in a 21st century U.S. population: A National Health Interview Survey analysis. PLOS ONE. 2023;18(7):e0287218.
7. Davey Smith G, Ebrahim S. 'Mendelian randomization': can genetic epidemiology contribute to understanding environmental determinants of disease? International Journal of Epidemiology. 2003;32(1):1-22.
8. Lawlor DA, Harbord RM, Sterne JA, Timpson N, Davey Smith G. Mendelian randomization: using genes as instruments for making causal inferences in epidemiology. Statistics in Medicine. 2008;27(8):1133-63.
9. Thanassoulis G, O'Donnell CJ. Mendelian randomization: nature's randomized trial in the post-genome era. JAMA. 2009;301(22):2386-8.
10. Burgess S, Swanson SA, Labrecque JA. Are Mendelian randomization investigations immune from bias due to reverse causation? European Journal of Epidemiology. 2021;36(3):253-7.
11. Martens EP, Pestman WR, de Boer A, Belitser SV, Klungel OH. Instrumental variables: application and limitations. Epidemiology. 2006:260-7.
12. Staley JR, Burgess S. Semiparametric methods for estimation of a nonlinear exposure-outcome relationship using instrumental variables with application to Mendelian randomization. Genet Epidemiol. 2017;41(4):341-52.
13. Burgess S, Davies NM, Thompson SG. Instrumental variable analysis with a nonlinear exposure-outcome relationship. Epidemiology. 2014;25(6):877-85.
14. Cole SR, Platt RW, Schisterman EF, Chu H, Westreich D, Richardson D, Poole C. Illustrating bias due to conditioning on a collider. International Journal of Epidemiology. 2010;39(2):417-20.
15. Small DS. Commentary: Interpretation and Sensitivity Analysis for the Localized Average Causal Effect Curve. Epidemiology. 2014;25(6).
16. Davey Smith G. Mendelian randomisation and vitamin D: the importance of model assumptions. The Lancet Diabetes & Endocrinology. 2023;11(1):14.
17. Burgess S. Violation of the constant genetic effect assumption can result in biased estimates for non-linear Mendelian randomization. Human Heredity. 2022(In Press).
18. Tian H, Mason AM, Liu C, Burgess S. Relaxing parametric assumptions for non-linear Mendelian randomization using a doubly-ranked stratification method. PLOS Genetics. 2023;19(6):e1010823.
19. Sun Y-Q, Burgess S, Staley JR, Wood AM, Bell S, Kaptoge SK, et al. Body mass index and all cause mortality in HUNT and UK Biobank studies: linear and non-linear mendelian randomisation analyses. British Medical Journal. 2019;364:l1042.
20. Krokstad S, Langhammer A, Hveem K, Holmen TL, Midthjell K, Stene TR, et al. Cohort Profile: The HUNT Study, Norway. International Journal of Epidemiology. 2013;42(4):968-77.

For peer review only - http://bmjopen.bmj.com/site/about/guidelines.xhtml

21. Sudlow C, Gallacher J, Allen N, Beral V, Burton P, Danesh J, et al. UK Biobank: an open access resource for identifying the causes of a wide range of complex diseases of middle and old age. PLOS Medicine. 2015;12(3):e1001779.
22. Astle WJ, Elding H, Jiang T, Allen D, Ruklisa D, Mann AL, et al. The allelic landscape of human blood cell trait variation and links to common complex disease. Cell. 2016;167(5):1415-29.
23. Locke AE, Kahali B, Berndt SI, Justice AE, Pers TH, Day FR, et al. Genetic studies of body mass index yield new insights for obesity biology. Nature. 2015;518(7538):197-206.
24. Yengo L, Sidorenko J, Kemper KE, Zheng Z, Wood AR, Weedon MN, et al. Meta-analysis of genome-wide association studies for height and body mass index in ~700000 individuals of European ancestry. Human Molecular Genetics. 2018;27(20):3641-9.
25. Verkouter I, de Mutsert R, Smit RAJ, Trompet S, Rosendaal FR, van Heemst D, et al. The contribution of tissue-grouped BMI-associated gene sets to cardiometabolic-disease risk: a Mendelian randomization study. International Journal of Epidemiology. 2020;49(4):1246-56.
26. Carreras-Torres R, Johansson M, Haycock PC, Relton CL, Davey Smith G, Brennan P, Martin RM. Role of obesity in smoking behaviour: Mendelian randomisation study in UK Biobank. BMJ. 2018;361:k1767.
27. Mason AM, Burgess S. Software Application Profile: SUMnlmr, an R package that facilitates flexible and reproducible non-linear Mendelian randomization analyses. International Journal of Epidemiology. 2022;51(6):2014-9.
28. Hamilton FW, Hughes DA, Spiller W, Tilling K, Davey Smith G. Non-linear mendelian randomization: evaluation of biases using negative controls with a focus on BMI and Vitamin D. medRxiv. 2023:2023.08.21.23293658.
29. Wade KH, Carslake D, Sattar N, Davey Smith G, Timpson NJ. BMI and Mortality in UK Biobank: Revised Estimates Using Mendelian Randomization. Obesity. 2018;26(11):1796-806.
30. Sulc J, Sjaarda J, Kutalik Z. Polynomial Mendelian randomization reveals non-linear causal effects for obesity-related traits. HGG Advances. 2022;3(3):100124.
31. Carslake D, Davey Smith G, Gunnell D, Davies N, Nilsen TIL, Romundstad P. Confounding by ill health in the observed association between BMI and mortality: evidence from the HUNT Study using offspring BMI as an instrument. International Journal of Epidemiology. 2018;47(3):760-70.
32. Hyppönen E, Carslake D, Berry DJ, Power C, Davey Smith G. Estimating the influence of body mass index (BMI) on mortality using offspring BMI as an instrumental variable. International Journal of Obesity. 2022;46(1):77-84.
33. Blond K, Carslake D, Gjærde LK, Vistisen D, Sørensen TIA, Smith GD, Baker JL. Instrumental variable analysis using offspring BMI in childhood as an indicator of parental BMI in relation to mortality. Scientific Reports. 2021;11(1):22408.
34. Kjøllesdal MKR, Davey Smith G, Ariansen I, Kinge JM, Degerud E, Næss Ø. The association between BMI and mortality using early adulthood BMI as an instrumental variable for midlife BMI. Scientific Reports. 2018;8(1):11499.
35. Gkatzionis A, Burgess S. Contextualizing selection bias in Mendelian randomization: how bad is it likely to be? International Journal of Epidemiology. 2018;48(3):691-701.
36. Small DS. Commentary: Interpretation and sensitivity analysis for the localized average causal effect curve. Epidemiology. 2014;25(6):886-8.

For peer review only - http://bmjopen.bmj.com/site/about/guidelines.xhtml

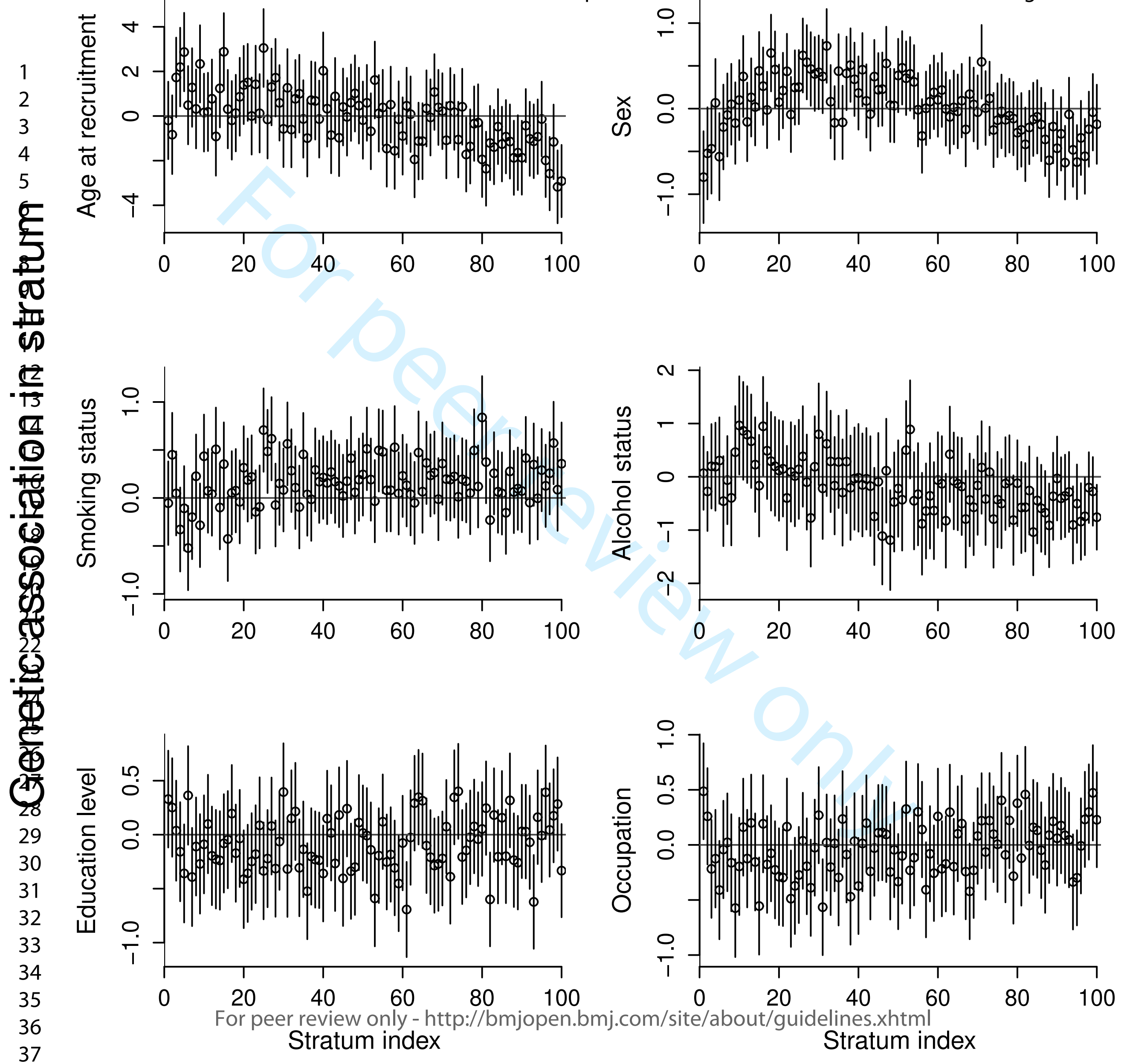
Genetic association in stratum
Age at recruitment
Sex
Smoking status
Alcohol status
Education level
Occupation
Stratum index
Stratum index

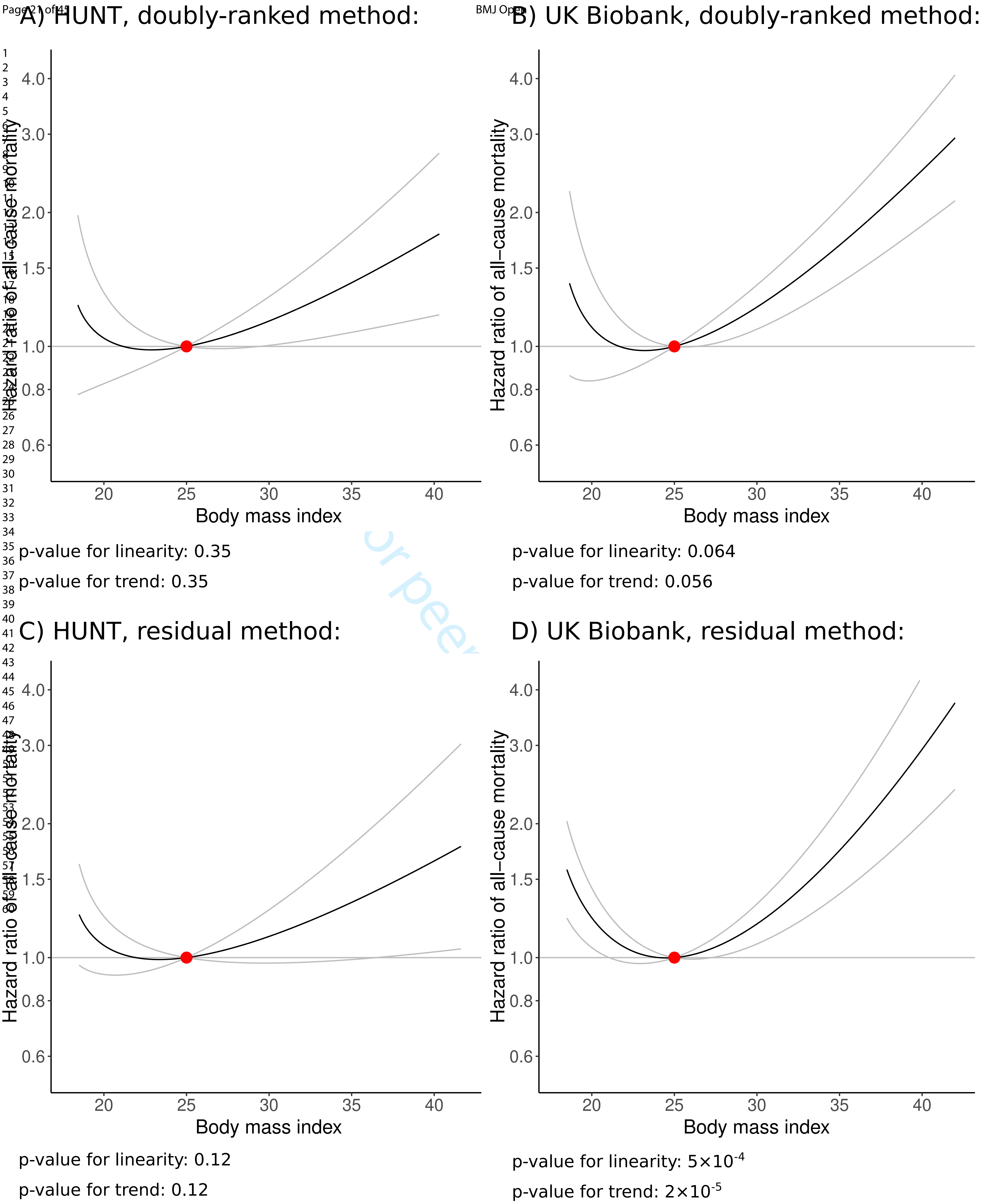
A) HUNT, doubly-ranked method:
Hazard ratio of all−cause mortality
Body mass index
p-value for linearity: 0.35
p-value for trend: 0.35
B) UK Biobank, doubly-ranked method:
Hazard ratio of all−cause mortality
Body mass index
p-value for linearity: 0.064
p-value for trend: 0.056
C) HUNT, residual method:
Hazard ratio of all−cause mortality
Body mass index
p-value for linearity: 0.12
p-value for trend: 0.12
D) UK Biobank, residual method:
Hazard ratio of all−cause mortality
Body mass index
p-value for linearity: 5×10-4
p-value for trend: 2×10-5

A) HUNT, men only:

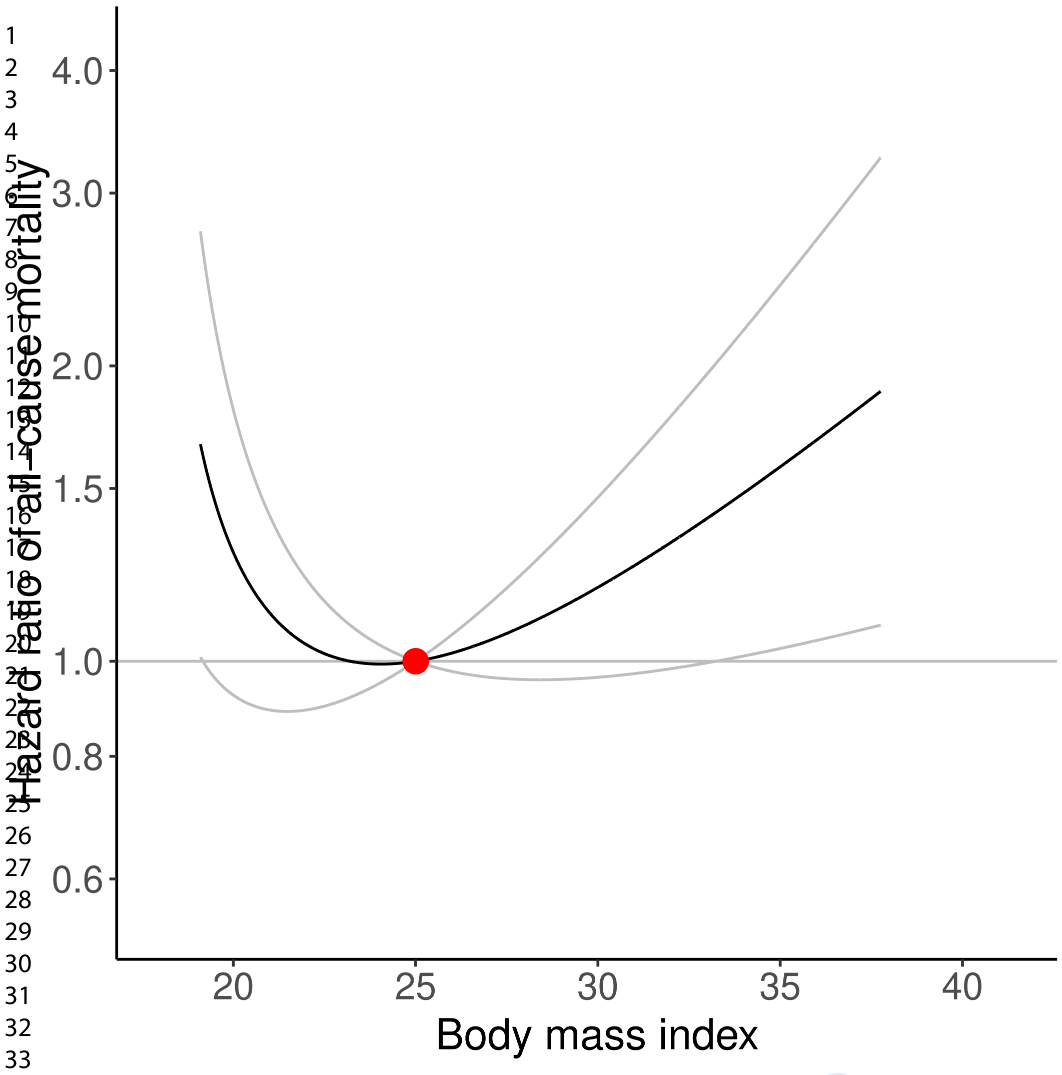

p-value for linearity: 0.050

p-value for trend: 0.22

B) HUNT, women only:

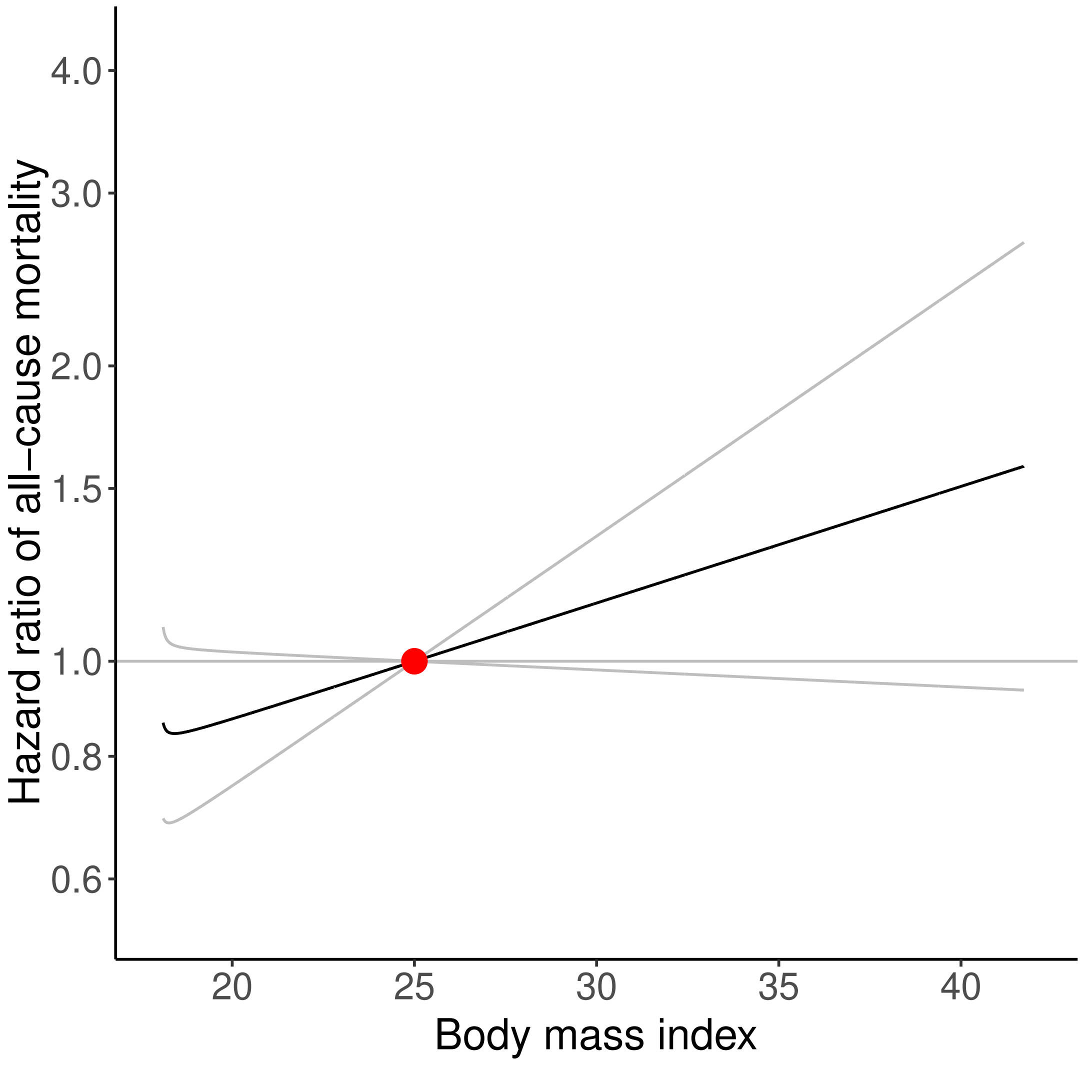

p-value for linearity: 0.99

p-value for trend: 0.79

C) UK Biobank, men only:

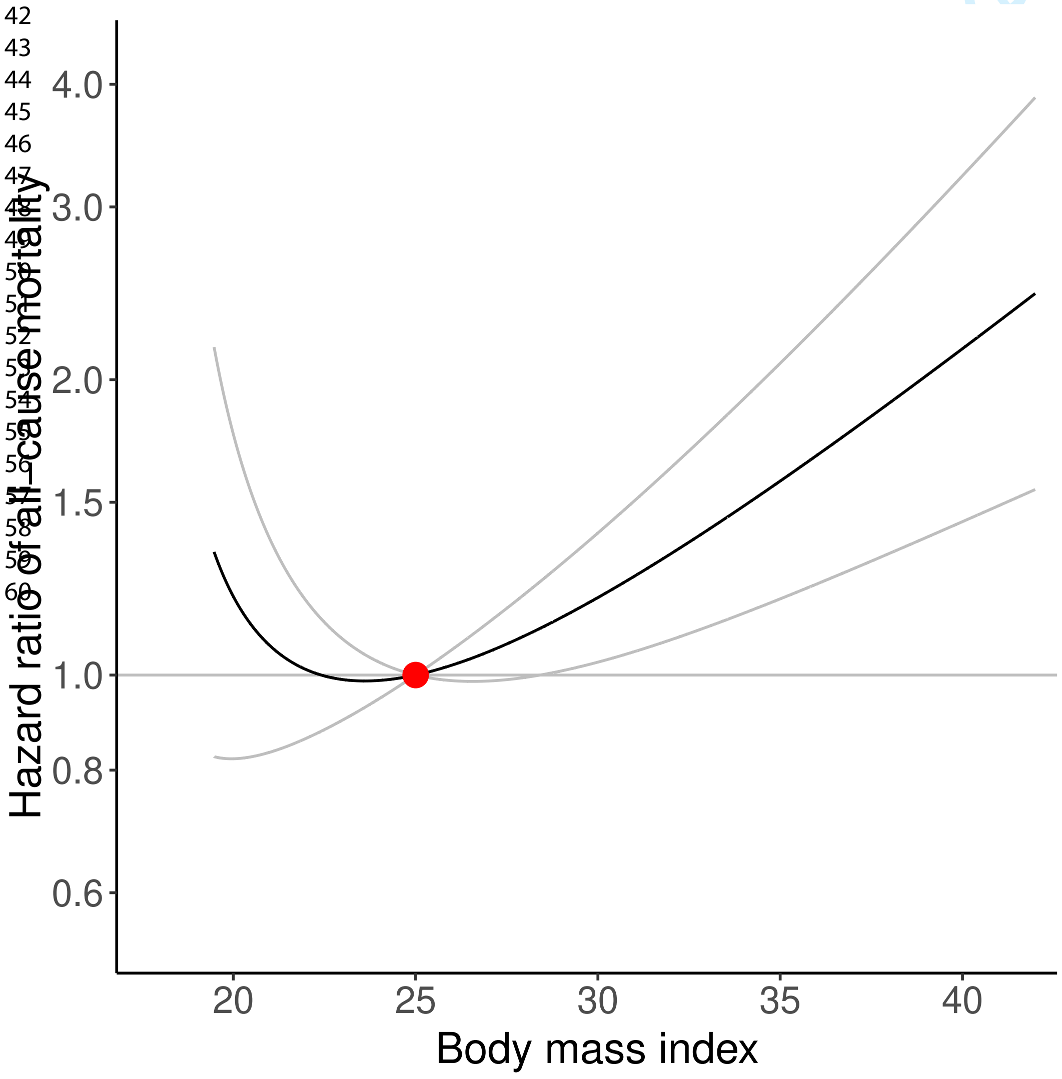

p-value for linearity: 0.18

p-value for trend: 0.18

D) UK Biobank, women only:

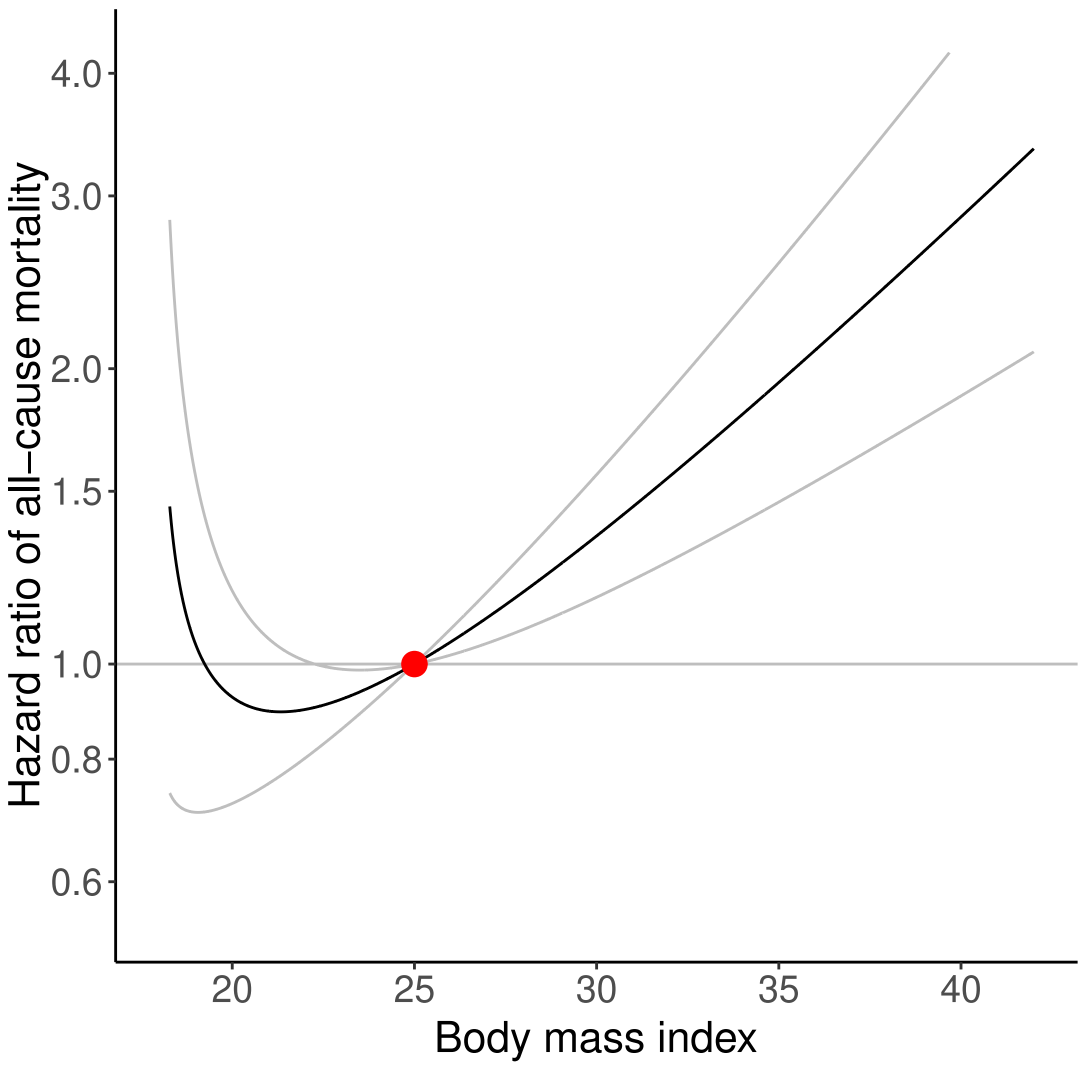

p-value for linearity: 0.21

p-value for trend: 0.18

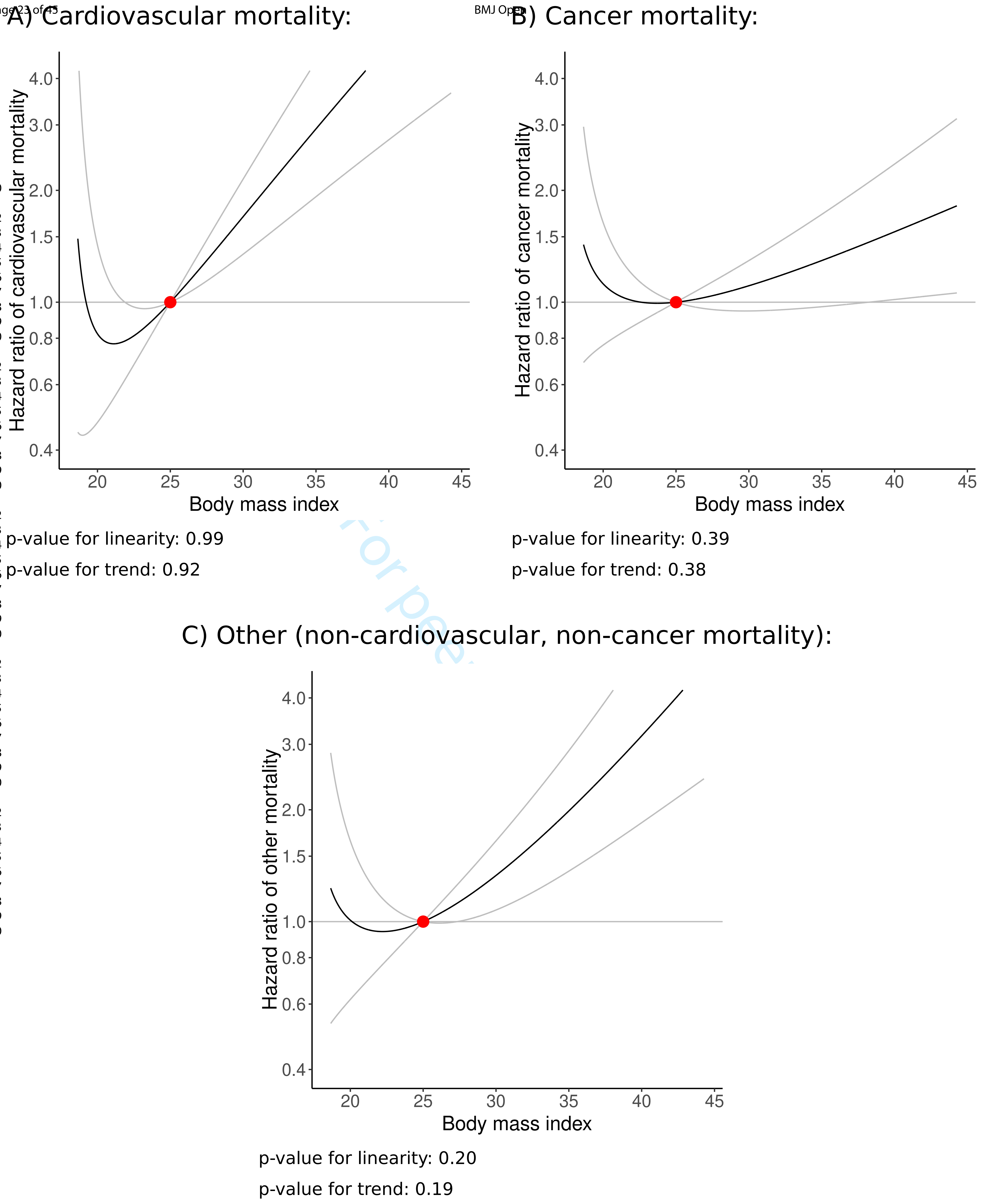
A) Cardiovascular mortality:
Hazard ratio of cardiovascular mortality
4.0
3.0
2.0
1.5
1.0
0.8
0.6
0.4
20
25
30
35
40
45
Body mass index
p-value for linearity: 0.99
p-value for trend: 0.92
B) Cancer mortality:
Hazard ratio of cancer mortality
4.0
3.0
2.0
1.5
1.0
0.8
0.6
0.4
20
25
30
35
40
45
Body mass index
p-value for linearity: 0.39
p-value for trend: 0.38
C) Other (non-cardiovascular, non-cancer mortality):
Hazard ratio of other mortality
4.0
3.0
2.0
1.5
1.0
0.8
0.6
0.4
20
25
30
35
40
45
Body mass index
p-value for linearity: 0.20
p-value for trend: 0.19

SUPPLEMENTARY MATERIAL

We here perform a simulation study investigating whether adjusting genetic associations for factors that influence selection reduces selection bias within strata in a non-linear Mendelian randomization framework. We generate simulated data for 1,000,000 individuals on a genetic variant *G*, an exposure *X*, an outcome *Y*, and competing risk factors *U* and *V*. For simplicity, all variables are modelled as continuous and normally distributed.

We consider two scenarios. In each scenario, the genetic variant is a valid instrumental variable in the population as a whole, and there is no true effect of the exposure on the outcome. This implies that the genetic variant should not be associated with the outcome. We estimate the association between the genetic variant and the outcome in a selected subset of the population.

In scenario 1, the data-generating model is:

$$G_i \sim Normal(0,1)$$
$$U_i \sim Normal(0,1)$$
$$V_i \sim Normal(0,1)$$
$$X_i \sim Normal(0.2\ G_i + 0.4\ U_i + 0.4V_i, 1)$$
$$Y_i \sim Normal(-0.5\ U_i + 0.2\ V_i, 1)$$

Participants (indexed by the *i* subscript) are selected into the sample with probability $expit(-2 + U_i + X_i)$, where $expit(x) = \frac{\exp{(x)}}{1+\exp{(x)}}$ is the inverse of the logistic (logit) function. Here *U* and *V* are both confounders of the relationship between the exposure and outcome, and selection depends on both *U* and *X*. We compare genetic associations with the outcome with and without adjustment for *U*.

In scenario 2, the data-generating model is:

$$G_i \sim Normal(0,1)$$
$$V_i \sim Normal(0,1)$$
$$X_i \sim Normal(0.2\ G_i + 0.8\ V_i, 1)$$
$$U_i \sim Normal(X_i, 1)$$
$$Y_i \sim Normal(0.5\ V_i, 1)$$

Participants are selected into the sample with probability $expit(-2 + U_i)$. Here *V* is a confounder (common cause) of the relationship between the exposure and outcome, and *U* is a downstream effect of the exposure *X*, and hence *U* is a collider (common effect) of *G* and *V*. Selection depends on *U* only. Again, we compare genetic associations with the outcome with and without adjustment for *U*.

In both cases, all normal distributions are independent. We simulate 1000 datasets for each scenario, and compare the mean estimate of the genetic association with the outcome and its empirical Type 1 error rate at a 5% significance level (that is, the proportion of 95% confidence intervals excluding the null). Around 15-20% of the 1,000,000 participants are selected in each simulated dataset.

In scenario 1, the mean association of the genetic variant with the outcome without adjustment for *U* was 0.014 (mean standard error 0.002), and the empirical Type 1 error rate was 100.0%. With adjustment for *U*, the mean association was -0.002 (mean standard error 0.002), and the empirical Type 1 error rate was 17.1%.

In scenario 2, the mean association of the genetic variant with the outcome without adjustment for *U* was -0.010 (mean standard error 0.002), and the empirical Type 1 error rate was 97.9%. With

For peer review only - http://bmjopen.bmj.com/site/about/guidelines.xhtml

adjustment for *U*, the mean association was -0.030 (mean standard error 0.002), and the empirical Type 1 error rate was 100.0%.

In scenario 1, bias reduced considerably on adjustment for the factor influencing selection. Mean bias did not reduce to zero, as selection also depends on the exposure. We note that if selection did not additionally depend on the exposure, there would not be selection bias, as *U* is a confounder not a collider. However, even in this case with imperfect adjustment, the reduction in bias was substantial. In scenario 2, there was bias without adjustment for the collider *U*, but mean bias sharpened (got worse) on adjustment for *U*. Scenario 1 is more likely to be representative of the situation we see in the paper, as sex and age cannot be consequences of the exposure, but are likely to be confounders.

This simulation study is deliberately designed to be relatively simple and limited in scope, as we do not believe that any simulation study would be able to prove conclusively that covariate adjustment eliminates selection bias in all cases where selection depends on a confounder. Indeed, we do not make that claim; rather we claim that adjustment for confounders that are key predictors of selection can ameliorate bias due to selection. There will always be intrinsic uncertainty in practice, as we do not know all the factors that influence selection, and we would be cautious to adjust for too many variables in a Mendelian randomization analysis, particularly for variables that could be mediators on the causal pathway from the exposure to the outcome, or could be colliders. As age and sex cannot logically be caused by the exposure, they can be safely adjusted for without risk of these variables being mediators or colliders [S1]. Hence, if age and sex are strong predictors of selection into the sample, then adjustment for age and sex should reduce selection bias in Mendelian randomization investigations.

We believe that any residual bias from selection is likely to be low, in line with previous simulation studies that have shown selection effects need to be substantial in magnitude to have non-negligible biasing effects on Mendelian randomization estimates [S2]. However, we acknowledge that this imposes an additional level of uncertainty on the results of a non-linear Mendelian randomization analysis, on top of the existing uncertainty in the Mendelian randomization paradigm due to the validity of the instrumental variable assumptions, and other factors. All methods for making causal inferences from observational data rely on untestable assumptions, and findings should be interpreted with appropriate caution.

SUPPLEMENTARY REFERENCES

S1. Burgess S, Davey Smith G, Davies NM, Dudbridge F, Gill D, Glymour MM, Hartwig FP, Kutalik Z, Holmes MV, Minelli C, Morrison JV, *et al*. Guidelines for performing Mendelian randomization investigations: update for summer 2023. Wellcome Open Research 2023; 4:186.

S2. Gkatzionis A, Burgess S. Contextualizing selection bias in Mendelian randomization: how bad is it likely to be? International Journal of Epidemiology. 2019; 48(3):691-701.

For peer review only - http://bmjopen.bmj.com/site/about/guidelines.xhtml

Supplementary Table 1: Genetic variants and associations with BMI based on European sex-combined analysis from GIANT [23]. Beta-coefficents are in standard deviation units.

SNP	Effect allele*	Other allele	Beta-coefficient	Standard error of beta	Number of subjects	*P*-value
rs1558902	A	T	0.082	0.003	320 073	7.51E-153
rs6567160	C	T	0.056	0.004	321 958	3.93E-53
rs10938397	G	A	0.040	0.003	320 955	3.21E-38
rs543874	G	A	0.048	0.004	322 008	2.62E-35
rs2207139	G	A	0.045	0.004	322 019	4.13E-29
rs11030104	A	G	0.041	0.004	322 103	5.56E-28
rs3101336	C	T	0.033	0.003	316 872	2.66E-26
rs7138803	A	G	0.032	0.003	322 092	8.15E-24
rs10182181	G	A	0.031	0.003	321 759	8.78E-24
rs3888190	A	C	0.031	0.003	321 930	3.14E-23
rs1516725	C	T	0.045	0.005	320 644	1.89E-22
rs12446632	G	A	0.040	0.005	316 758	1.48E-18
rs2287019	C	T	0.036	0.004	300 921	4.59E-18
rs3817334	T	C	0.026	0.003	321 959	5.15E-17
rs2112347	T	G	0.026	0.003	322 019	6.19E-17
rs12566985	G	A	0.024	0.003	319 282	3.28E-15
rs3810291	A	G	0.028	0.004	296 261	4.81E-15
rs7141420	T	C	0.024	0.003	321 970	1.23E-14
rs13078960	G	T	0.030	0.004	322 135	1.74E-14
rs10968576	G	A	0.025	0.003	322 061	6.61E-14
rs17024393	C	T	0.066	0.009	297 874	7.03E-14
rs657452	A	G	0.023	0.003	313 651	5.48E-13
rs12429545	A	G	0.033	0.005	312 934	1.09E-12
rs12286929	G	A	0.022	0.003	321 903	1.31E-12
rs13107325	T	C	0.048	0.007	321 461	1.83E-12
rs11165643	T	C	0.022	0.003	320 730	2.07E-12
rs7903146	C	T	0.023	0.003	322 130	1.11E-11
rs10132280	C	A	0.023	0.003	321 797	1.14E-11
rs17405819	T	C	0.022	0.003	322 085	2.07E-11
rs1016287	T	C	0.023	0.003	321 969	2.25E-11
rs4256980	G	C	0.021	0.003	320 028	2.90E-11
rs17094222	C	T	0.025	0.004	321 770	5.94E-11
rs12401738	A	G	0.021	0.003	322 070	1.15E-10
rs7599312	G	A	0.022	0.003	322 024	1.17E-10
rs2365389	C	T	0.020	0.003	316 768	1.63E-10
rs205262	G	A	0.022	0.004	315 542	1.75E-10
rs2820292	C	A	0.020	0.003	321 707	1.83E-10
rs12885454	C	A	0.021	0.003	320 823	1.94E-10
rs16851483	T	G	0.048	0.008	233 929	3.55E-10
rs1167827	G	A	0.020	0.003	306 238	6.33E-10
rs758747	T	C	0.023	0.004	308 688	7.47E-10
rs1928295	T	C	0.019	0.003	321 979	7.91E-10
rs9925964	A	G	0.019	0.003	318 385	8.11E-10

For peer review only - http://bmjopen.bmj.com/site/about/guidelines.xhtml

rs11126666	A	G	0.021	0.003	321 979	1.33E-09
rs2650492	A	G	0.021	0.004	319 464	1.92E-09
rs6804842	G	A	0.019	0.003	321 463	2.48E-09
rs12940622	G	A	0.018	0.003	322 032	2.49E-09
rs11847697	T	C	0.049	0.008	306 243	3.99E-09
rs4740619	T	C	0.018	0.003	321 887	4.56E-09
rs13191362	A	G	0.028	0.005	321 902	7.34E-09
rs3736485	A	G	0.018	0.003	321 398	7.41E-09
rs17001654	G	C	0.031	0.005	233 722	7.76E-09
rs11191560	C	T	0.031	0.005	321 893	8.45E-09
rs1528435	T	C	0.018	0.003	321 924	1.20E-08
rs2075650	A	G	0.026	0.005	308 408	1.25E-08
rs1000940	G	A	0.019	0.003	321 836	1.28E-08
rs2033529	G	A	0.019	0.003	321 917	1.39E-08
rs11583200	C	T	0.018	0.003	322 095	1.48E-08
rs9400239	C	T	0.019	0.003	321 988	1.61E-08
rs10733682	A	G	0.017	0.003	320 727	1.83E-08
rs11688816	G	A	0.017	0.003	322 051	1.89E-08
rs11057405	G	A	0.031	0.006	314 111	2.02E-08
rs2121279	T	C	0.025	0.004	322 065	2.31E-08
rs29941	G	A	0.018	0.003	321 970	2.41E-08
rs11727676	T	C	0.036	0.006	296 401	2.55E-08
rs3849570	A	C	0.019	0.003	284 339	2.60E-08
rs6477694	C	T	0.017	0.003	322 048	2.67E-08
rs7899106	G	A	0.040	0.007	321 770	2.96E-08
rs2176598	T	C	0.020	0.004	316 848	2.97E-08
rs2245368	C	T	0.032	0.006	205 675	3.19E-08
rs17724992	A	G	0.019	0.004	319 588	3.42E-08
rs7243357	T	G	0.022	0.004	322 107	3.86E-08
rs1808579	C	T	0.017	0.003	322 032	4.17E-08

*BMI-increasing allele

For peer review only

For peer review only - http://bmjopen.bmj.com/site/about/guidelines.xhtml

Supplementary Table 2: ICD-10 codes for cause-specific mortality outcomes

Cause-specific mortality	ICD-10 codes
Cardiovascular	G45, I01, I03-I82, I87, I95-I99, F01, Q20-Q28, R96
Cancer	C00-C97, D00-D48
Non-cardiovascular non-cancer	All others

For peer review only

Supplementary Table 3: Distribution of BMI (kg/m^2) in strata defined by the doubly-ranked stratification method

	HUNT			UK Biobank		
Stratum number	Mean	Lower decile	Upper decile	Mean	Lower decile	Upper decile
1	18.4	17.5	19.8	18.6	17.8	20.0
2	19.3	18.2	20.4	19.6	18.5	20.7
3	19.9	18.8	20.8	20.1	19.1	21.1
4	20.3	19.4	21.1	20.5	19.6	21.5
5	20.6	19.7	21.5	20.9	20.0	21.8
6	20.9	20.1	21.7	21.1	20.3	22.0
7	21.1	20.3	21.9	21.4	20.6	22.3
8	21.3	20.5	22.1	21.6	20.8	22.5
9	21.5	20.7	22.3	21.8	21.0	22.7
10	21.7	20.9	22.5	22.0	21.2	22.8
11	21.8	21.1	22.7	22.2	21.4	23.0
12	22.0	21.2	22.8	22.4	21.6	23.2
13	22.1	21.4	22.9	22.5	21.7	23.3
14	22.3	21.5	23.1	22.7	21.9	23.5
15	22.4	21.7	23.2	22.8	22.0	23.7
16	22.5	21.8	23.3	23.0	22.2	23.8
17	22.7	21.9	23.5	23.1	22.3	23.9
18	22.8	22.0	23.6	23.2	22.4	24.1
19	22.9	22.1	23.7	23.4	22.6	24.2
20	23.0	22.2	23.8	23.5	22.7	24.3
21	23.1	22.4	23.9	23.6	22.8	24.4
22	23.2	22.5	24.0	23.7	22.9	24.5
23	23.3	22.6	24.1	23.8	23.0	24.7
24	23.4	22.7	24.2	24.0	23.2	24.8
25	23.6	22.8	24.3	24.1	23.3	24.9
26	23.6	22.9	24.5	24.2	23.4	25.0
27	23.8	23.0	24.5	24.3	23.5	25.2
28	23.8	23.1	24.7	24.4	23.6	25.3
29	23.9	23.1	24.8	24.5	23.7	25.4
30	24.0	23.2	24.8	24.6	23.8	25.5
31	24.1	23.3	25.0	24.7	23.9	25.6
32	24.2	23.4	25.0	24.8	24.0	25.7
33	24.3	23.5	25.1	24.9	24.1	25.8
34	24.4	23.6	25.2	25.0	24.2	25.9
35	24.5	23.7	25.3	25.1	24.3	26.0
36	24.6	23.8	25.4	25.2	24.4	26.1
37	24.7	23.8	25.5	25.3	24.5	26.2
38	24.8	23.9	25.6	25.4	24.6	26.3
39	24.8	24.0	25.7	25.5	24.7	26.4
40	24.9	24.1	25.8	25.6	24.8	26.6
41	25.0	24.2	25.9	25.7	24.9	26.7
42	25.1	24.3	26.0	25.8	25.0	26.8
43	25.2	24.3	26.1	25.9	25.1	26.9
44	25.3	24.4	26.2	26.0	25.1	27.0
45	25.4	24.5	26.3	26.1	25.3	27.1

For peer review only - http://bmjopen.bmj.com/site/about/guidelines.xhtml

For peer review only

46	25.5	24.6	26.4	26.2	25.4	27.2
47	25.6	24.7	26.4	26.3	25.4	27.3
48	25.6	24.8	26.6	26.5	25.5	27.4
49	25.7	24.8	26.7	26.6	25.6	27.5
50	25.8	25.0	26.8	26.7	25.7	27.6
51	25.9	25.0	26.9	26.8	25.8	27.7
52	26.0	25.1	26.9	26.9	25.9	27.8
53	26.1	25.2	27.0	27.0	26.0	28.0
54	26.2	25.3	27.1	27.1	26.1	28.1
55	26.3	25.4	27.2	27.2	26.2	28.2
56	26.4	25.5	27.3	27.3	26.3	28.3
57	26.5	25.5	27.4	27.4	26.4	28.4
58	26.6	25.6	27.5	27.5	26.5	28.6
59	26.7	25.7	27.6	27.6	26.6	28.7
60	26.8	25.8	27.7	27.7	26.7	28.8
61	26.9	25.9	27.9	27.9	26.8	28.9
62	27.0	26.0	27.9	28.0	26.9	29.1
63	27.1	26.1	28.1	28.1	27.0	29.2
64	27.2	26.2	28.2	28.2	27.1	29.3
65	27.3	26.3	28.3	28.3	27.2	29.5
66	27.4	26.4	28.4	28.5	27.3	29.6
67	27.5	26.5	28.6	28.6	27.5	29.8
68	27.6	26.6	28.7	28.7	27.6	29.9
69	27.7	26.7	28.8	28.9	27.7	30.0
70	27.9	26.8	28.9	29.0	27.8	30.2
71	28.0	26.9	29.0	29.1	28.0	30.4
72	28.1	27.0	29.1	29.3	28.1	30.5
73	28.2	27.1	29.4	29.4	28.2	30.7
74	28.3	27.2	29.5	29.6	28.4	30.9
75	28.5	27.4	29.6	29.8	28.5	31.1
76	28.6	27.5	29.8	29.9	28.7	31.2
77	28.7	27.6	29.9	30.1	28.8	31.4
78	28.9	27.8	30.1	30.3	28.9	31.6
79	29.0	27.9	30.3	30.4	29.1	31.8
80	29.2	28.0	30.5	30.6	29.3	32.1
81	29.4	28.2	30.7	30.8	29.4	32.3
82	29.5	28.3	30.9	31.0	29.6	32.6
83	29.7	28.4	31.1	31.3	29.8	32.8
84	29.9	28.6	31.3	31.5	30.0	33.0
85	30.1	28.8	31.5	31.7	30.2	33.3
86	30.3	28.9	31.8	32.0	30.4	33.7
87	30.6	29.1	32.0	32.3	30.6	34.0
88	30.8	29.3	32.3	32.5	30.9	34.4
89	31.0	29.5	32.6	32.9	31.1	34.7
90	31.3	29.7	33.0	33.2	31.4	35.2
91	31.6	30.0	33.3	33.6	31.7	35.6
92	32.0	30.3	33.7	34.0	32.0	36.2
93	32.3	30.6	34.2	34.5	32.4	36.7
94	32.8	30.9	34.8	35.0	32.8	37.4
95	33.3	31.4	35.5	35.7	33.3	38.2
96	33.9	31.8	36.1	36.4	33.9	39.2

For peer review only

97	34.7	32.4	37.1	37.4	34.6	40.5
98	35.8	33.3	38.7	38.7	35.5	42.2
99	37.3	34.1	40.5	40.5	36.7	44.8
100	40.3	35.6	43.2	44.3	38.8	47.9

We report the mean, lower decile (10th percentile of the distribution), and upper decile (90th percentile of the distribution) of the BMI distribution for participants in each stratum created by the doubly-ranked method. In the first and 100th strata, we instead report the 20th and 80th percentiles of the distribution.

For peer review only

For peer review only - http://bmjopen.bmj.com/site/about/guidelines.xhtml

Supplementary Figure 1: Associations between the genetic score and BMI in strata defined by the doubly-ranked method plotted against mean BMI in each stratum for: A) HUNT, B) UK Biobank. Error bars represent 95% confidence intervals.

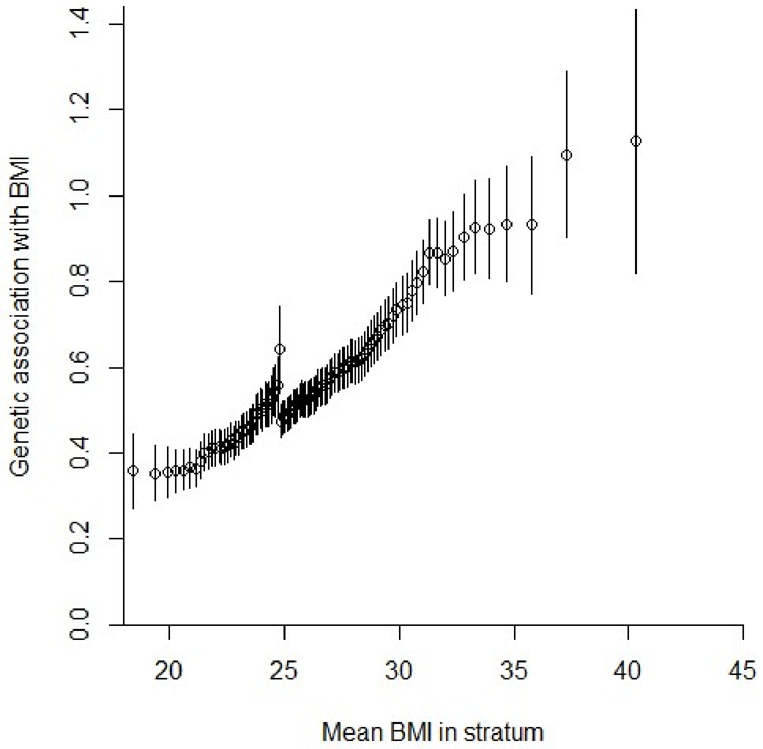

A.

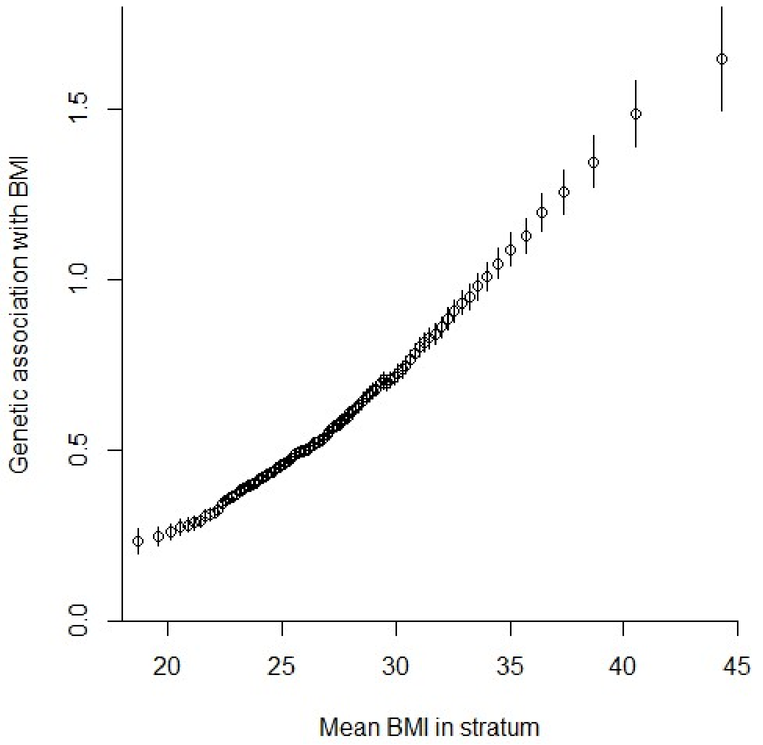

B.

For peer review only - http://bmjopen.bmj.com/site/about/guidelines.xhtml

Supplementary Figure 2: Genetic associations with potential competing risk factors in strata of the HUNT study population

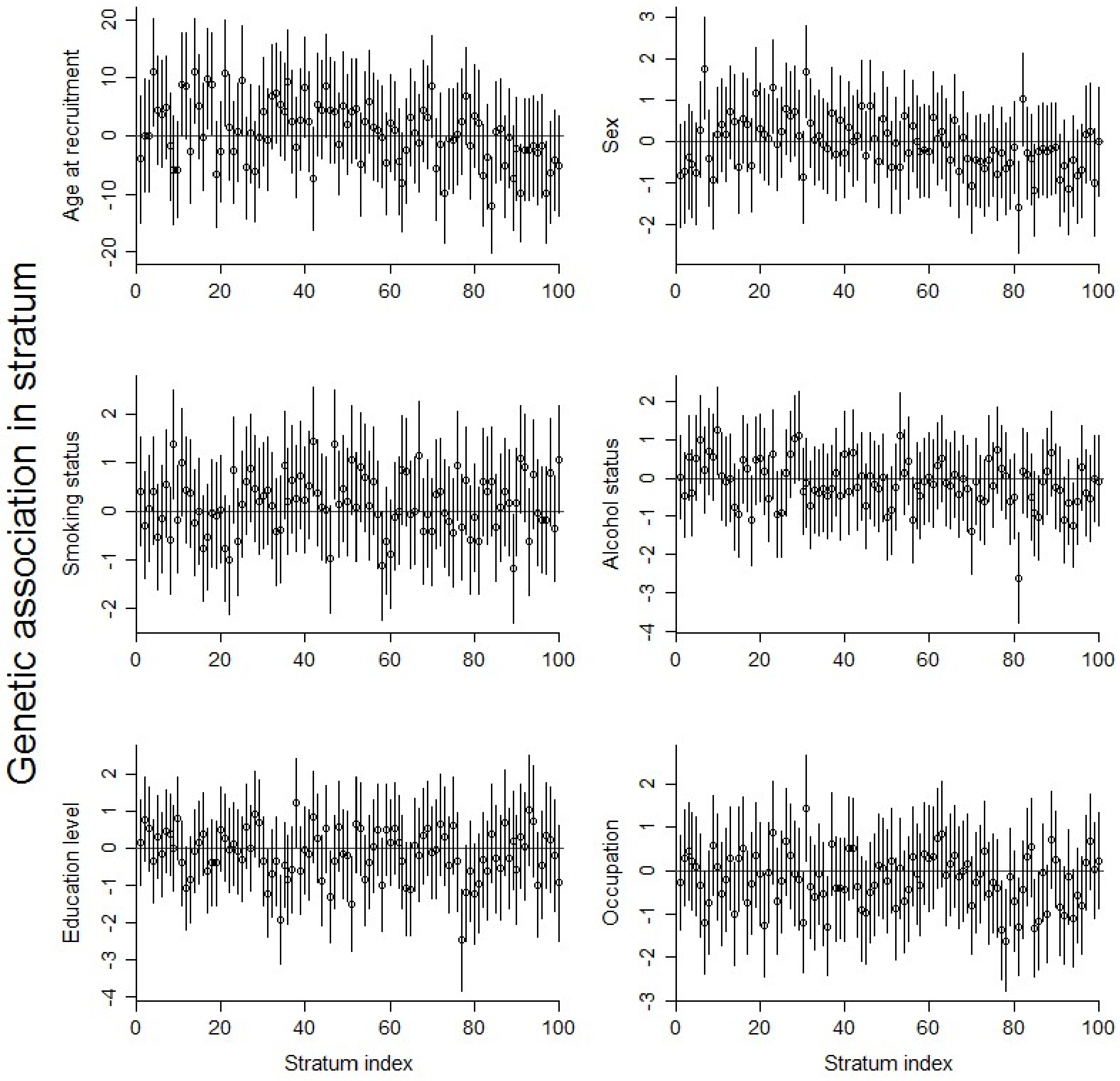

Points represent association estimates from linear (age at recruitment, years) or logistic regression (all other traits). Error bars represent 95% confidence intervals. Stratum 1 has lowest average BMI levels, stratum 100 has highest average BMI levels.

For peer review only - http://bmjopen.bmj.com/site/about/guidelines.xhtml

Supplementary Figure 3: Localized average causal effect estimates (stratum-specific estimates) for all-cause mortality plotted against mean BMI in each stratum. Estimates represent hazard ratios per 1 kg/m^2 increase in genetically-predicted BMI. Error bars represent 95% confidence intervals.

A) HUNT, doubly-ranked method:

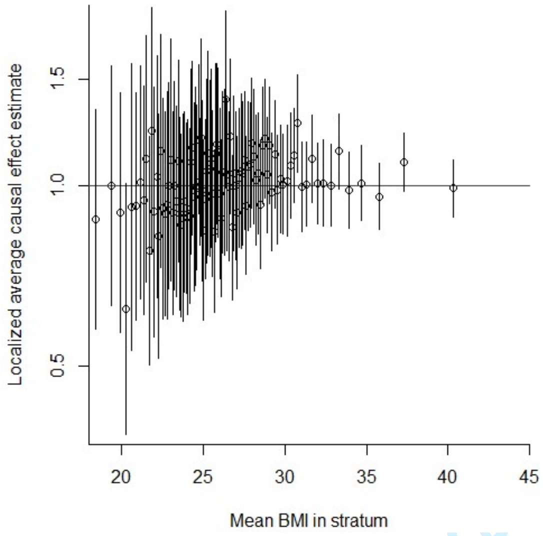

B) UK Biobank, doubly-ranked method:

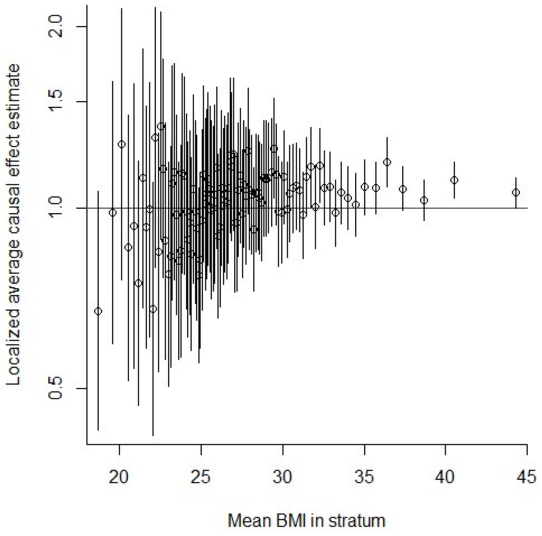

C) HUNT, residual method:

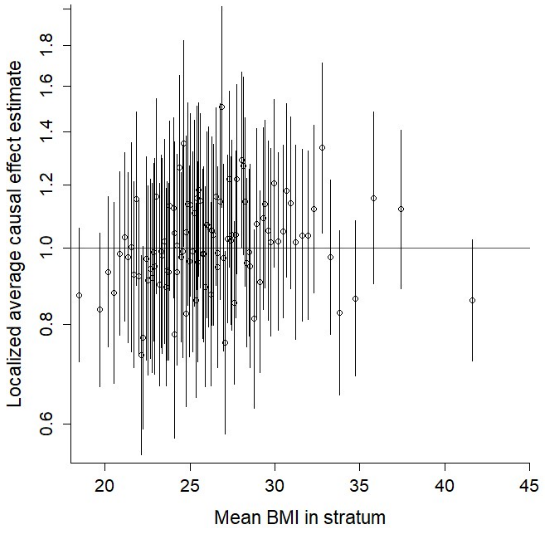

D) UK Biobank, residual method:

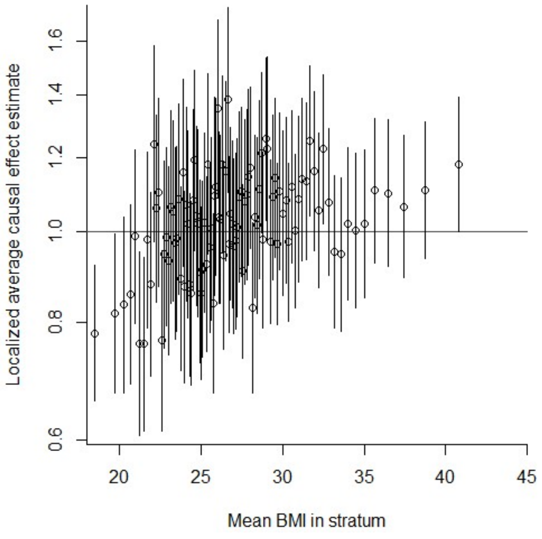

This presentation does not smooth over stratum-specific estimates, hence providing a representation of the causal effect of BMI which is not dependent on the choice of fractional polynomial smoothing. However, stratum-specific estimates are imprecise and variable, as they each only use 1% of the overall sample size. These plots differ from the presentation in the main text Figures: here, we plot the stratum-specific Mendelian randomization estimates, which (when log-transformed) reflect the slope of the curves in the main text figures.

Supplementary Figure 4: Non-linear Mendelian randomization – dose-response curve between body mass index and all-cause mortality using the doubly-ranked method with inverse-normal rank transformed BMI values in UK Biobank. Grey lines represent 95% confidence intervals. The reference value for BMI was taken as the median of the BMI distribution at 26.7 kg/m^2.

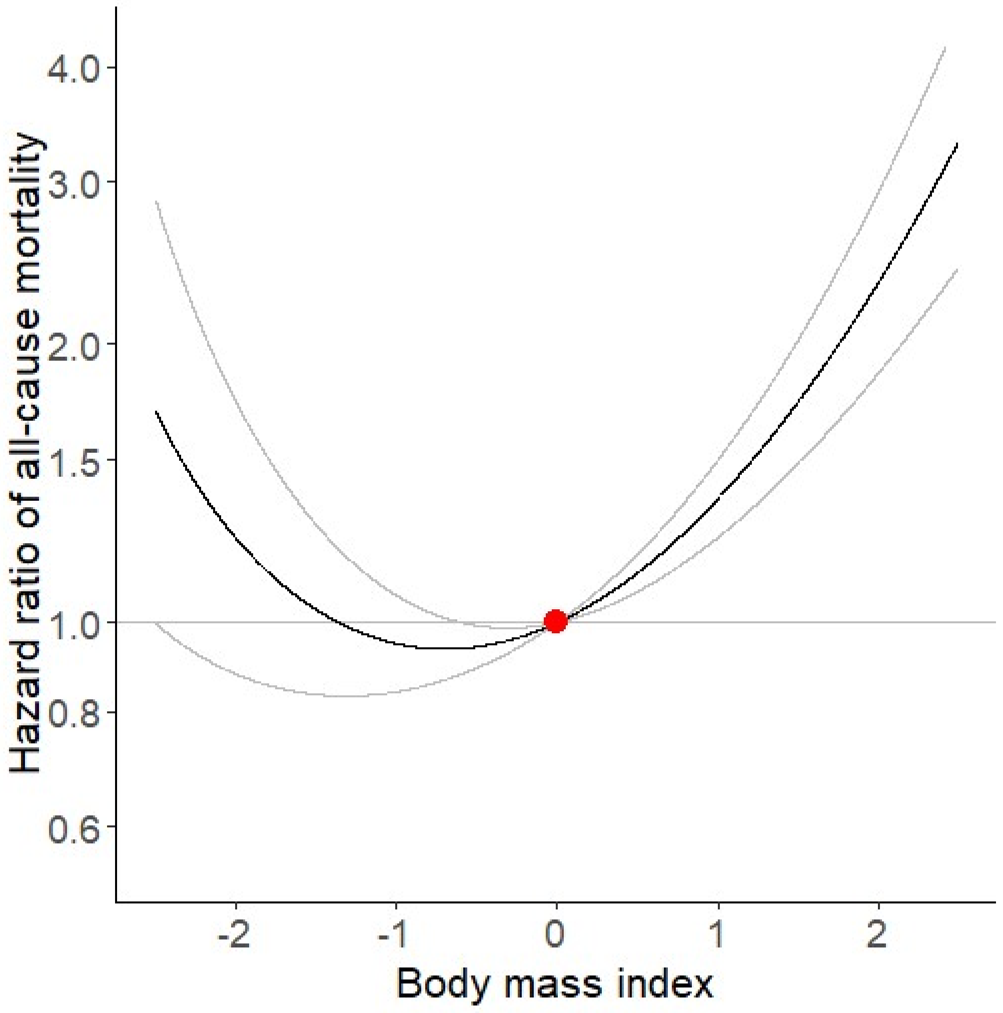

The labels on the horizontal axis represent the number of standard deviations below or above the median of the BMI distribution: for example, -1 represents one standard deviation below the median, and +2 represents two standard deviations above the median.

The reference values are:
-2 = 19.9 kg/m^2
-1 = 23.0 kg/m^2
0 = 26.7 kg/m^2
+1 = 31.6 kg/m^2
+2 = 39.2 kg/m^2

For peer review only - http://bmjopen.bmj.com/site/about/guidelines.xhtml

Supplementary Figure 5: Non-linear Mendelian randomization – dose-response curve between body mass index and all-cause mortality estimated using the doubly-ranked method: A) HUNT, B) UK Biobank dividing into 50 strata; C) HUNT, D) UK Biobank dividing into 200 strata. Gradient at each point of the curve is the localized average causal effect. Grey lines represent 95% confidence intervals. The reference value for BMI was taken as 25 kg/m^2.

A) HUNT, 50 strata:

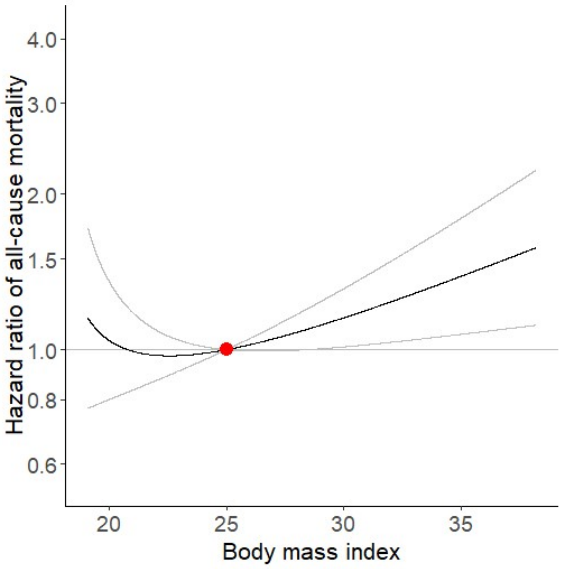

p-value for linearity: 0.50
p-value for trend: 0.49

B) UK Biobank, 50 strata:

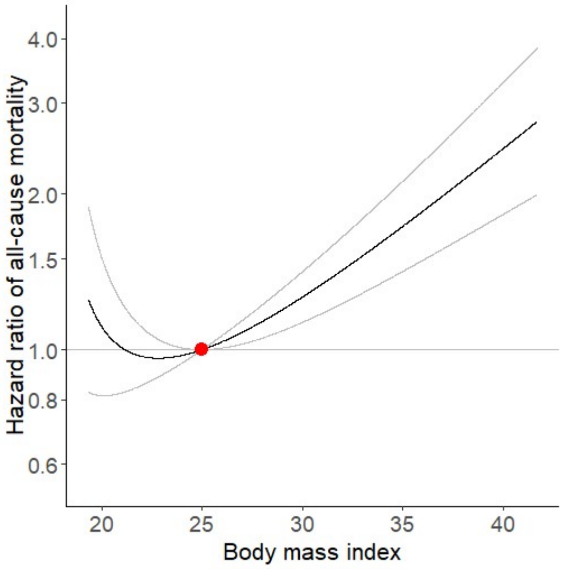

p-value for linearity: 0.10
p-value for trend: 0.09

C) HUNT, 200 strata:

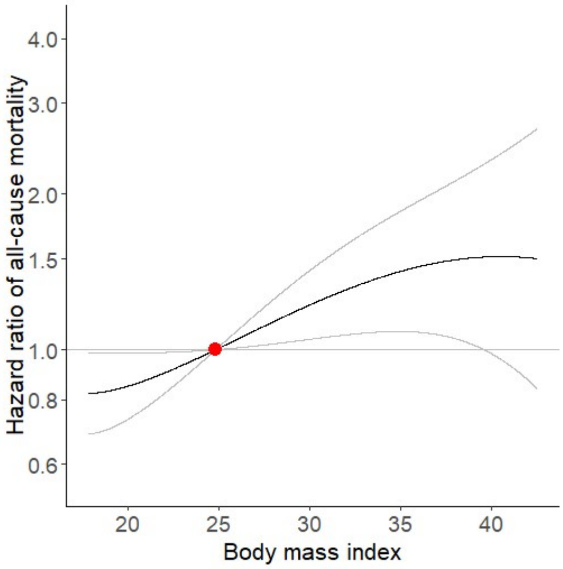

p-value for linearity: 0.99
p-value for trend: 0.88

D) UK Biobank, 200 strata:

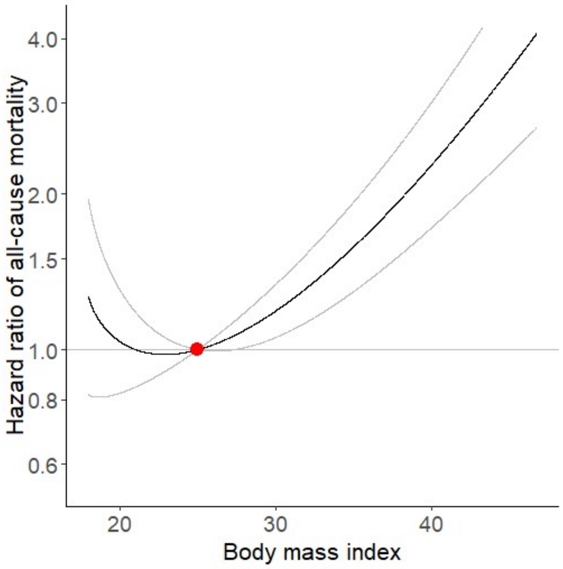

p-value for linearity: 0.28
p-value for trend: 0.16

For peer review only - http://bmjopen.bmj.com/site/about/guidelines.xhtml

Supplementary Figure 6: Non-linear Mendelian randomization – dose-response curve between body mass index and all-cause mortality estimated using the doubly-ranked method and the extended genetic score in UK Biobank. Gradient at each point of the curve is the localized average causal effect. Grey lines represent 95% confidence intervals. The reference value for BMI was taken as 25 kg/m^2.

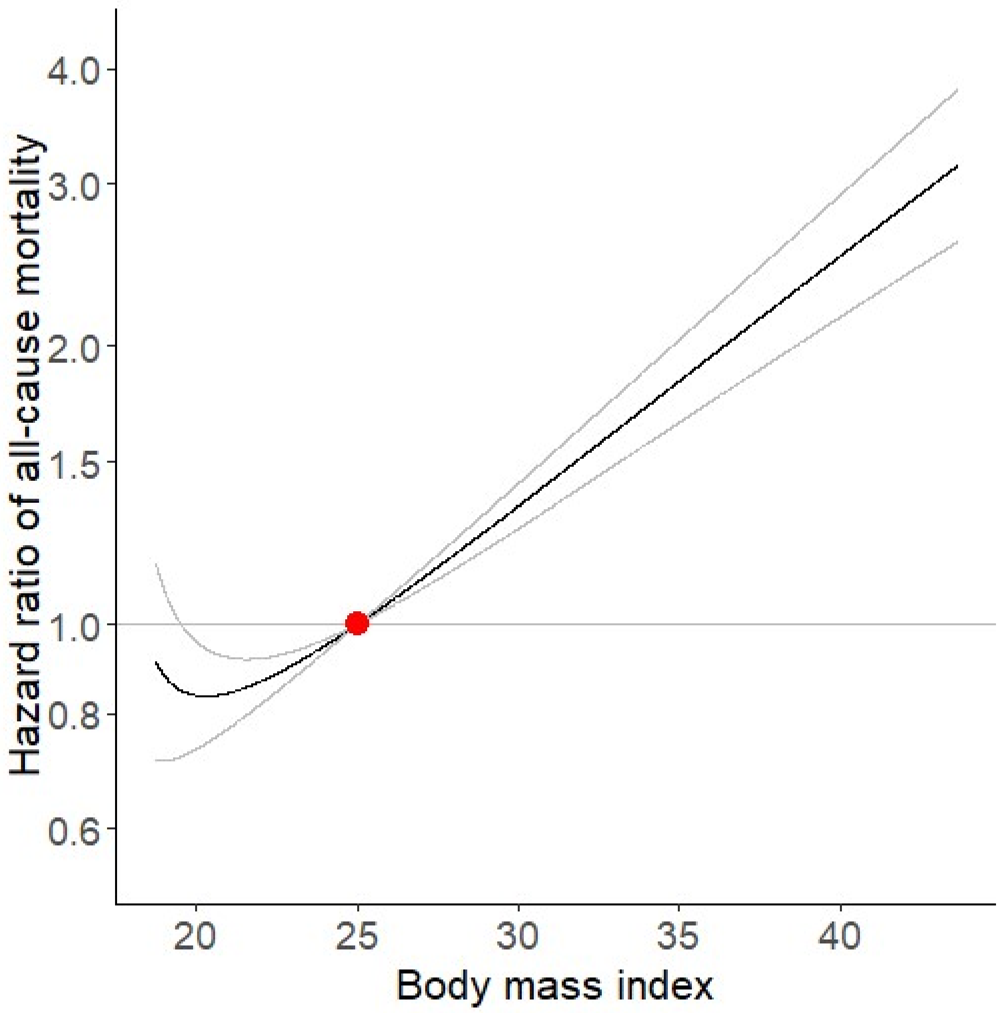

p-value for linearity: 0.99
p-value for trend: 0.32

Supplementary Figure 7: Localized average causal effect estimates (stratum-specific estimates) for all-cause mortality plotted against mean BMI in each stratum estimated in men and women separately. Estimates represent hazard ratios per 1 kg/m^2 increase in genetically-predicted BMI. Error bars represent 95% confidence intervals.

A) HUNT, men only:

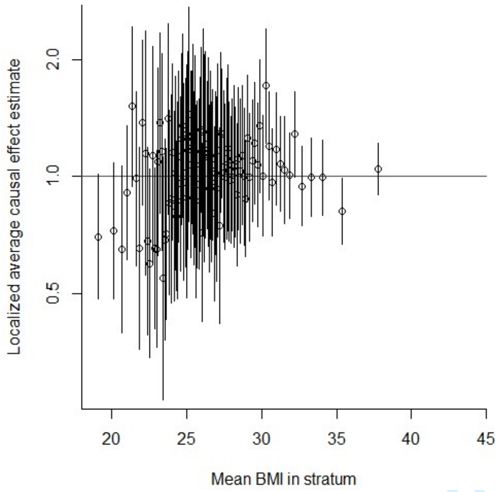

B) HUNT, women only:

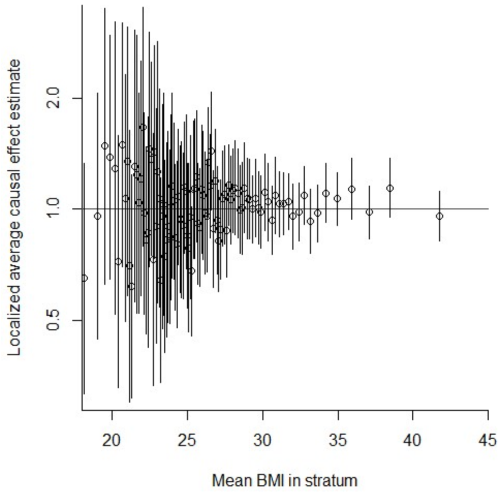

C) UK Biobank, men only:

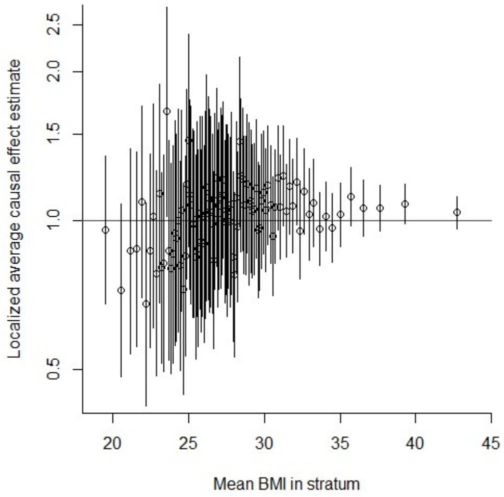

D) UK Biobank, women only:

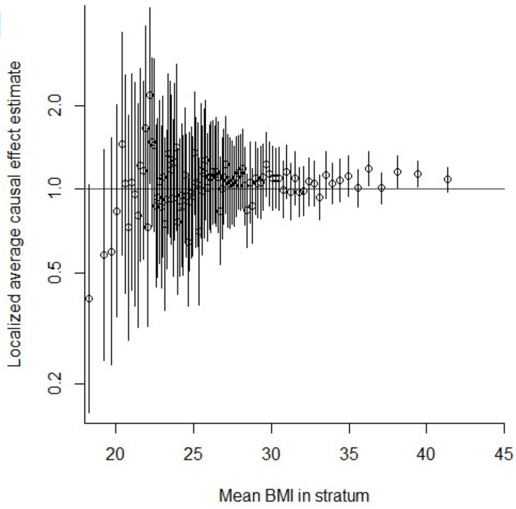

For peer review only - http://bmjopen.bmj.com/site/about/guidelines.xhtml

Supplementary Figure 8: Localized average causal effect estimates (stratum-specific estimates) for cause-specific mortality plotted against mean BMI in each stratum. Estimates represent hazard ratios per 1 kg/m^2 increase in genetically-predicted BMI. Error bars represent 95% confidence intervals.

A: Cardiovascular mortality

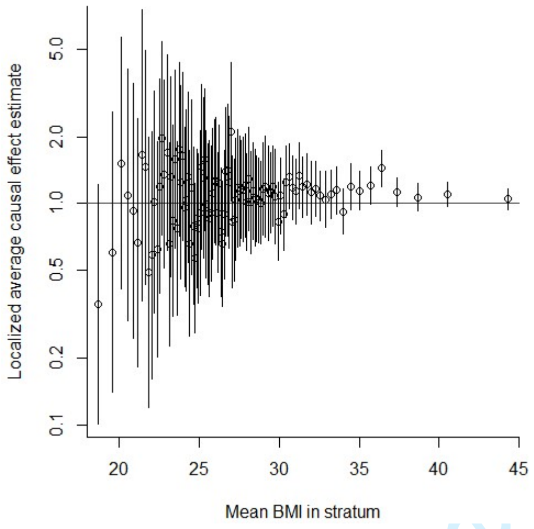

B: Cancer mortality

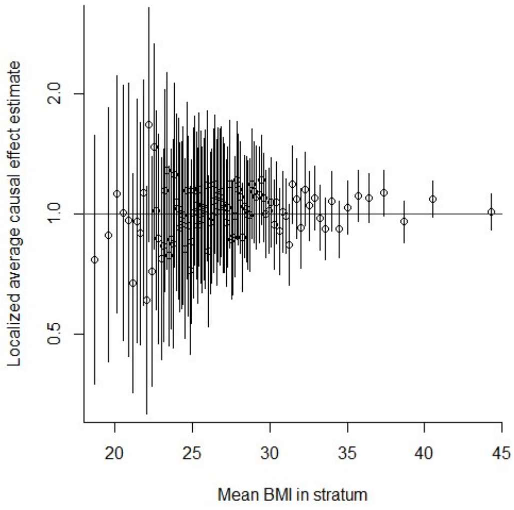

C: Other (non-cardiovascular, non-cancer) mortality

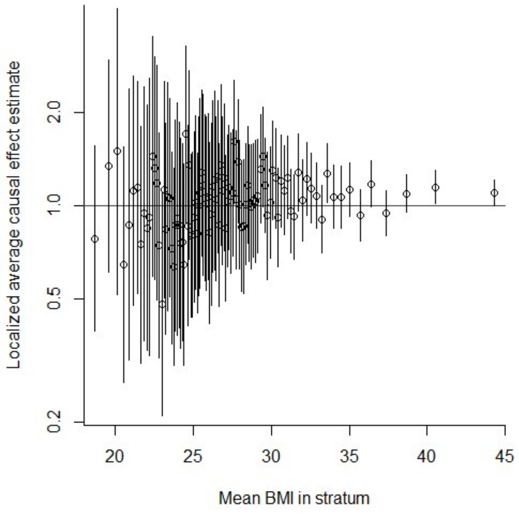

For peer review only - http://bmjopen.bmj.com/site/about/guidelines.xhtml

STROBE-MR checklist of recommended items to address in reports of Mendelian randomization studies[1 2]

Item No.	Section	Checklist item	Page No.	Relevant text from manuscript
1	**TITLE and ABSTRACT**	Indicate Mendelian randomization (MR) as the study's design in the title and/or the abstract if that is a main purpose of the study	1-2	Title: "Body mass index and all-cause mortality in HUNT and UK Biobank studies: revised non-linear Mendelian randomization analyses"<br>Abstract: "**Objectives**: To estimate the shape of the causal relationship between body mass index (BMI) and mortality risk in a Mendelian randomization framework."
	**INTRODUCTION**			
2	**Background**	Explain the scientific background and rationale for the reported study. What is the exposure? Is a potential causal relationship between exposure and outcome plausible? Justify why MR is a helpful method to address the study question	2,4	Abstract: "To estimate the shape of the causal relationship between body mass index (BMI) and mortality risk in a Mendelian randomization framework."<br>Introduction: "The epidemiological relationship between BMI and mortality is complex, with many observational studies conducted in Western countries showing a J-shaped relationship between BMI and mortality risk."<br>"However, such findings may not reflect the causal relationship between BMI and mortality, as observation association are influenced by confounding and reverse causation."
3	**Objectives**	State specific objectives clearly, including pre-specified causal hypotheses (if any). State that MR is a method that, under specific assumptions, intends to estimate causal effects	2,4	Abstract: "To estimate the shape of the causal relationship between body mass index (BMI) and mortality risk in a Mendelian randomization framework."<br>Introduction: "We first investigate whether the constant genetic effect assumption holds for this example, and explore the validity of the instrumental variable assumptions in strata of the population. We then present non-linear Mendelian randomization findings from the doubly-ranked stratification method."<br>"Hence, associations between genetic predictors of BMI and mortality provide insights into the causal effect of BMI on mortality."

For peer review only - http://bmjopen.bmj.com/site/about/guidelines.xhtml

	**METHODS**				
4	**Study design and data sources**		Present key elements of the study design early in the article. Consider including a table listing sources of data for all phases of the study. For each data source contributing to the analysis, describe the following:		
		a)	Setting: Describe the study design and the underlying population, if possible. Describe the setting, locations, and relevant dates, including periods of recruitment, exposure, follow-up, and data collection, when available.	6	See sections "The HUNT study" and "The UK Biobank study" (some of this detail is in reference 19, as indicated: "We present abbreviated descriptions of the datasets included in the analysis; detailed descriptions are in the original paper [19]").
		b)	Participants: Give the eligibility criteria, and the sources and methods of selection of participants. Report the sample size, and whether any power or sample size calculations were carried out prior to the main analysis	6	See sections "The HUNT study" and "The UK Biobank study" (detail is in reference 19: "We present abbreviated descriptions of the datasets included in the analysis; detailed descriptions are in the original paper [19]").
		c)	Describe measurement, quality control and selection of genetic variants	6	See section "SNPs and genetic score used as instrumental variables"
		d)	For each exposure, outcome, and other relevant variables, describe methods of assessment and diagnostic criteria for diseases	NA	See reference 19 for detail.
		e)	Provide details of ethics committee approval and participant informed consent, if relevant	7	See section "Ethical Approval" and "Patient Involvement"
5	**Assumptions**		Explicitly state the three core IV assumptions for the main analysis (relevance, independence and exclusion restriction) as well assumptions for any additional or sensitivity analysis	4,6	Introduction: "According to Mendel's laws of inheritance, genetic variants should be uncorrelated with traits that they do not affect, and hence should be independent of potential confounders"<br>Methods: "We assessed the instrumental variable assumptions by estimating associations between the genetic score and various traits that are competing risk factors in UK Biobank: smoking status (ever versus never), alcohol status (current versus other), education level (post-secondary or higher versus other), occupation (currently employed versus other), age at recruitment, and sex."
6	**Statistical methods: main analysis**		Describe statistical methods and statistics used	6-7	See Section "Statistical analyses".

For peer review only - http://bmjopen.bmj.com/site/about/guidelines.xhtml

		a)	Describe how quantitative variables were handled in the analyses (i.e., scale, units, model)	7	See Section "Statistical analyses".
		b)	Describe how genetic variants were handled in the analyses and, if applicable, how their weights were selected	6	See Section "SNPs and genetic score used as instrumental variables"
		c)	Describe the MR estimator (e.g. two-stage least squares, Wald ratio) and related statistics. Detail the included covariates and, in case of two-sample MR, whether the same covariate set was used for adjustment in the two samples	7	See Section "Statistical analyses":<br>"We then calculated the linear Mendelian randomization estimate, referred to as a localized average causal effect (LACE), in each stratum of the population as a ratio of coefficients: the association of the genetic score with the outcome divided by the associations of the genetic score with the exposure. associations with the exposure (BMI) were obtained from linear regression; associations with the outcome (mortality) were obtained from Cox proportional hazards regression, using age as the timescale. All associations were adjusted for age, age-squared, sex, and centre (for UK Biobank)."
		d)	Explain how missing data were addressed	6	"We excluded participants without data on BMI or genetic variants for BMI, leaving 56,150 individuals for analysis."
		e)	If applicable, indicate how multiple testing was addressed	N/A	We did not account for multiple testing, as the presentation of p-values is not a major aspect of the manuscript.
7	**Assessment of assumptions**		Describe any methods or prior knowledge used to assess the assumptions or justify their validity	6	See Section "SNPs and genetic score used as instrumental variables":<br>"We assessed the instrumental variable assumptions by estimating associations between the genetic score and various traits that are competing risk factors in UK Biobank: smoking status (ever versus never), alcohol status (current versus other), education level (post-secondary versus other), occupations (currently employed versus other), age at recruitment, and sex.
8	**Sensitivity analyses and additional analyses**		Describe any sensitivity analyses or additional analyses performed (e.g. comparison of effect estimates from different approaches, independent replication, bias analytic techniques, validation of instruments, simulations)	7	Sensitivity Analyses: "In brief, first we divided the sample into 100 strata using the doubly-ranked method. For comparison, we also present results from stratification using the residual method for the primary outcome of all-cause mortality."<br>Additional analyses: Testing for linearity:

For peer review only - http://bmjopen.bmj.com/site/about/guidelines.xhtml

					"We report two tests for non-linearity: a linearity test, which assesses whether a non-linear model fits the LACE estimates better than a linear model, and a trend test, which tests for a linear trend amongst the LACE estimates. "
9	**Software and pre-registration**				
		a)	Name statistical software and package(s), including version and settings used	7	"All statistical analyses were performed in R (version 4.3.1), and non-linear Mendelian randomisation analyses were performed using the SUMnlmr package [25]."
		b)	State whether the study protocol and details were pre-registered (as well as when and where)	N/A	The study protocol was not pre-registered.
	**RESULTS**				
10	**Descriptive data**				
		a)	Report the numbers of individuals at each stage of included studies and reasons for exclusion. Consider use of a flow diagram		All numbers provided in Table 1.
		b)	Report summary statistics for phenotypic exposure(s), outcome(s), and other relevant variables (e.g. means, SDs, proportions)		All numbers provided in Table 1.
		c)	If the data sources include meta-analyses of previous studies, provide the assessments of heterogeneity across these studies		N/A – we present results from the HUNT and UK Biobank studies separately, and do not attempt to meta-analyse or otherwise combine results.
		d)	For two-sample MR:<br>i. Provide justification of the similarity of the genetic variant-exposure associations between the exposure and outcome samples<br>ii. Provide information on the number of individuals who overlap between the exposure and outcome studies		N/A – not two-sample MR.
11	**Main results**				
		a)	Report the associations between genetic variant and exposure, and between genetic variant and outcome, preferably on an interpretable scale		Genetic associations with exposure are shown in Supplementary Figure 1.<br>Scaled genetic association with outcome are shown in Supplementary Figures 3, 5, and 6.
		b)	Report MR estimates of the relationship between exposure and outcome, and the measures of uncertainty from the MR analysis, on an interpretable scale, such as odds ratio or relative risk per SD difference		MR estimates are shown directly in Supplementary Figures 3, 5, and 6. Smoothed versions of these associations are shown in Figures 2, 3, and 4.

For peer review only - http://bmjopen.bmj.com/site/about/guidelines.xhtml

		c)	If relevant, consider translating estimates of relative risk into absolute risk for a meaningful time period		N/A
		d)	Consider plots to visualize results (e.g. forest plot, scatterplot of associations between genetic variants and outcome versus between genetic variants and exposure)		N/A
12	**Assessment of assumptions**				
		a)	Report the assessment of the validity of the assumptions	8	See section "Assessment of instrument validity"
		b)	Report any additional statistics (e.g., assessments of heterogeneity across genetic variants, such as I^2, Q statistic or E-value)	6	F statistics are provided in the Methods section under "SNPs and genetic score used as instrumental variables.<br>Nonlinear tests are reported in Figures 2, 3, and 4.
13	**Sensitivity analyses and additional analyses**				
		a)	Report any sensitivity analyses to assess the robustness of the main results to violations of the assumptions		See Figure 2: residual versus ranked stratification.
		b)	Report results from other sensitivity analyses or additional analyses		Figure 2: residual versus ranked stratification<br>Figure 3: results stratified by sex<br>Figure 4: exploring different cause-specific mortality subtypes.
		c)	Report any assessment of direction of causal relationship (e.g., bidirectional MR)		N/A, directionality not considered.
		d)	When relevant, report and compare with estimates from non-MR analyses	11	In Discussion, paragraph beginning "Previous investigations into the non-linear shape of the causal relationship between BMI and mortality have used a variety of approaches." and in particular "Carslake *et al* performed an instrumental variable analysis to investigate the impact of BMI on mortality in the HUNT study, but using offspring BMI as an instrument for the BMI".
		e)	Consider additional plots to visualize results (e.g., leave-one-out analyses)		N/A
	**DISCUSSION**				
14	**Key results**		Summarize key results with reference to study objectives	10	Results are summarized in the Discussion section:

For peer review only - http://bmjopen.bmj.com/site/about/guidelines.xhtml

					"While the curves generally display a J-shaped relationship, evidence for a harmful average effect of decreased BMI on mortality at low BMI levels was limited. Evidence supporting non-linearity from the doubly-ranked method was unconvincing for all mortality outcomes." Also see Abstract / Results.
15	**Limitations**		Discuss limitations of the study, taking into account the validity of the IV assumptions, other sources of potential bias, and imprecision. Discuss both direction and magnitude of any potential bias and any efforts to address them	11-12	Limitations are clearly discussed in the Discussion section, paragraph beginning "However, there are important limitations…" Several potential limitations are discussed, including validity of instruments, pleiotropy, bias due to selection bias, representativeness, and reverse causation.
16	**Interpretation**				
		a)	Meaning: Give a cautious overall interpretation of results in the context of their limitations and in comparison with other studies	10	Interpretation of results is provided in the Discussion section: "In conclusion, non-linear Mendelian randomization analyses using the doubly-ranked stratification method provide strong evidence for harmful effects of increased BMI on mortality above 25 kg/m2. Evidence for a harmful effect of low BMI was only present in some analyses, and where present, only below 20 kg/m2.". Also see Abstract/Conclusion.
		b)	Mechanism: Discuss underlying biological mechanisms that could drive a potential causal relationship between the investigated exposure and the outcome, and whether the gene-environment equivalence assumption is reasonable. Use causal language carefully, clarifying that IV estimates may provide causal effects only under certain assumptions		N/A (mechanisms linking BMI and mortality have been discussed elsewhere at length, and evidence for specific mechanisms is not provided here).
		c)	Clinical relevance: Discuss whether the results have clinical or public policy relevance, and to what extent they inform effect sizes of possible interventions	10	Discussion: "Our findings challenge the "obesity paradox" consensus from observational epidemiology that increases in BMI might reduce risk of mortality for a substantial proportion of the population."
17	**Generalizability**		Discuss the generalizability of the study results (a) to other populations, (b) across other exposure periods/timings, and (c) across other levels of exposure	11	Discussion / Limitations: "Our findings may therefore not be applicable to other populations. "

For peer review only - http://bmjopen.bmj.com/site/about/guidelines.xhtml

	**OTHER INFORMATION**			
18	**Funding**	Describe sources of funding and the role of funders in the present study and, if applicable, sources of funding for the databases and original study or studies on which the present study is based	1	See “Funding statement”
19	**Data and data sharing**	Provide the data used to perform all analyses or report where and how the data can be accessed, and reference these sources in the article. Provide the statistical code needed to reproduce the results in the article, or report whether the code is publicly accessible and if so, where	31	See “Data sharing statement”
20	**Conflicts of Interest**	All authors should declare all potential conflicts of interest	1	Provided under “Competing interest statement”

This checklist is copyrighted by the Equator Network under the Creative Commons Attribution 3.0 Unported (CC BY 3.0) license.

1. Skrivankova VW, Richmond RC, Woolf BAR, Yarmolinsky J, Davies NM, Swanson SA, et al. Strengthening the Reporting of Observational Studies in Epidemiology using Mendelian Randomization (STROBE-MR) Statement. JAMA. 2021;under review.

2. Skrivankova VW, Richmond RC, Woolf BAR, Davies NM, Swanson SA, VanderWeele TJ, et al. Strengthening the Reporting of Observational Studies in Epidemiology using Mendelian Randomisation (STROBE-MR): Explanation and Elaboration. BMJ. 2021;375:n2233.

For peer review only
